# Supplementary material for: Cyclohumulanoid Sesquiterpenes from the Culture Broth of the Basidiomycetous Fungus Daedaleopsis tricolor
Source: Molecules. 2021 Jul 19;26(14):4364. doi: 10.3390/molecules26144364 (PMC8303491; doi:10.3390/molecules26144364)
Supplement: Supplementary file 1 [file molecules-26-04364-s001.zip › Supporting Informatiomolecules-126864.pdf]

Supporting Information

Cyclohumulanoid Sesquiterpenes from the Culture Broth of the Basidiomycetous Fungus *Daedaleopsis tricolor*

Ryuhi Kanehara, Akio Tonouchi, Katsuhiro Konno, Masaru Hashimoto*

Index

IR spectrum of **1a** (film) SI-5
ESI-TOFMS spectrum of **1a** SI-6
^{1}H NMR spectrum of **1a** (500 MHz, $CDCl_3$) SI-7
^{13}C NMR spectrum of **1a** (125 MHz, $CDCl_3$) SI-8
DQF COSY spectrum of **1a** (500 MHz, $CDCl_3$) SI-9
HSQC spectrum of **1a** (500 MHz, $CDCl_3$) SI-10
HMBC spectrum of **1a** (500 MHz, $CDCl_3$) SI-11
NOE 1D spectra of **1a** (500 MHz, $CDCl_3$) SI-12
NOESY spectrum of **1a** (500 MHz, $CDCl_3$) SI-13
IR spectrum of **1b** (film) SI-14
ESI-TOFMS spectrum of **1b** SI-15
^{1}H NMR spectrum of **1b** (500 MHz, $CDCl_3$) SI-16
^{13}C NMR spectrum of **1b** (125 MHz, $CDCl_3$) SI-17
DQF COSY spectrum of **1b** (500 MHz, $CDCl_3$) SI-18
HSQC spectrum of **1b** (500 MHz, $CDCl_3$) SI-19
HMBC spectrum of **1b** (500 MHz, $CDCl_3$) SI-20
NOESY spectrum of **1b** (500 MHz, $CDCl_3$) SI-21
IR spectrum of **1c** (film) SI-22
ESI-TOFMS spectrum of **1c** SI-23
^{1}H NMR spectrum of **1c** (500 MHz, acetone-d_6) SI-24
^{13}C NMR spectrum of **1c** (125 MHz, acetone-d_6) SI-25
DQF COSY spectrum of **1c** (500 MHz, acetone-d_6) SI-26
HSQC spectrum of **1c** (500 MHz, acetone-d_6) SI-27
HMBC spectrum of **1c** (500 MHz, acetone-d_6) SI-28
NOESY spectrum of **1c** (500 MHz, acetone-d_6) SI-29
IR spectrum of **2a** (film) SI-30
ESI-TOFMS spectrum of **2a** SI-31
^{1}H NMR spectrum of **2a** (500 MHz, $CDCl_3$) SI-32
^{13}C NMR spectrum of **2b** (125 MHz, $CDCl_3$) SI-33
DQF COSY spectrum of **2a** (500 MHz, $CDCl_3$) SI-34
HSQC spectrum of **2a** (500 MHz, $CDCl_3$) SI-35
HMBC spectrum of **2a** (500 MHz, $CDCl_3$) SI-36
IR spectrum of **2b** (film) SI-37
ESI-TOFMS spectrum of **2b** SI-38
^{1}H NMR spectrum of **2b** (500 MHz, $CDCl_3$) SI-39
^{13}C NMR spectrum of **2b** (125 MHz, $CDCl_3$) SI-40
NOE 1D spectra of **2b** (500 MHz, $CDCl_3$) SI-41
IR spectrum of **3** (film) SI-42
ESI-TOFMS spectrum of **3** SI-43

^{1}H NMR spectrum of **3** (500 MHz, $CDCl_3$) SI-44
^{13}C NMR spectrum of **3** (125 MHz, $CDCl_3$) SI-45
DQF COSY spectrum of **3** (500 MHz, $CDCl_3$) SI-46
HSQC spectrum of **3** (500 MHz, $CDCl_3$) SI-47
HMBC spectrum of **3** (500 MHz, $CDCl_3$) SI-48
NOESY spectrum of **3** (500 MHz, $CDCl_3$) SI-49
IR spectrum of **4** (film) SI-50
ESI-TOFMS spectrum of **4** SI-51
^{1}H NMR spectrum of **4** (500 MHz, $CDCl_3$) SI-52
^{13}C NMR spectrum of **4** (125 MHz, $CDCl_3$) SI-53
DQF COSY spectrum of **4** (500 MHz, $CDCl_3$) SI-54
HSQC spectrum of **4** (500 MHz, $CDCl_3$) SI-55
HMBC spectrum of **4** (500 MHz, $CDCl_3$) SI-56
NOE 1D spectra of **4** (500 MHz, $CDCl_3$) SI-57
NOESY spectrum of **4** (500 MHz, $CDCl_3$) SI-58
IR spectrum of **5** (film) SI-59
ESI-TOFMS spectrum of **5** SI-60
^{1}H NMR spectrum of **5** (500 MHz, $CDCl_3$) SI-61
^{13}C NMR spectrum of **5** (125 MHz, $CDCl_3$) SI-62
DQF COSY spectrum of **5** (500 MHz, $CDCl_3$) SI-63
HMQC spectrum of **5** (500 MHz, $CDCl_3$) SI-64
HMBC spectrum of **5** (500 MHz, $CDCl_3$) SI-65
NOE 1D spectra of **5** (500 MHz, $CDCl_3$) SI-66
IR spectrum of **6** (film) SI-67
ESI-TOFMS spectrum of **6** SI-68
^{1}H NMR spectrum of **6** (500 MHz, $CDCl_3$) SI-69
^{13}C NMR spectrum of **6** (125 MHz, $CDCl_3$) SI-70
DQF COSY spectrum of **6** (500 MHz, $CDCl_3$) SI-71
HSQC spectrum of **6** (500 MHz, $CDCl_3$) SI-72
HMBC spectrum of **6** (500 MHz, $CDCl_3$) SI-73
NOE 1D spectra of **6** (500 MHz, $CDCl_3$) SI-74
NOESY spectrum of **6** (500 MHz, $CDCl_3$) SI-75
IR spectrum of 12-*O*-acetate of **6** (film) SI-76
ESI-TOFMS spectrum of 12-*O*-acetate of **6** SI-77
^{1}H NMR spectrum of 12-*O*-acetate of **6** (500 MHz, $CDCl_3$) SI-78
^{13}C NMR spectrum of 12-*O*-acetate of **6** (125 MHz, $CDCl_3$) SI-79
DQF COSY spectrum of 12-*O*-acetate of **6** (500 MHz, $CDCl_3$) SI-80
IR spectrum of **7** (film) SI-81
ESI-TOFMS spectrum of **7** SI-82
^{1}H NMR spectrum of **7** (500 MHz, $CDCl_3$) SI-83
^{13}C NMR spectrum of **7** (125 MHz, $CDCl_3$) SI-84
DQF COSY spectrum of **7** (500 MHz, $CDCl_3$) SI-85
HSQC spectrum of **7** (500 MHz, $CDCl_3$) SI-86
HMBC spectrum of **7** (500 MHz, $CDCl_3$) SI-87
NOESY spectrum of **7** (500 MHz, $CDCl_3$) SI-88
^{1}H NMR spectrum of **8** (500 MHz, $CDCl_3$) SI-89
^{13}C NMR spectrum of **8** (125 MHz, $CDCl_3$) SI-90
NOESY spectrum of **8** (500 MHz, $CDCl_3$) SI-91
IR spectrum of **8-OBz** (film) SI-92
ESI-TOFMS spectrum of **8-OBz** SI-93
^{1}H NMR spectrum of **8-OBz** (500 MHz, $CDCl_3$) SI-94
^{13}C NMR spectrum of **8-OBz** (125 MHz, $CDCl_3$) SI-95
HSQC spectrum of **8-OBz** (500 MHz, $CDCl_3$) SI-96
HMBC spectrum of **8-OBz** (500 MHz, $CDCl_3$) SI-97

Chemical shift calculations SI-98
Calculated ^{13}C NMR chemical shift of **1a** and its isomers SI-99
Calculated ^{1}H NMR chemical shift of **1a** and its isomers SI-99

Statistical analysis of $\delta^{13}C$ and $\delta^{1}H$ for **1a** and its isomers SI-100
Calculated ^{13}C NMR chemical shift of **1b** and its isomers SI-101
Calculated ^{1}H NMR chemical shift of **1b** and its isomers SI-102
Statistical analysis of $\delta^{13}C$ and $\delta^{1}H$ for **1b** and its isomers SI-103
Calculated ^{13}C NMR chemical shift of **1c** and its isomers SI-104
Calculated ^{1}H NMR chemical shift of **1c** and its isomers SI-104
Statistical analysis of $\delta^{13}C$ and $\delta^{1}H$ for **1c** and its isomers SI-105
Calculated ^{13}C NMR chemical shift of **2a** and its isomers SI-106
Calculated ^{1}H NMR chemical shift of **2a** and its isomers SI-106
Statistical analysis of $\delta^{13}C$ and $\delta^{1}H$ for **2a** and its isomers SI-107
Calculated ^{13}C NMR chemical shift of **2b** and its isomers SI-108
Calculated ^{1}H NMR chemical shift of **2b** and its isomers SI-108
Statistical analysis of $\delta^{13}C$ and $\delta^{1}H$ for **2b** and its isomers SI-109
Calculated ^{13}C NMR chemical shift of **3** and its isomers. SI-110
Calculated ^{1}H NMR chemical shift of **3** and its isomers. SI-111
Statistical analysis of $\delta^{13}C$ and $\delta^{1}H$ for **3** and its isomers SI-112
Calculated ^{13}C NMR chemical shift of **4** and its isomers SI-113
Calculated ^{1}H NMR chemical shift of **4** and its isomers SI-113
Statistical analysis of $\delta^{13}C$ and $\delta^{1}H$ for **4** and its isomers SI-114
Calculated ^{13}C NMR chemical shift of **5** and its isomers SI-115
Calculated ^{1}H NMR chemical shift of **5** and its isomers SI-115
Statistical analysis of $\delta^{13}C$ and $\delta^{1}H$ for **5** and its isomers SI-115
Calculated ^{13}C NMR chemical shift of **6** and its isomers SI-116
Calculated ^{1}H NMR chemical shift of **6** and its isomers SI-116
Statistical analysis of $\delta^{13}C$ and $\delta^{1}H$ for **6** and its isomers SI-117
Calculated ^{13}C NMR chemical shift of **7** and its isomers SI-118
Calculated ^{1}H NMR chemical shift of **7** and its isomers SI-118
Statistical analysis of $\delta^{13}C$ and $\delta^{1}H$ for **7** and its isomers SI-119
Calculated ^{13}C NMR chemical shift of **8** and its isomers SI-120
Calculated ^{1}H NMR chemical shift of **8** and its isomers SI-121
Statistical analysis of $\delta^{13}C$ and $\delta^{1}H$ for **8** and its isomers SI-122

ECD calculations SI-123
SCF energy (au), chemical potential (kJ/mol). relative free energy, and Boltzmann distribution of *ent*-**1a** based on B3LYP/def2-TZVP SI-124
Wavelength (nm) oscillator strength (UV), and rotatory strength (CD) of *ent*-**1a** based on B3LYP/def2-TZVP SI-124
Parameters used for ECD reproduction of *ent*-**1a** SI-125
SCF energy (au), chemical potential (kJ/mol). relative free energy, and Boltzmann distribution of *ent*-**1b** based on B3LYP/def2-TZVP SI-126
Wavelength (nm) oscillator strength (UV), and rotatory strength (CD) of **1b** based on B3LYP/def2-TZVP SI-126
Parameters used for ECD reproduction of *ent*-**1b** SI-128
SCF energy (au), chemical potential (kJ/mol). relative free energy, and Boltzmann distribution of *ent*-**1c** based on B3LYP/def2-TZVP SI-129
Wavelength (nm) oscillator strength (UV), and rotatory strength (CD) of **1c** based on B3LYP/def2-TZVP SI-129
Parameters used for ECD reproduction of *ent*-**1c** SI-131
Boltzmann distribution of **2a** based on B3LYP/def2-TZVP SI-132
Wavelength (nm) oscillator strength (UV), and rotatory strength (CD) of **2a** based on B3LYP/def2-TZVP SI-132
Parameters used for ECD reproduction of **2a** SI-133
SCF energy (au), chemical potential (kJ/mol). relative free energy, and Boltzmann distribution of **2b** based on B3LYP/def2-TZVP SI-134
Wavelength (nm) oscillator strength (UV), and rotatory strength (CD) of **2b** based on B3LYP/def2-TZVP SI-134
Parameters used for ECD reproduction of **2b** SI-135

SCF energy (au), chemical potential (kJ/mol). relative free energy, and
Boltzmann distribution of **3** based on BHLYP/def2-TZVP. SI-136
Wavelength (nm) oscillator strength (UV), and rotatory strength (CD)
of **3** based on BHLYP/def2-TZVP SI-136
Parameters used for ECD reproduction of **3** SI-137
SCF energy (au), chemical potential (kJ/mol). relative free energy, and
Boltzmann distribution of *ent*-**4** based on B3LYP/def2-TZVP SI-138
Wavelength (nm) oscillator strength (UV), and rotatory strength (CD)
of *ent*-**4** based on B3LYP/def2-TZVP SI-138
Parameters used for ECD reproduction of *ent*-**4** SI-140
SCF energy (au), chemical potential (kJ/mol). relative free energy, and
Boltzmann distribution of *ent*-**5** based on B3LYP/def2-TZVP SI-141
Wavelength (nm) oscillator strength (UV), and rotatory strength (CD)
of *ent*-**5** based on B3LYP/def2-TZVP SI-141
Parameters used for ECD reproduction of *ent*-**5** SI-142
SCF energy (au), chemical potential (kJ/mol). relative free energy, and
SCF energy (au), chemical potential (kJ/mol). relative free energy, and
Boltzmann distribution of **8-OBz** based on B3LYP/def2-TZVP SI-143
Wavelength (nm) oscillator strength (UV), and rotatory strength (CD)
of **8-OBz** based on B3LYP/def2-TZVP SI-143
Parameters used for ECD reproduction of **8-OBz** SI-144
Wavelength (nm) and rotatory strength (CD) of *ent*-model **I**, *ent*-model **II**, and
ent-model **III** based on B3LYP/def2-TZVP, and their calculated ECD spectra SI-145
Parameters used for ECD reproduction of *ent*-model **I**, *ent*-model **II**, and *ent*-model **III** SI-145
XYZ data of *ent*-model **I** used for reproduction of the ECD spectrum. SI-146
XYZ data of *ent*-model **II** used for reproduction of the ECD spectrum. SI-146
XYZ data of *ent*-model **III** used for reproduction of the ECD spectrum. SI-147
Wavelength (nm) oscillator strength (UV), and rotatory strength (CD) of model **IV**
based on B3LYP/def2-TZVP when dihedral angle ∠O/C-5/C-9/C-7 was set to15° SI-148
Parameters used for ECD reproduction of model **IV** SI-149
XYZ data of model **IV** used for reproduction of the ECD spectrum. SI-149
Wavelength (nm) and rotatory strength (CD) of model **V** based on B3LYP/def2-TZVP
when dihedral angle ∠C-8/C-9/C-10/O was set to180° SI-150
Parameters used for ECD reproduction of model **V** SI-150
XYZ data of model **V** used for reproduction of the ECD spectrum. SI-151
Calculated and experimental UV/ECD spectra of **3** with B3LYP/def2-TZVP SI-152
Hyphal growth inhibitions of *Cochliobolus miyabeanus* by **2a** and **5** at 5.0 μg/mL SI-153

IR spectrum of **1a** (film)

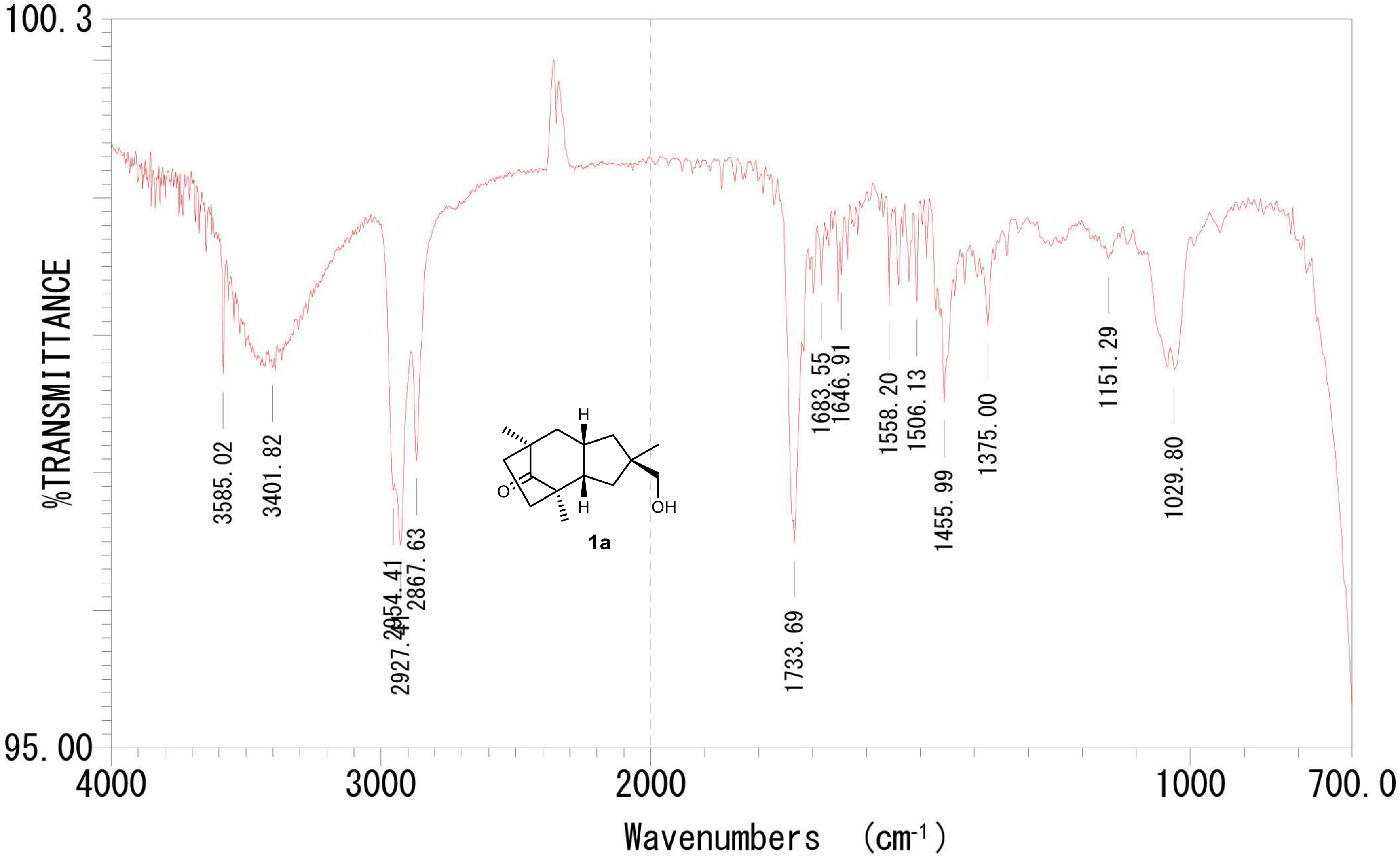

ESI-TOFMS spectrum of **1a**

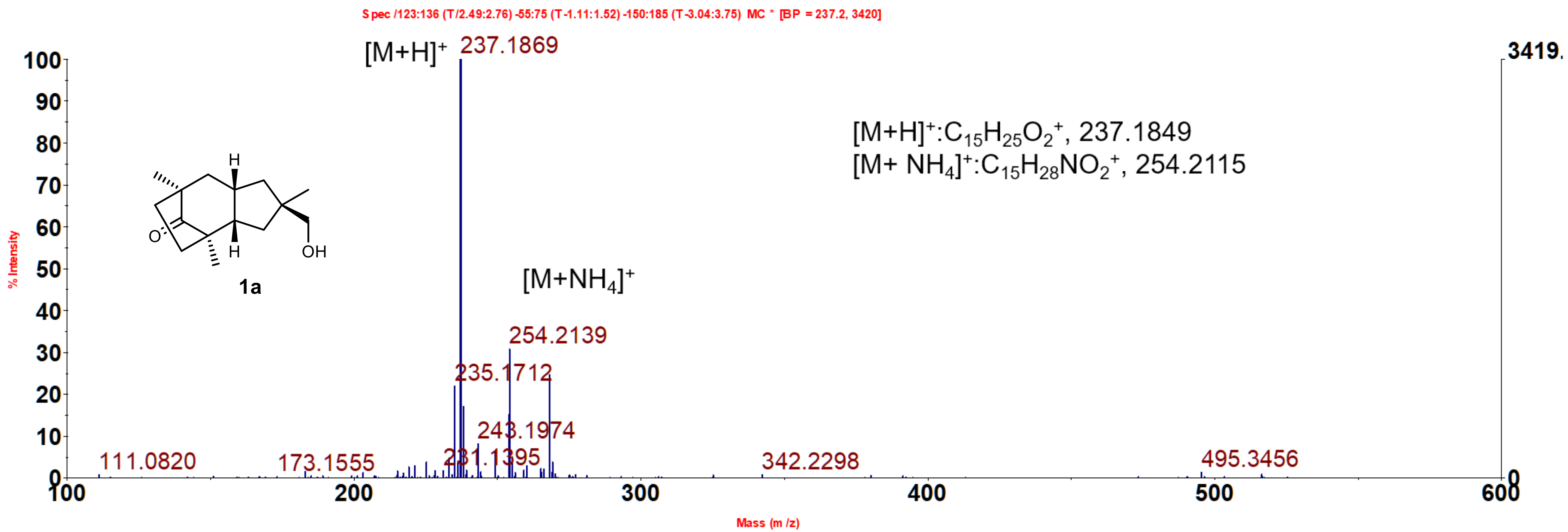

^{1}H NMR spectrum of **1a** (500 MHz, $CDCl_3$)

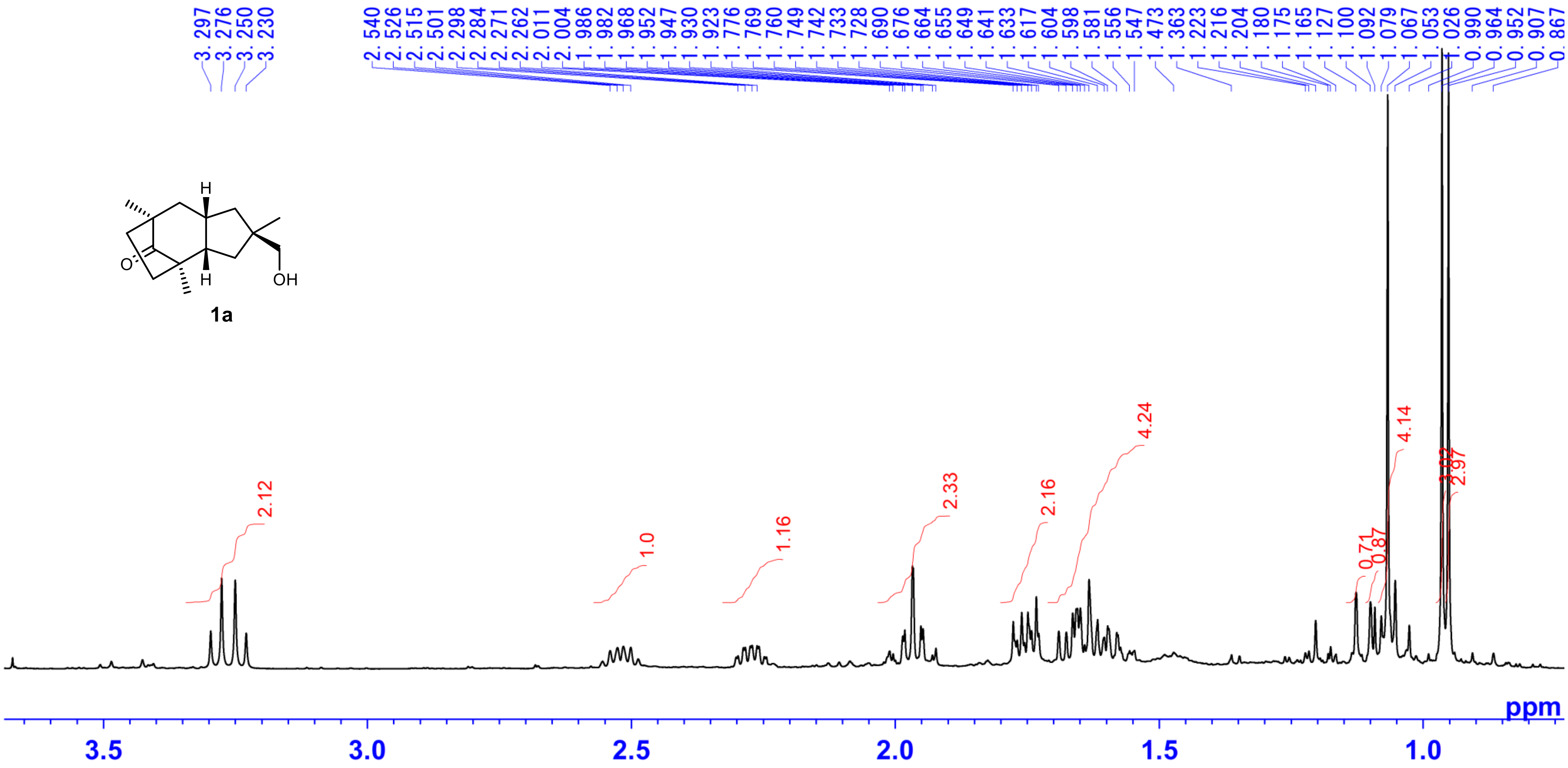

^{13}C NMR spectrum of **1a** (125 MHz, $CDCl_3$)

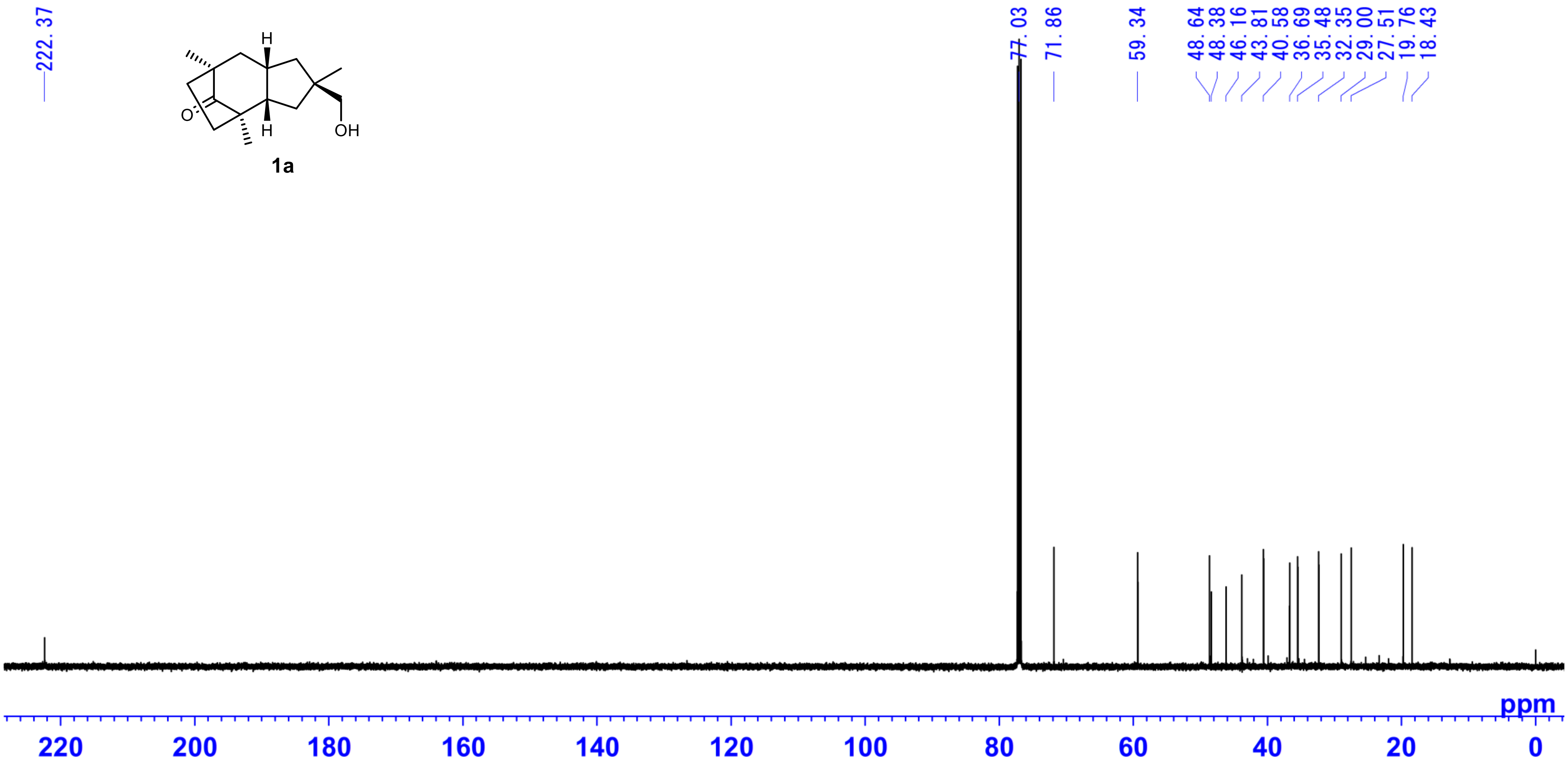

DQF COSY spectrum of **1a** (500 MHz, $CDCl_3$)

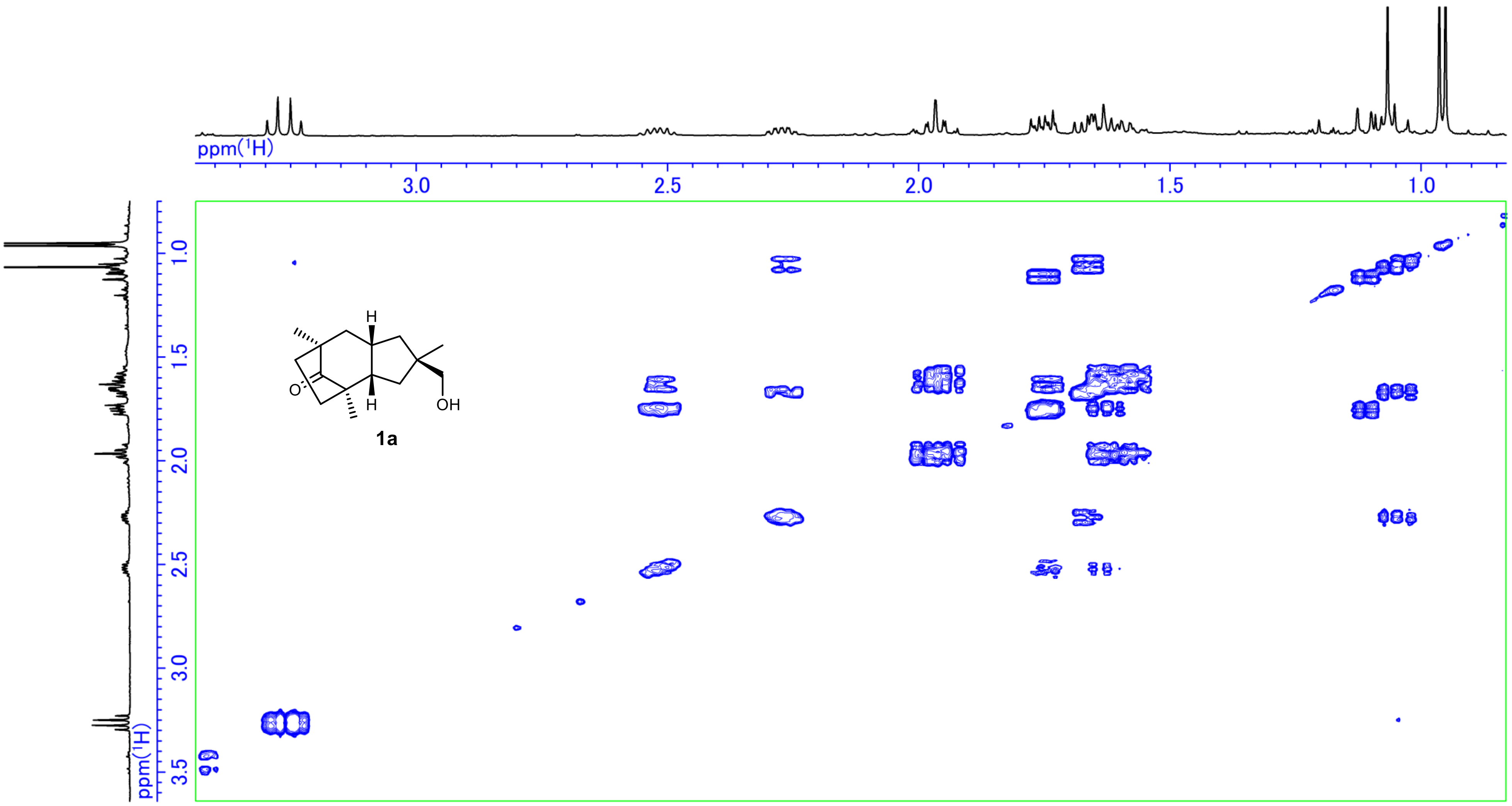

HSQC spectrum of **1a** (500 MHz, $CDCl_3$)

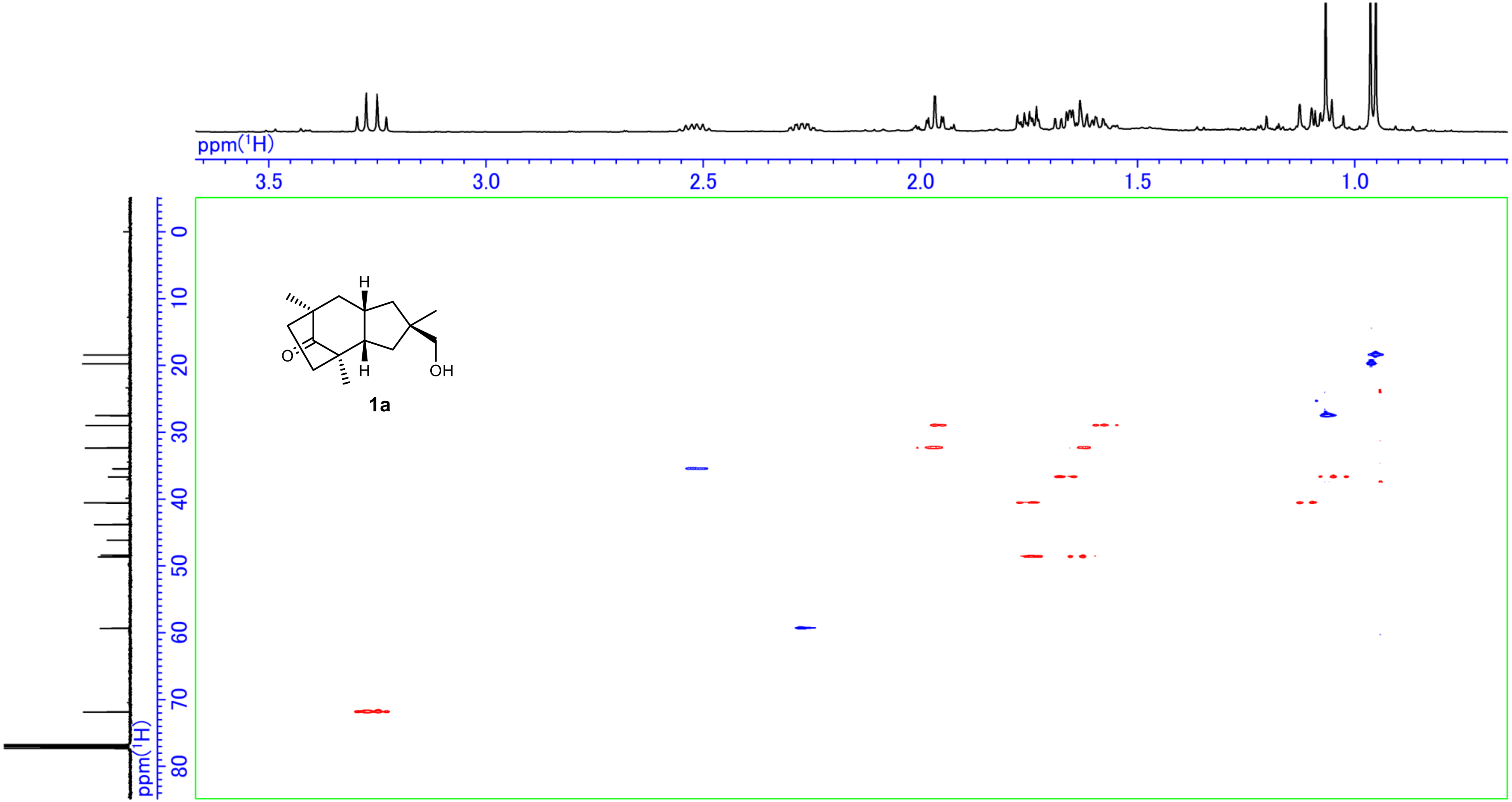

HMBC spectrum of **1a** (500 MHz, $CDCl_3$)

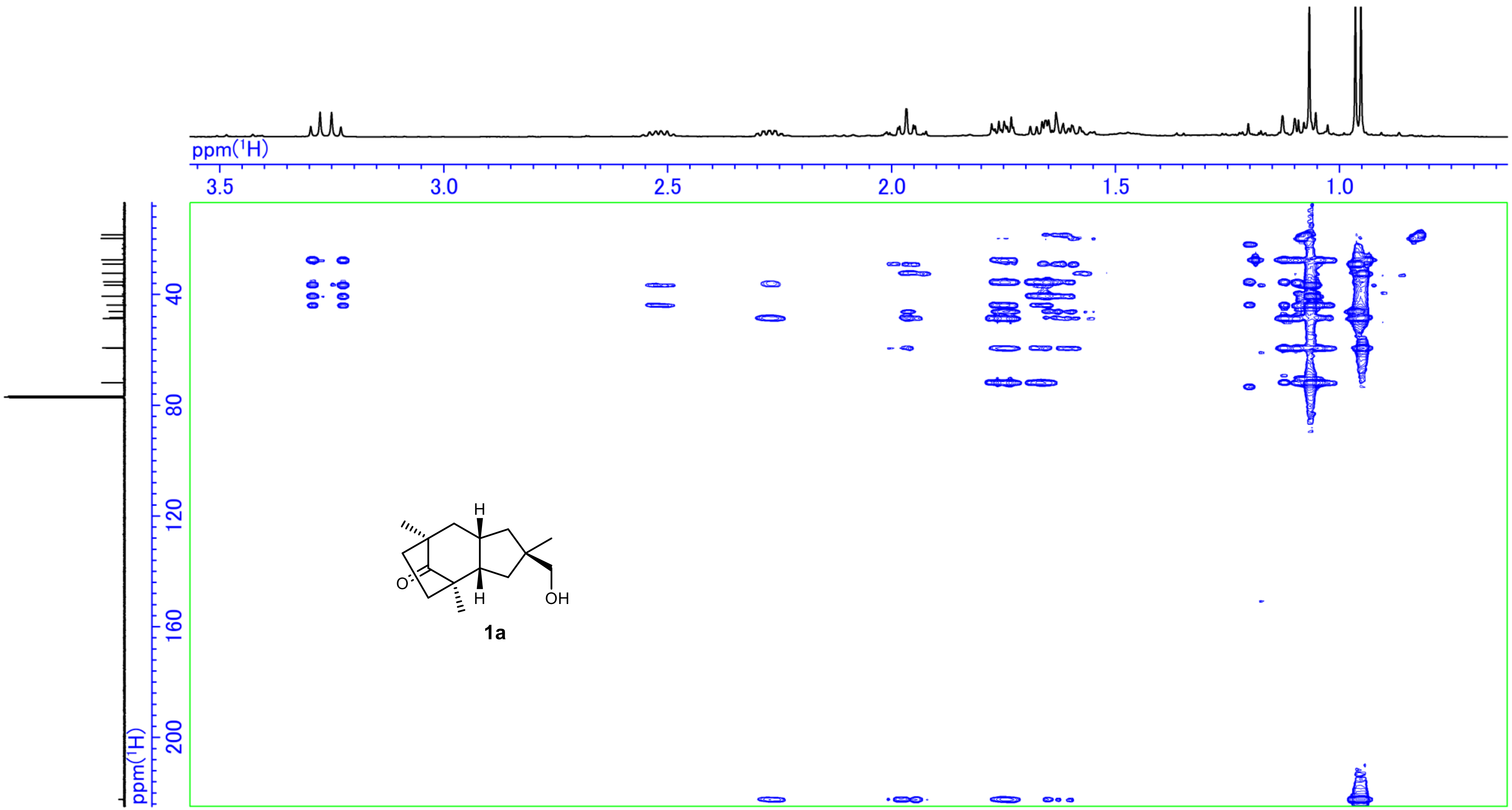

NOE 1D spectra of **1a** (500 MHz, $CDCl_3$)

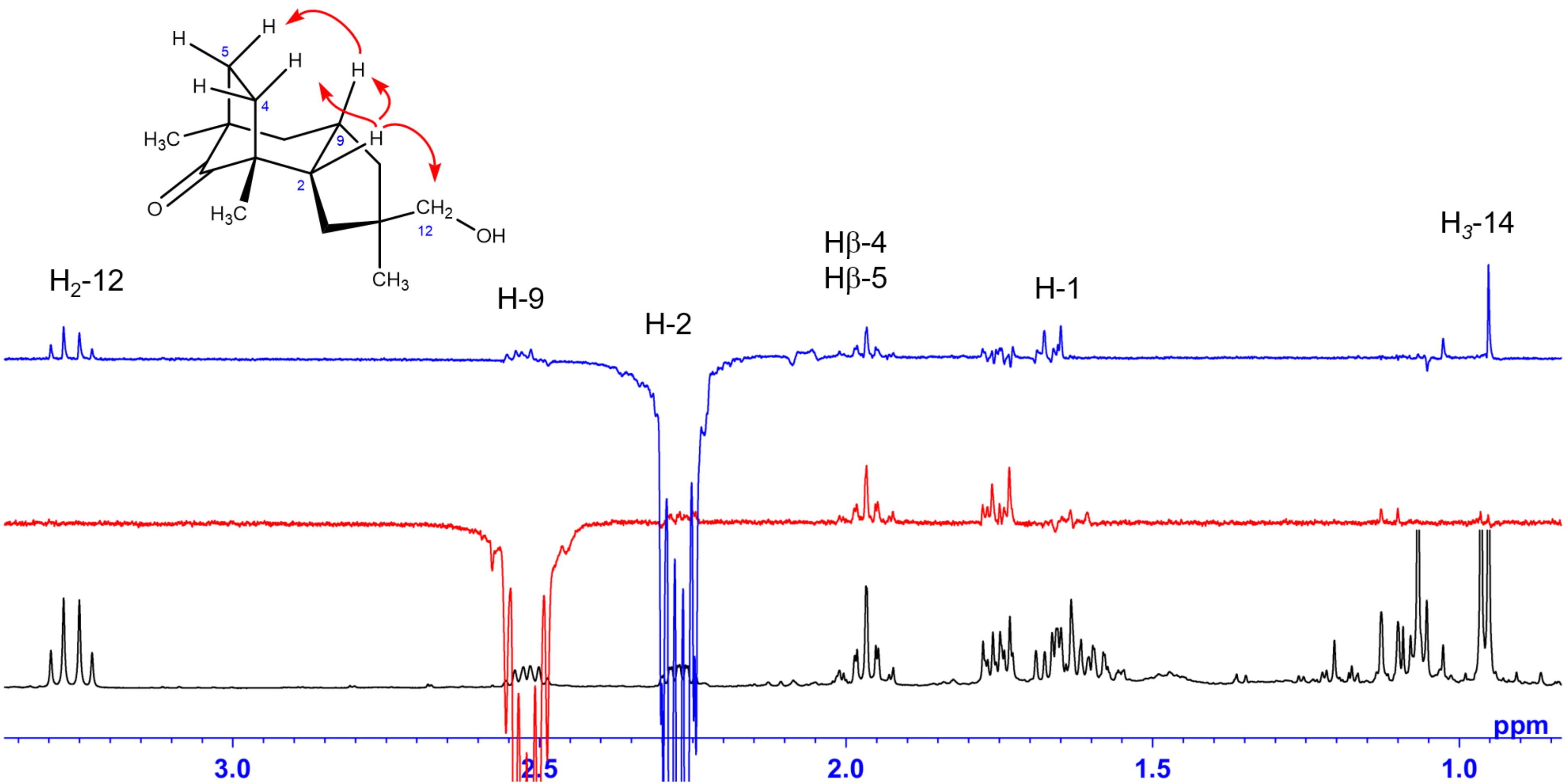

NOESY spectrum of **1a** (500 MHz, $CDCl_3$)

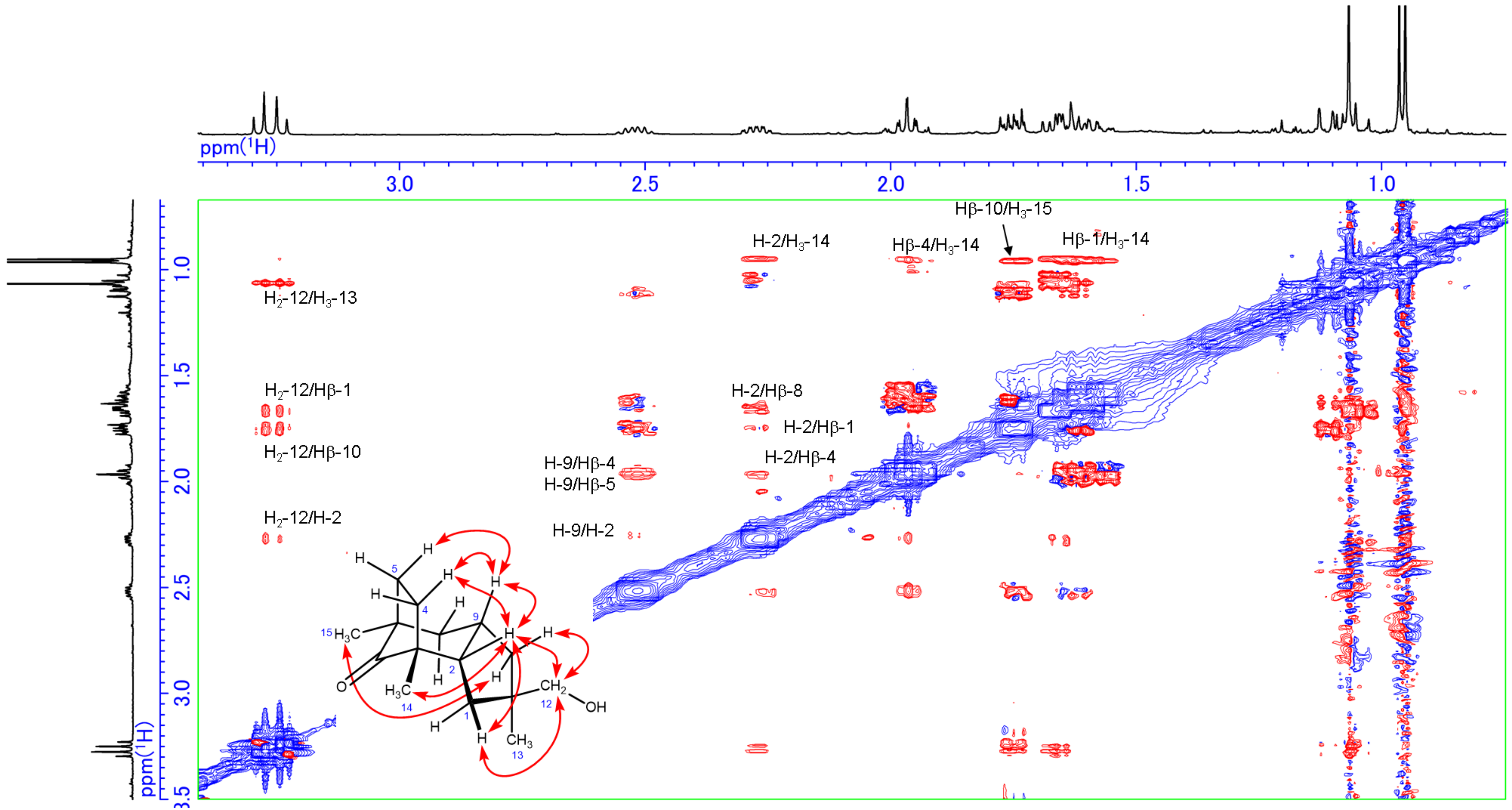

IR spectrum of **1b** (film)

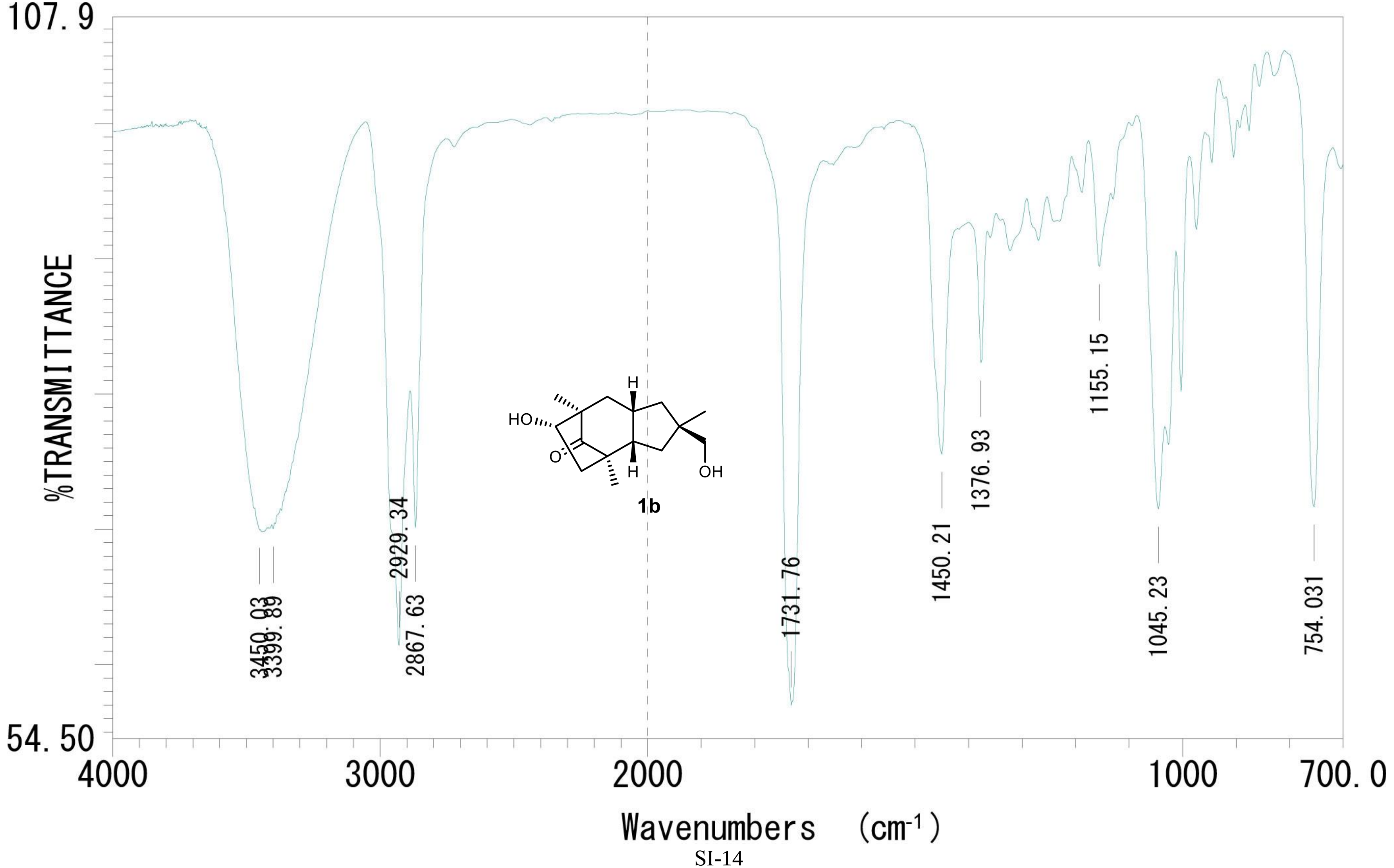

ESI-TOFMS spectrum of **1b**

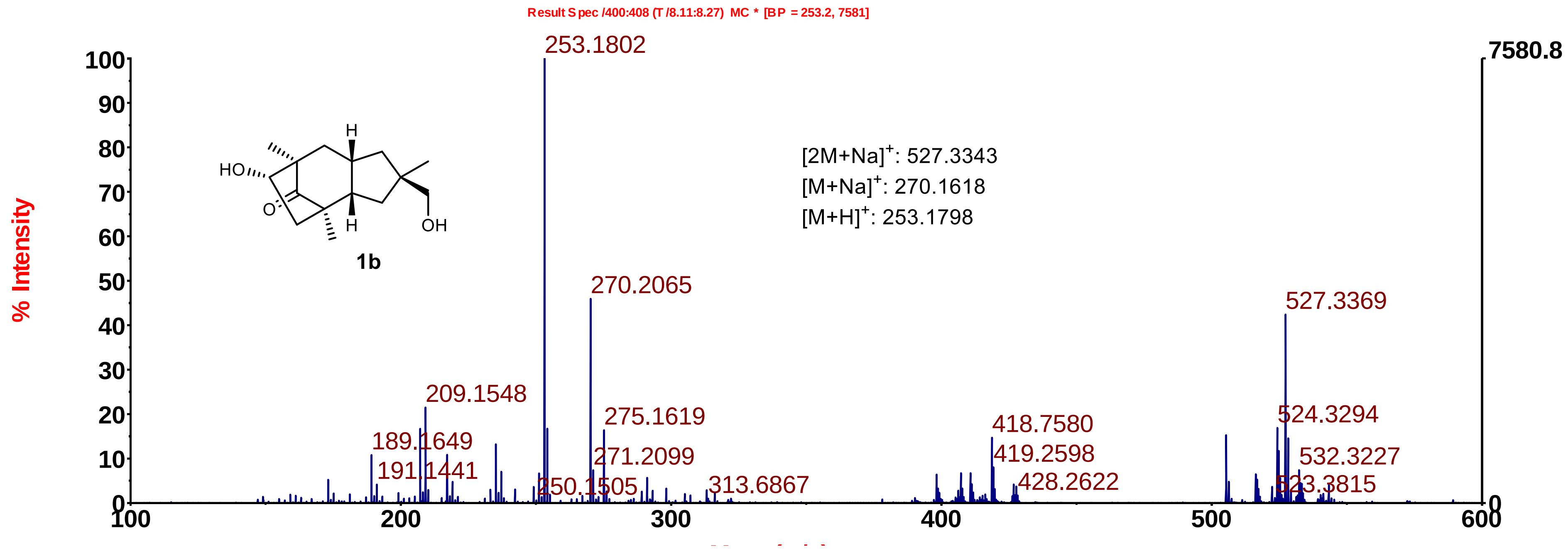

^{1}H NMR spectrum of **1b** (500 MHz, $CDCl_3$)

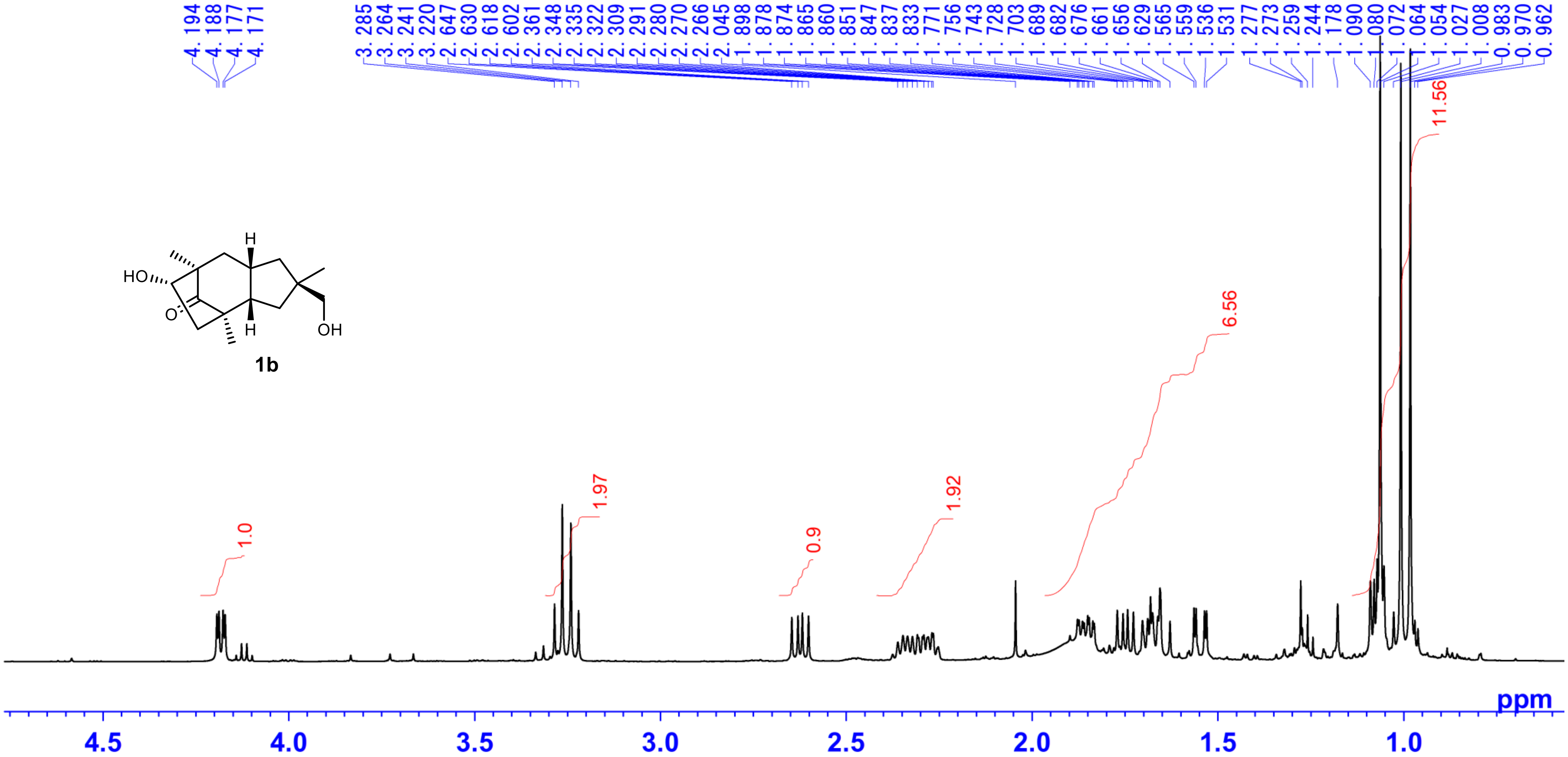

^{13}C NMR spectrum of **1b** (125 MHz, $CDCl_3$)

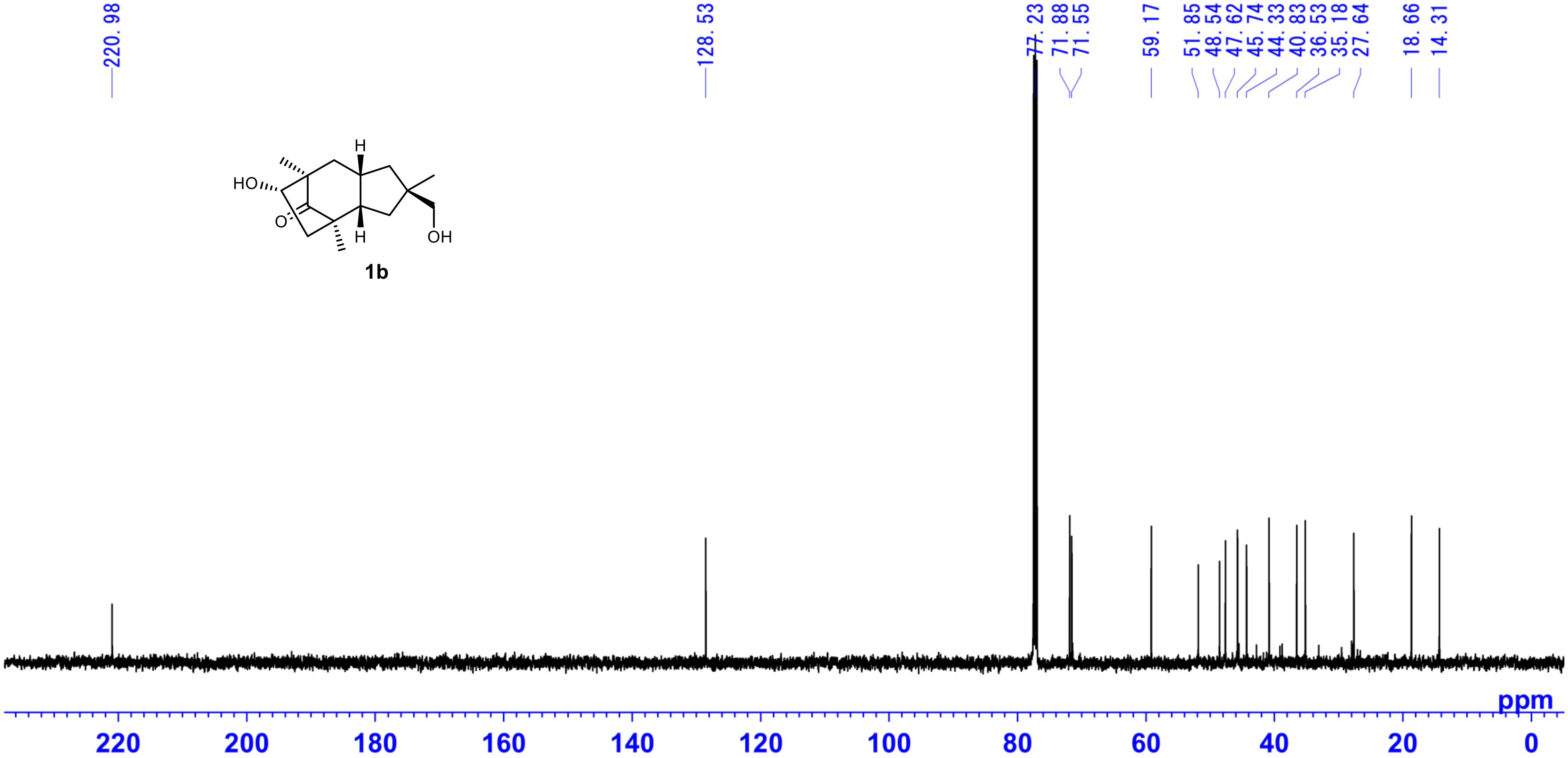

DQF COSY spectrum of **1b** (500 MHz, $CDCl_3$)

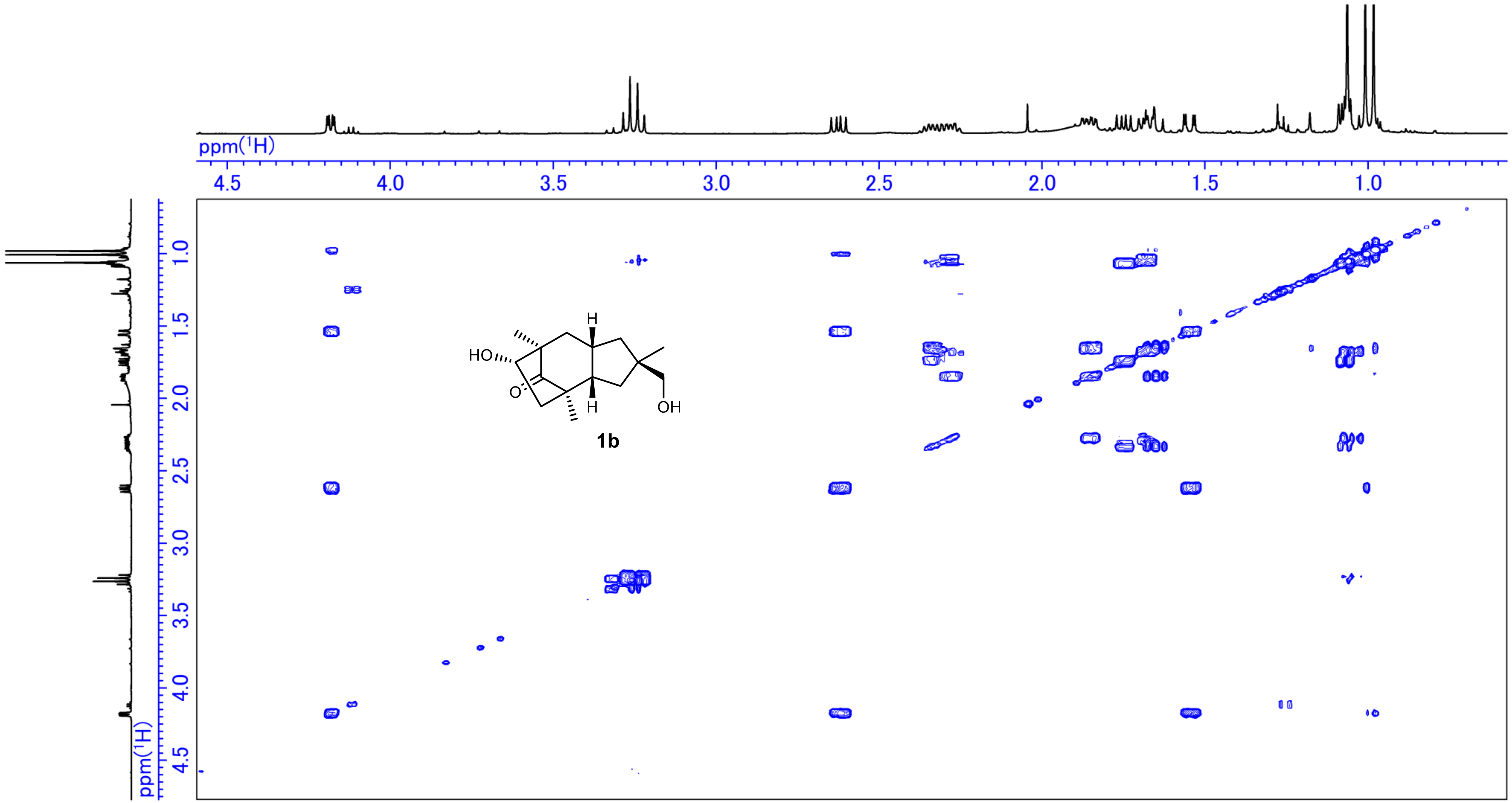

HSQC spectrum of **1b** (500 MHz, $CDCl_3$)

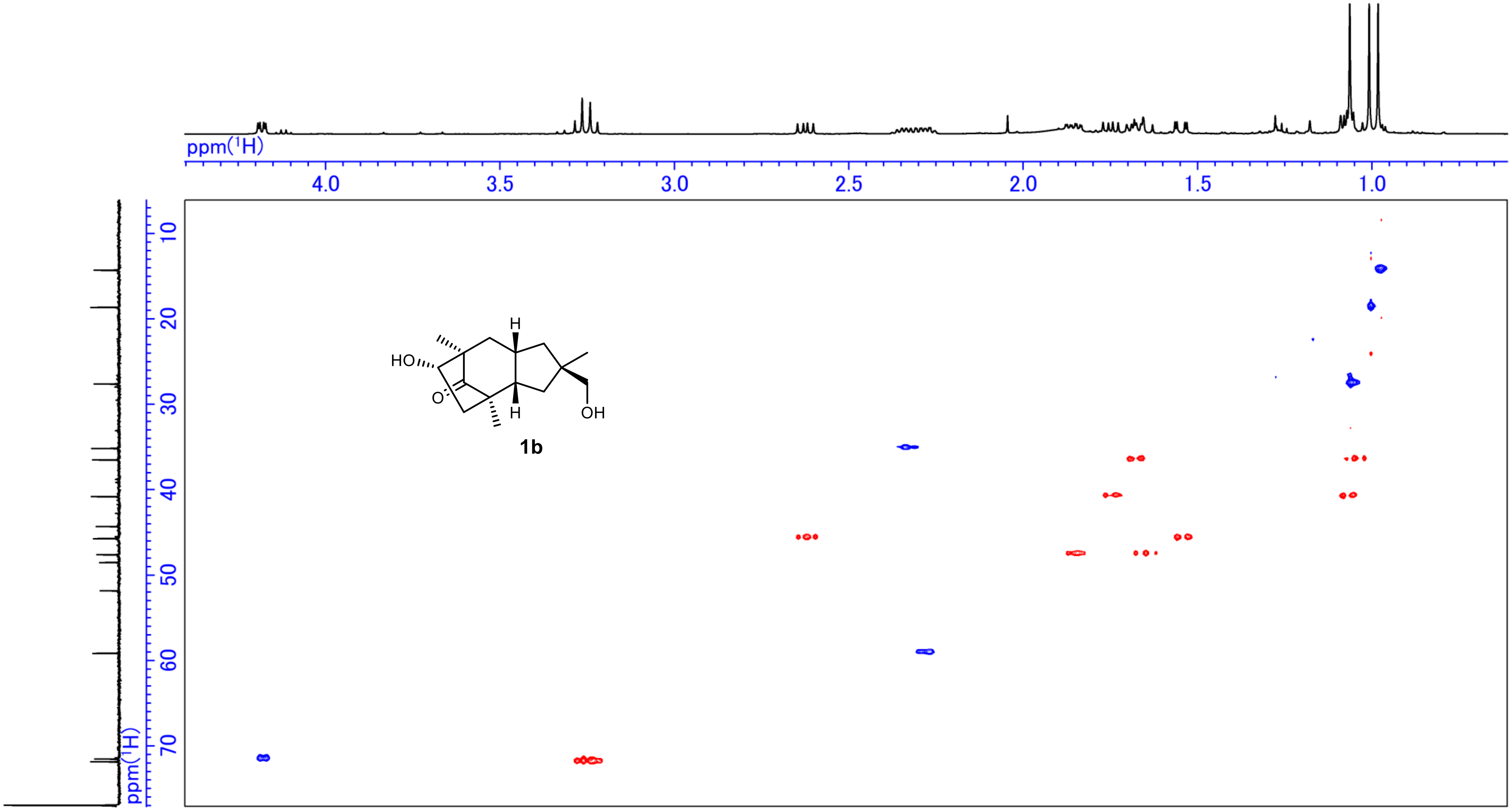

HMBC spectrum of **1b** (500 MHz, $CDCl_3$)

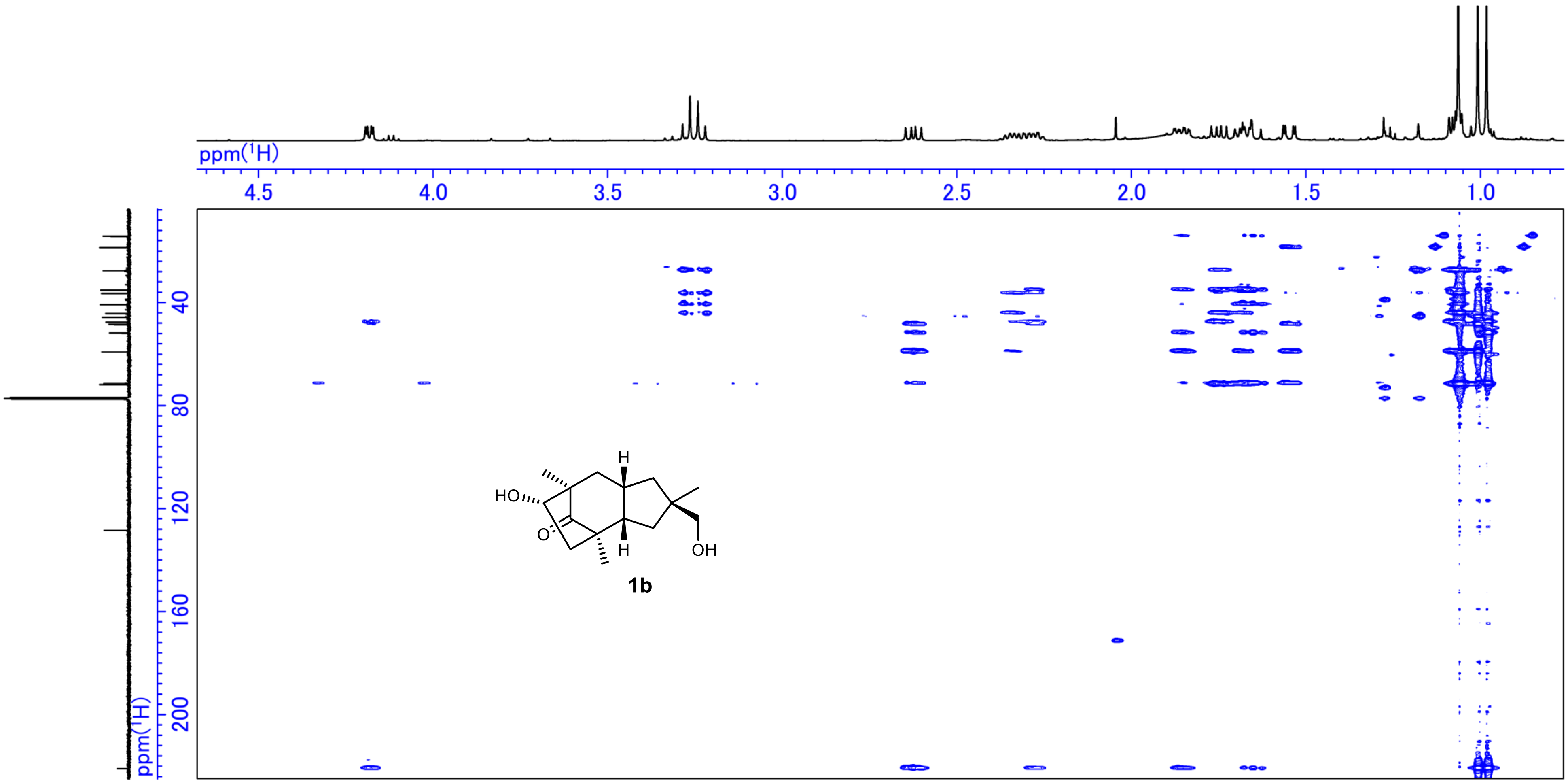

NOESY spectrum of **1b** (500 MHz, $CDCl_3$)

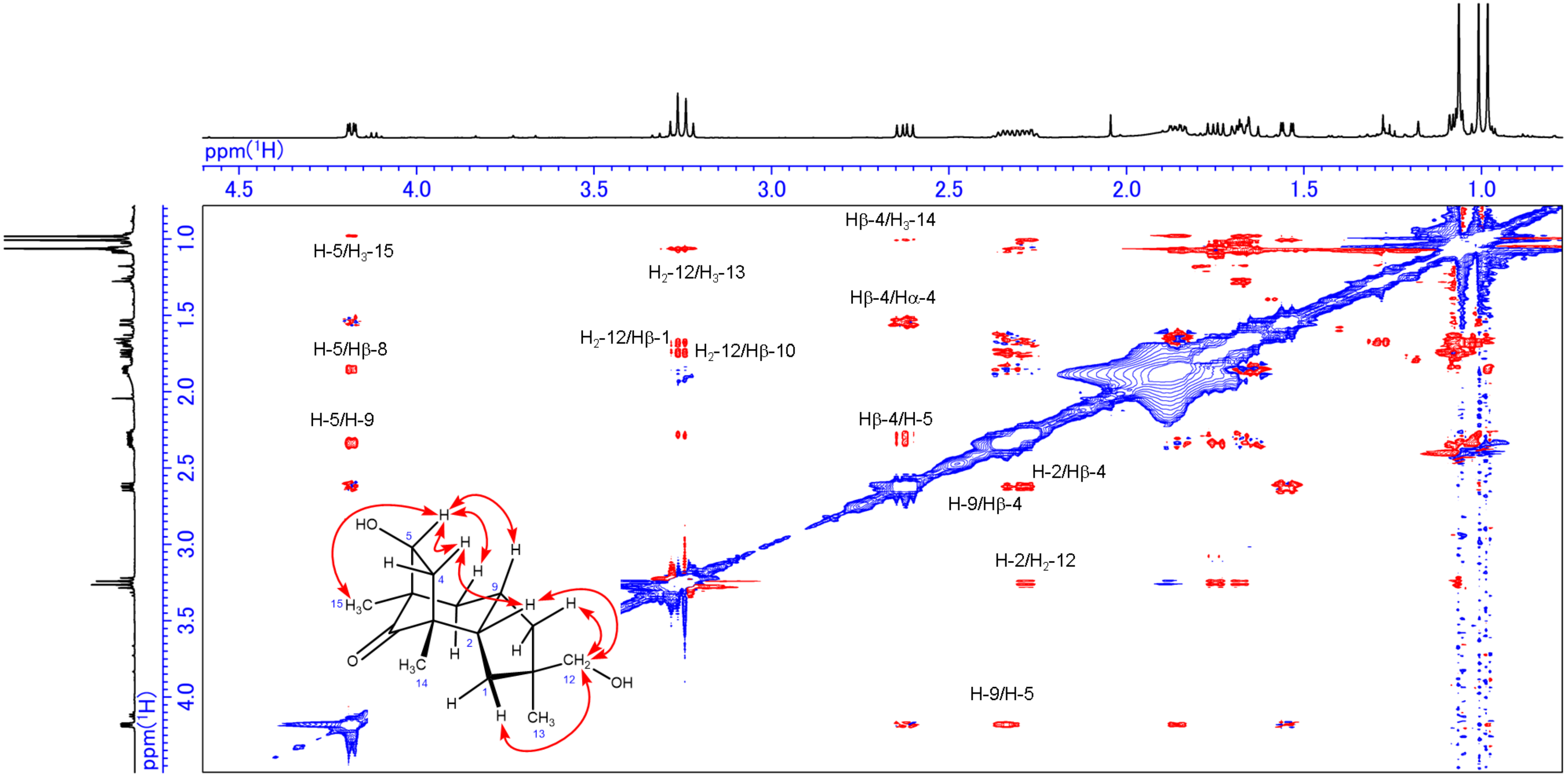

IR spectrum of **1c** (film).

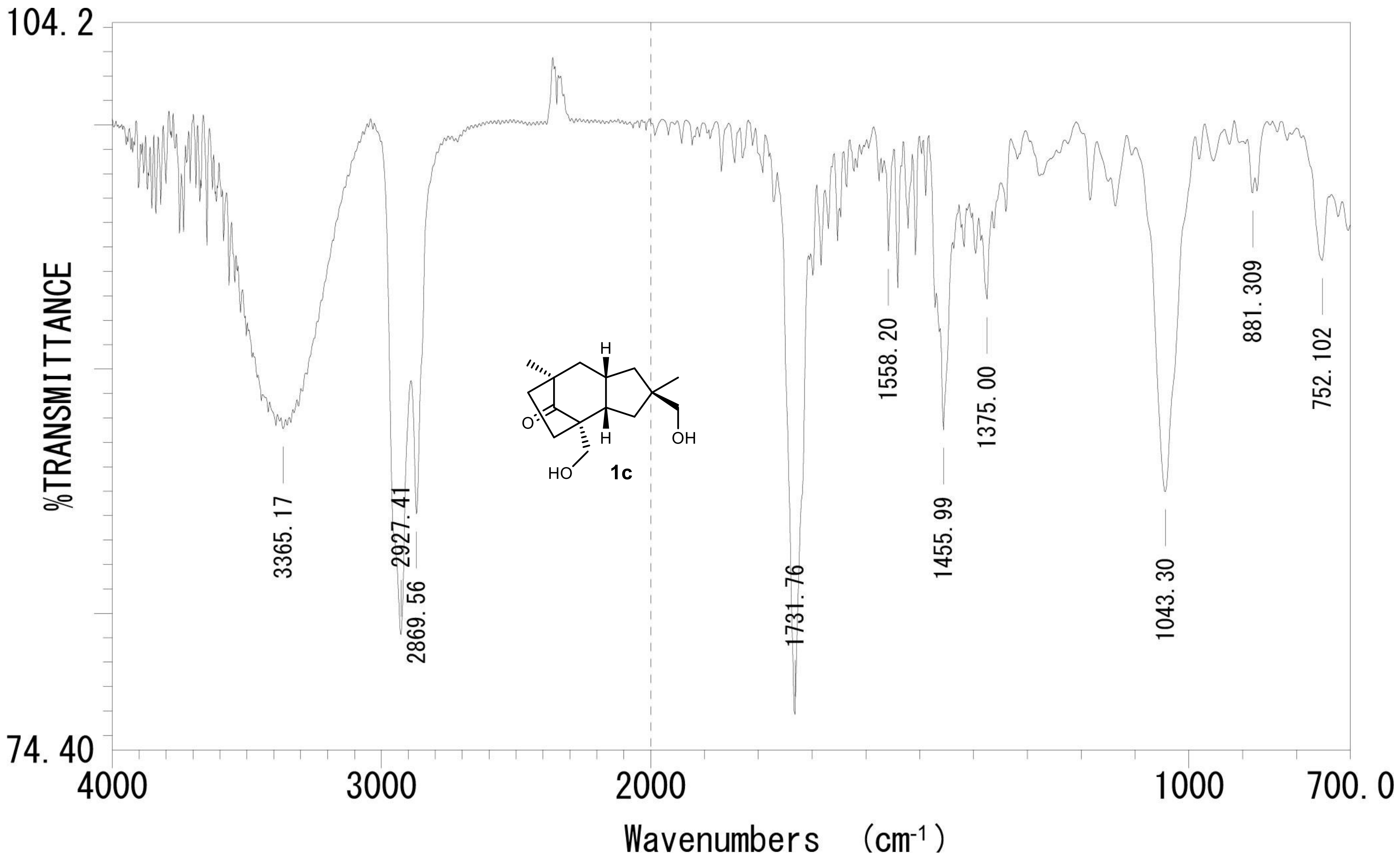

ESI-TOFMS spectrum of **1c**

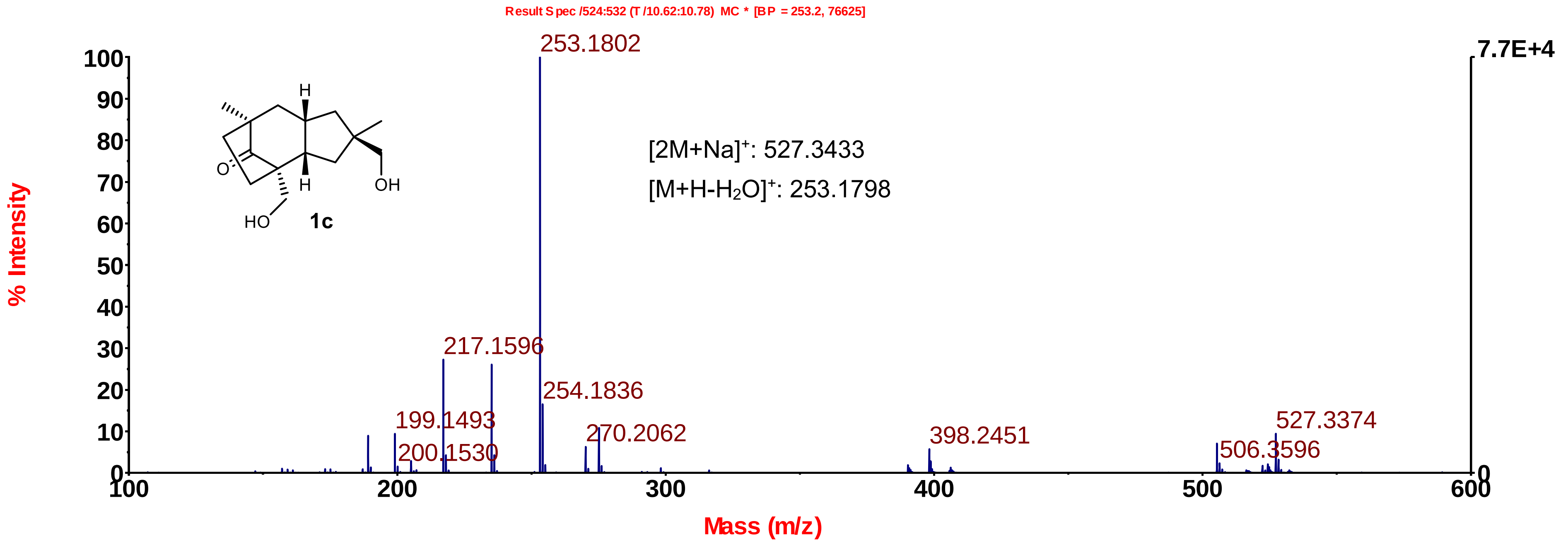

^{1}H NMR spectrum of **1c** (500 MHz, acetone-d_6)

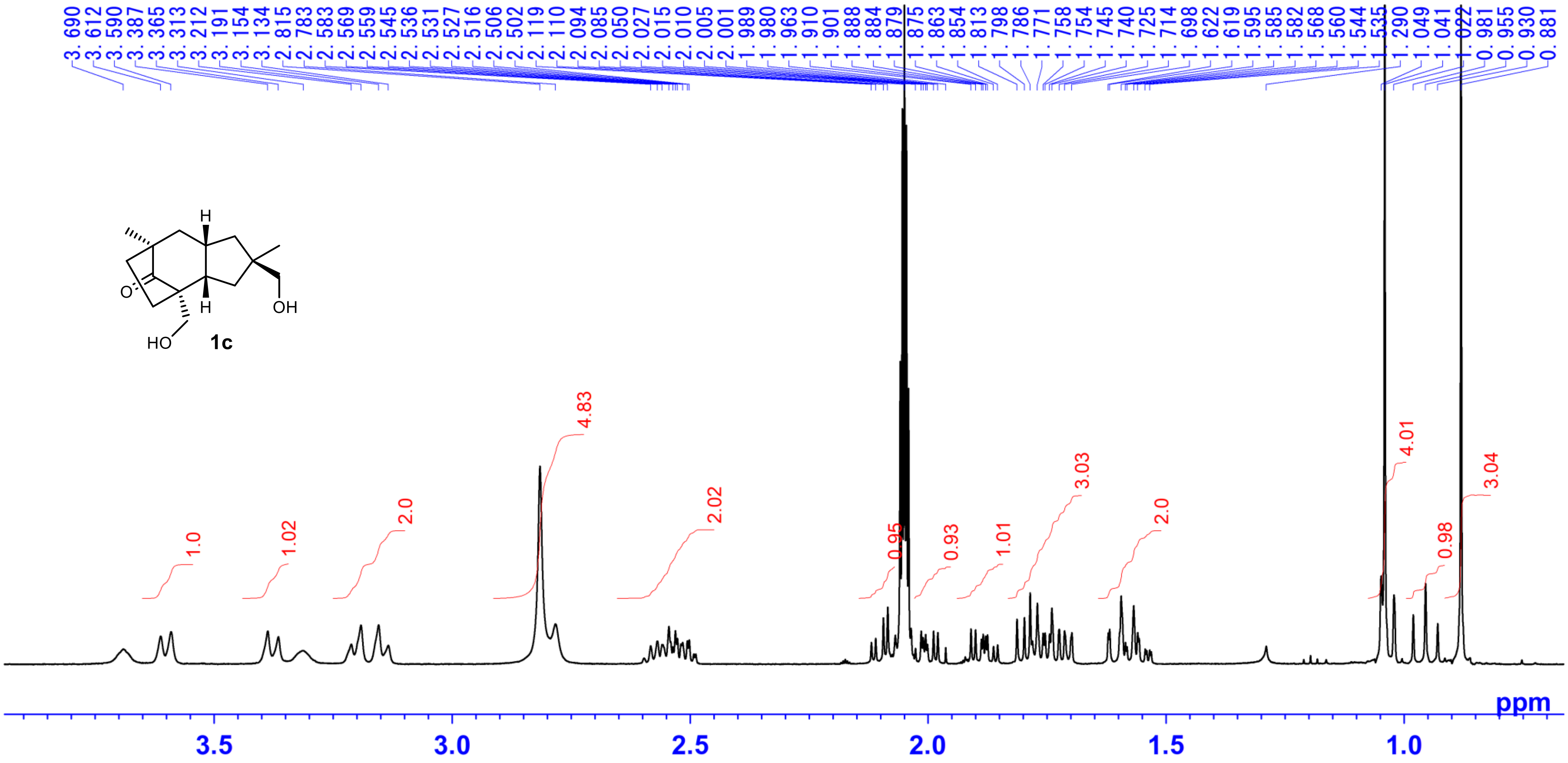

^{13}C NMR spectrum of **1c** (125 MHz, acetone-d_6)

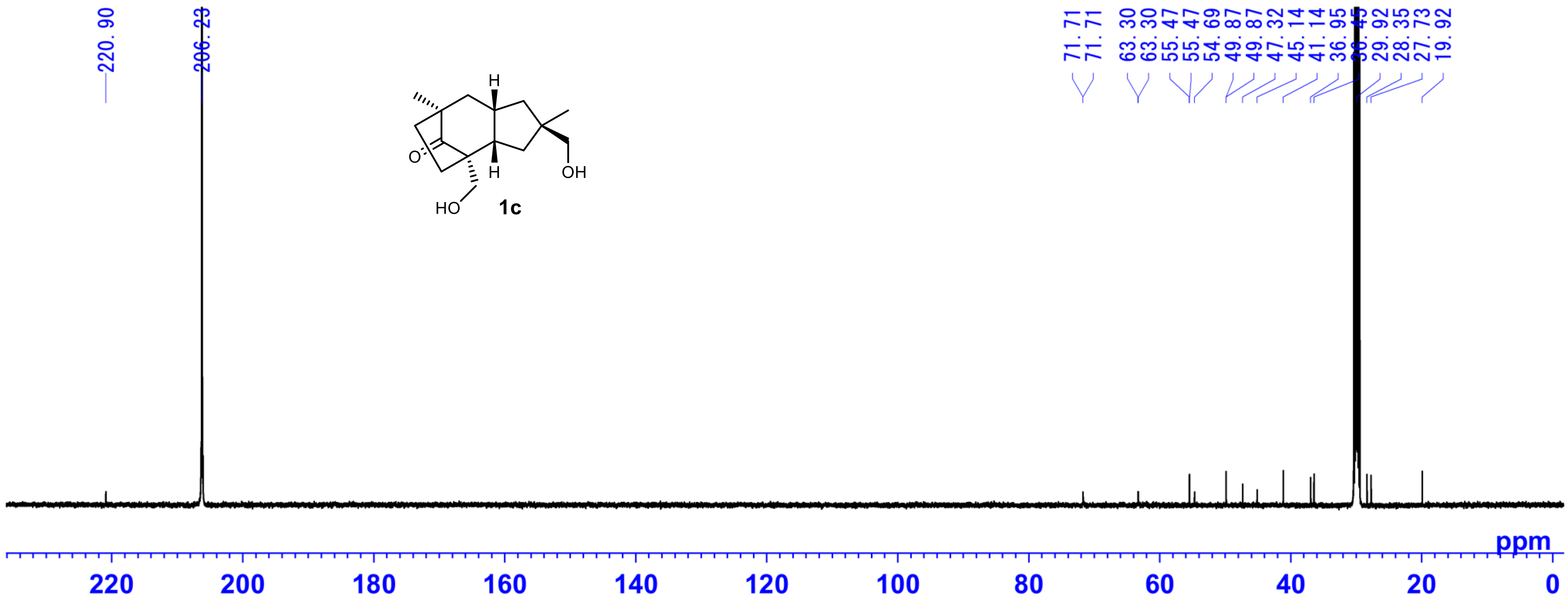

DQF COSY spectrum of **1c** (500 MHz, acetone-d_6)

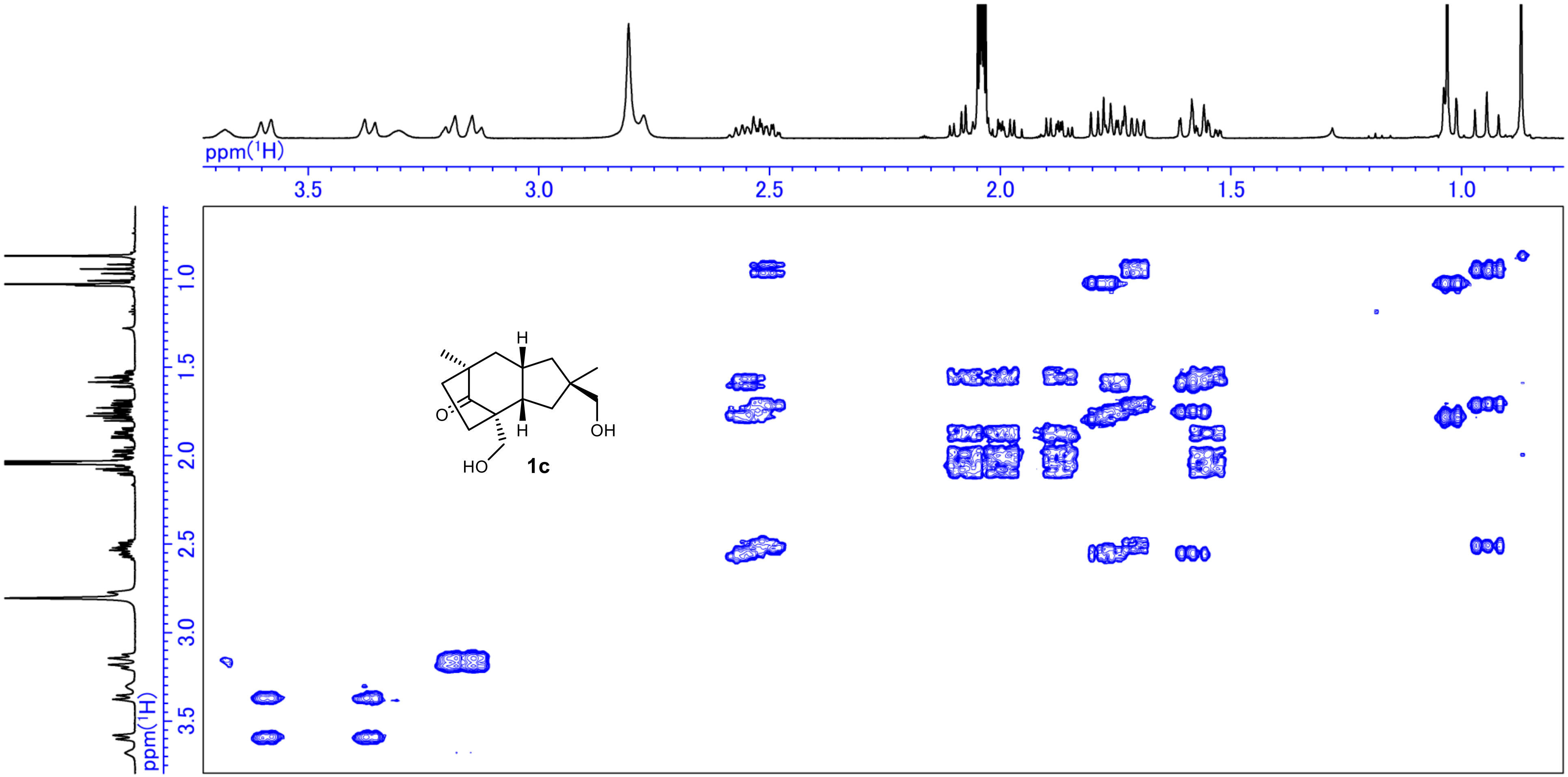

HSQC spectrum of **1c** (500 MHz, acetone-d_6)

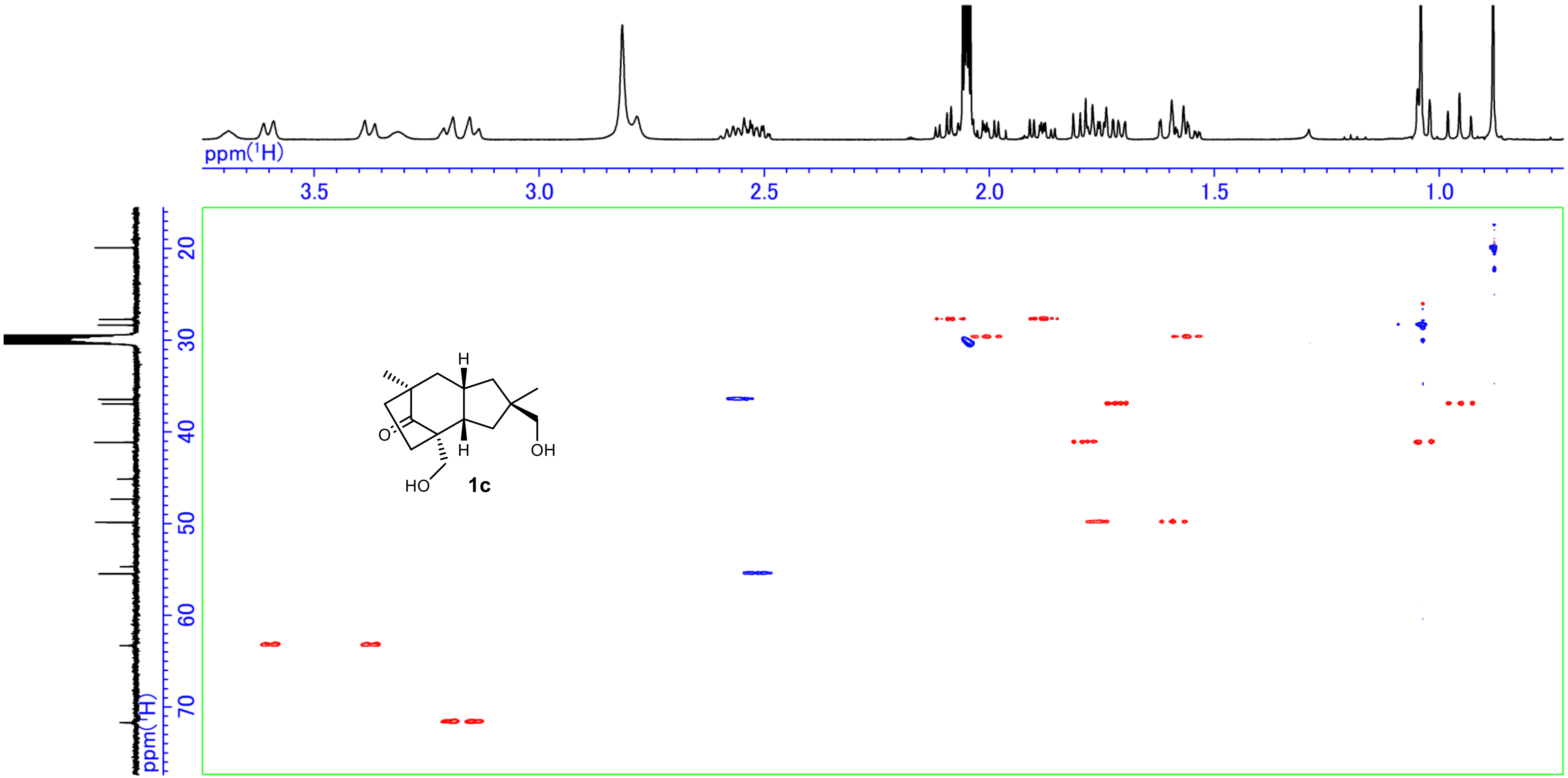

HMBC spectrum of **1c** (500 MHz, acetone-d_6)

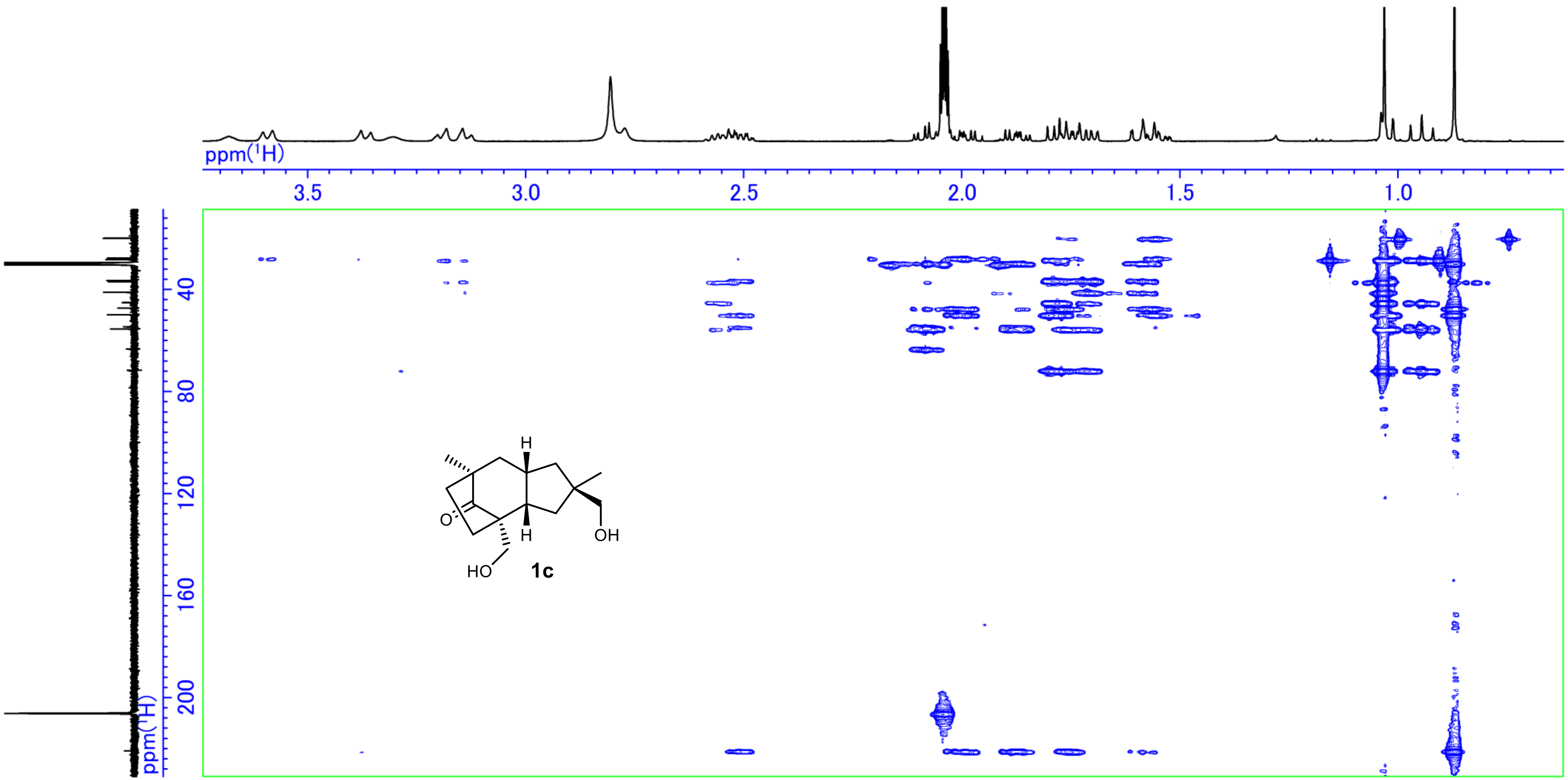

NOESY spectrum of **1c** (500 MHz, acetone-d_6)

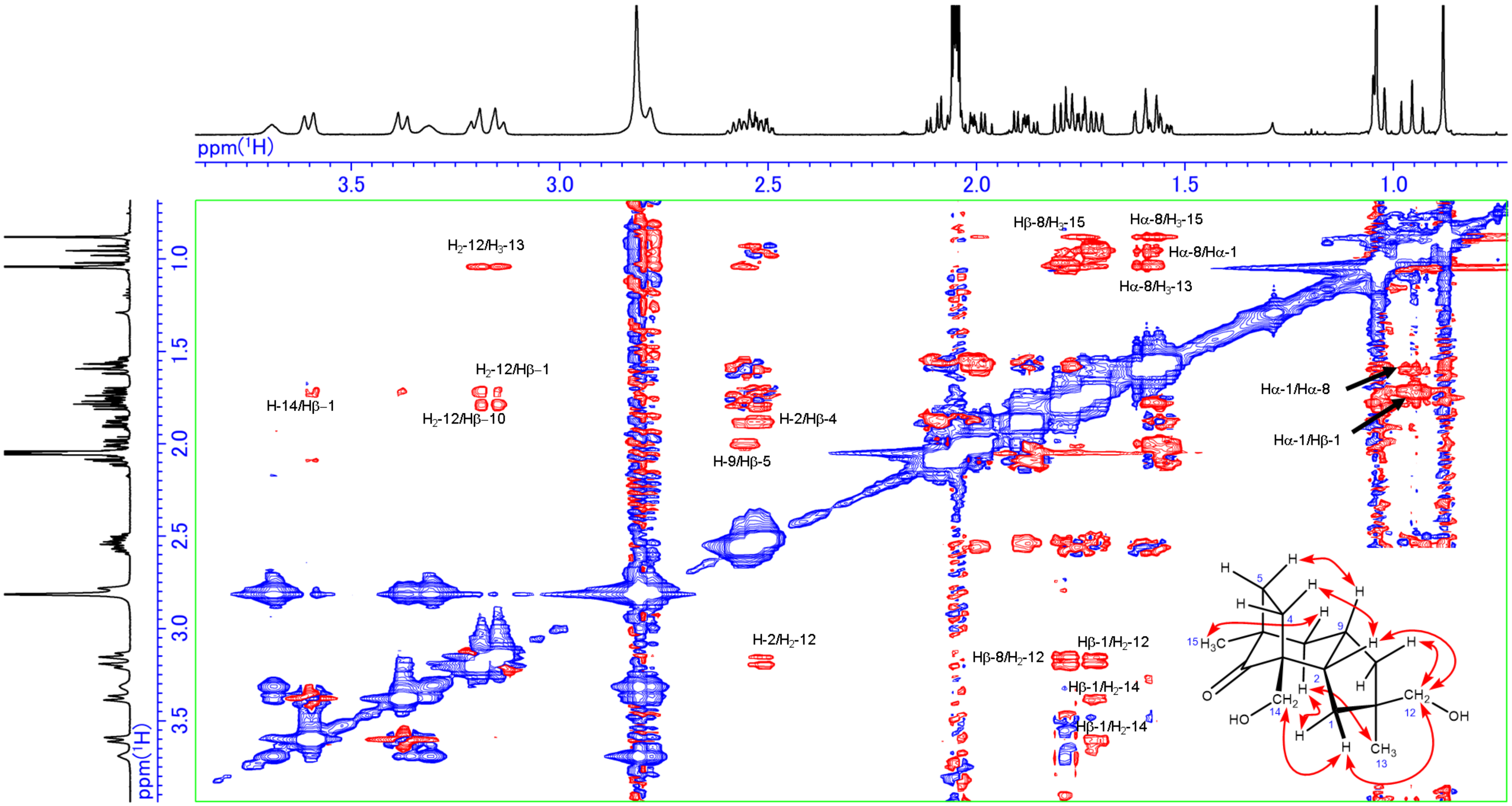

IR spectrum (film) of **2a**

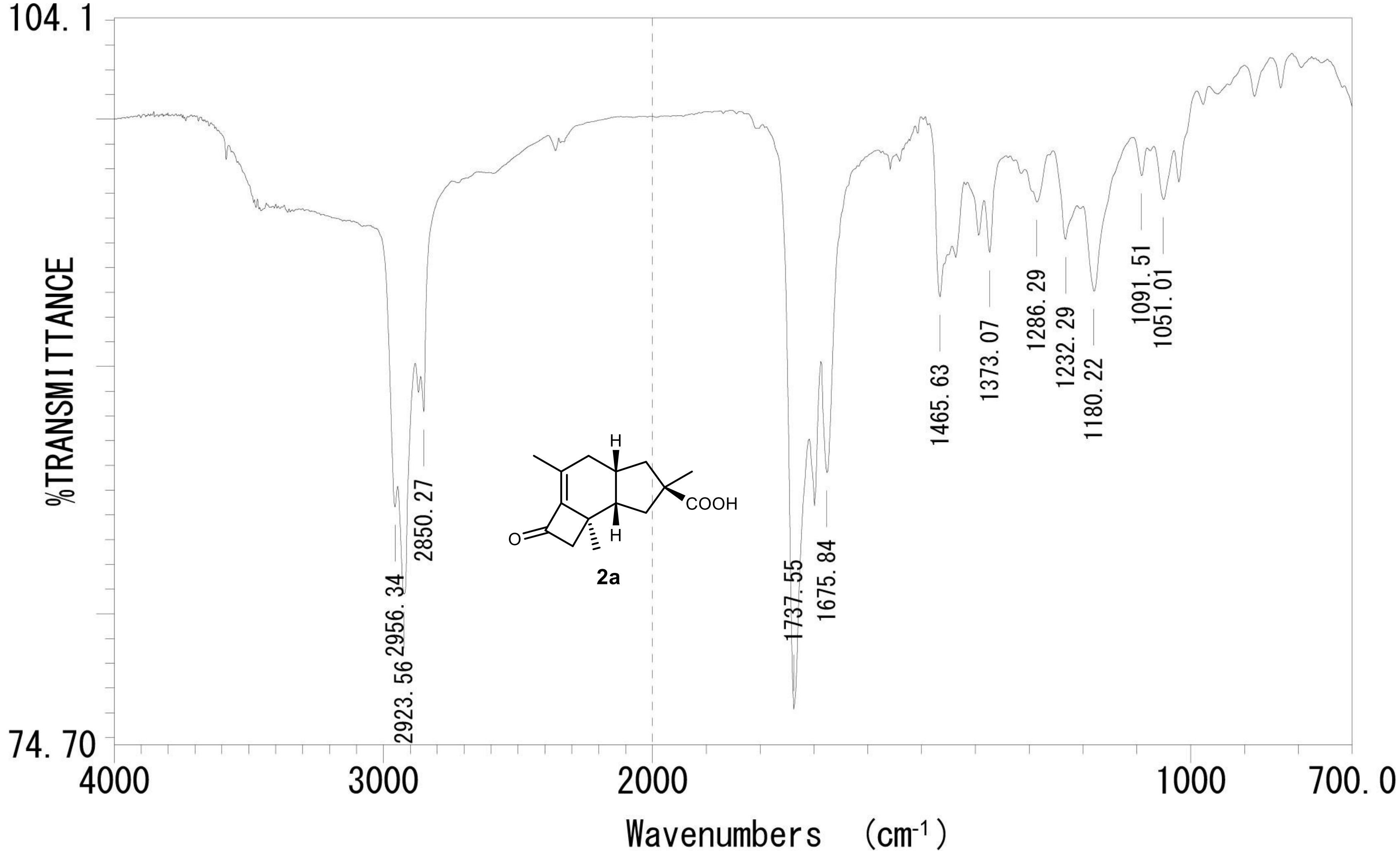

ESI-TOFMS spectrum of **2a**,

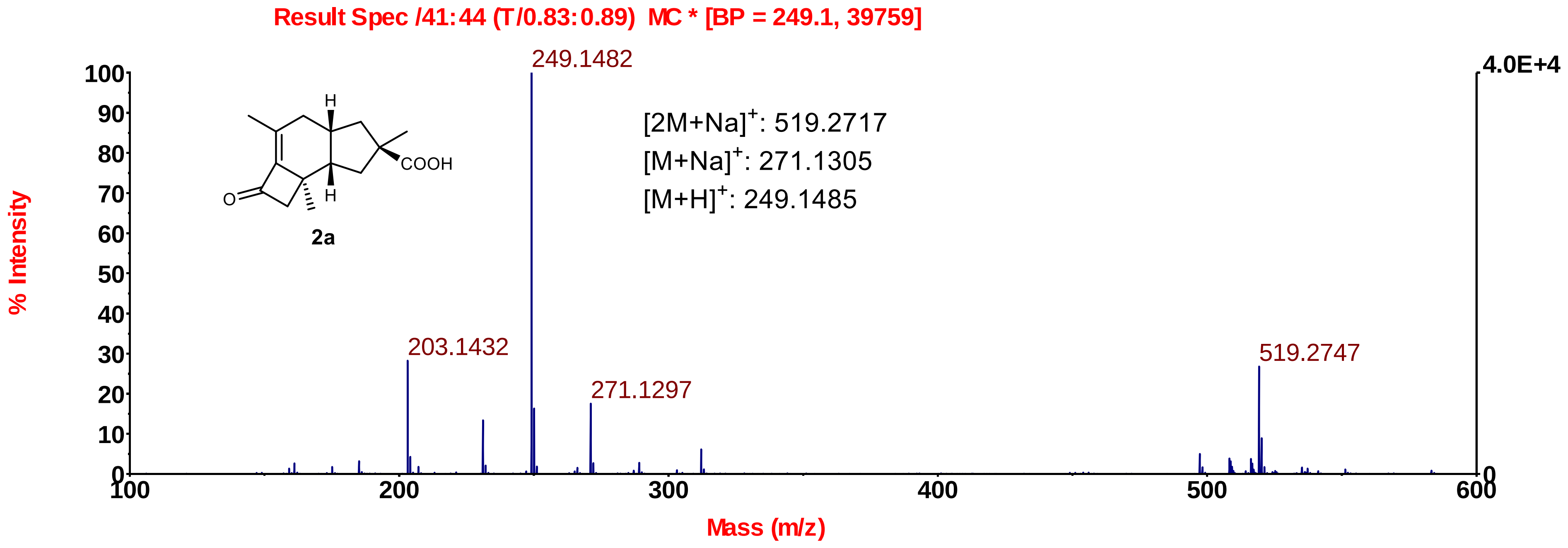

^{1}H NMR spectrum of **2a** (500 MHz, $CDCl_3$)

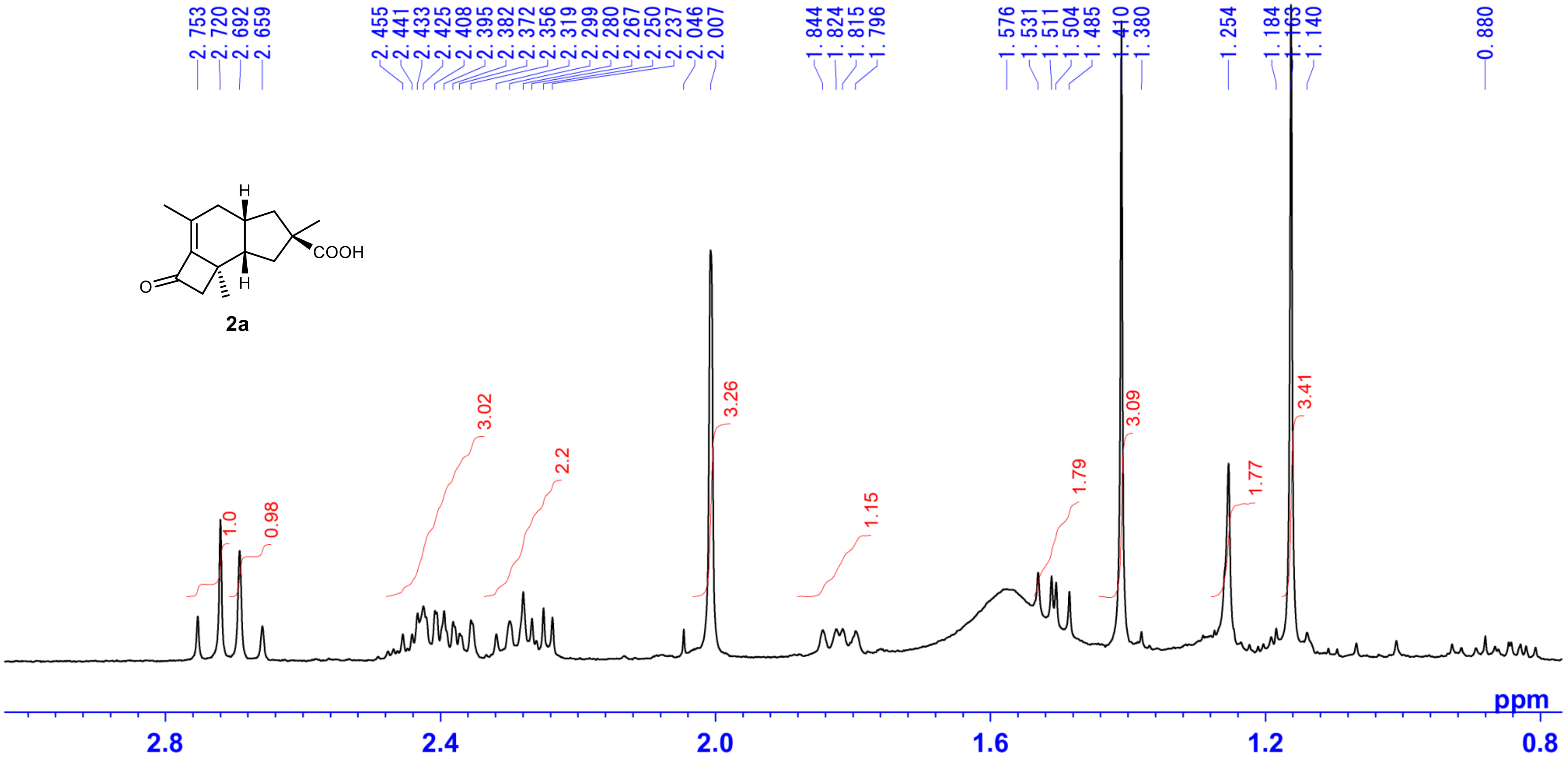

^{13}C NMR spectrum of **2a** (125 MHz, $CDCl_3$)

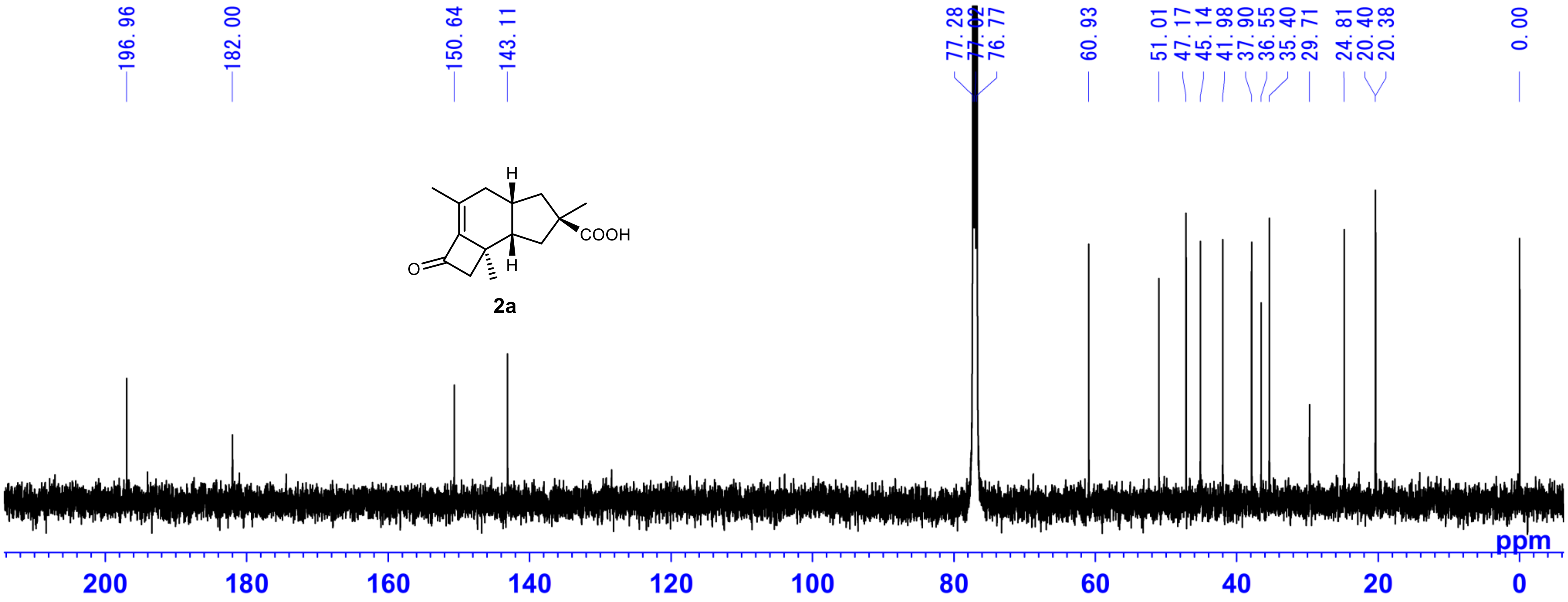

DQF COSY spectrum of **2a** (500 MHz, $CDCl_3$)

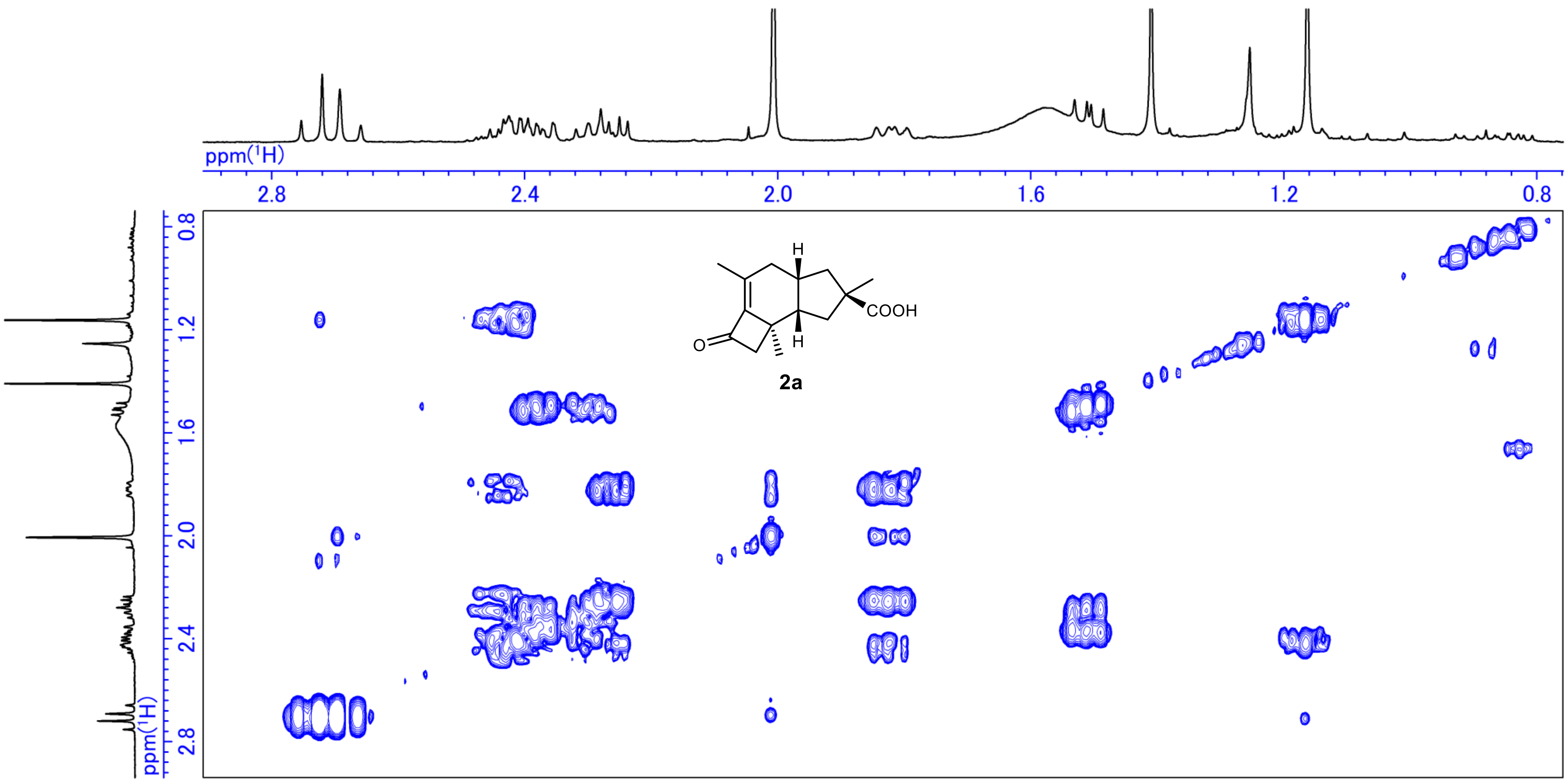

HSQC spectrum of **2a** (500 MHz, $CDCl_3$)

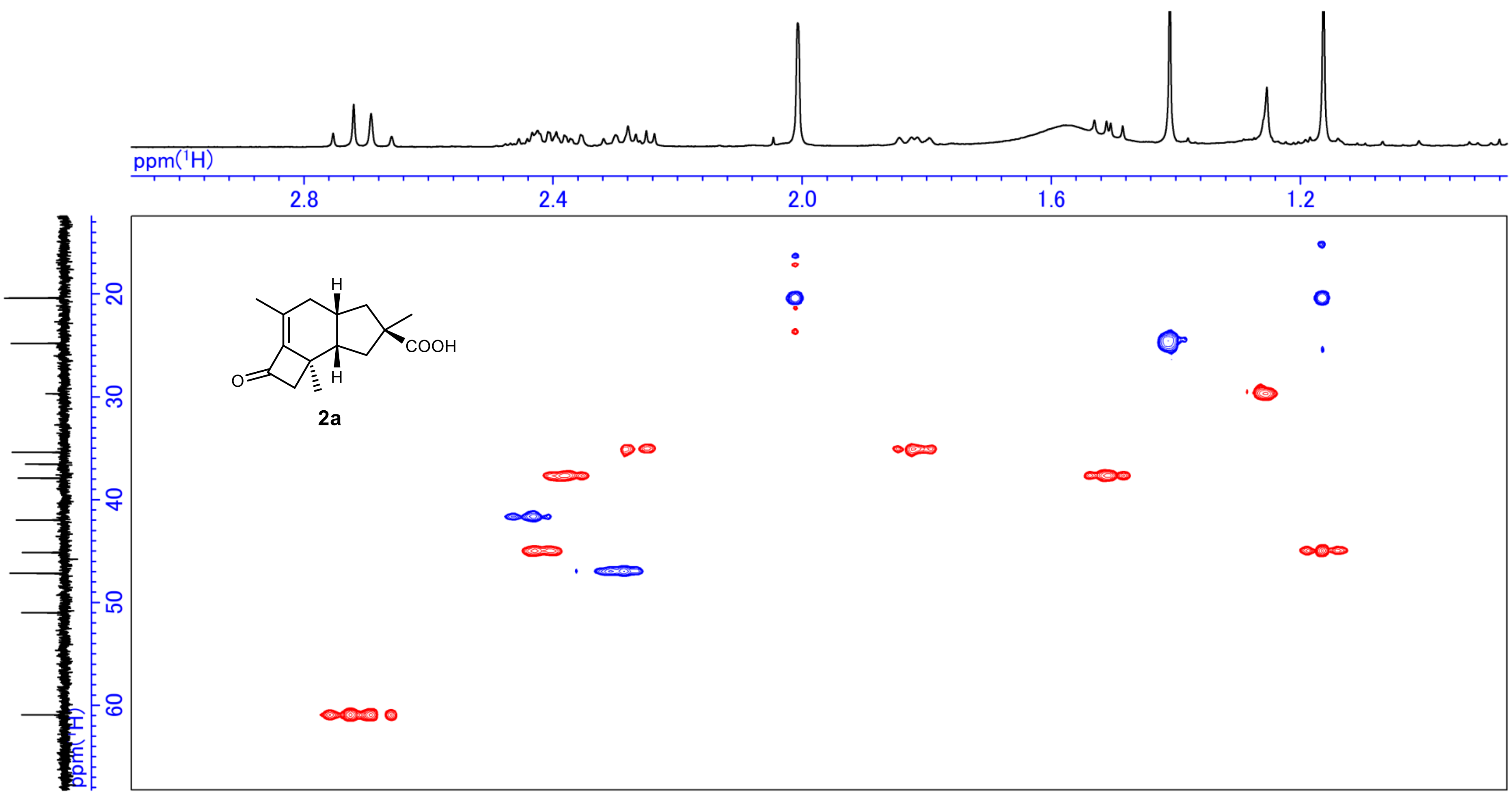

HMBC spectrum of **2a** (500 MHz, $CDCl_3$)

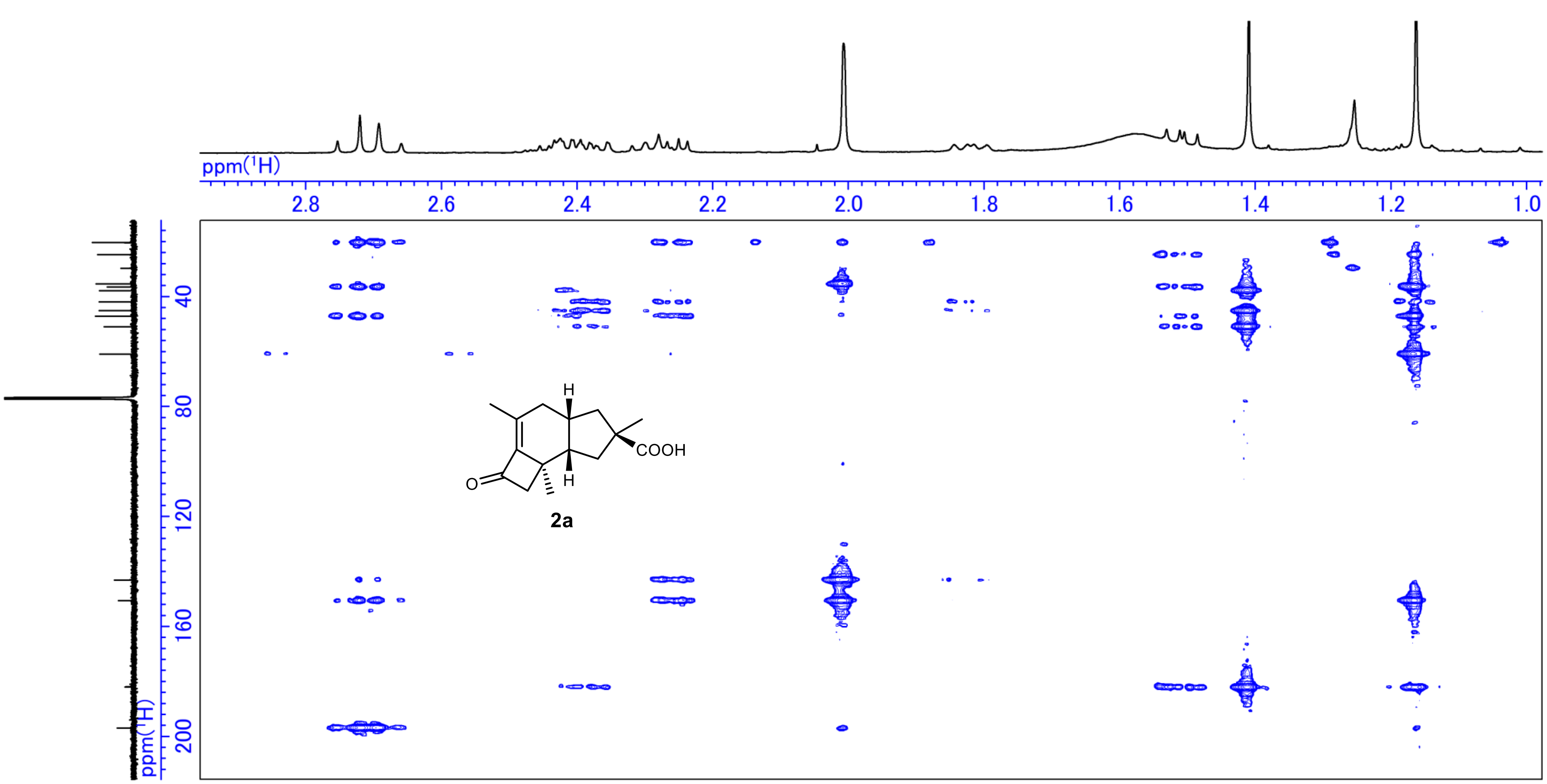

IR spectrum of **2b** (film)

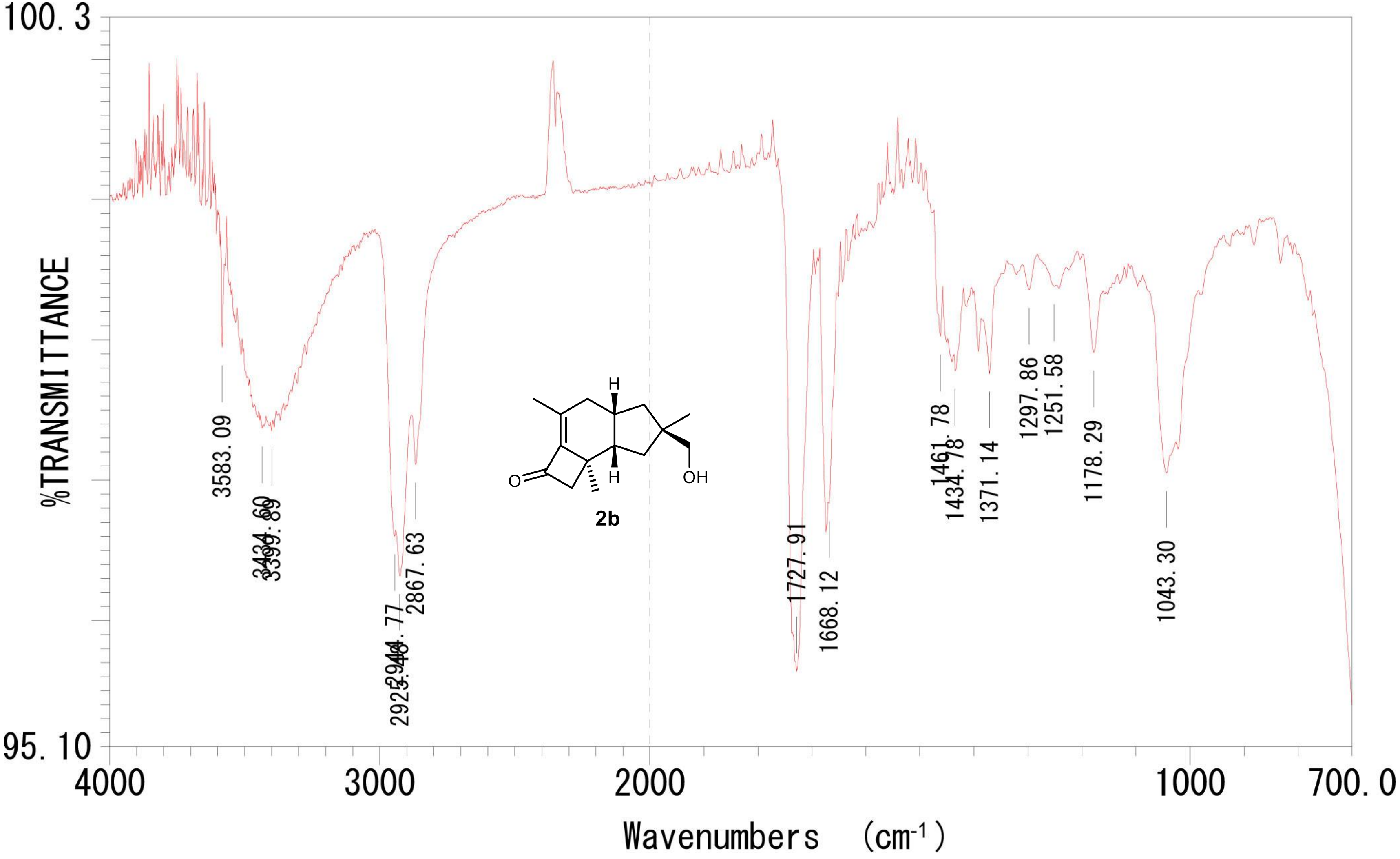

ESI-TOFMS spectrum of **2b**

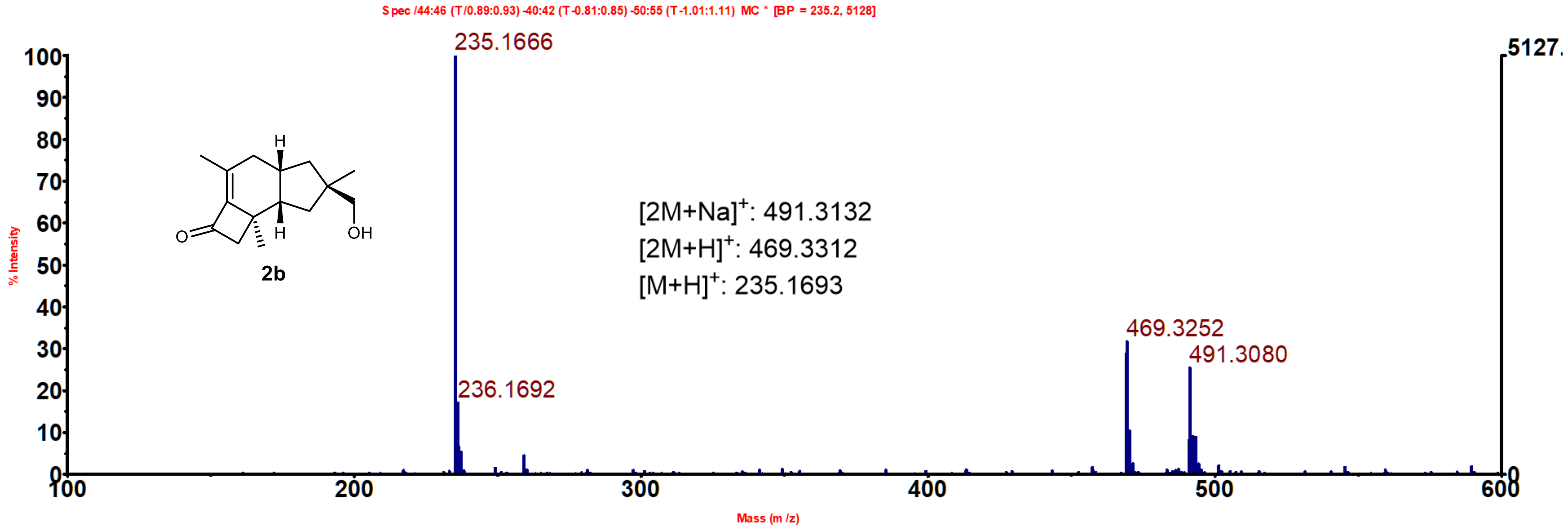

^{1}H NMR spectrum of **2b** (500 MHz, $CDCl_3$)

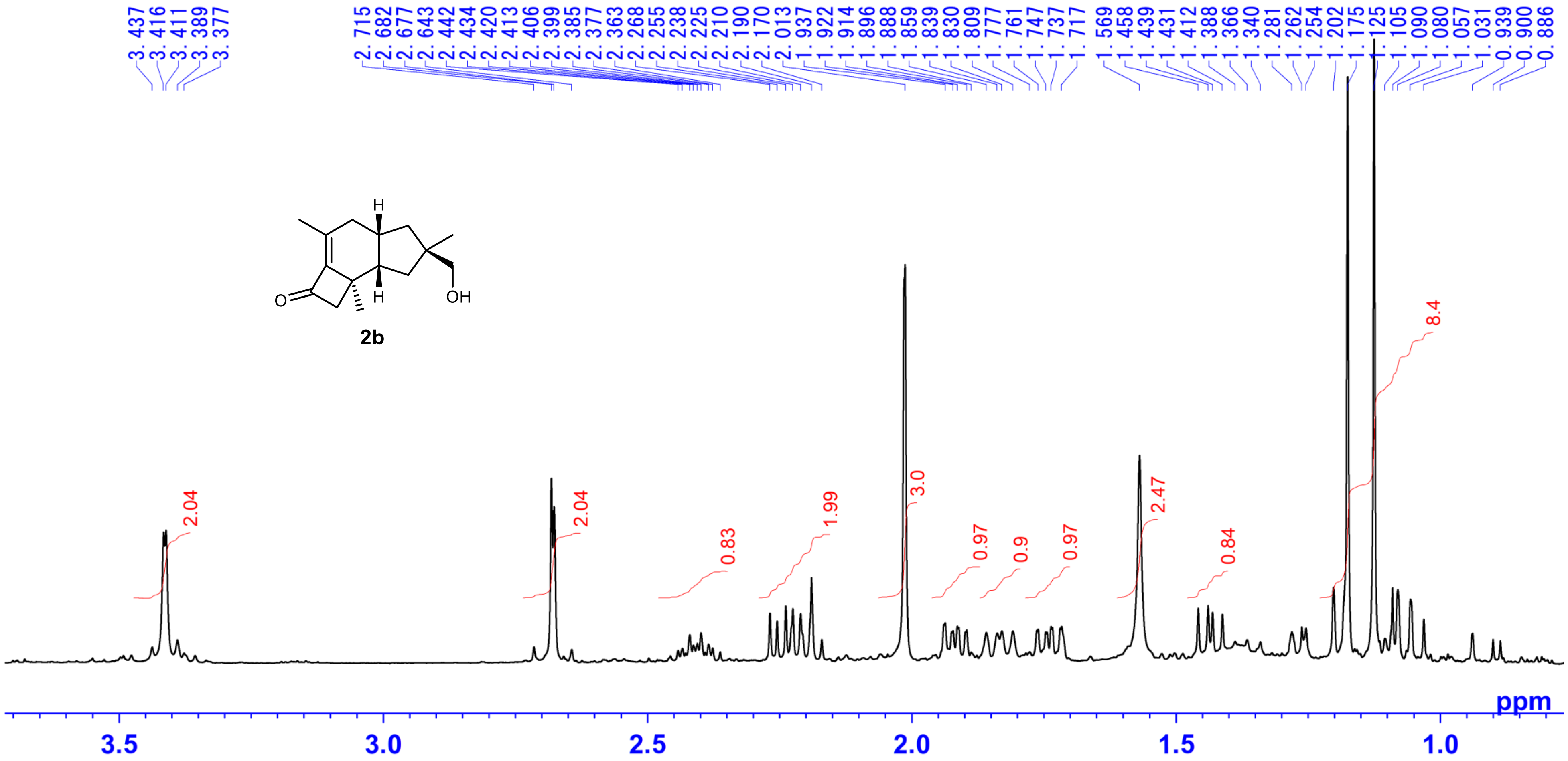

^{13}C NMR spectrum of **2b** (125 MHz, $CDCl_3$)

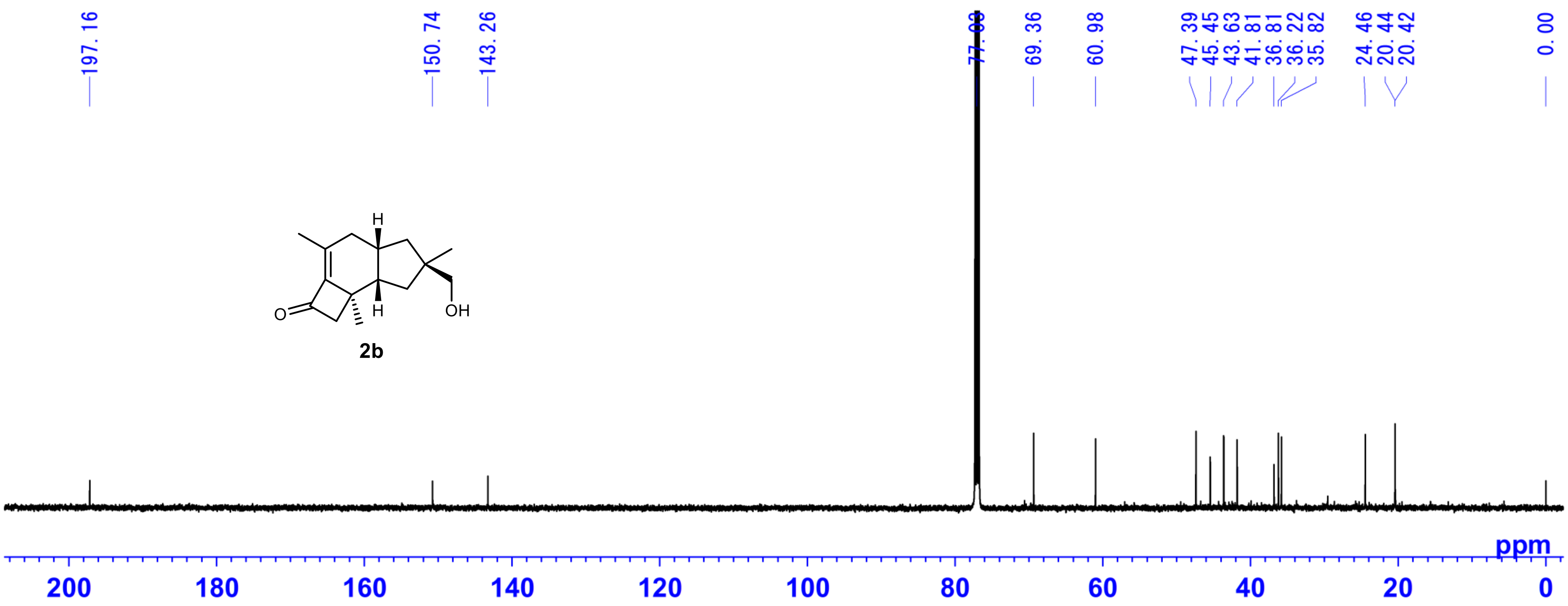

NOE 1D spectra of **2b** (500 MHz, $CDCl_3$)

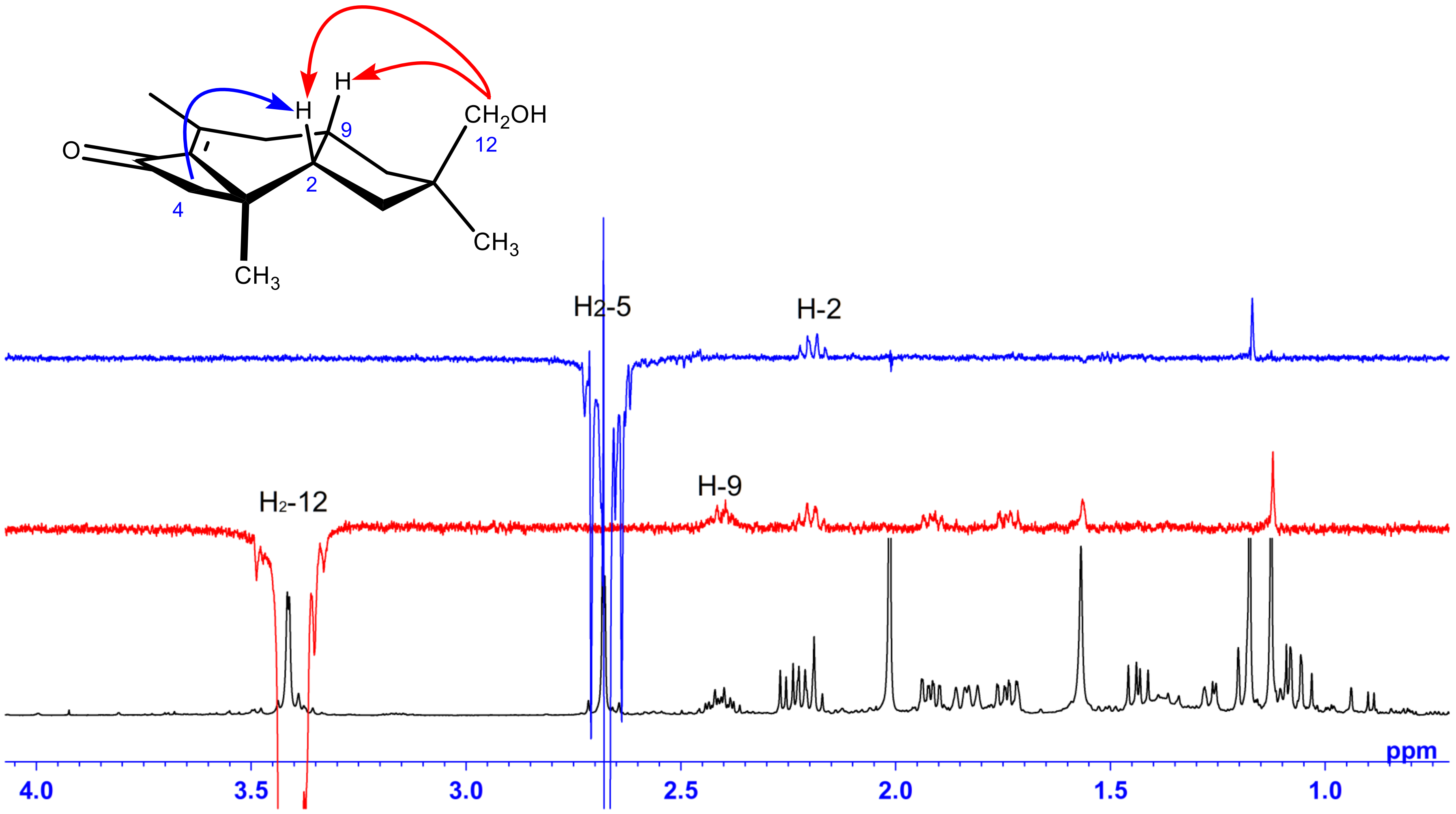

IR spectrum of **3** (film)

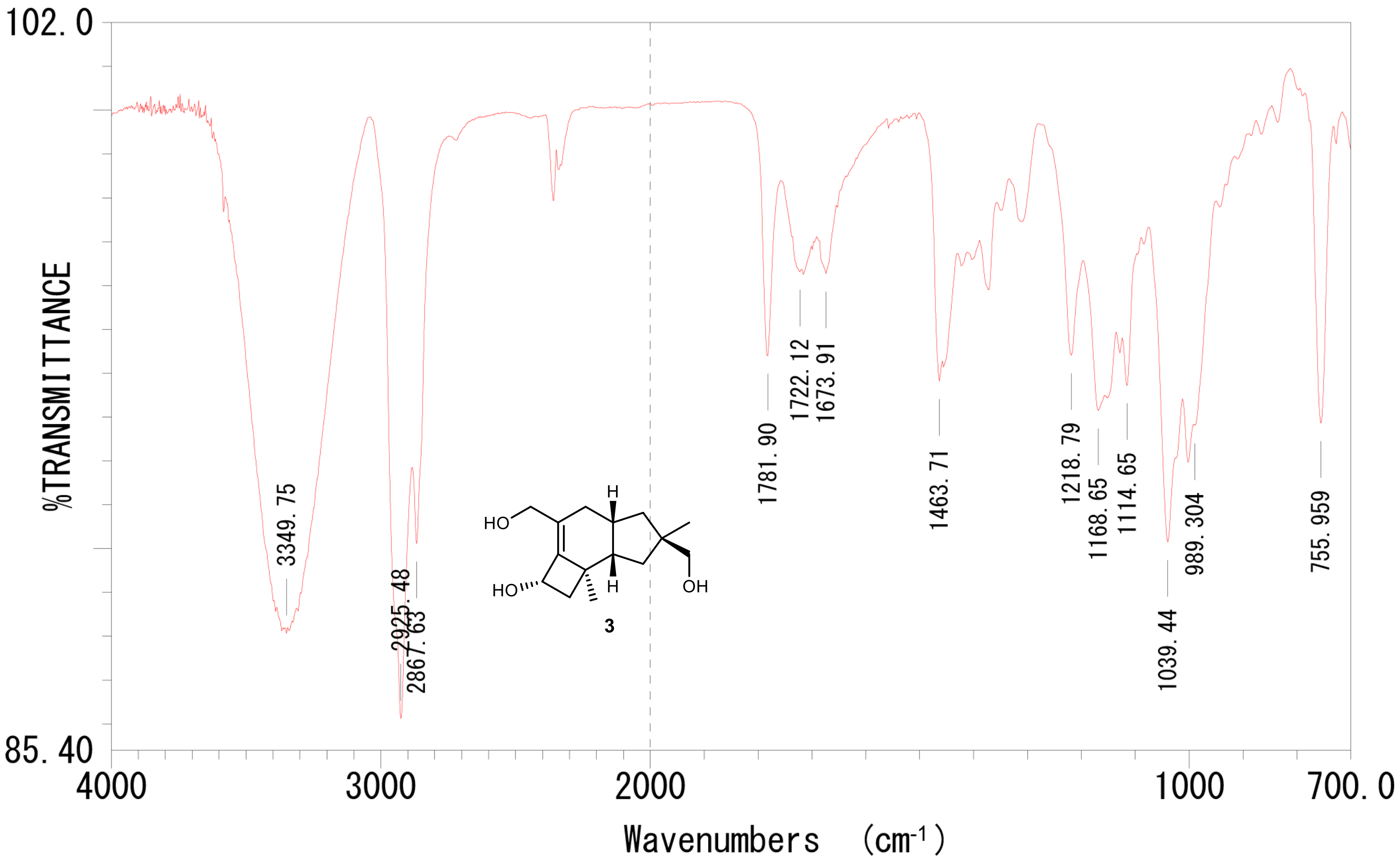

ESI-TOFMS spectrum of **3**.

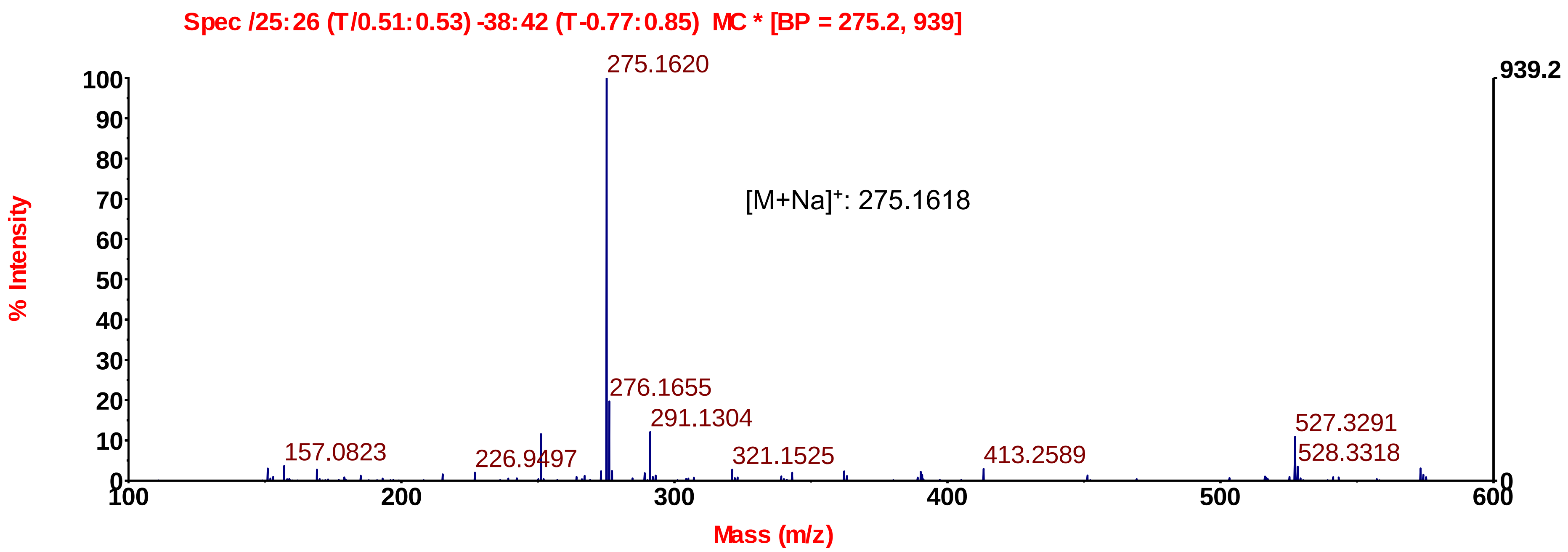

^{1}H NMR spectrum of **3** (500 MHz, $CDCl_3$)

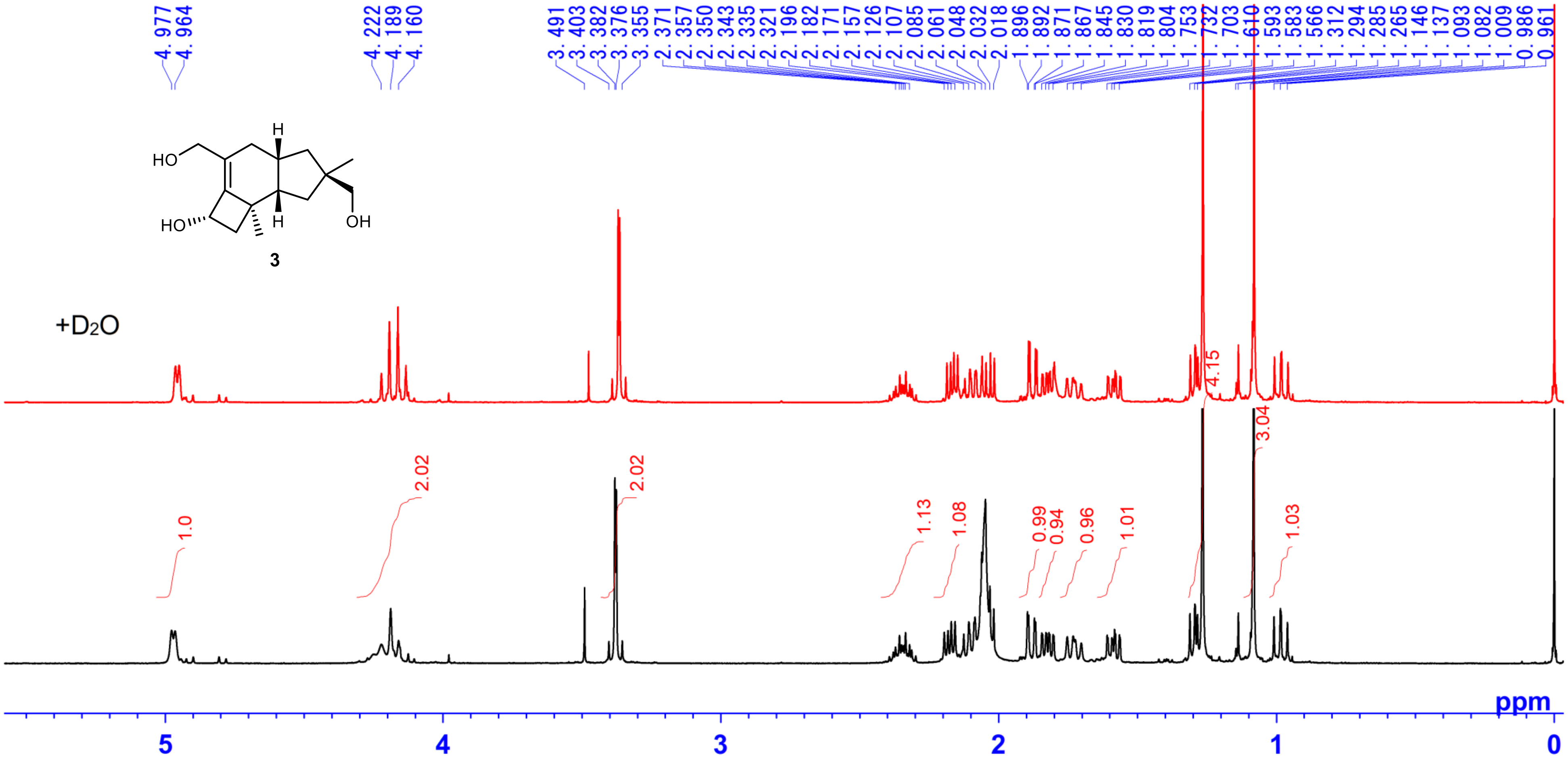

^{13}C NMR spectrum of **3** (125 MHz, $CDCl_3$)

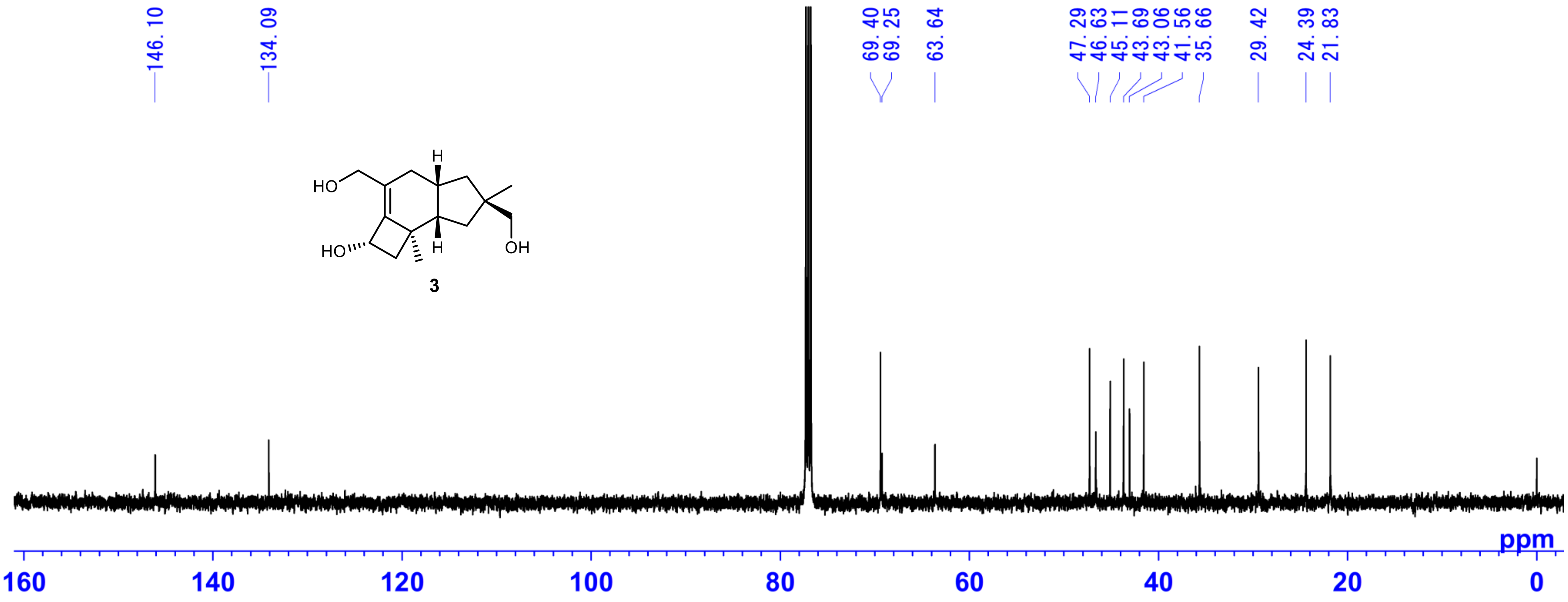

DQF COSY spectrum of **3** (500 MHz, $CDCl_3$)

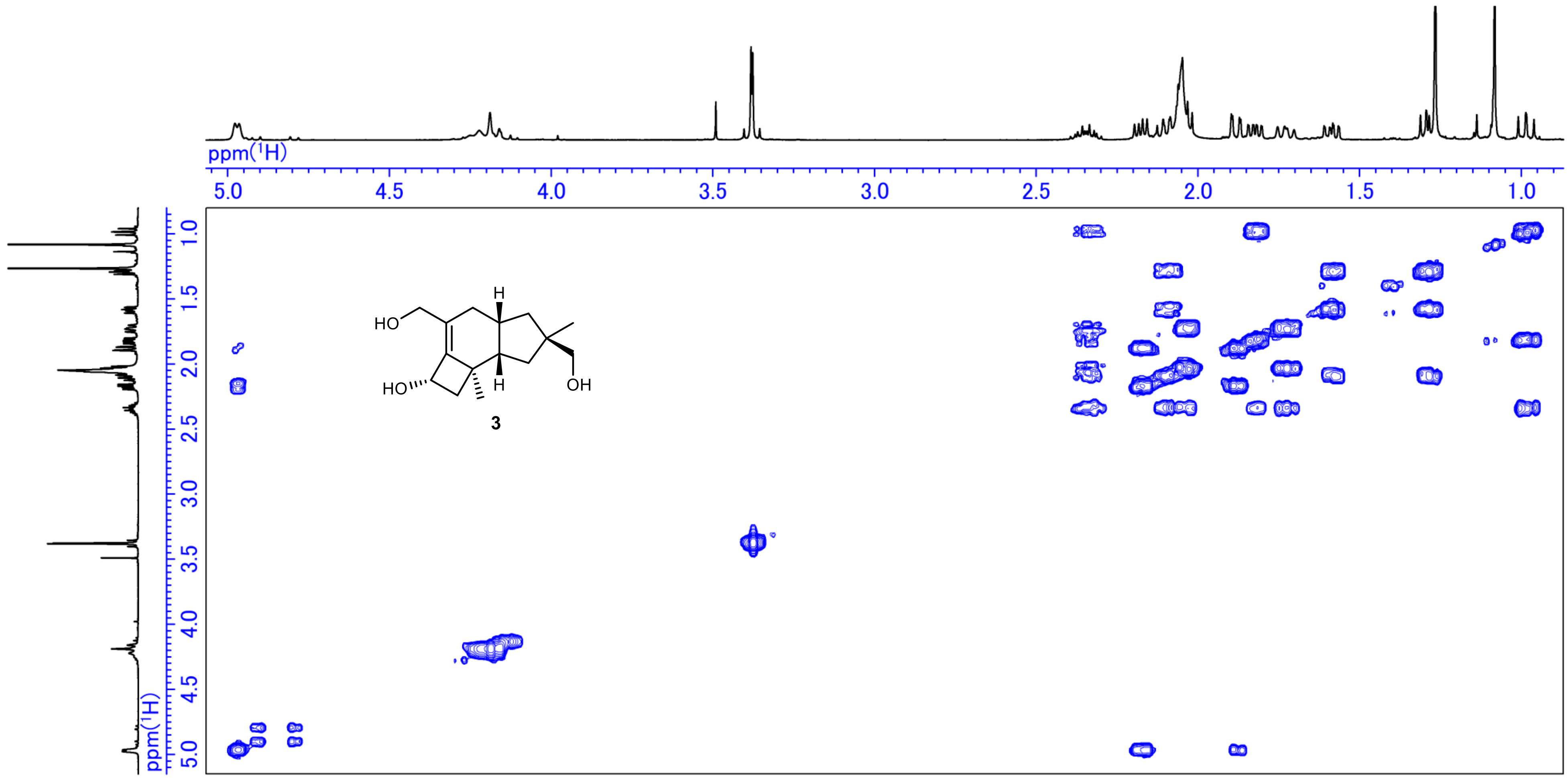

HSQC spectrum of **3** (500 MHz, $CDCl_3$)

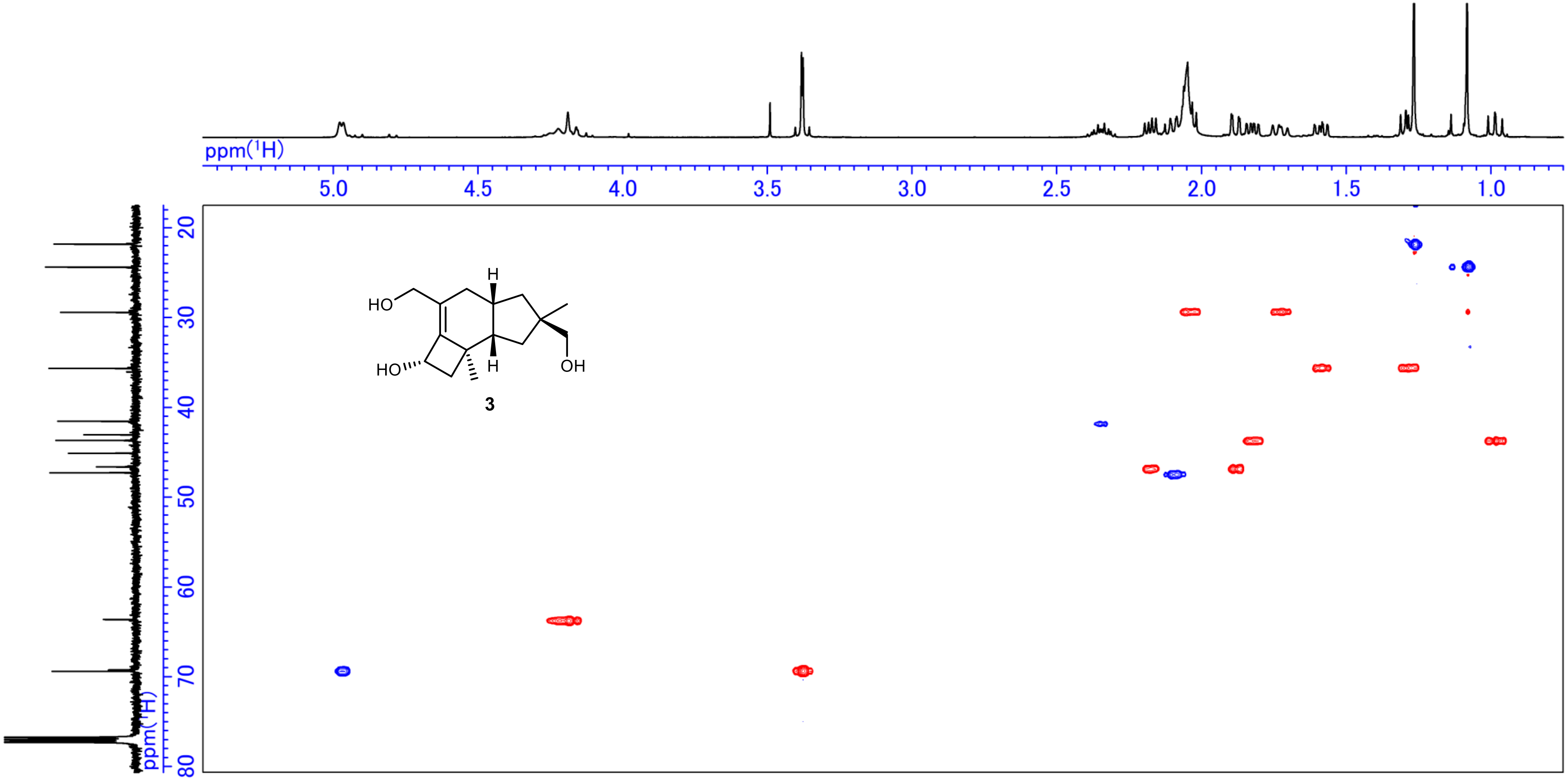

HMBC spectrum of **3** (500 MHz, $CDCl_3$)

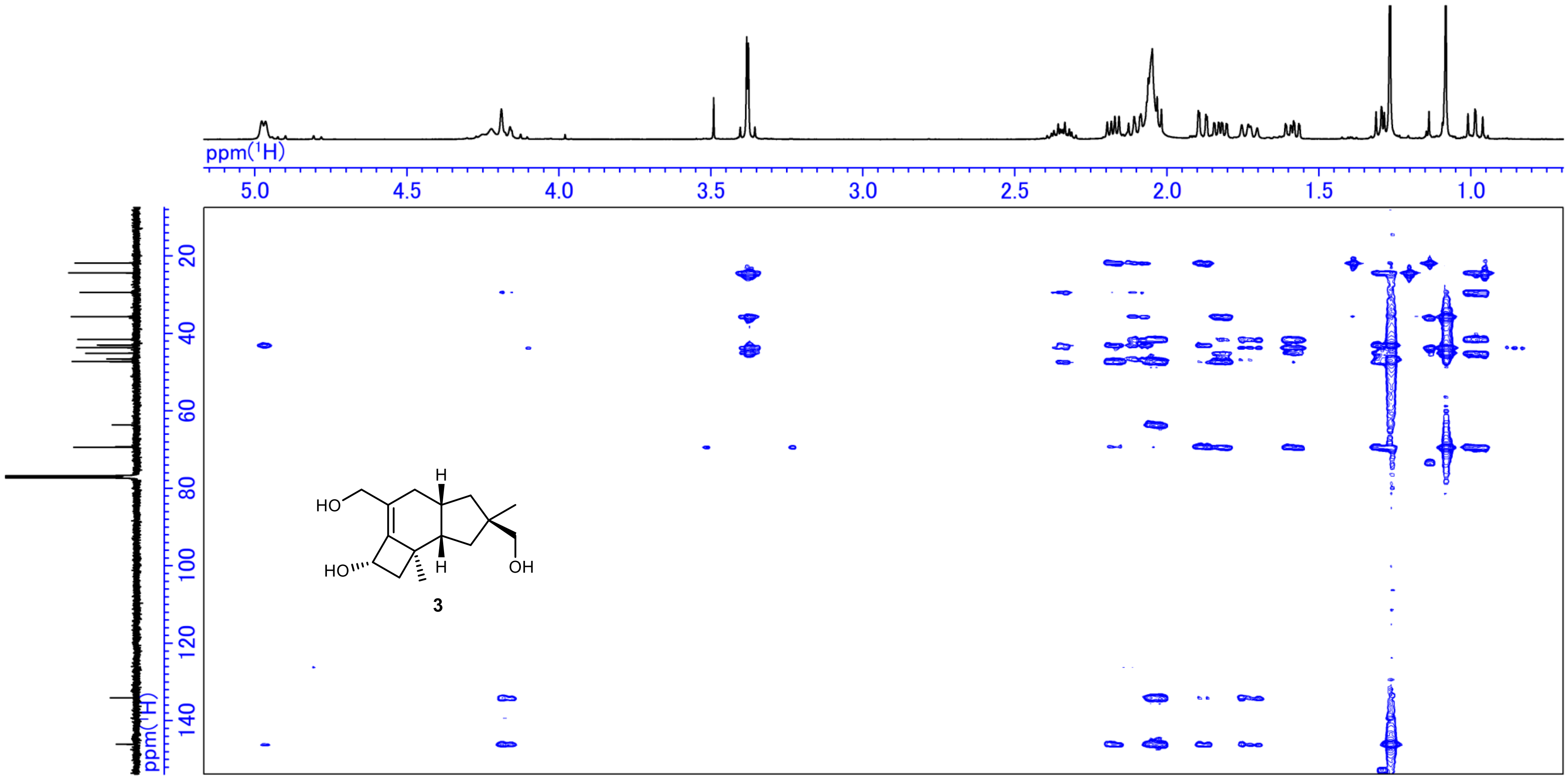

NOESY spectrum of **3** (500 MHz, $CDCl_3$)

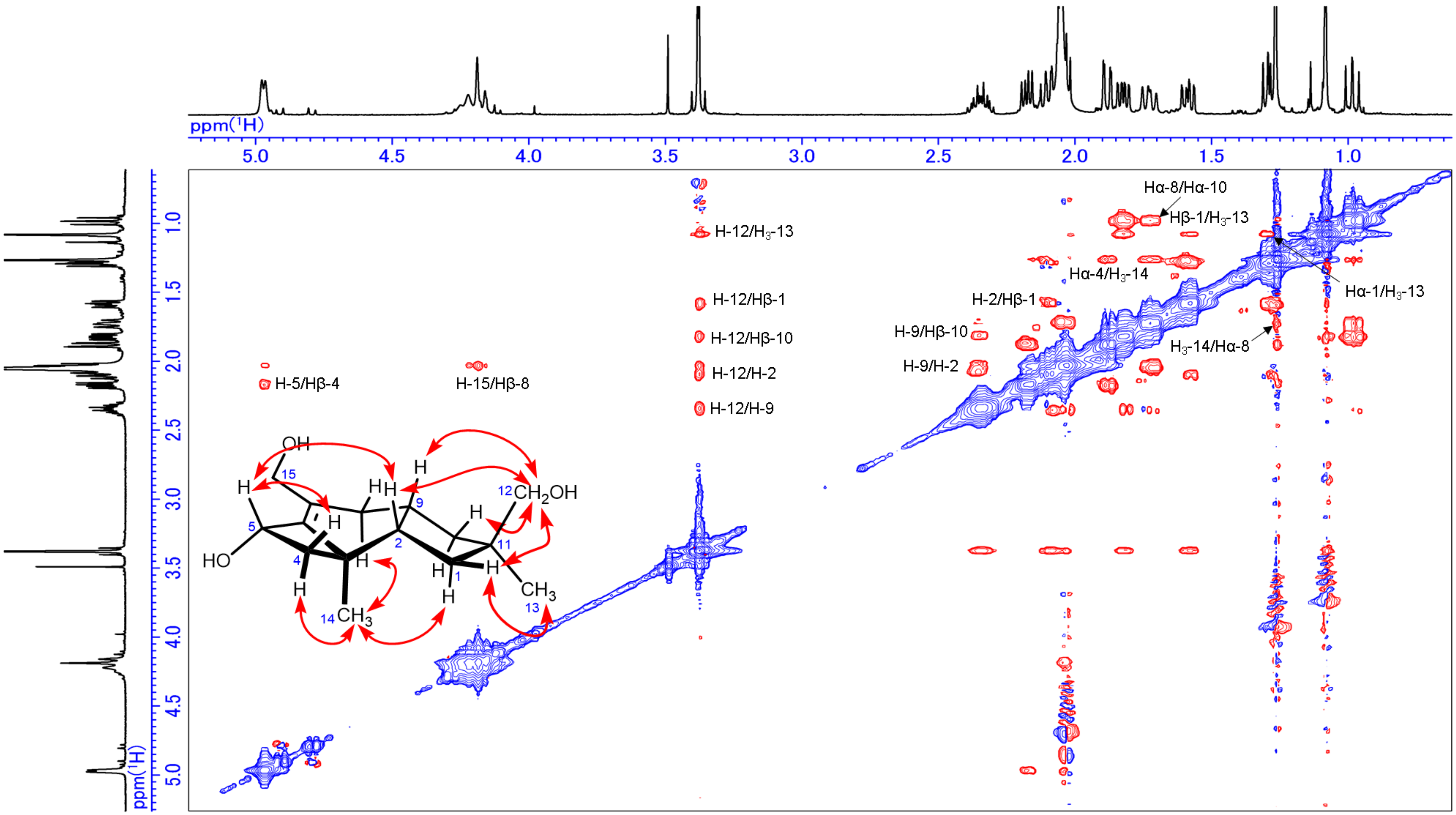

IR spectrum of **4** (film)

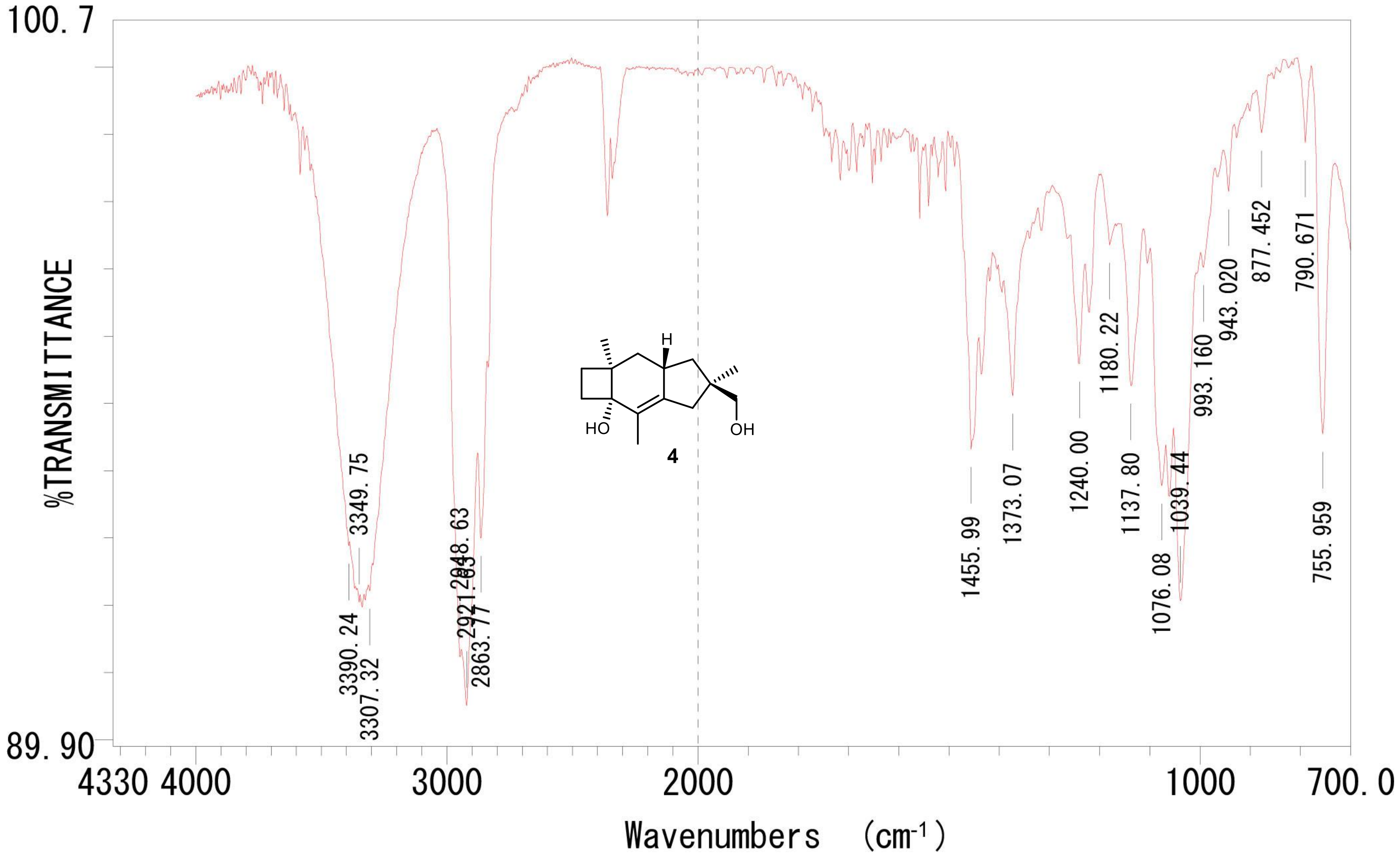

ESI-TOFMS spectrum of **4**.

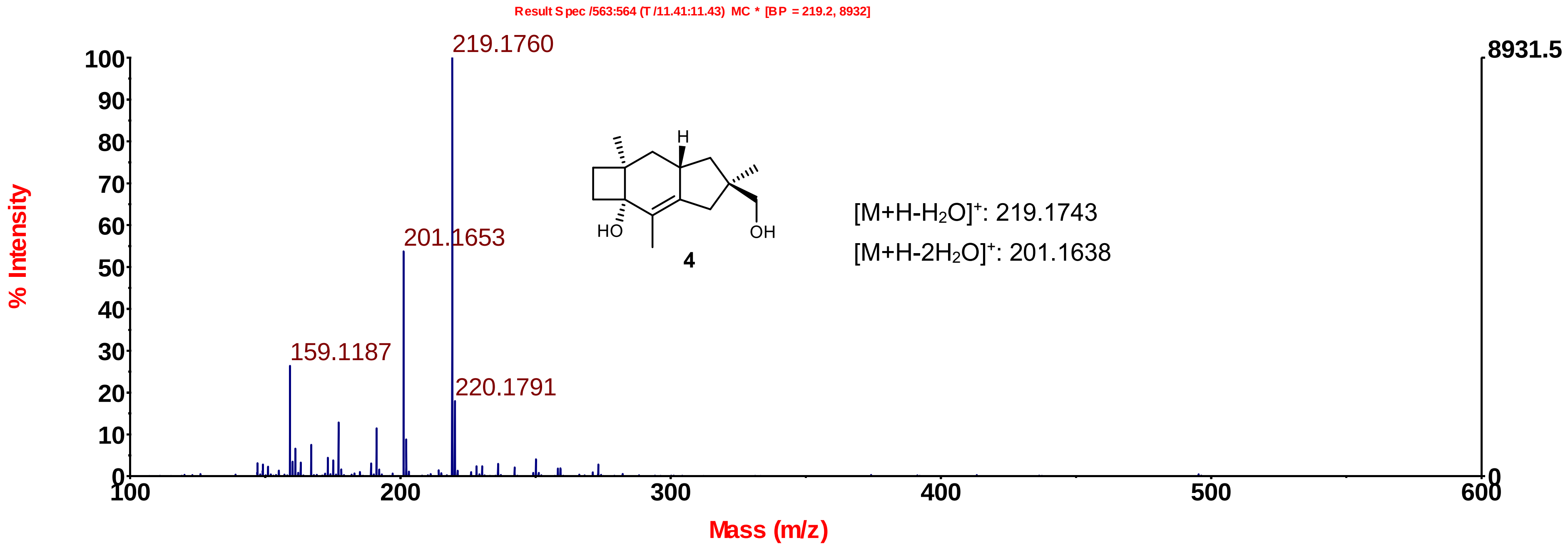

^{1}H NMR spectrum of **4** (500 MHz, $CDCl_3$)

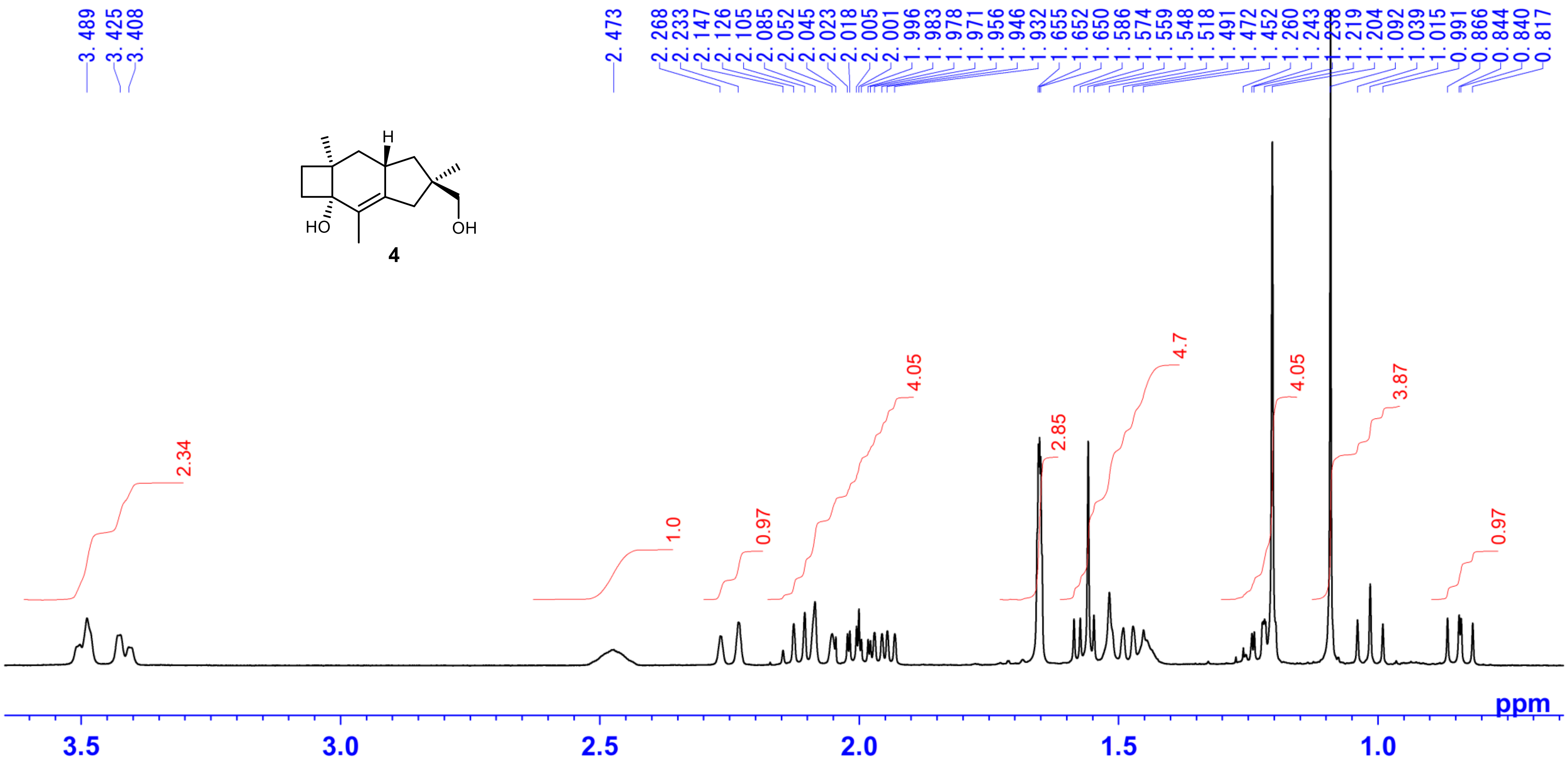

^{13}C NMR spectrum of **4** (125 MHz, $CDCl_3$)

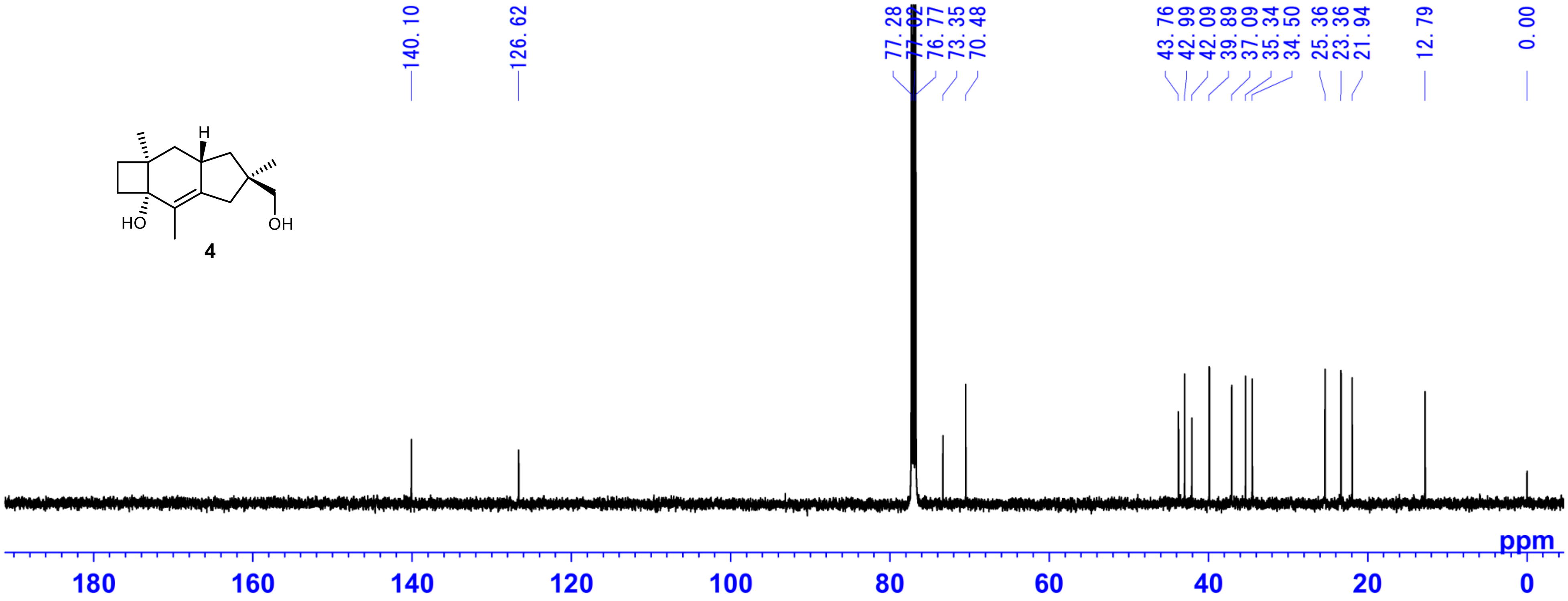

DQF COSY spectrum of **4** (500 MHz, $CDCl_3$)

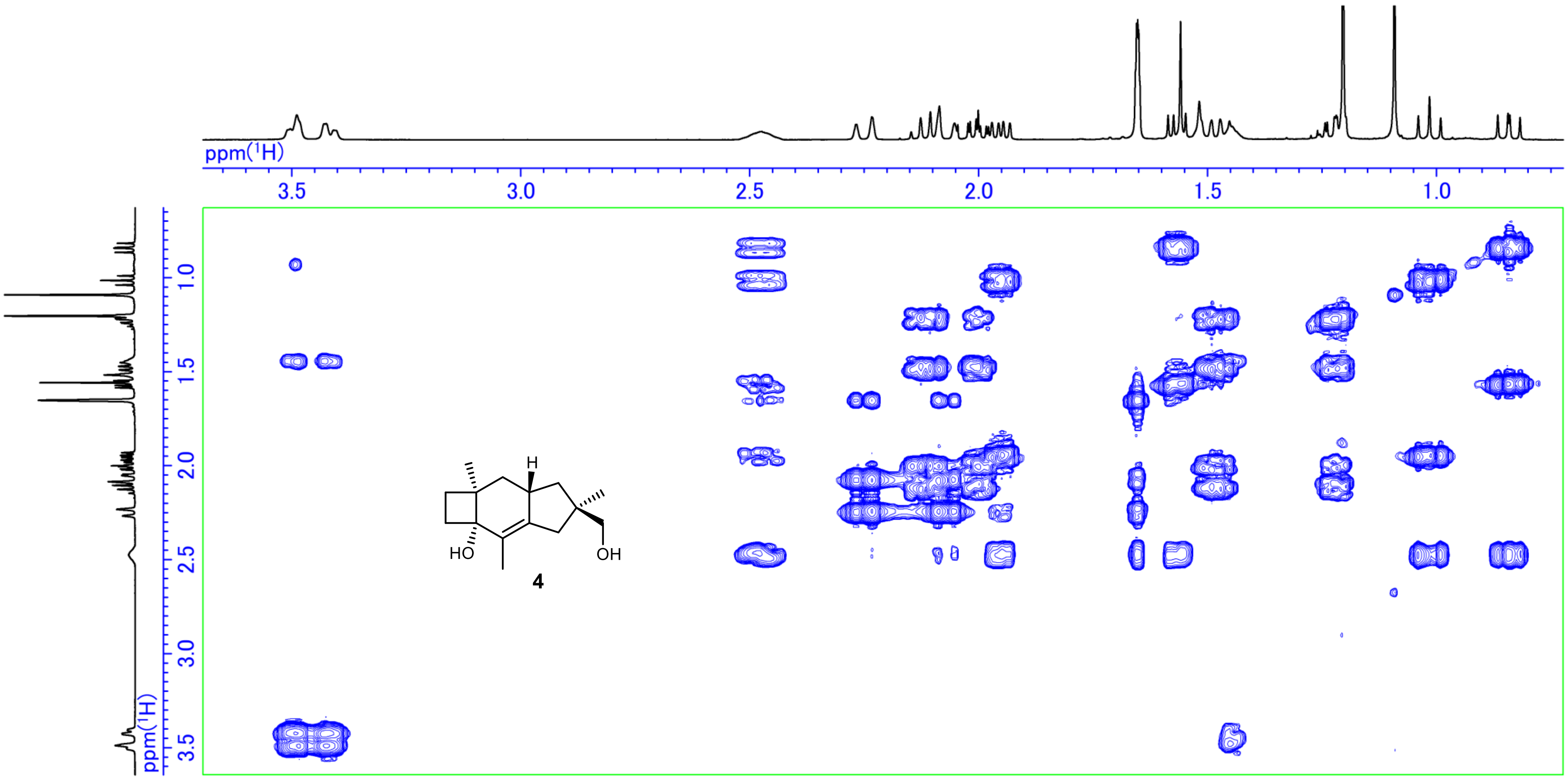

HSQC spectrum of **4** (500 MHz, $CDCl_3$)

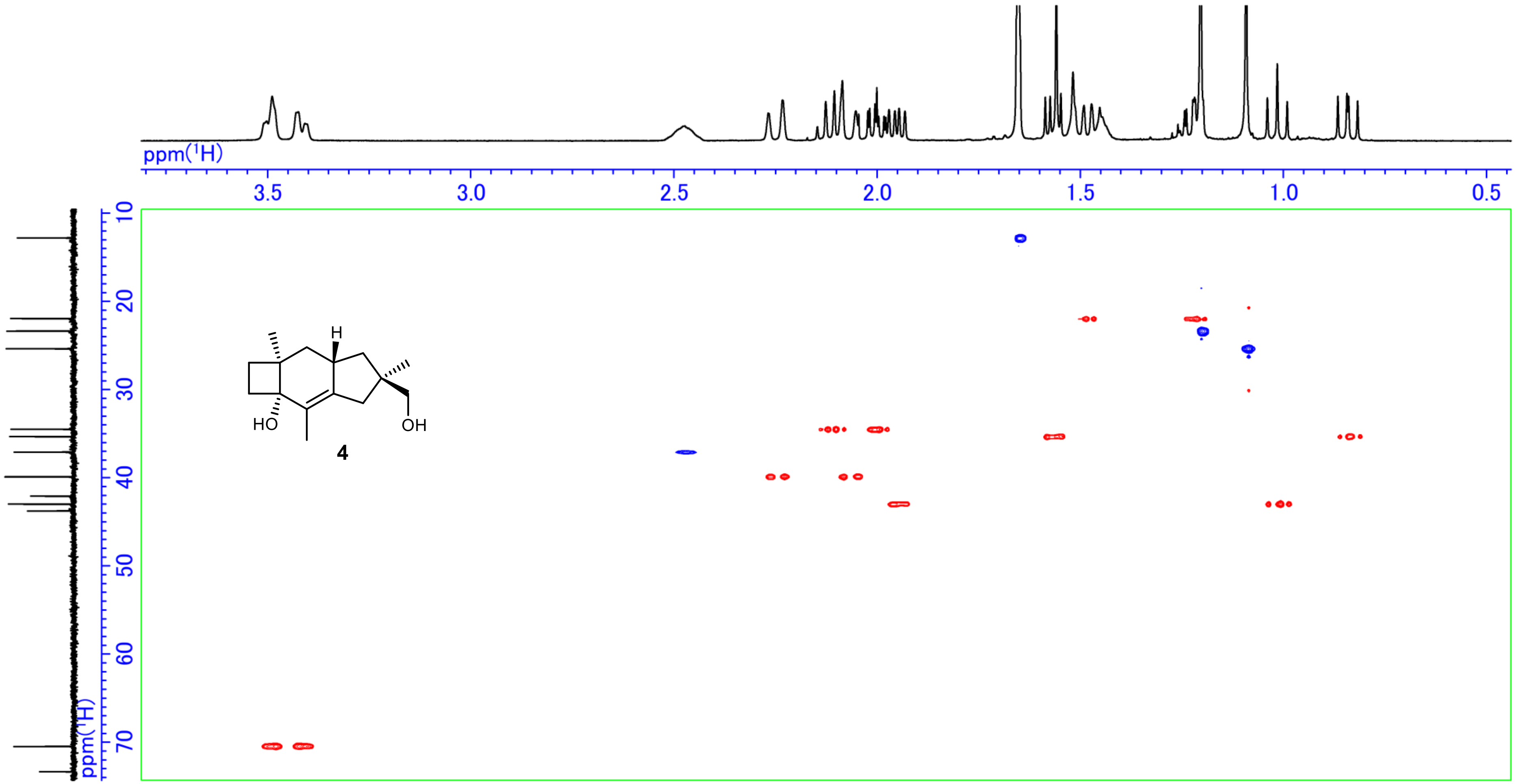

HMBC spectrum of **4** (500 MHz, $CDCl_3$)

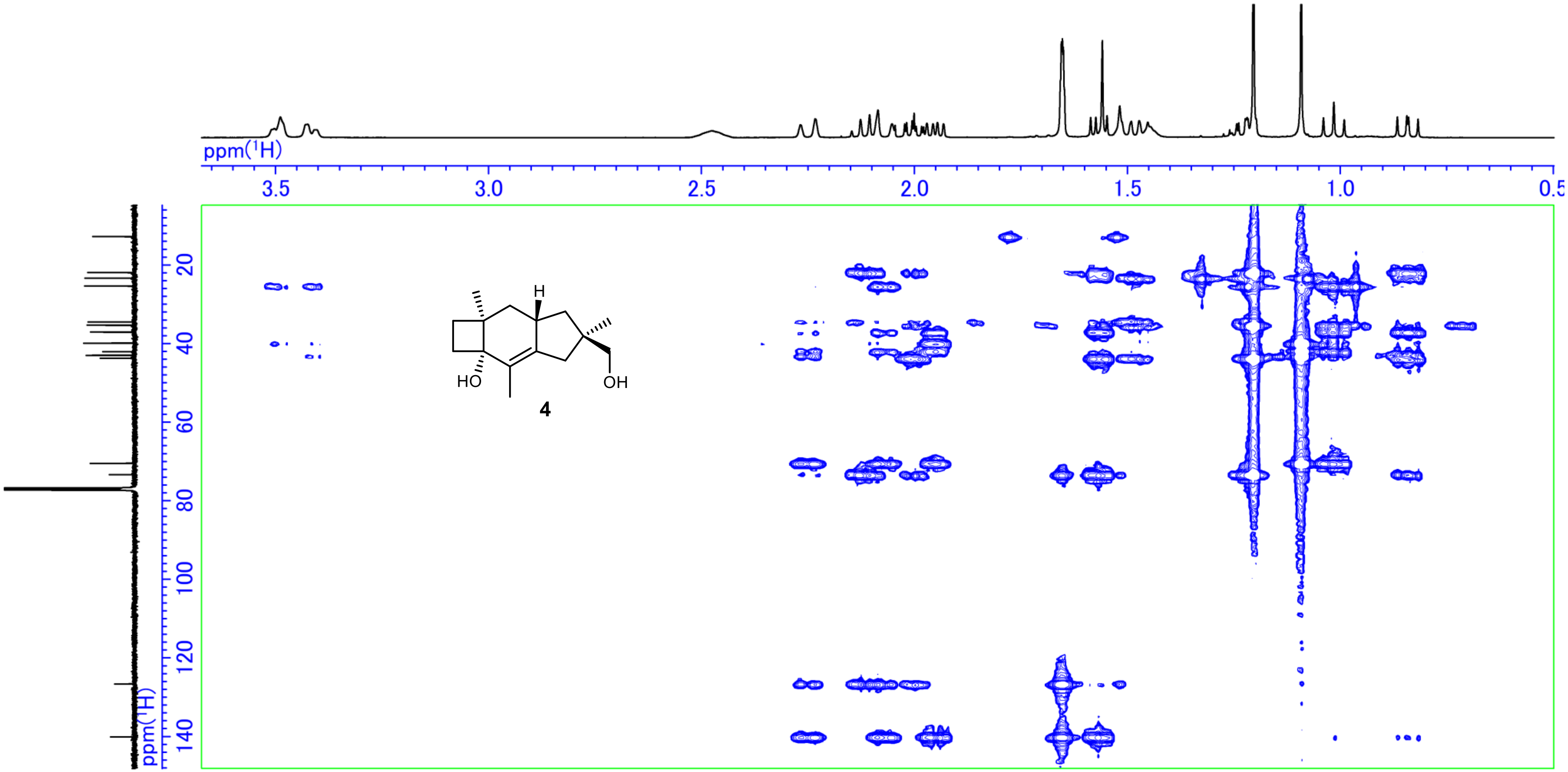

NOE 1D spectra of **4** (500 MHz, $CDCl_3$)

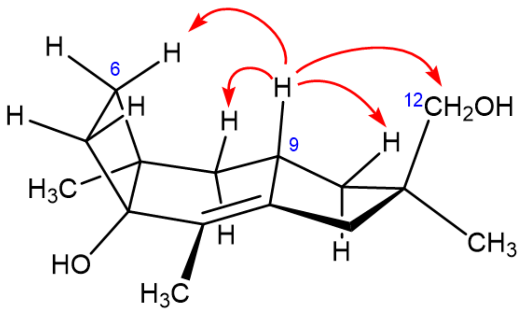

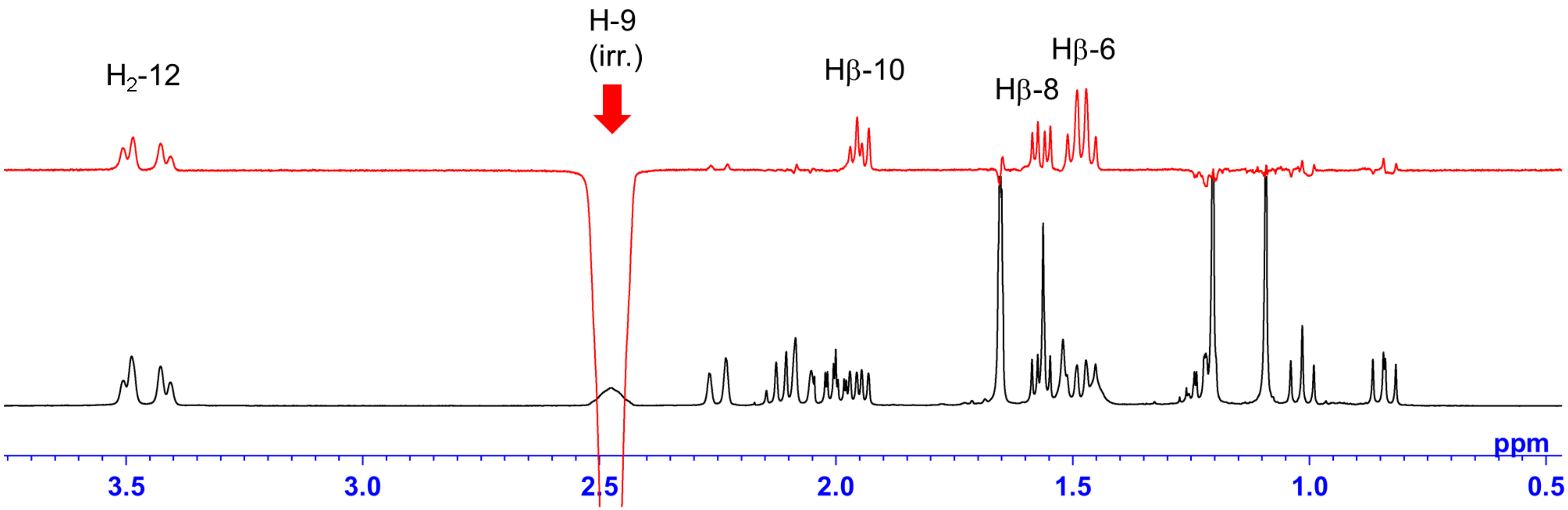

NOESY spectrum of **4** (500 MHz, $CDCl_3$)

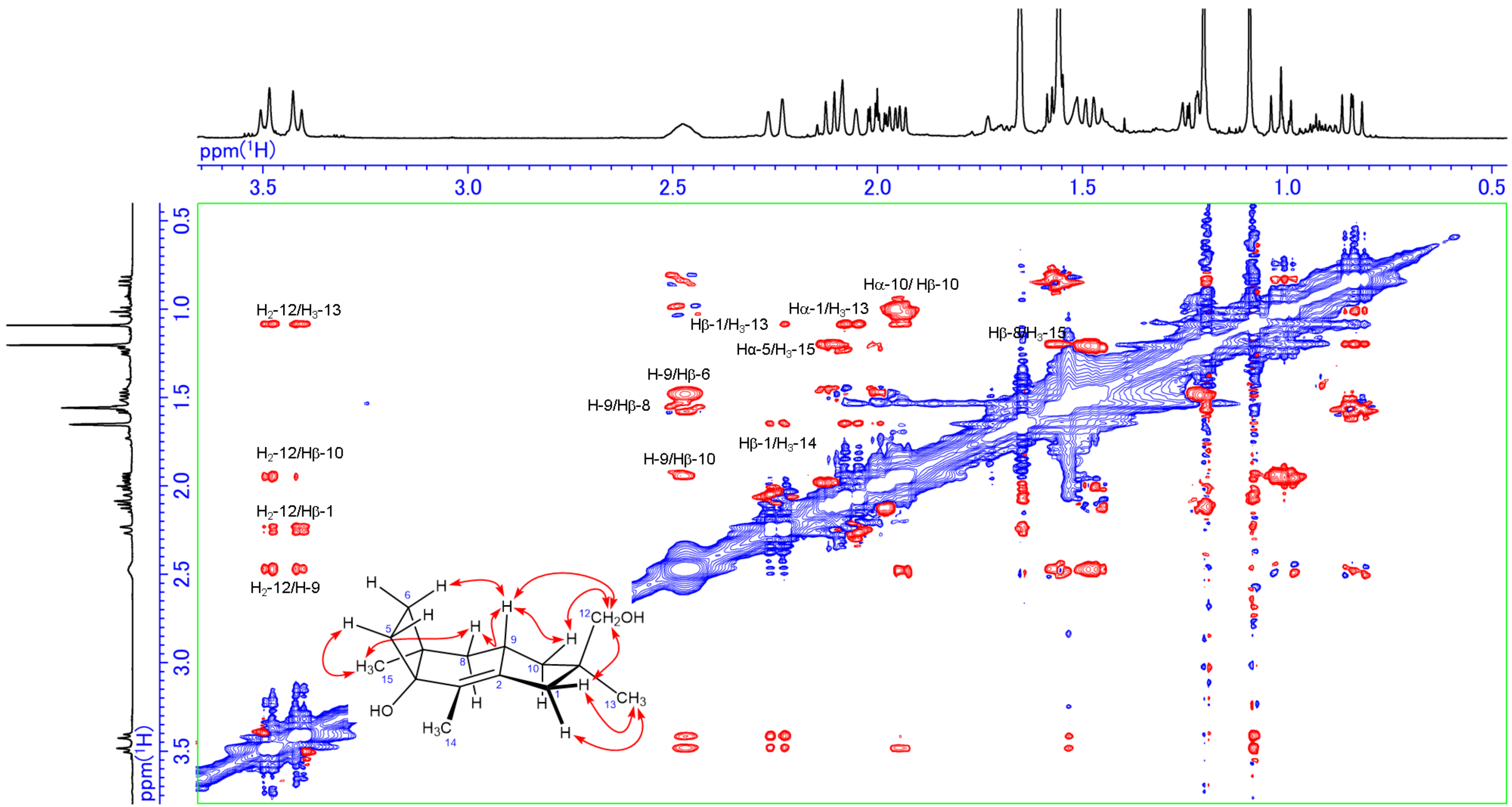

IR spectrum of **5** (film)

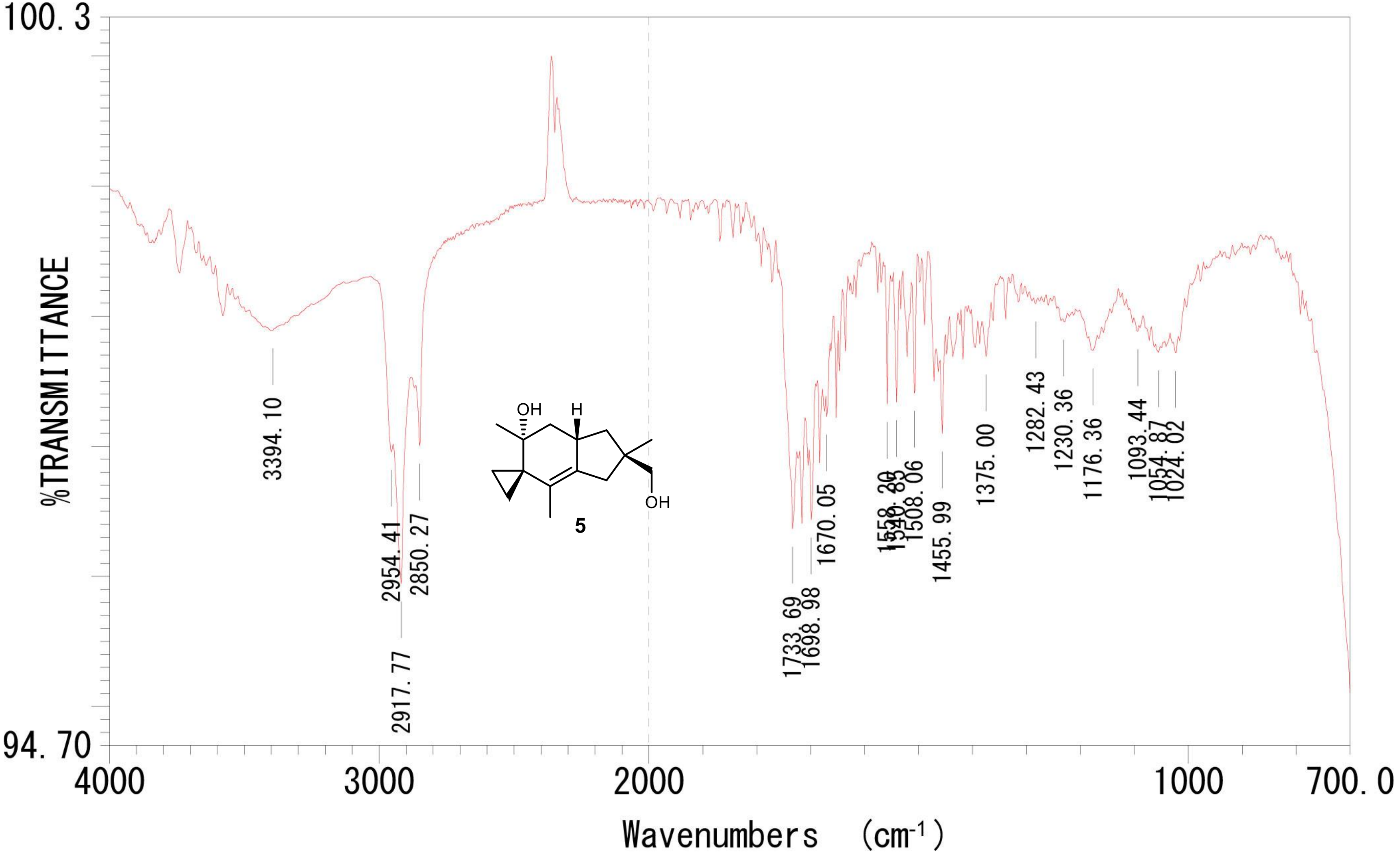

ESI-TOFMS spectrum of **5**.

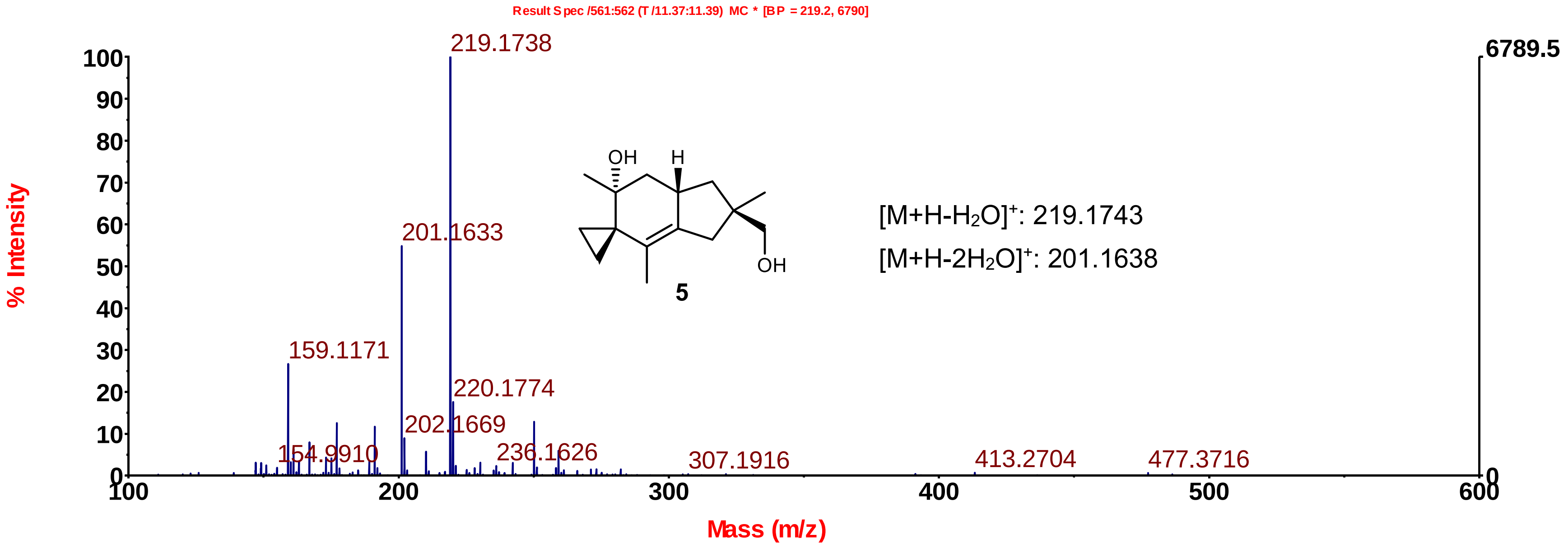

^{1}H NMR spectrum of **5** (500 MHz, $CDCl_3$)

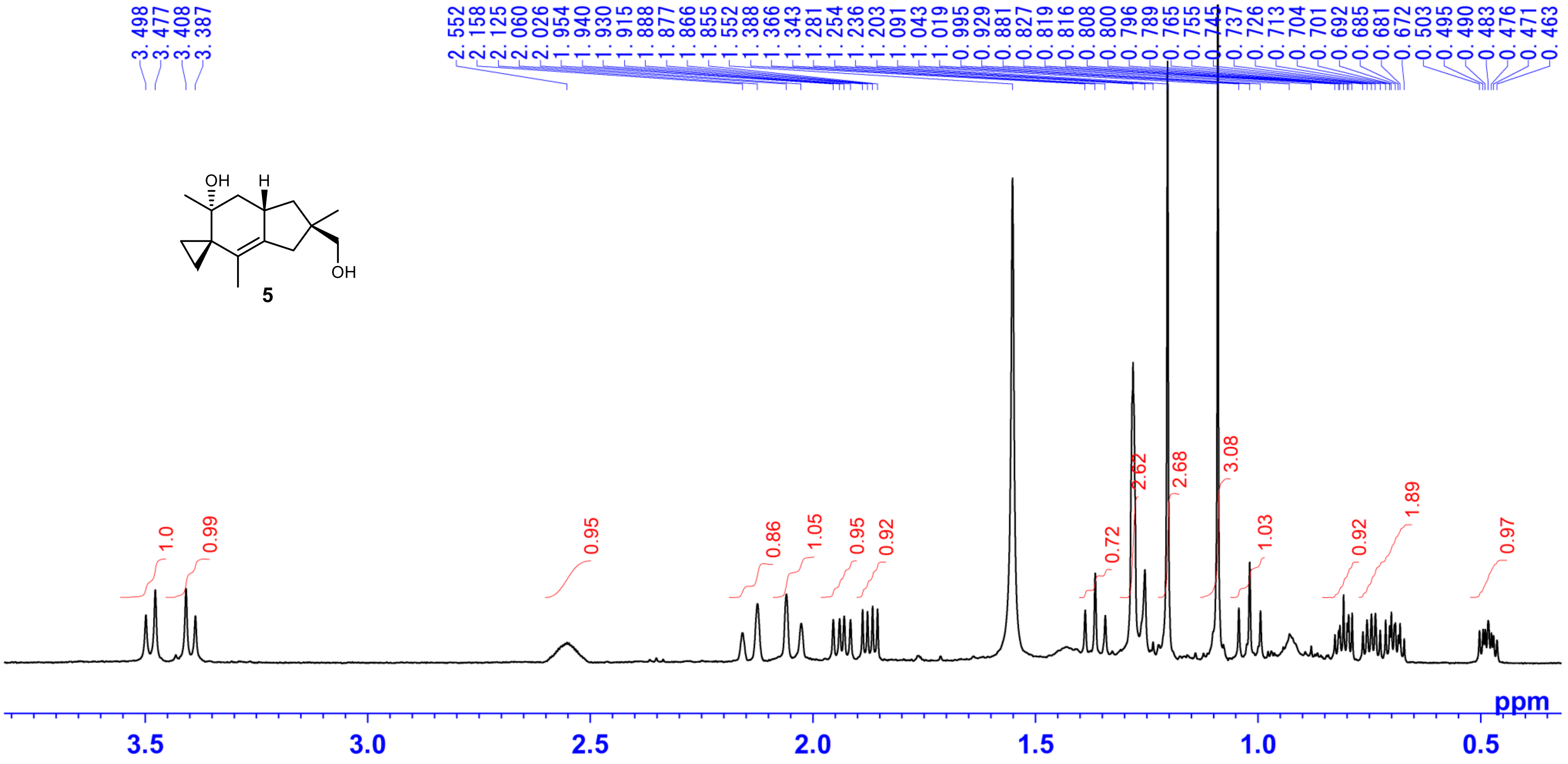

^{13}C NMR spectrum of **5** (125 MHz, $CDCl_3$)

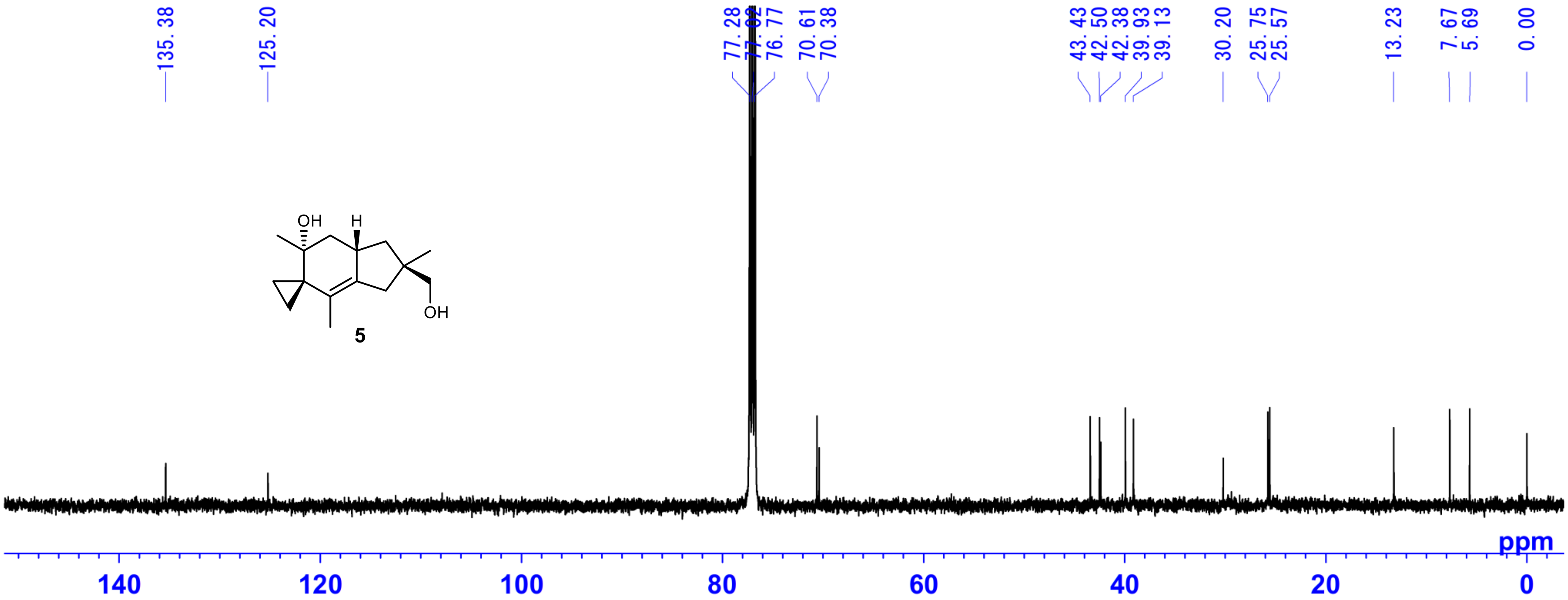

DQF COSY spectrum of **5** (500 MHz, $CDCl_3$)

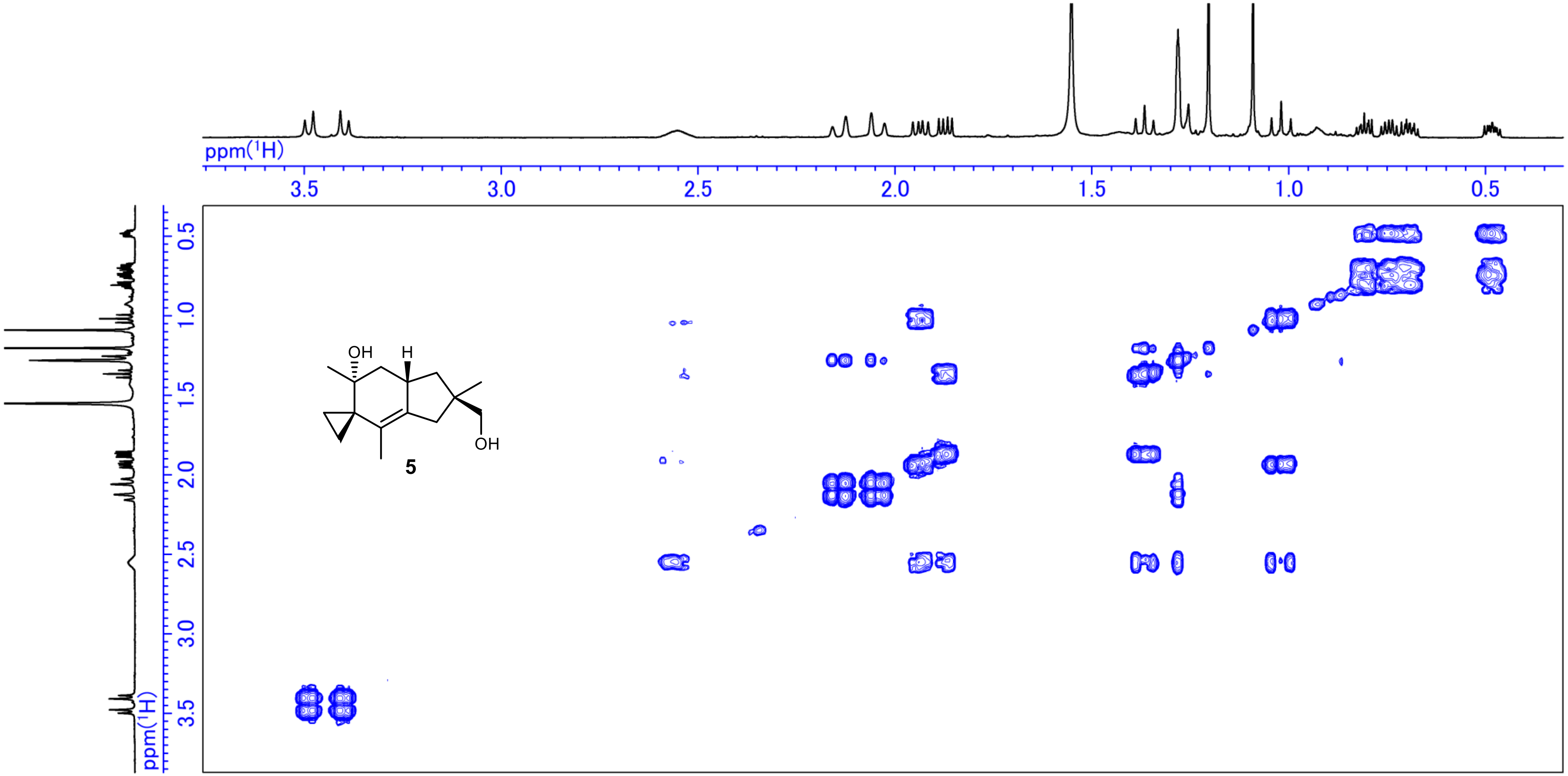

HMQC spectrum of **5** (500 MHz, $CDCl_3$)

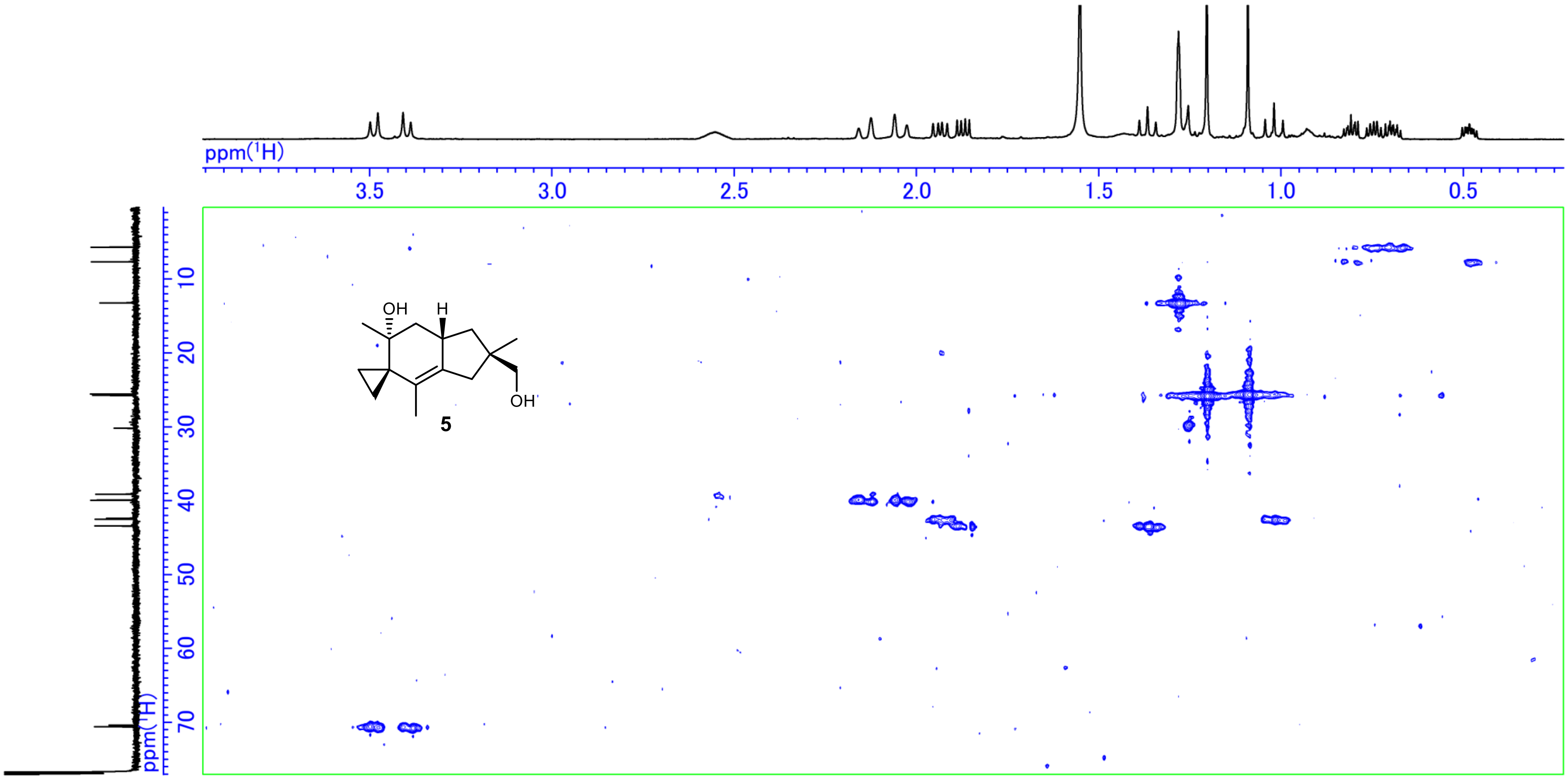

HMBC spectrum of **5** (500 MHz, $CDCl_3$)

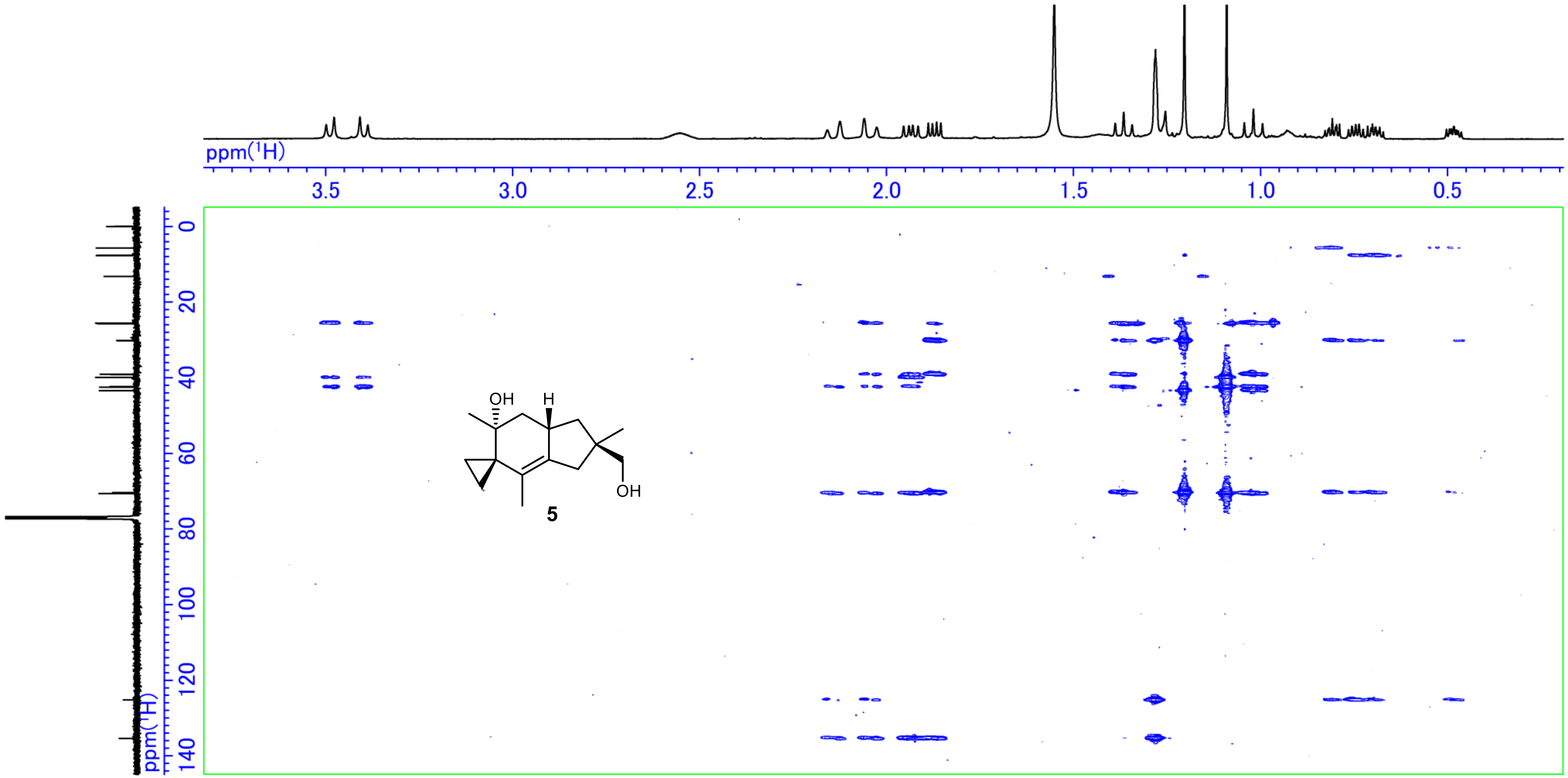

NOE 1D spectra of **5** (500 MHz, $CDCl_3$)

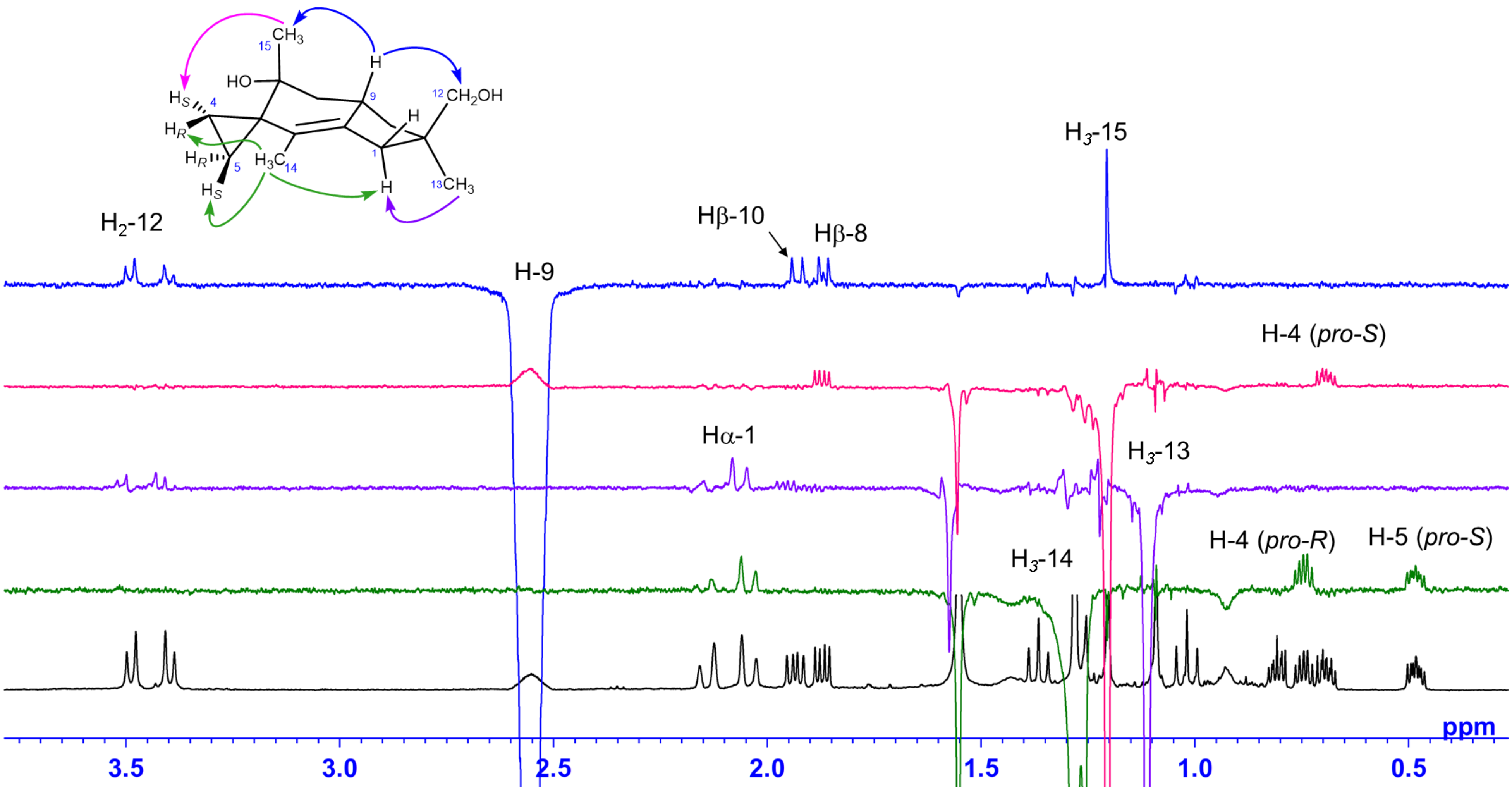

IR spectrum of **6** (film)

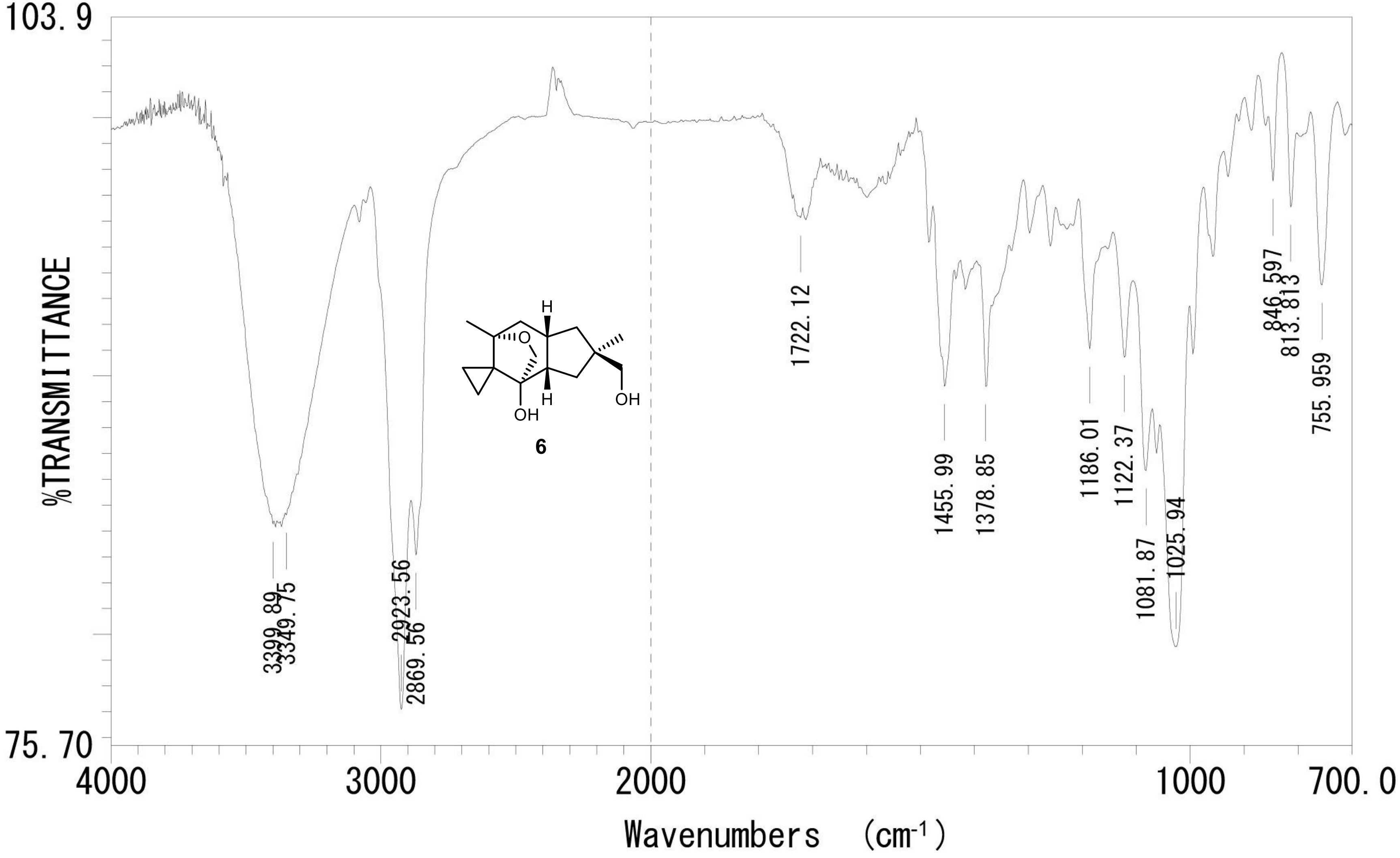

ESI-TOFMS spectrum of **6**

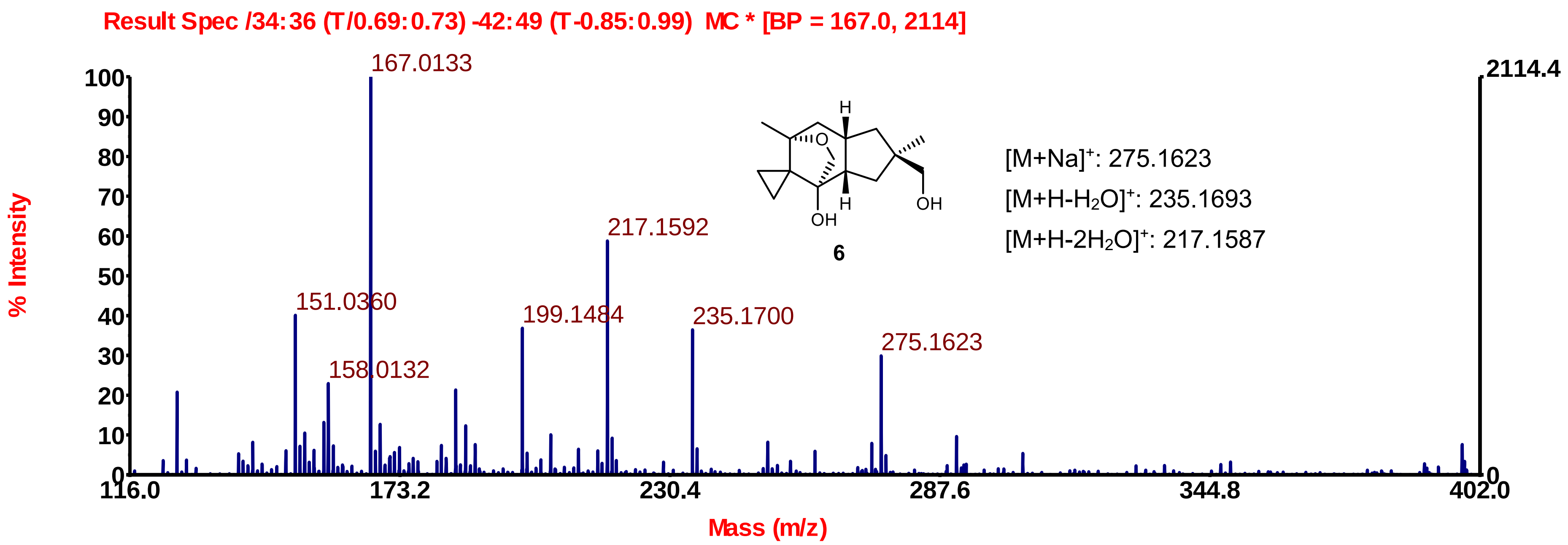

^1H NMR spectrum of **6** (500 MHz, $CDCl_3$)

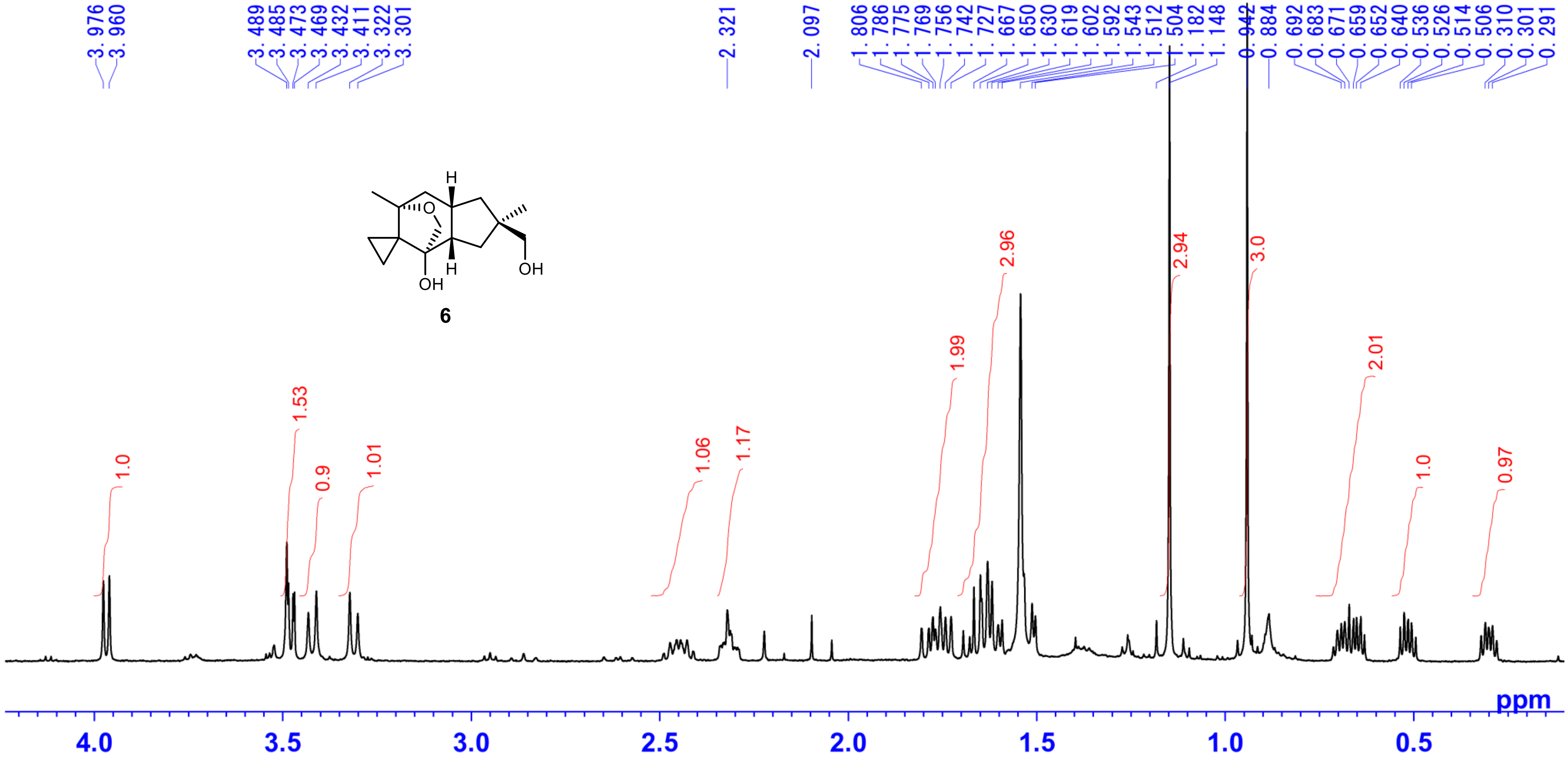

^{13}C NMR spectrum of **6** (125 MHz, $CDCl_3$)

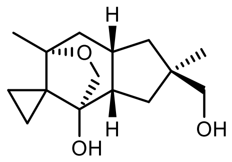

6

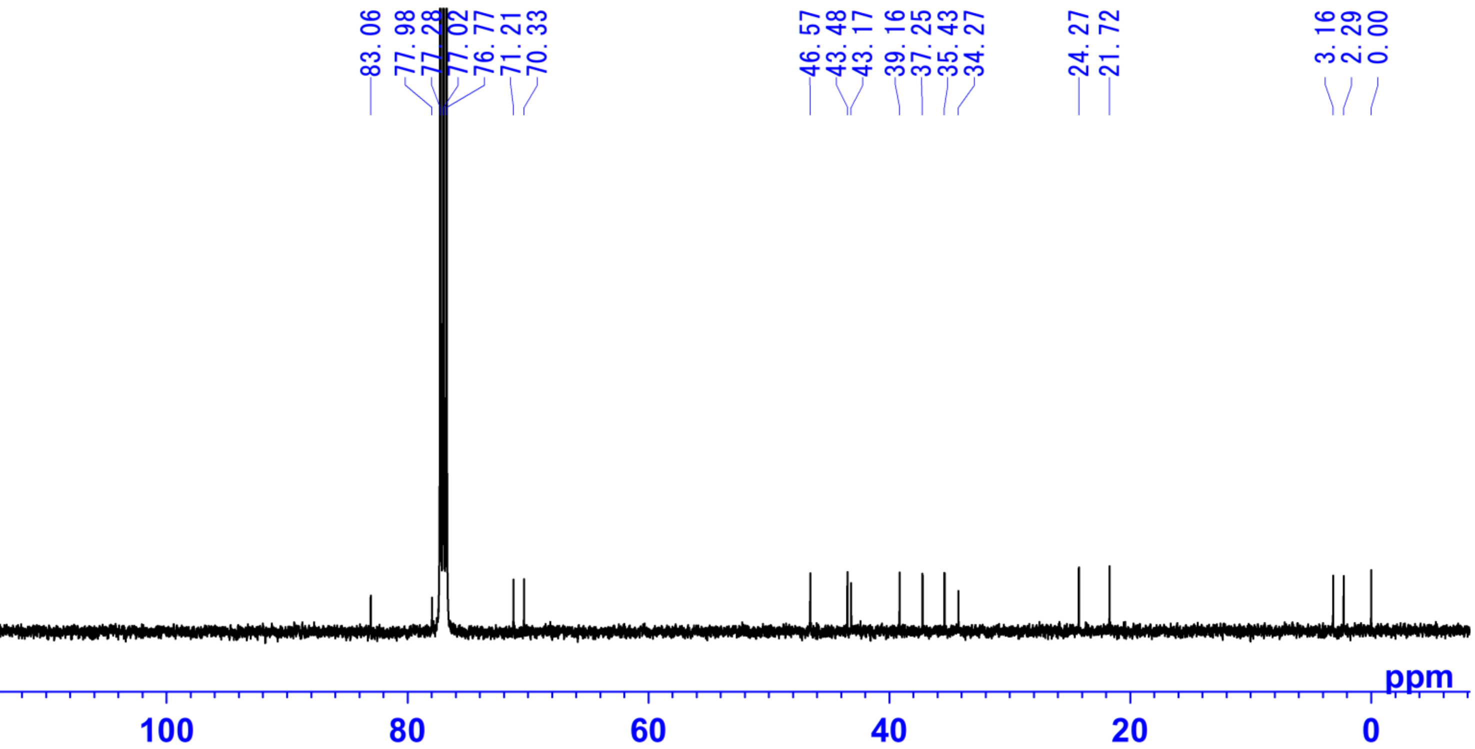

DQF COSY spectrum of **6** (500 MHz, $CDCl_3$)

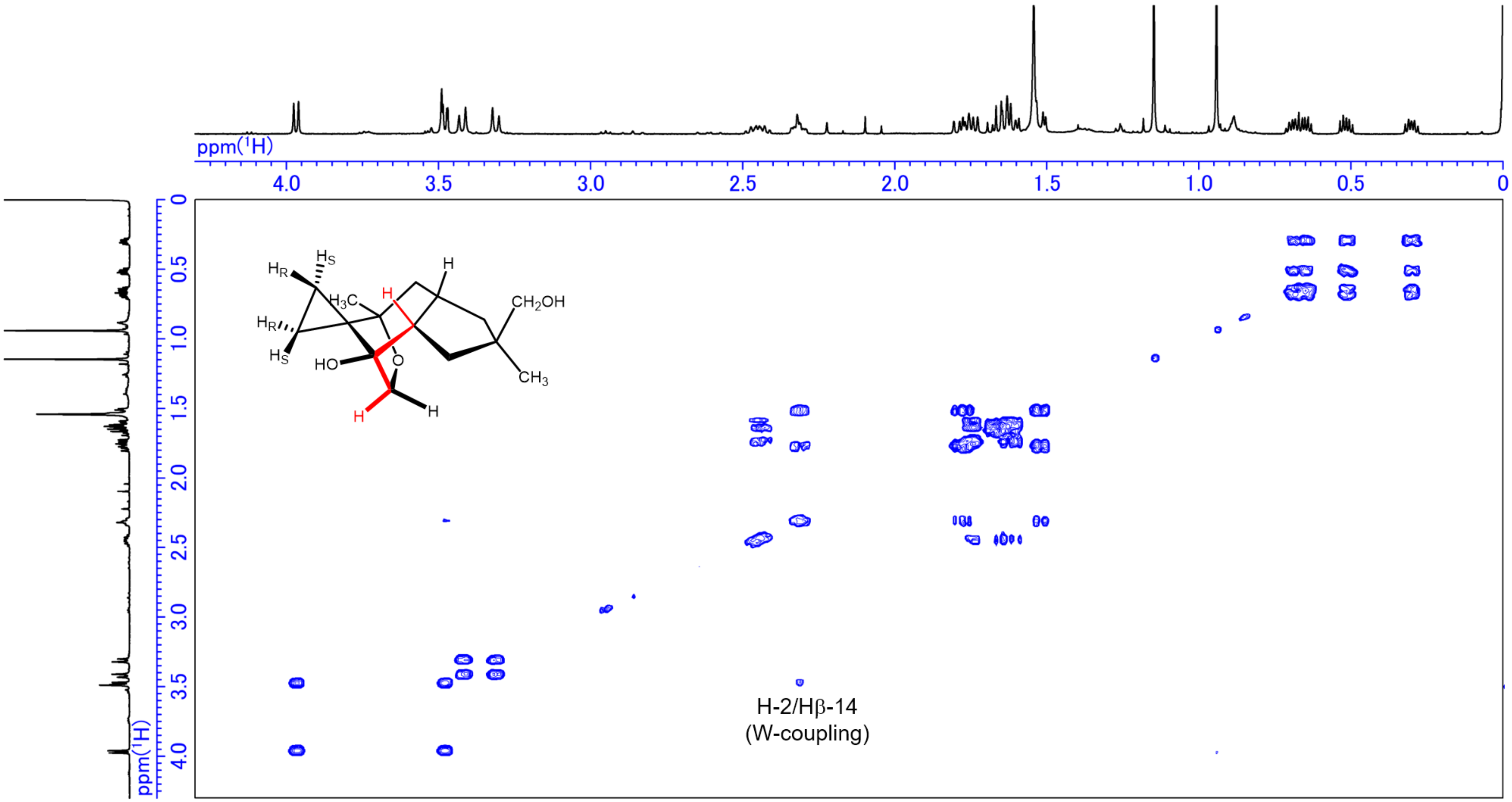

HSQC spectrum of **6** (500 MHz, $CDCl_3$)

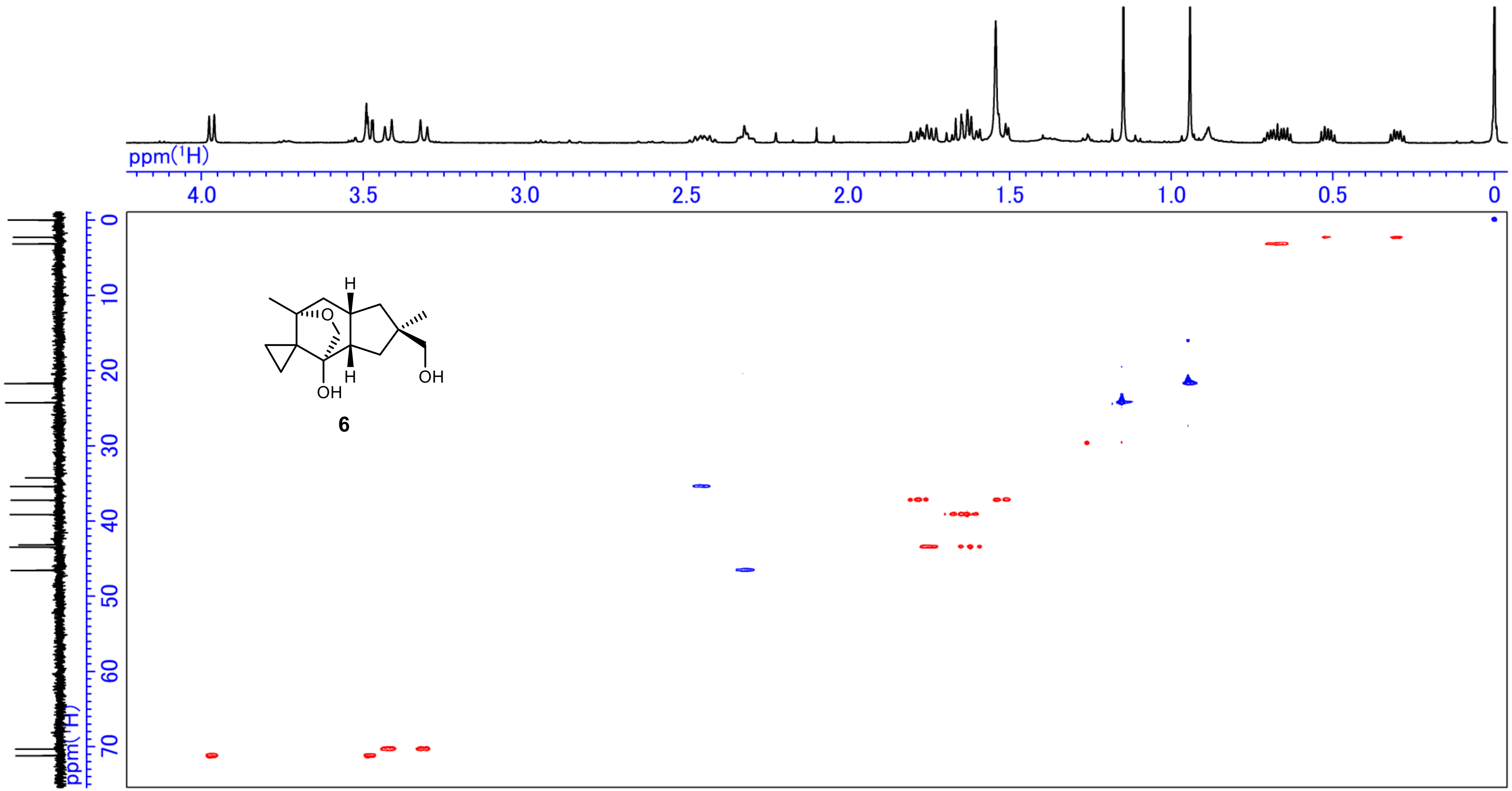

HMBC spectrum of **6** (500 MHz, $CDCl_3$)

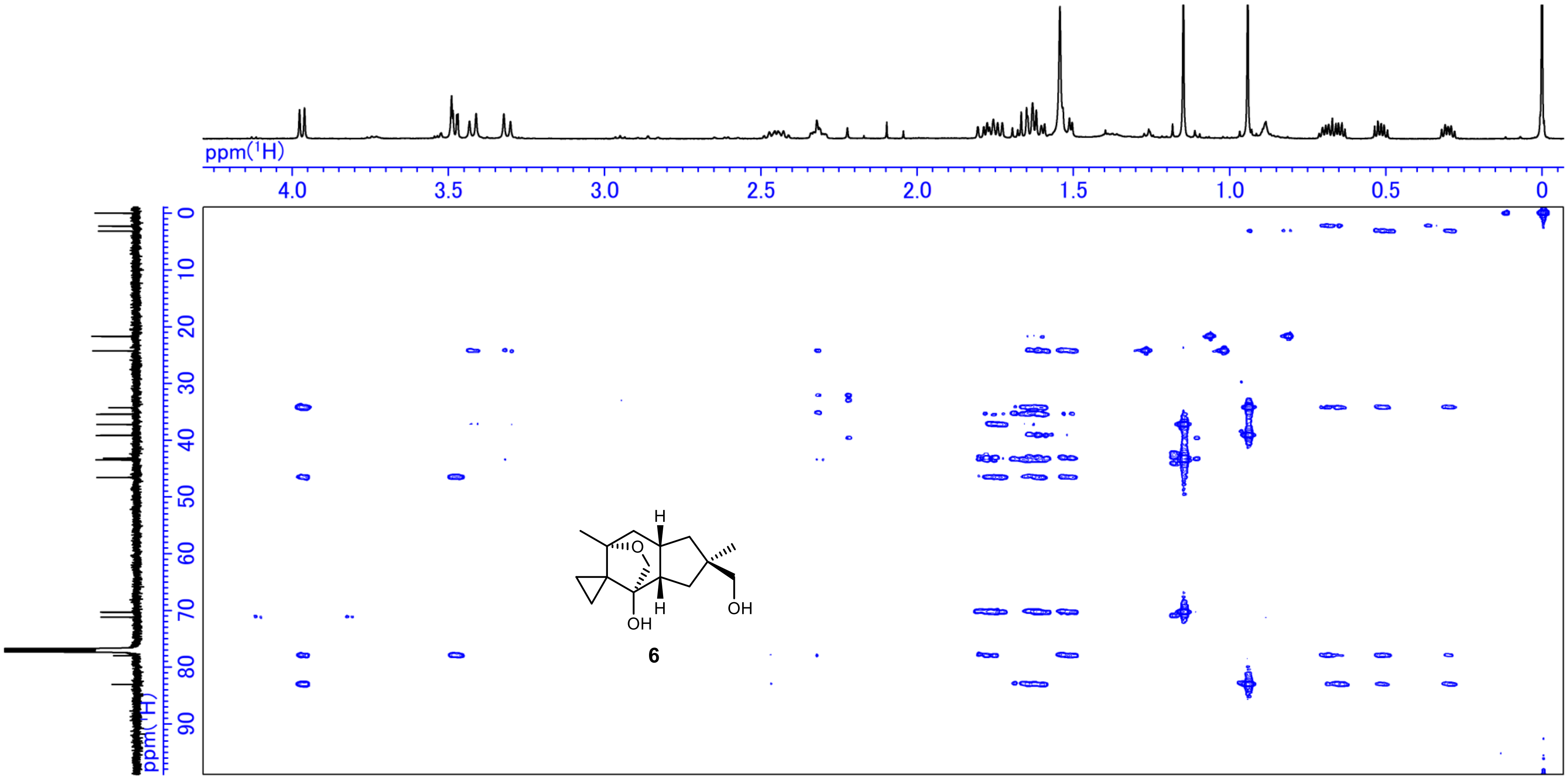

NOE 1D spectra of **6** (500 MHz, $CDCl_3$)

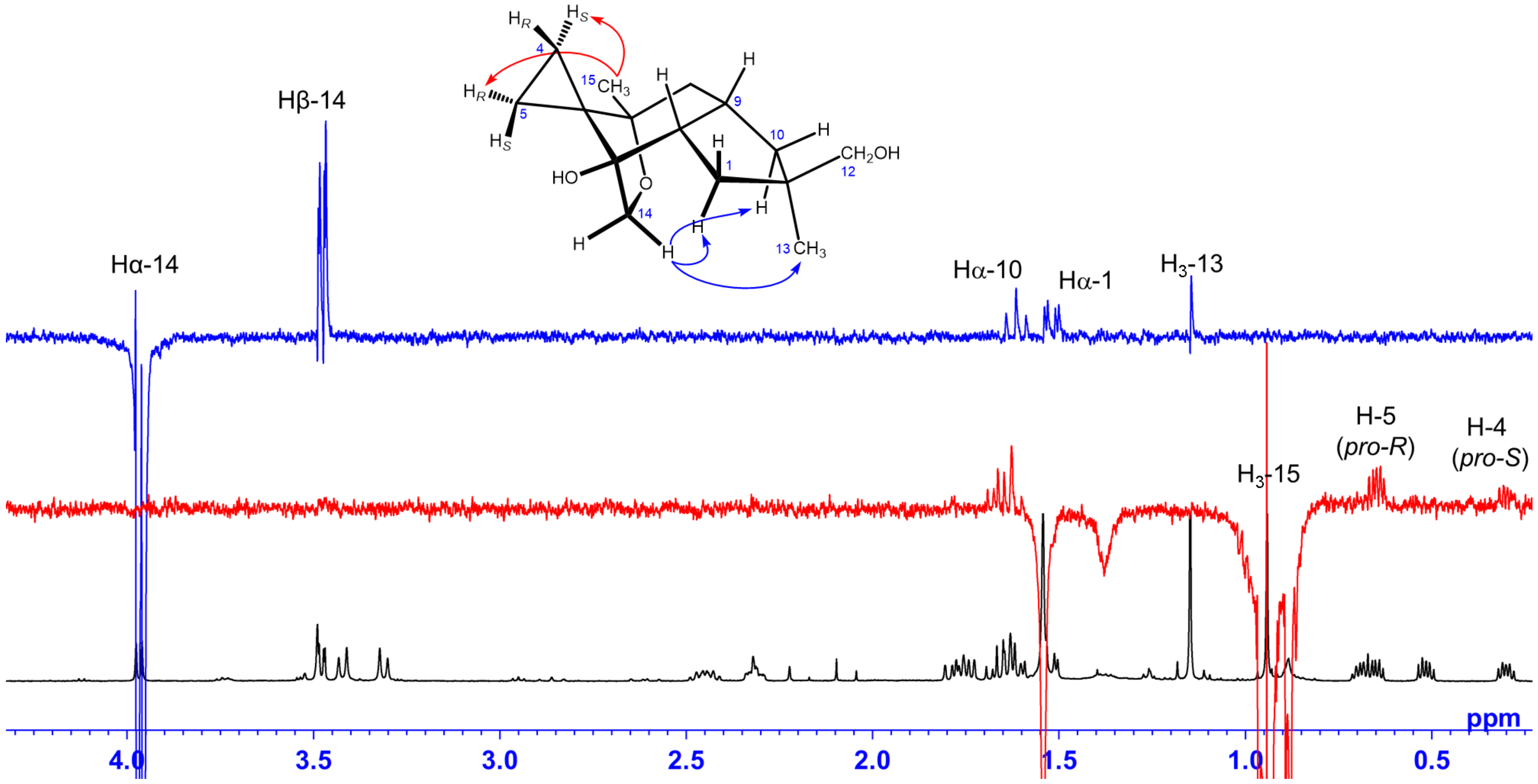

NOESY spectrum of **6** (500 MHz, $CDCl_3$)

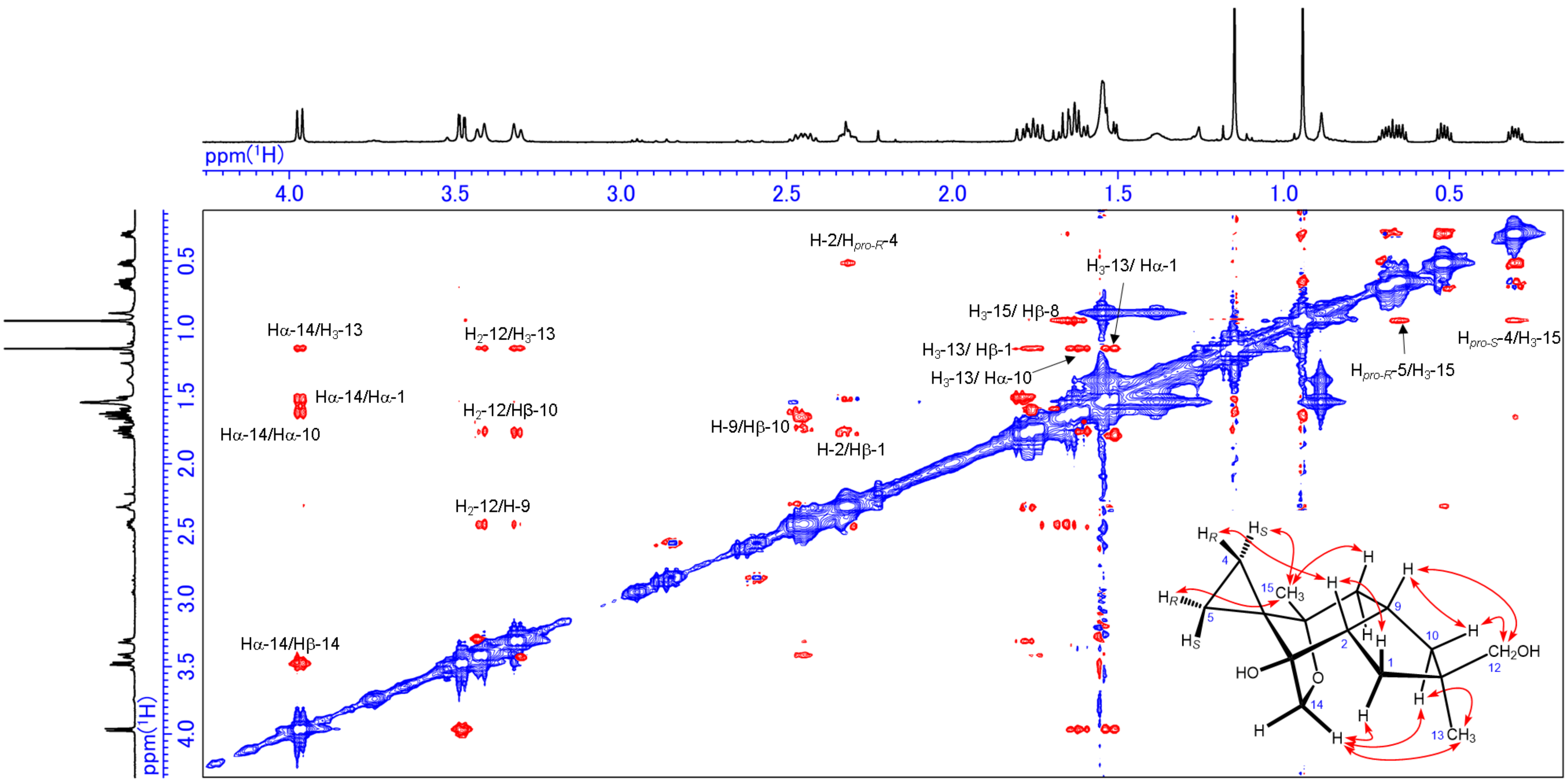

IR spectrum of 12-*O*-acetate of **6** (film)

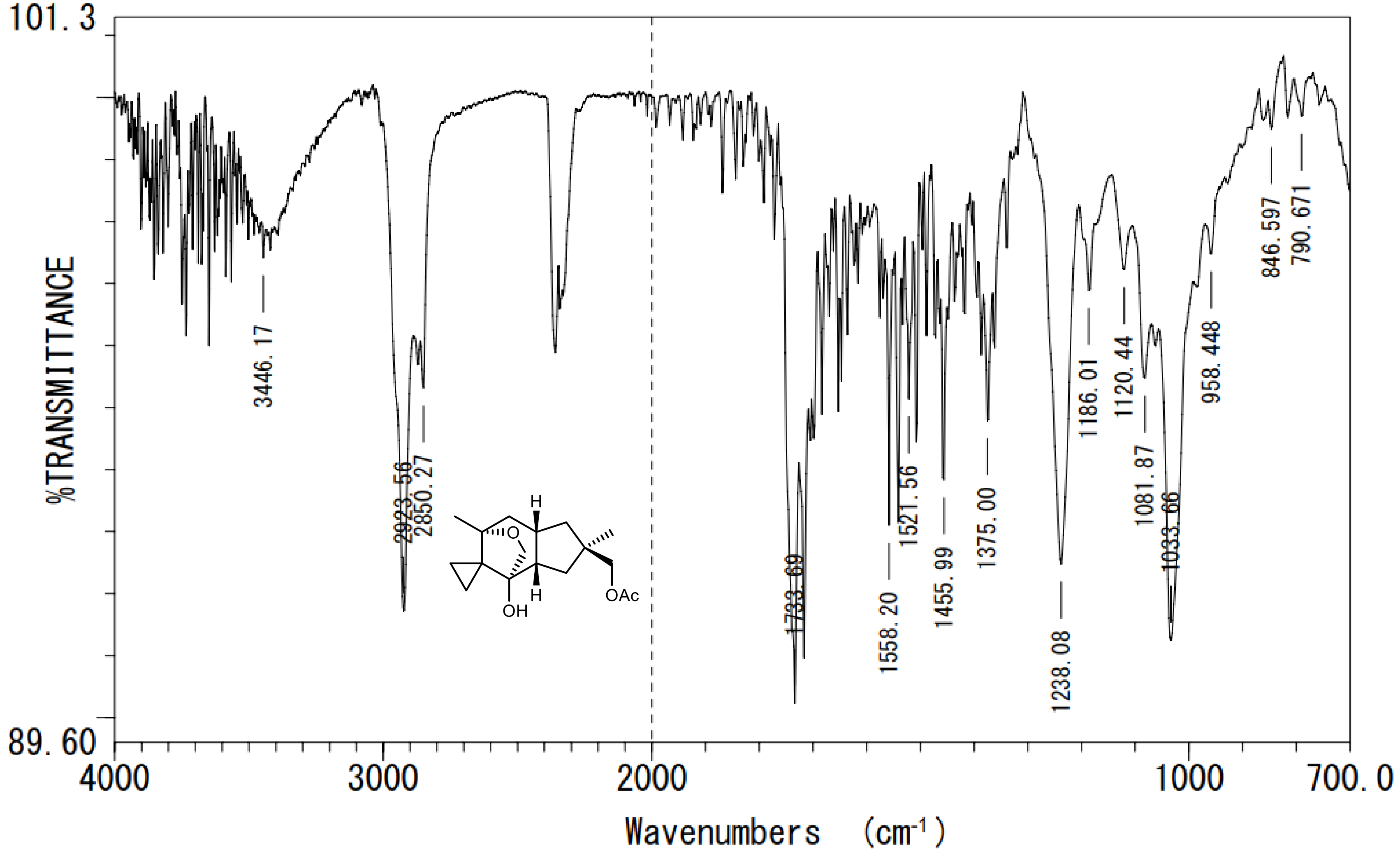

ESI-TOFMS spectrum of 12-*O*-acetate of **6**.

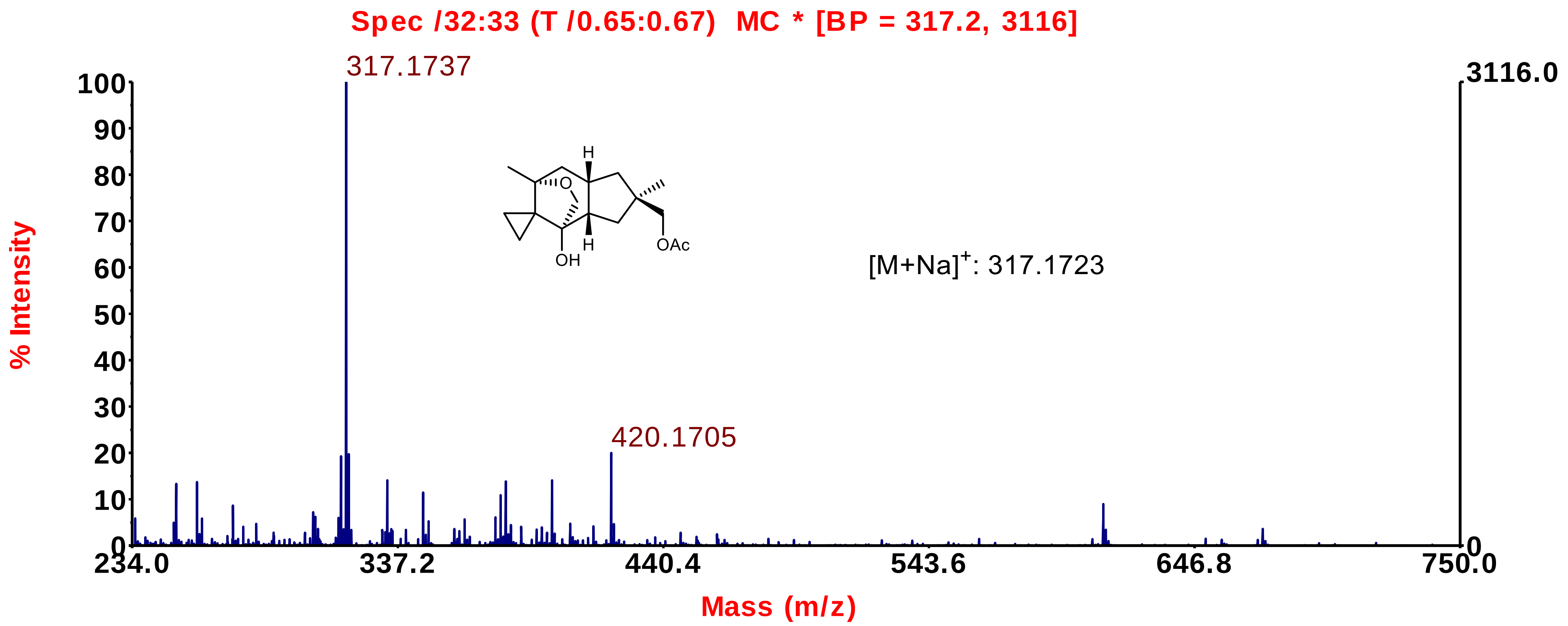

^{1}H NMR spectrum of 12-*O*-acetate of **6** (500 MHz, $CDCl_3$)

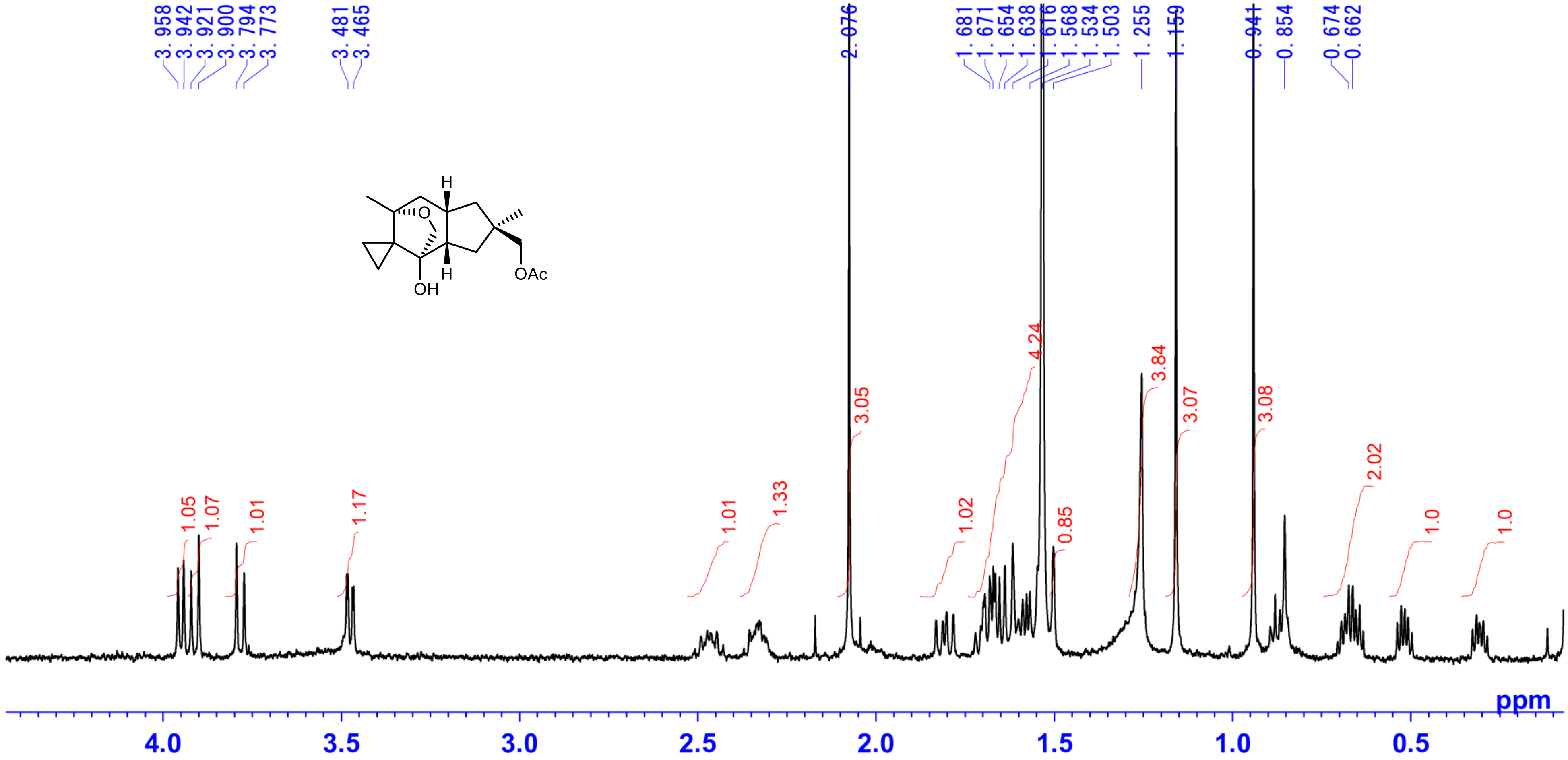

^{13}C NMR spectrum of 12-*O*-acetate of **6** (125 MHz, $CDCl_3$)

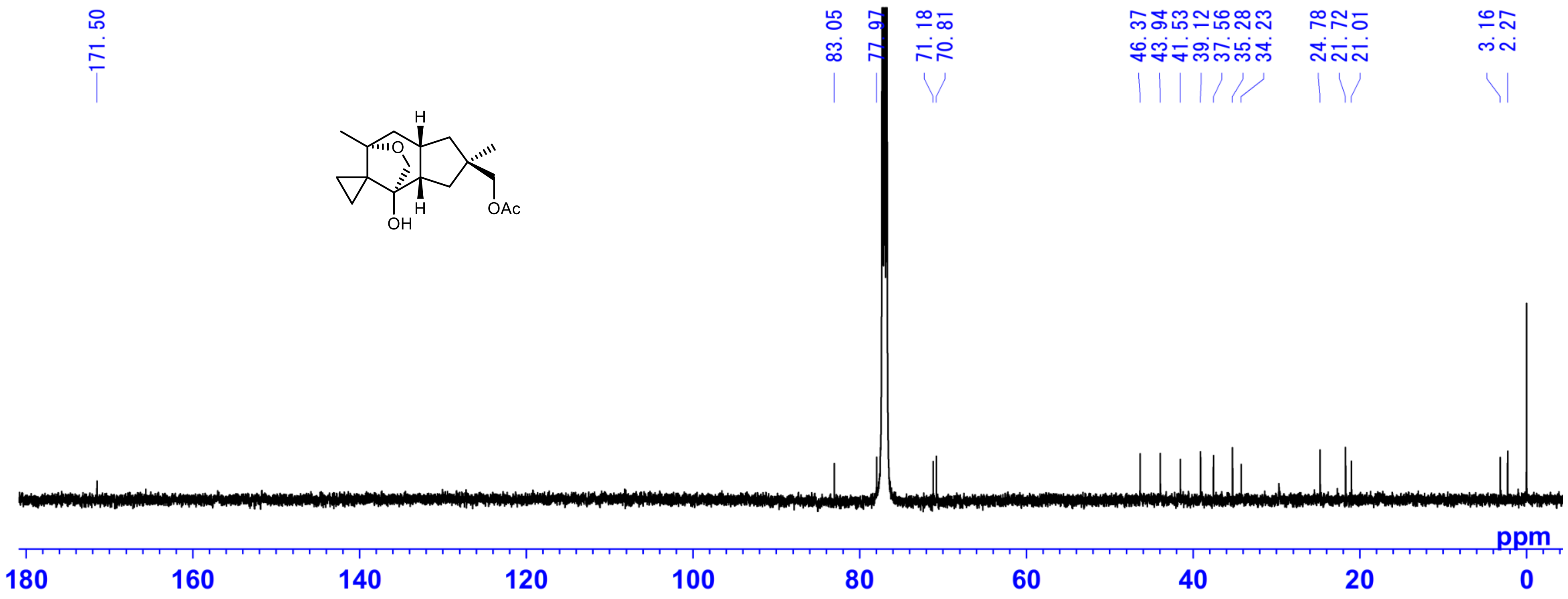

DQF COSY spectrum of 12-*O*-acetate of **6** (500 MHz, $CDCl_3$)

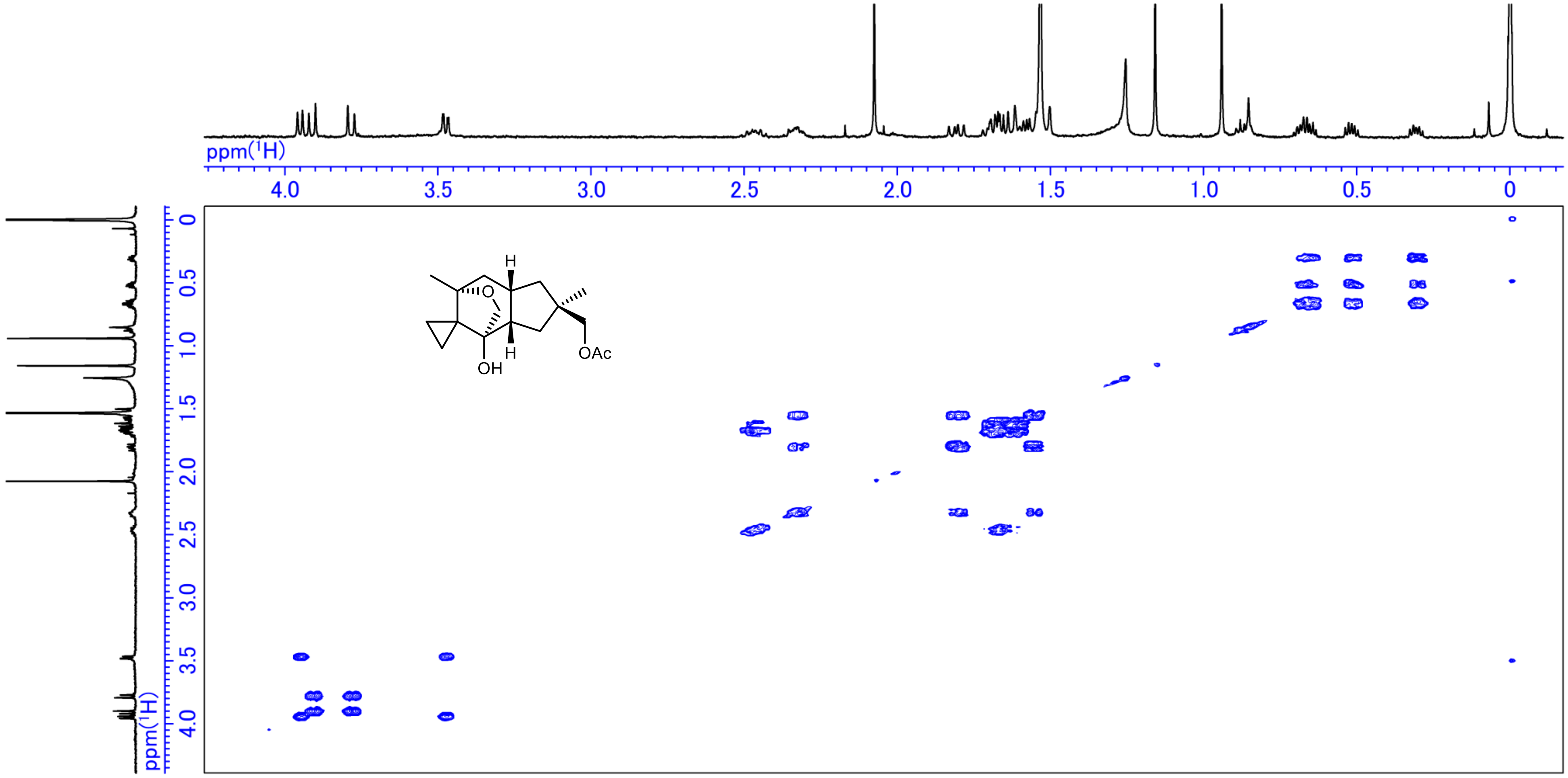

IR spectrum of **7** (film)

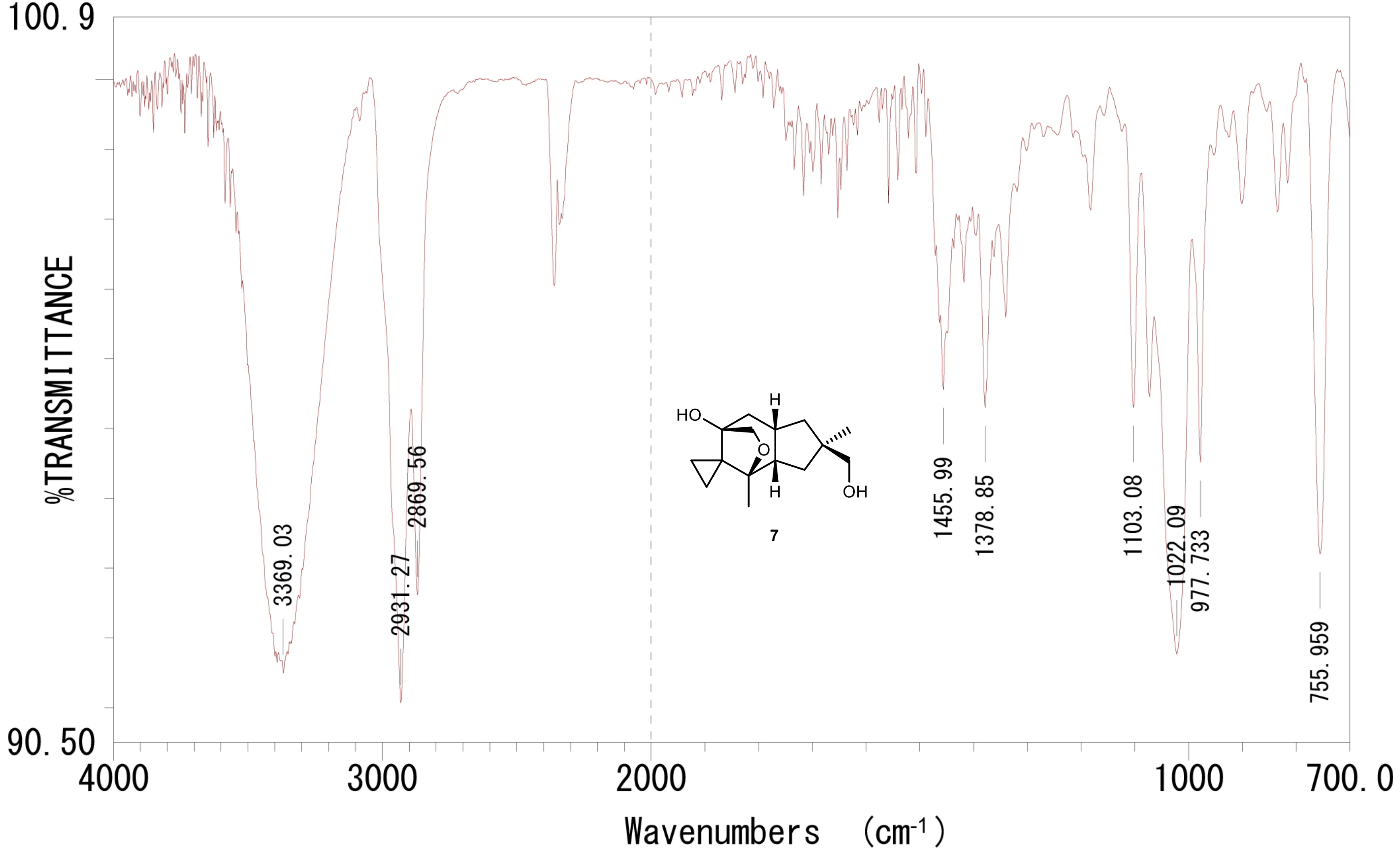

ESI-TOFMS spectrum of **7**

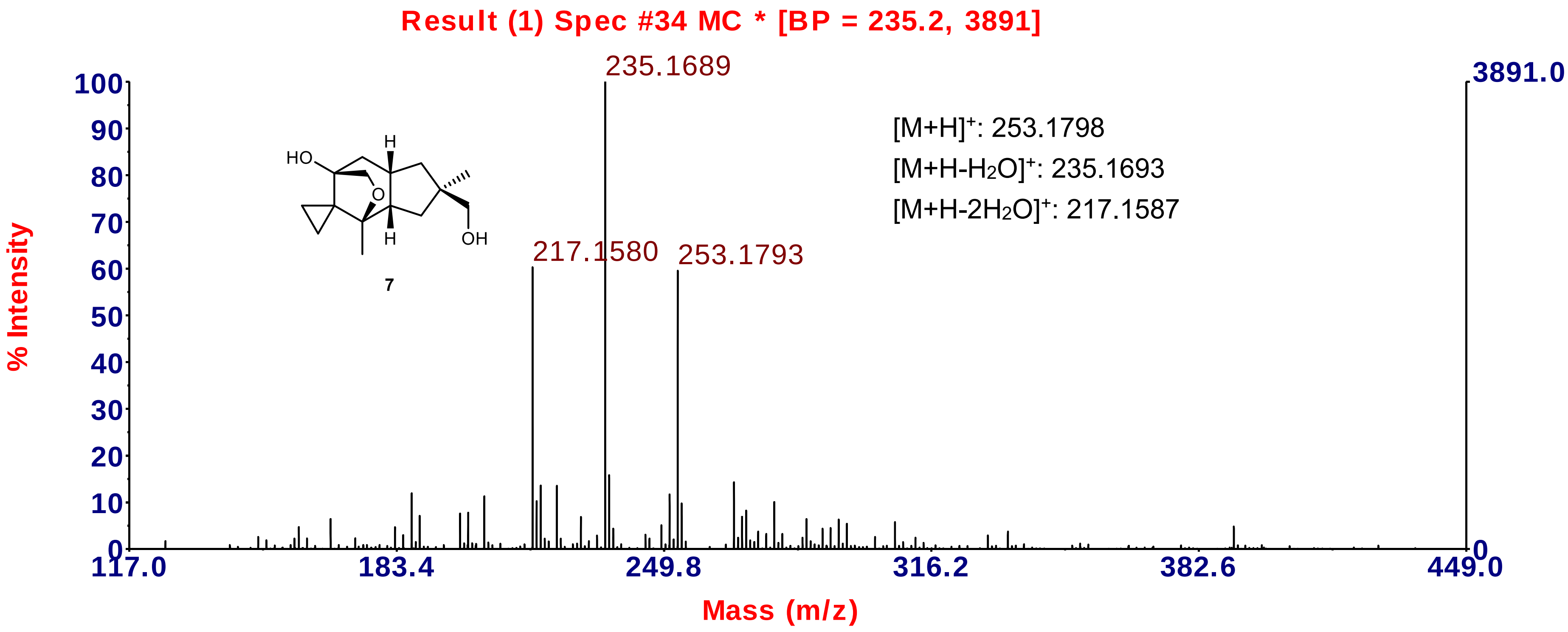

^{1}H NMR spectrum of **7** (500 MHz, $CDCl_3$)

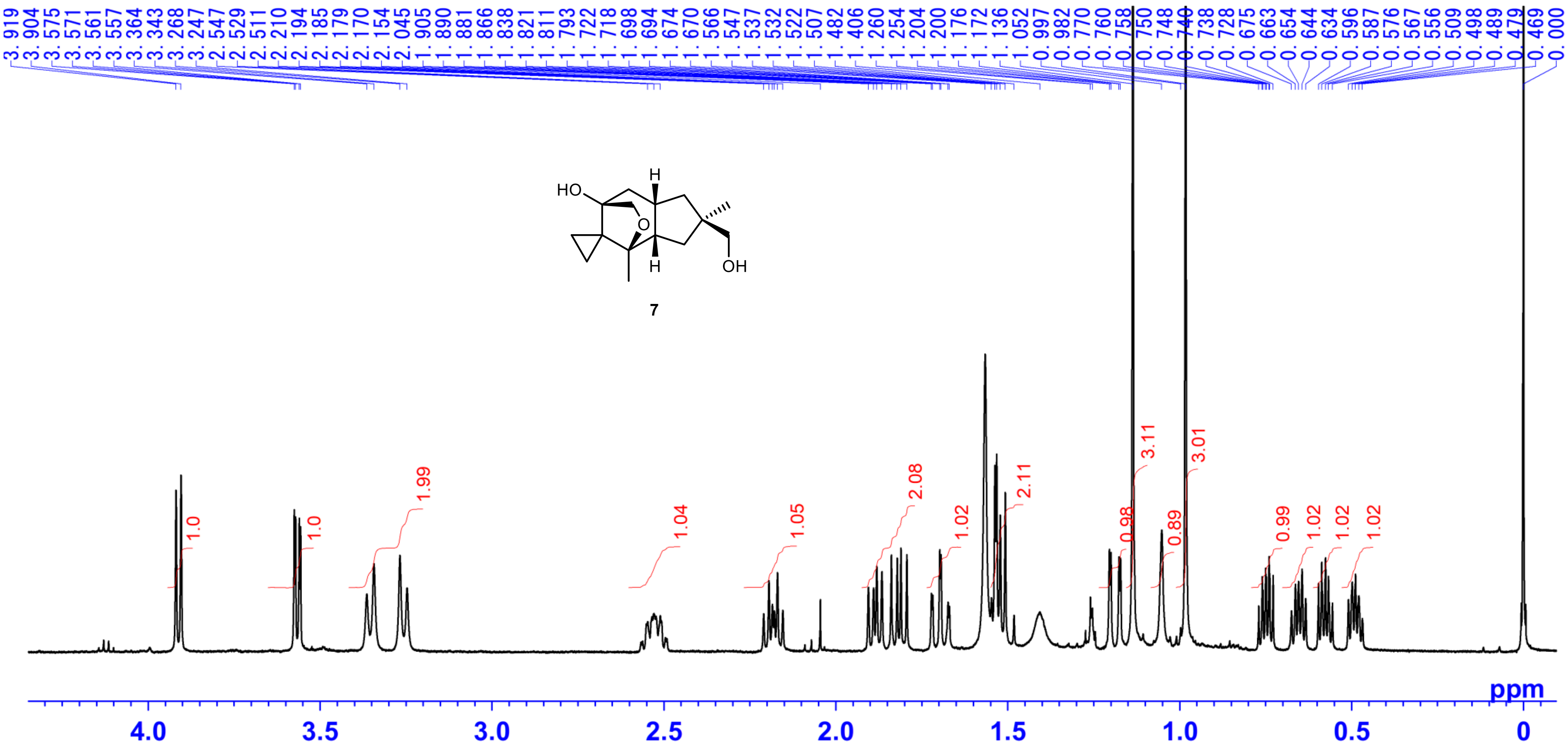

^{13}C NMR spectrum of **7** (125 MHz, $CDCl_3$)

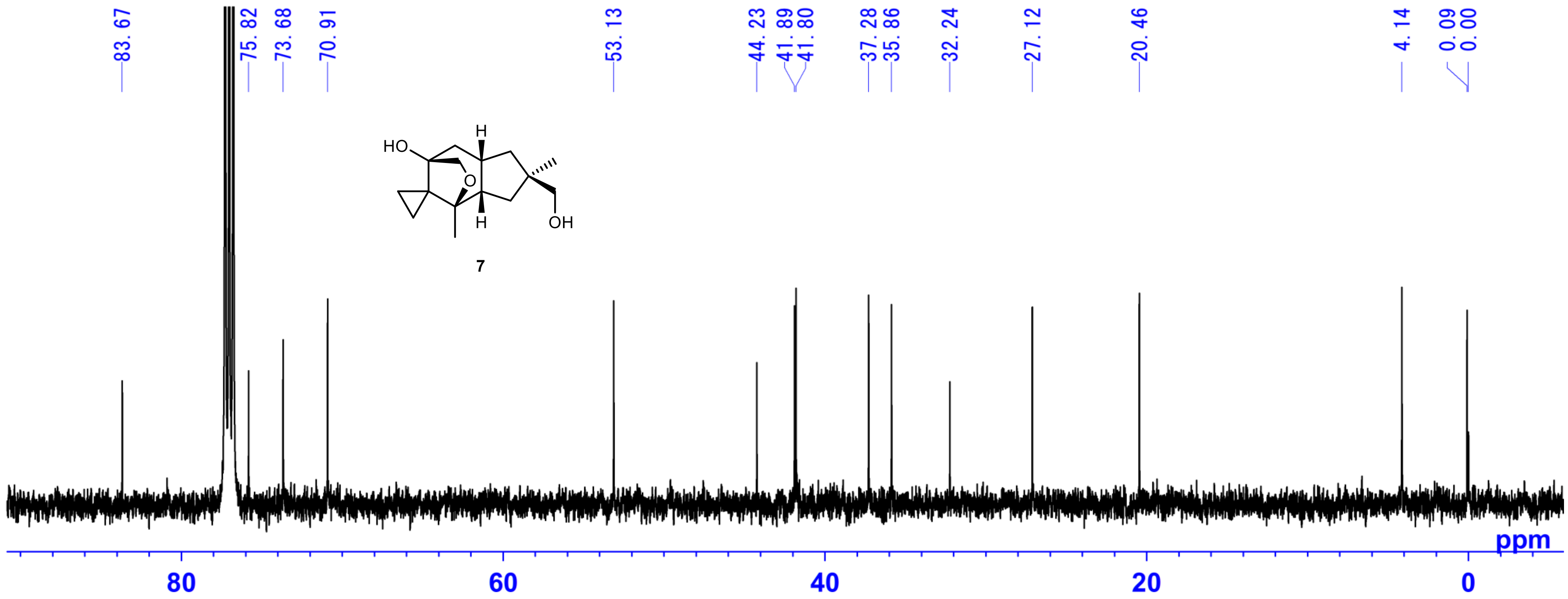

DQF COSY spectrum of **7** (500 MHz, $CDCl_3$)

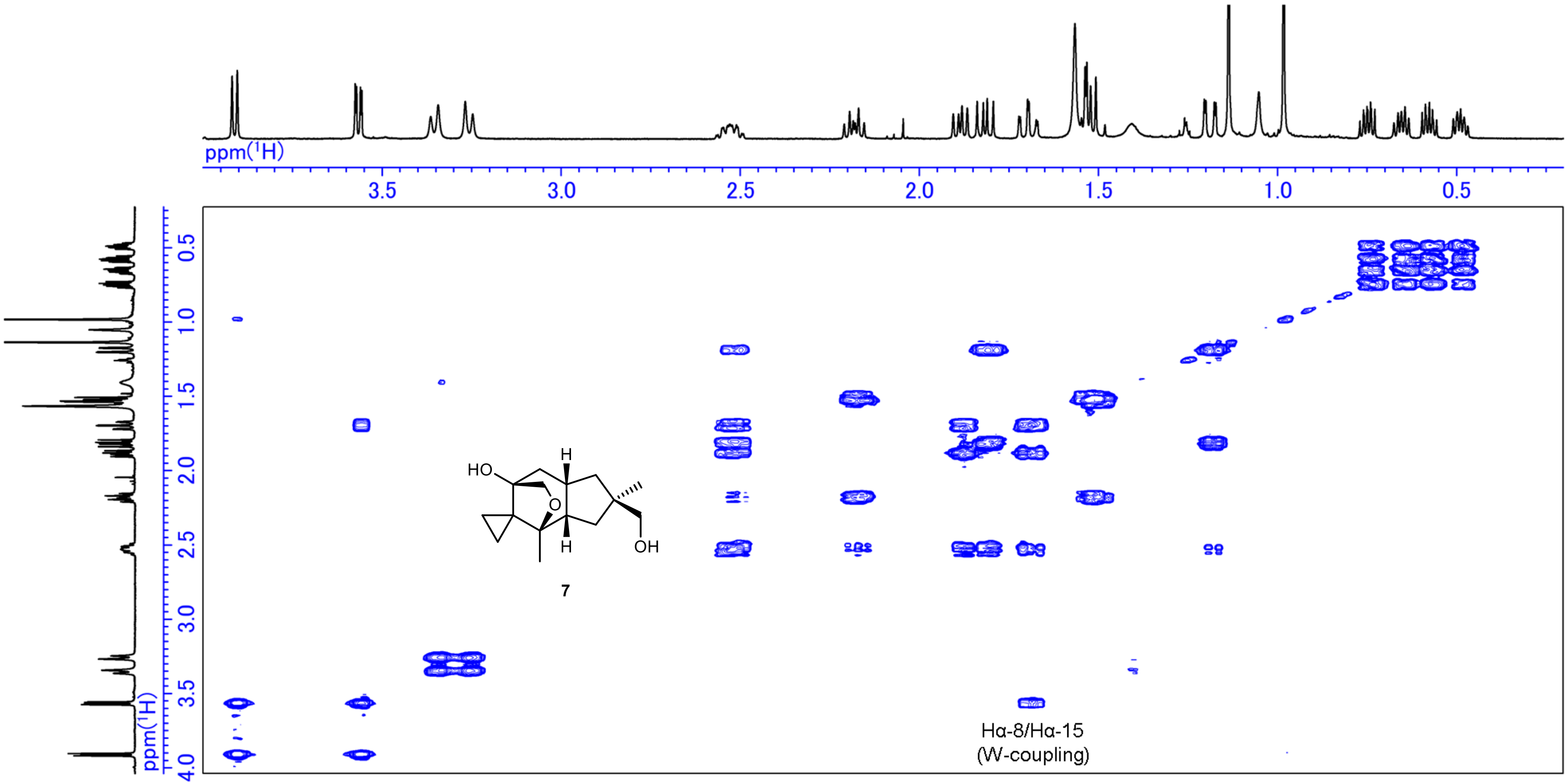

HSQC spectrum of **7** (500 MHz, $CDCl_3$)

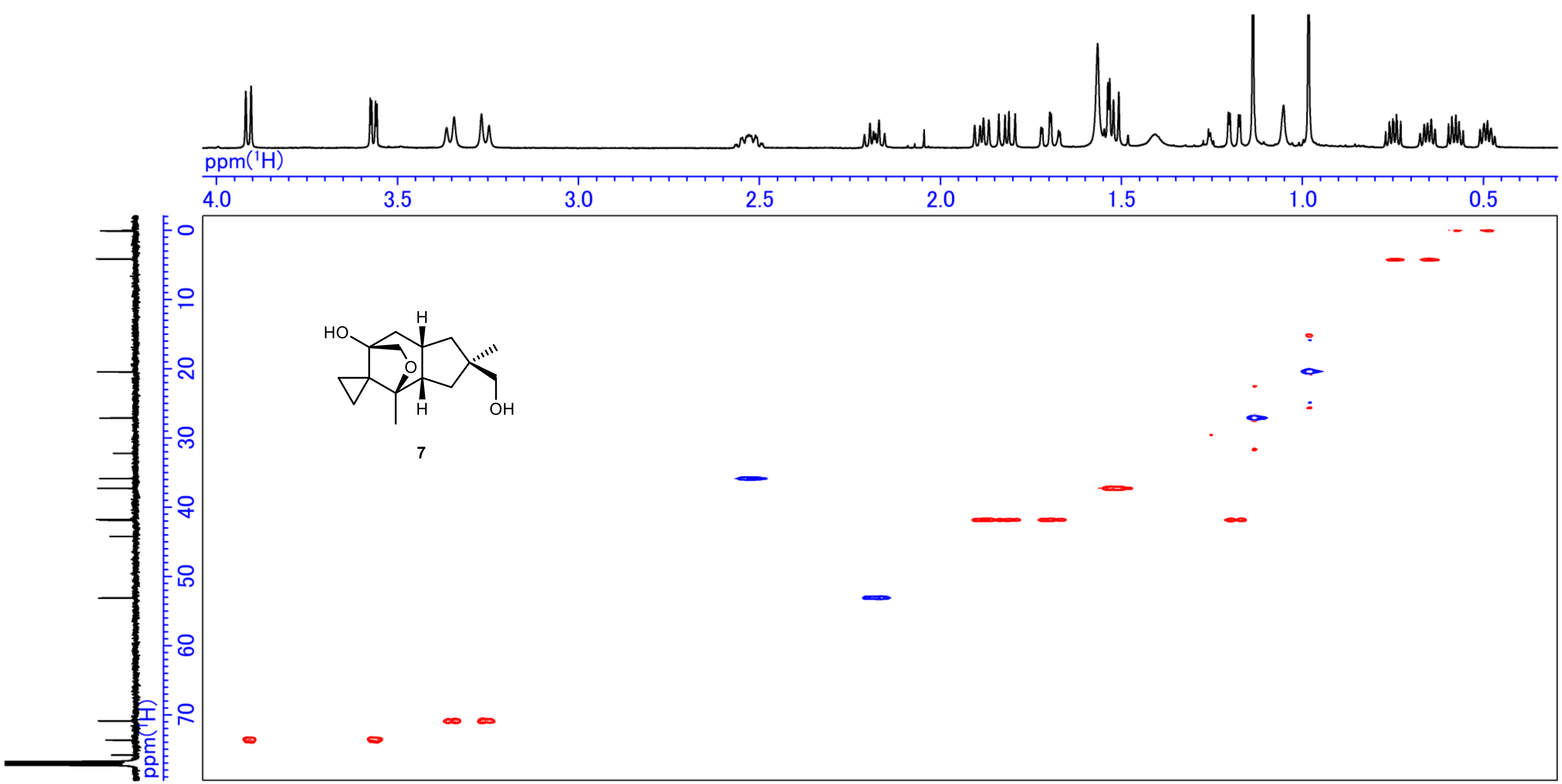

HMBC spectrum of **7** (500 MHz, $CDCl_3$)

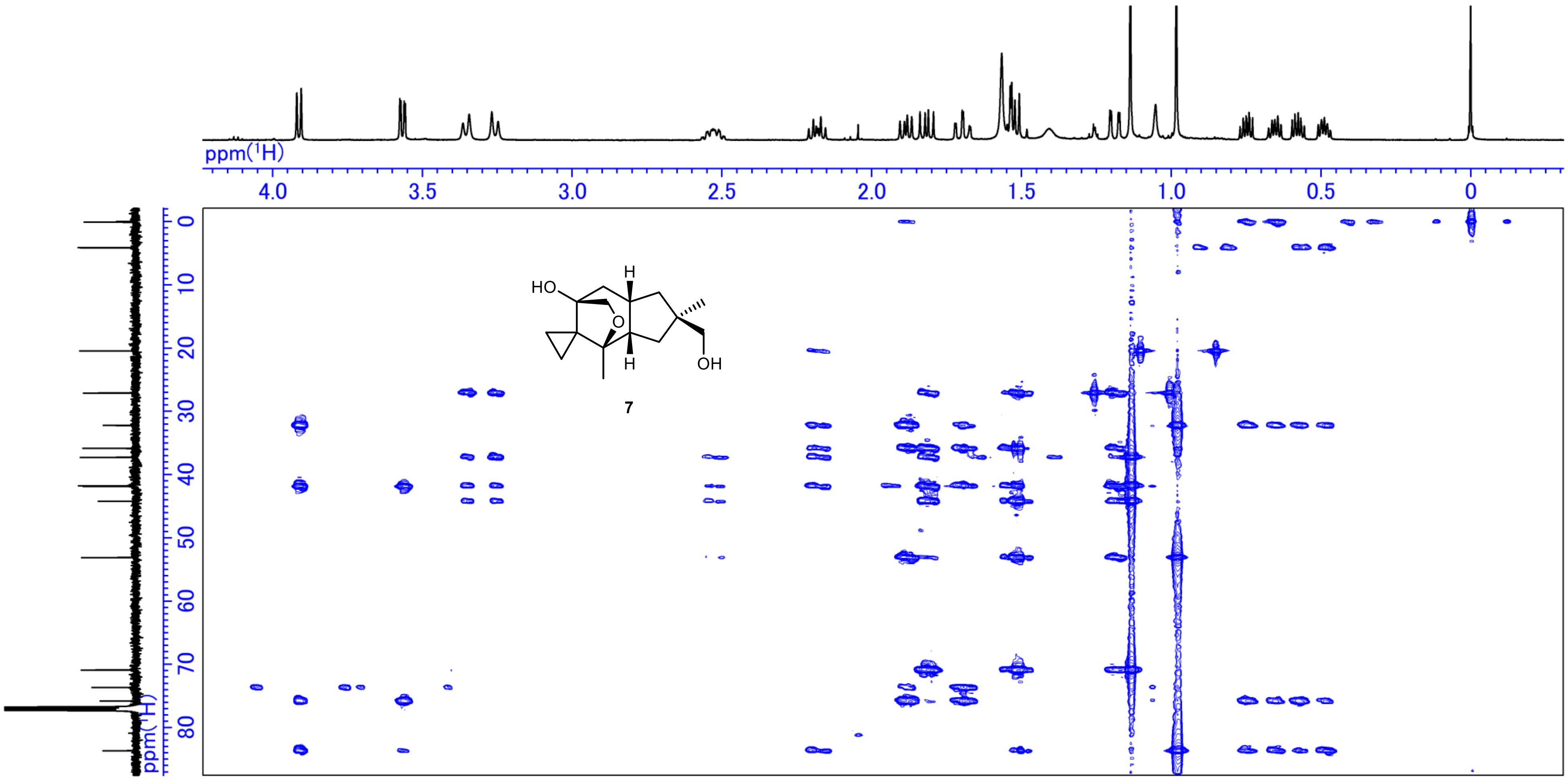

NOESY spectrum of **7** (500 MHz, $CDCl_3$)

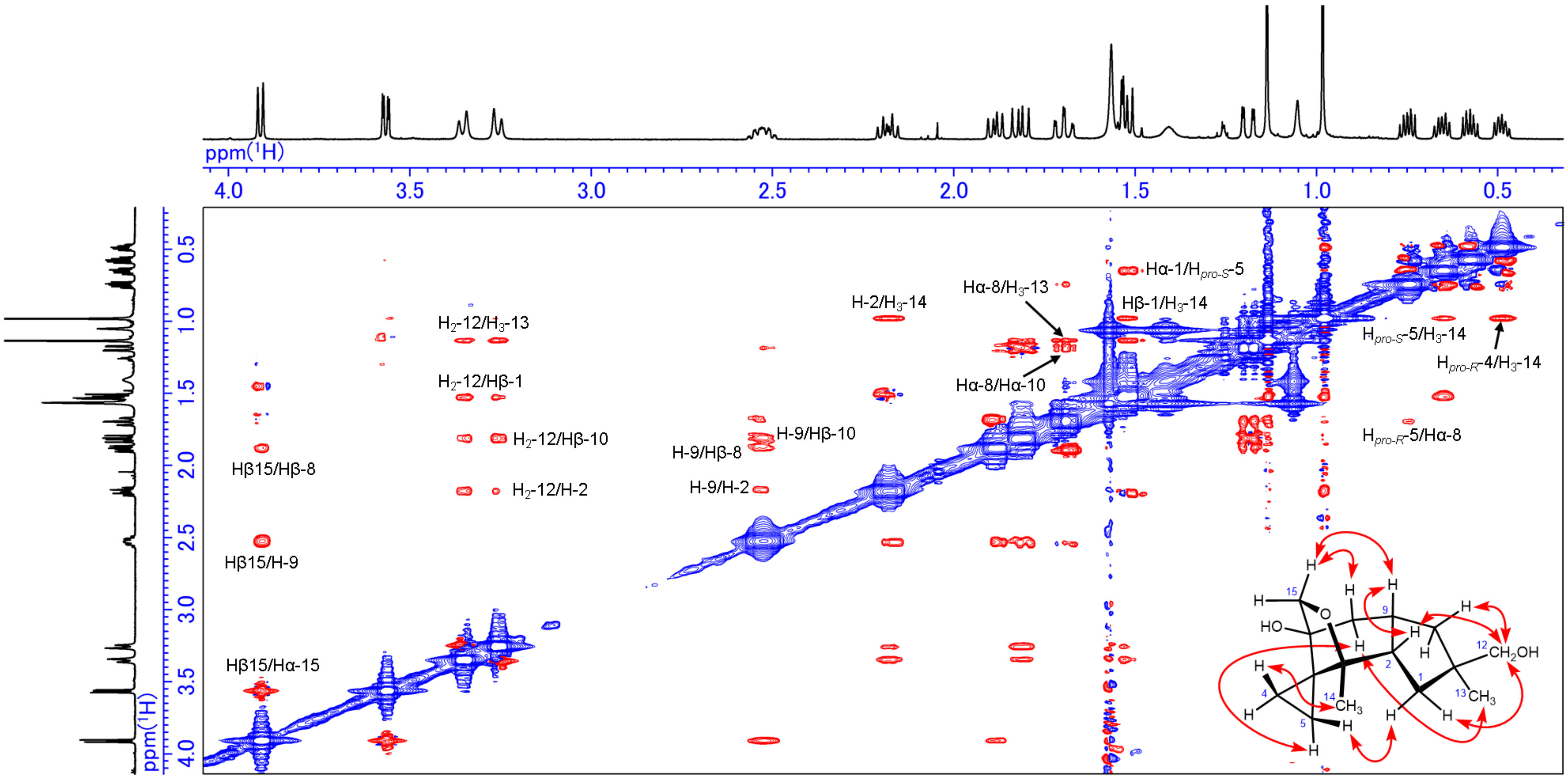

^{1}H NMR spectrum of **8** (500 MHz, $CDCl_3$)

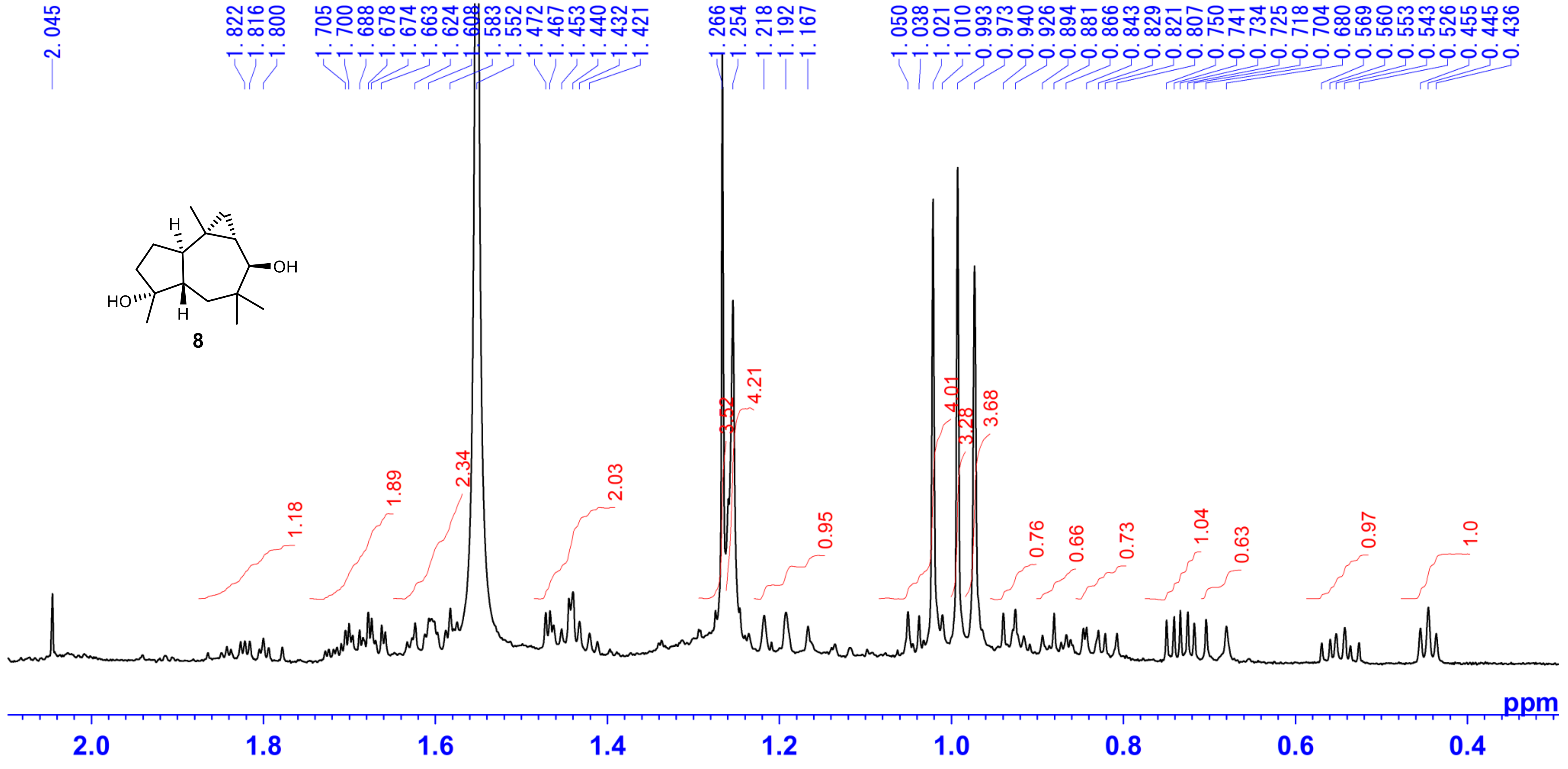

^{13}C NMR spectrum of **8** (125 MHz, $CDCl_3$)

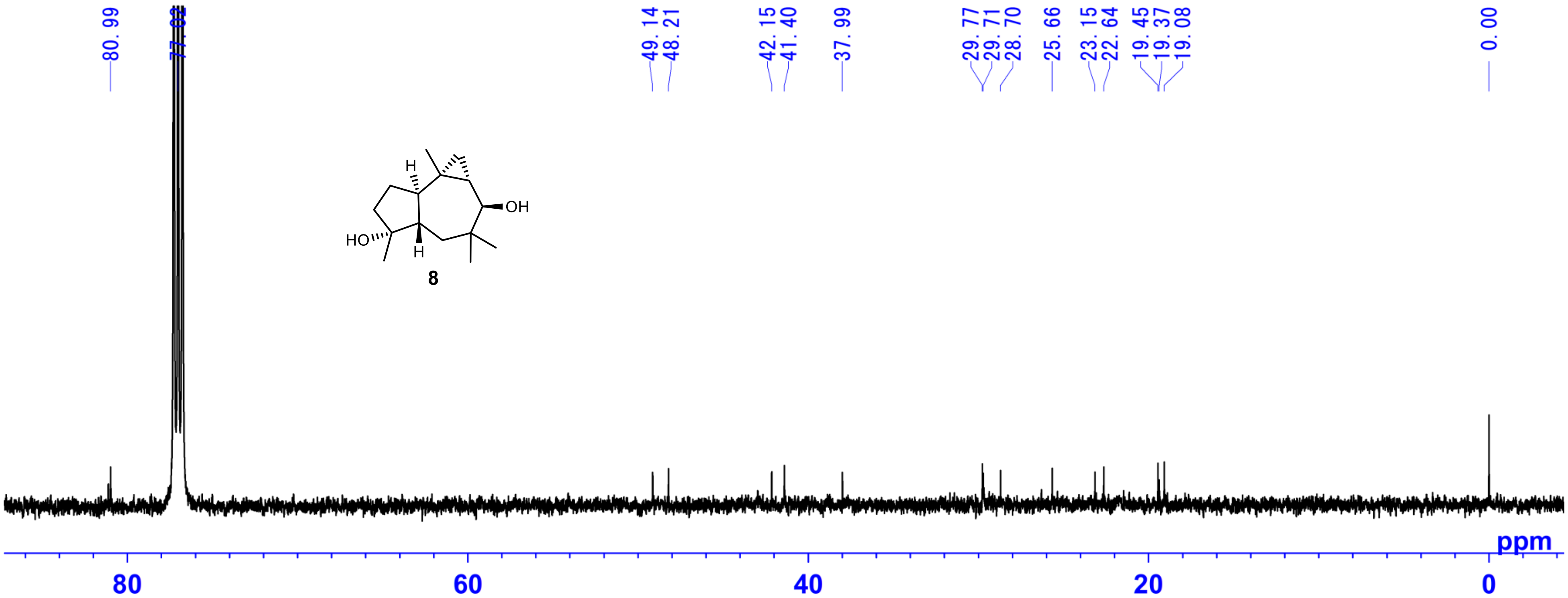

NOESY spectrum of **8** (500 MHz, $CDCl_3$).

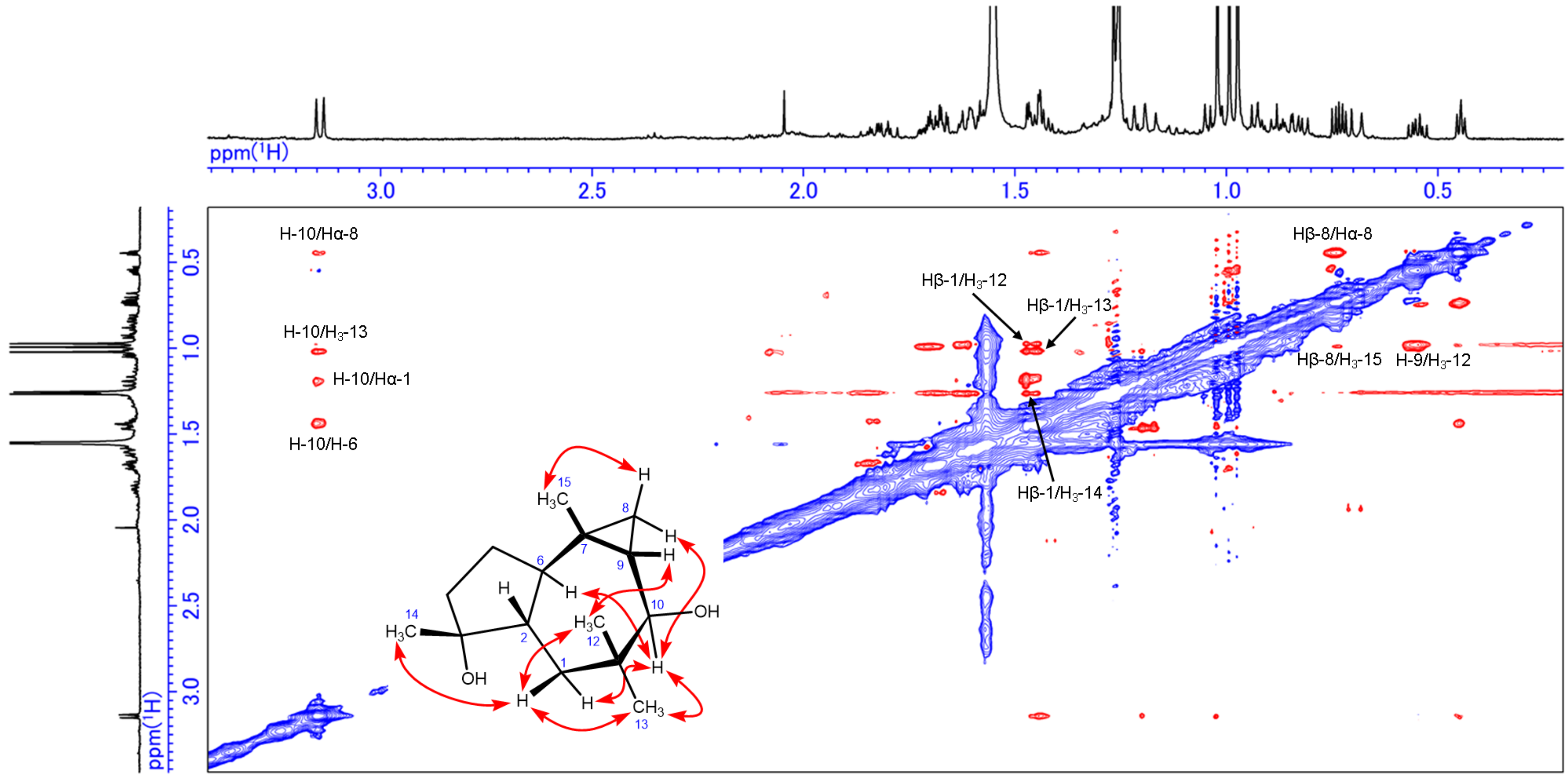

IR spectrum of **8-OBz** (film)

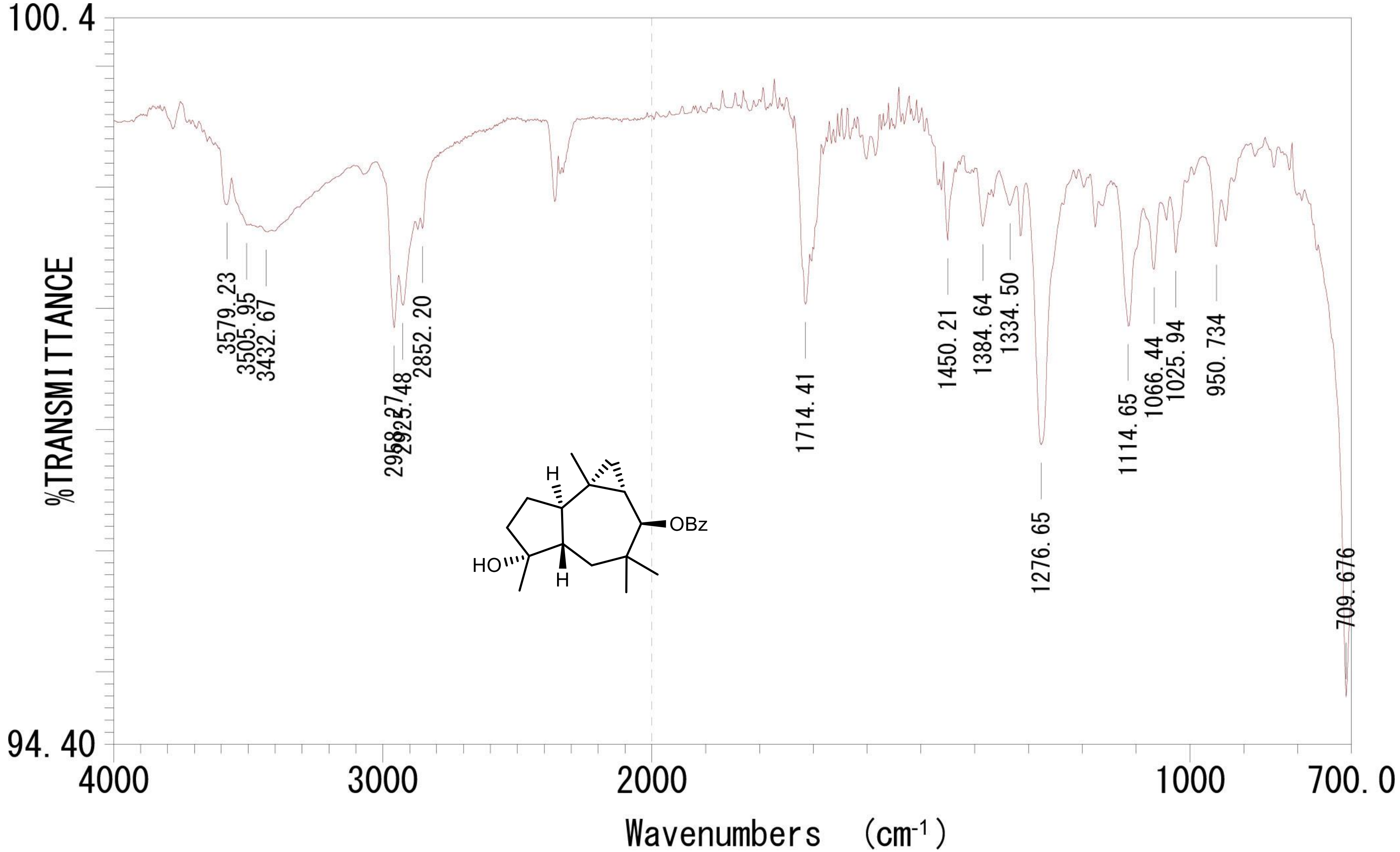

ESI-TOFMS spectrum of **8-OBz**

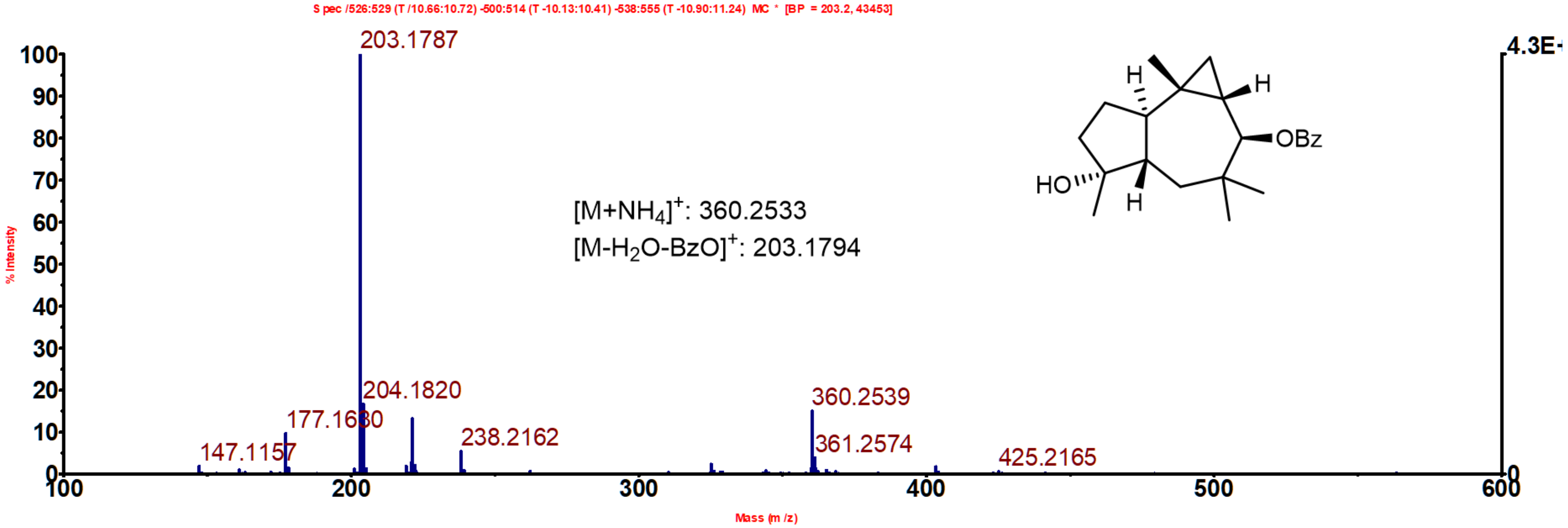

^{1}H NMR spectrum of **8-OBz** (500 MHz, $CDCl_3$)

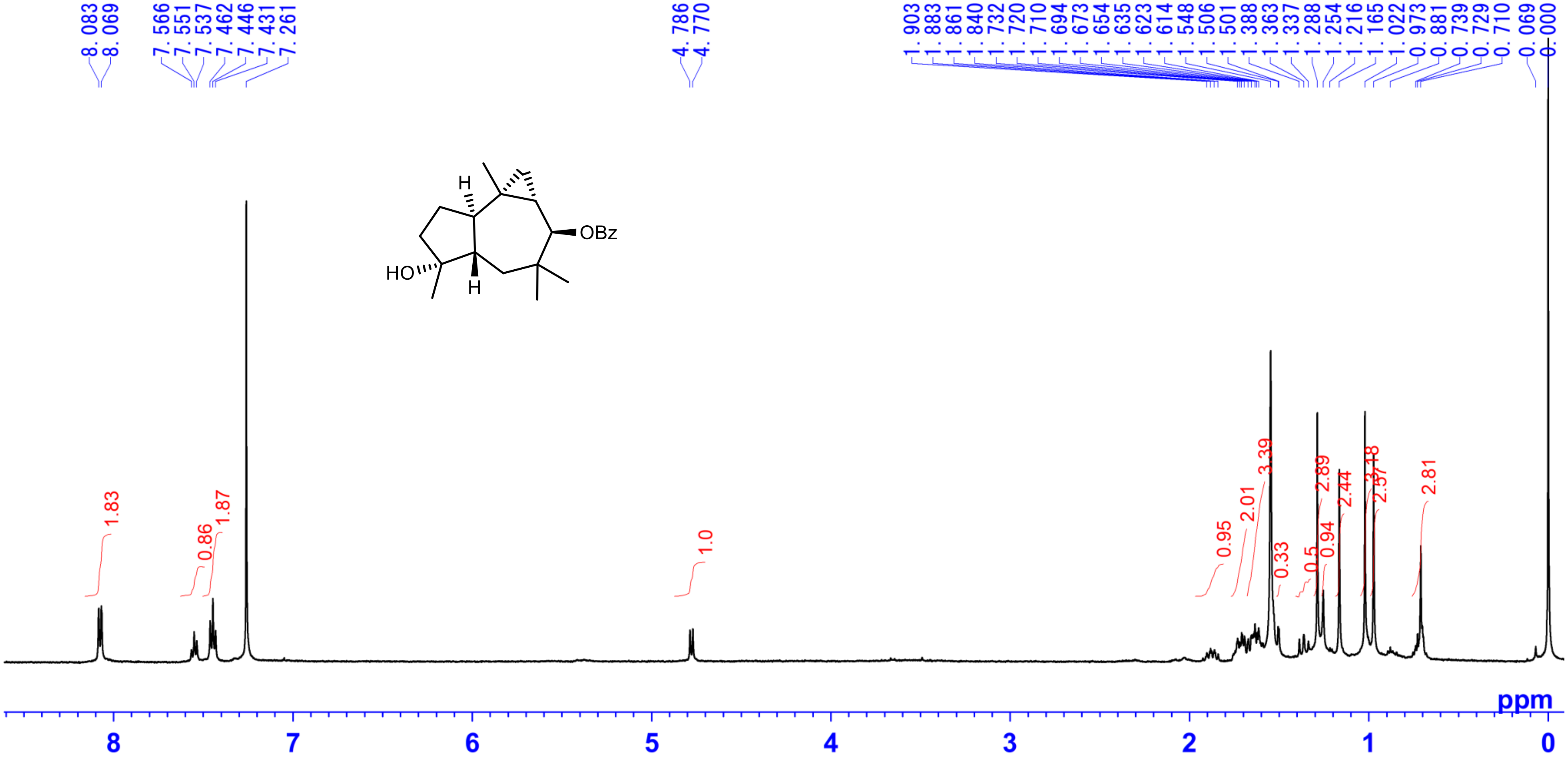

^{13}C NMR spectrum of **8-OBz** (125 MHz, $CDCl_3$)

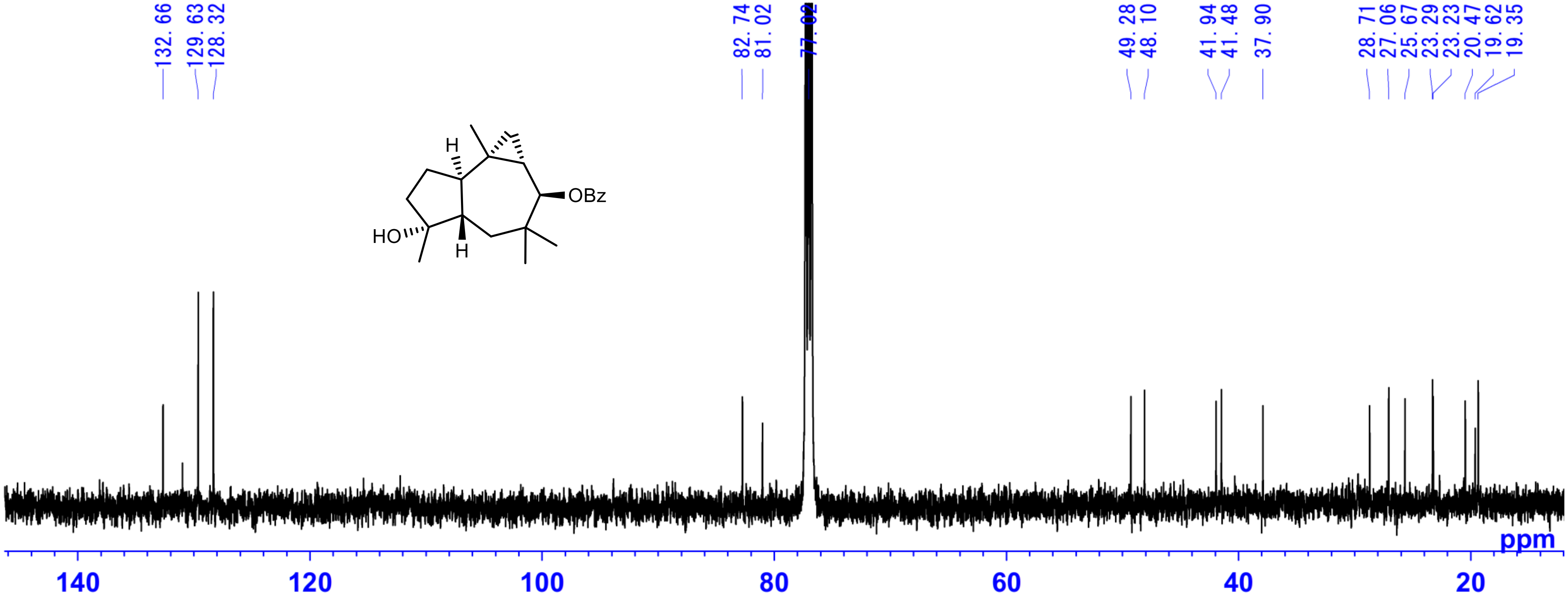

HSQC spectrum of **8-OBz** (500 MHz, $CDCl_3$)

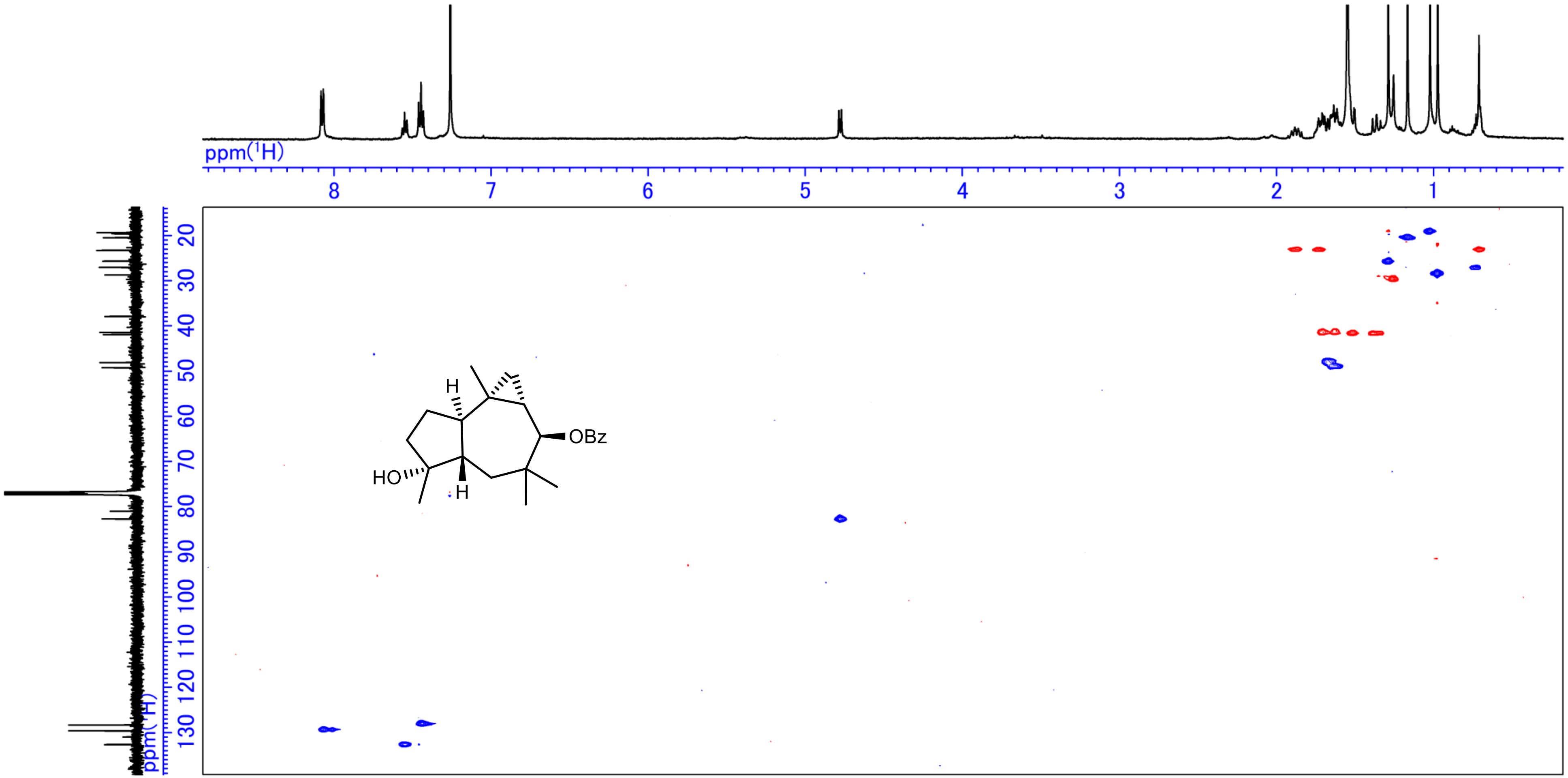

HMBC spectrum of **8-OBz** (500 MHz, $CDCl_3$)

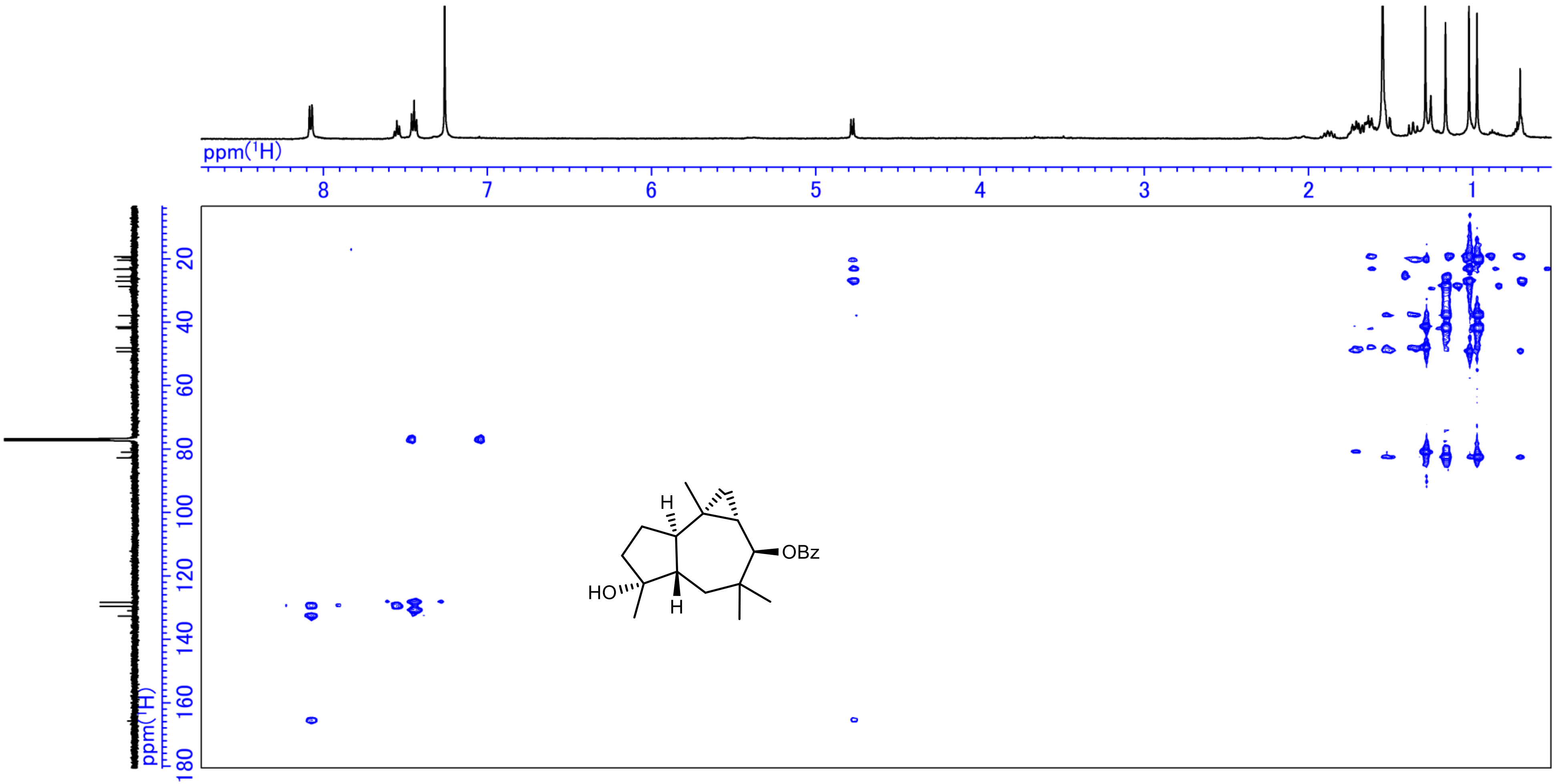

Chemical shift calculations.

- Calculations were performed with Spartan'18 Parallel Suite for Windows (Wavefunction Inc.) was used with default setting.
- All calculation results were checked manually. If abnormal conformers were found, these were recalculated after updating the initial conformation.
- Boltzmann distributions were obtained based on the SCF energy using ωB97X-V/6-311+G(2df,2p)[6-311G*]//ωB97X-D/6-31G*
- Solvent effect was not considered in all calculations.
- The original parameters by Goodman (^{13}C: σ = 2.306 ppm, ν = 11.38, ^{1}H: σ = 0.185 ppm, ν = 14.18) by Goodman (*J. Am. Chem. Soc*. 2010, 132, 12946) was used for DP4 analysis, although the present protocol was proved to give much accurate ^{13}C chemical shifts.
- Although DP4 analysis requires empirical correction for both δ^{13}C and δ^{1}H, it was skipped because the protocol involves similar correction.
- For proposed diastereomers, prochiral methylene ^{1}H signals were strictly arranged based on NOEs except for H_2-5 and H_2-6 of **8.** Their orientation could not be elucidated by NOE experiments. Thus, these were arranged to become the chemical shift differences from the calculated values smaller).
- Prochiral methylene protons in other diastereomers were arranged to become the chemical shift differences from the calculated values smaller.
- Details such as conformation search, chemical shift of individual conformers, conformational distributions, obtained geometries (SDF format) are seen in the associated Excel files

Calculated ^{13}C NMR chemical shift of **1a** and its isomers.

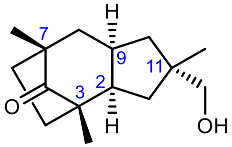

(2*R*,3*S*,7*R*,9*R*,11*S*)
the mirror image of natural product

	C-1	C-2	C-3	C-4	C-5	C-6	C-7	C-8	C-9	C-10	C-11	C-12	C-13	C-14	C-15
experimental	36.7	59.3	48.4	32.3	29.0	222.4	46.2	48.6	35.5	40.6	43.8	71.9	27.5	18.4	19.8
repraesentin B[a]	36.8	58.6	48.5	32.5	29.0	222.3	46.2	48.2	35.2	40.6	44.4	72.7	26.7	18.5	19.7
**(2*R*,3*S*,7*R*,9*R*,11*S*)**	37.2	58.5	48.5	32.5	29.2	221.4	46.0	47.3	36.3	39.9	43.5	73.2	27.5	19.8	20.7
(2*R*,3*S*,7*R*,9*R*,11*R*)	35.9	57.5	48.2	32.4	29.2	221.5	46.0	46.0	35.9	40.2	44.2	72.1	27.1	19.8	20.6
(2*R*,3*S*,7*R*,9*S*,11*R*)	38.5	54.2	47.1	34.6	35.3	220.9	41.6	47.2	38.7	41.6	42.9	72.4	26.5	16.5	22.2
(2*R*,3*S*,7*R*,9*S*,11*S*)	39.4	55.0	47.2	34.5	35.3	221.2	41.7	47.2	37.8	40.6	42.8	72.6	26.4	16.5	22.2
(2*S*,3*S*,7*R*,9*R*,11*R*)	36.2	55.7	48.7	25.9	31.0	221.4	45.4	43.0	37.4	39.6	43.2	72.4	26.5	18.4	20.9
(2*S*,3*S*,7*R*,9*R*,11*S*)	35.0	55.1	48.8	26.0	31.0	221.7	45.5	43.1	38.5	40.8	43.0	72.8	26.4	18.5	21.0
(2*S*,3*S*,7*R*,9*S*,11*S*)	38.0	53.0	50.6	25.0	30.4	223.2	45.5	43.6	34.7	41.6	43.4	70.9	25.2	20.4	21.8
(2*S*,3*S*,7*R*,9*S*,11*R*)	39.3	54.1	50.2	26.5	30.3	223.0	45.4	43.5	34.7	42.9	43.4	71.1	24.6	20.2	21.6

Calculated ^{1}H NMR chemical shift of **1a** and its isomers.

	Hβ-1	Hα-1	H-2	Hβ-4	Hα-4	Hβ-5	Hα-5	Hα-8	Hβ-8	H-9	Hβ-10	Hα-10	H-12	H-12	H-13	H-14	H-15
experimental	1.67	1.05	2.27	1.98	1.63	1.95	1.58	1.64	1.74	2.52	1.75	1.11	3.24	3.29	1.06	0.95	0.96
repraesentin B[a]	1.32	1.19	2.37	2.01	1.64	1.95	1.59	1.58	1.7	2.55	1.54	1.33	3.34	3.34	0.99	0.97	0.94
**(2*R*,3*S*,7*R*,9*R*,11*S*)**	1.68	1.37	2.33	1.93	1.61	1.90	1.58	1.64	1.74	2.40	1.97	1.18	3.30	3.30	1.00	0.96	0.95
(2*R*,3*S*,7*R*,9*R*,11*R*)	1.50	1.30	2.33	1.94	1.63	1.89	1.58	1.50	1.91	2.40	1.52	1.51	3.33	3.35	0.97	0.96	0.93
(2*R*,3*S*,7*R*,9*S*,11*R*)	1.59	1.42	2.23	1.78	1.31	1.95	1.84	1.48	1.83	1.60	1.79	0.90	3.34	3.36	1.01	0.99	1.03
(2*R*,3*S*,7*R*,9*S*,11*S*)	1.80	1.29	2.30	1.77	1.29	1.97	1.83	1.52	1.84	1.63	1.40	1.32	3.39	3.36	0.97	0.98	1.03
(2*S*,3*S*,7*R*,9*R*,11*R*)	1.59	1.14	1.69	1.92	1.39	1.79	1.58	1.61	1.35	2.04	1.39	1.31	3.39	3.37	1.07	0.95	0.97
(2*S*,3*S*,7*R*,9*R*,11*S*)	1.69	1.45	2.30	2.06	1.45	1.97	1.68	1.79	1.88	2.24	1.86	1.81	3.44	3.28	1.11	0.89	0.96
(2*S*,3*S*,7*R*,9*S*,11*S*)	2.02	1.50	2.35	2.57	1.31	1.95	1.64	1.81	1.91	2.28	2.40	1.20	3.53	3.53	0.93	0.90	0.96
(2*S*,3*S*,7*R*,9*S*,11*R*)	1.77	1.45	2.30	2.07	1.46	1.97	1.68	1.79	1.88	2.25	1.83	1.80	3.44	3.31	1.13	0.89	0.96

a: Hirota, M.; Shimizu, Y.; Kamo, T.; Makabe, H.; Shibata, H. *Bioscience, Biotechnology, and Biochemistry* **2003,** *67*, 1597-1600.

Statistical analysis of δ^{13}C for **1a** and its isomers.

	vs experimental			repraesentin B[b]			
	RMSD	max deviation	DP4	RMSD	max deviation	DP4	
**(2*R*,3*S*,7*R*,9*R*,11*S*)**	**0.8**	**1.4**	**66.7%**	**0.7**	**1.3**	**64.3%**	proposed diastereomer
(2*R*,3*S*,7*R*,9*R*,11*R*)	1.0	2.6	33.3%	0.9	2.2	35.7%	the 11-epimer
(2*R*,3*S*,7*R*,9*S*,11*R*)	2.9	6.3	0.0%	2.9	6.3	0.0%	
(2*R*,3*S*,7*R*,9*S*,11*S*)	2.8	6.3	0.0%	2.8	6.3	0.0%	
(2*S*,3*S*,7*R*,9*R*,11*R*)	2.6	6.4	0.0%	2.6	6.6	0.0%	
(2*S*,3*S*,7*R*,9*R*,11*S*)	2.7	6.3	0.0%	2.7	6.5	0.0%	
(2*S*,3*S*,7*R*,9*S*,11*S*)	3.1	7.4	0.0%	3.1	7.5	0.0%	
(2*S*,3*S*,7*R*,9*S*,11*R*)	2.9	5.9	0.0%	2.8	6.0	0.0%	

Statistical analysis of δ^{1}H for **1a** and its isomers.

	vs experimental			vs. repraesentin B			
	RMSD	max deviation	DP4	RMSD	max deviation	DP4	
**(2*R*,3*S*,7*R*,9*R*,11*S*)**	**0.10**	**0.32**	**99.5%**	**0.16**	**0.70**	**1.5%**	proposed diastereomer
(2*R*,3*S*,7*R*,9*R*,11*R*)	0.15	0.40	0.5%	0.10	0.70	98.5%	the 11-epimer
(2*R*,3*S*,7*R*,9*S*,11*R*)	0.28	0.91	0.0%	0.30	0.71	0.0%	
(2*R*,3*S*,7*R*,9*S*,11*S*)	0.28	0.88	0.0%	0.29	0.70	0.0%	
(2*S*,3*S*,7*R*,9*R*,11*R*)	0.24	0.58	0.0%	0.25	0.69	0.0%	
(2*S*,3*S*,7*R*,9*R*,11*S*)	0.23	0.70	0.0%	0.22	0.68	0.0%	
(2*S*,3*S*,7*R*,9*S*,11*S*)	0.30	0.65	0.0%	0.34	0.71	0.0%	
(2*S*,3*S*,7*R*,9*S*,11*R*)	0.23	0.69	0.0%	0.22	0.68	0.0%	

b: Hirota, M.; Shimizu, Y.; Kamo, T.; Makabe, H.; Shibata, H. *Bioscience, Biotechnology, and Biochemistry* **2003,** *67*, 1597-1600.

Calculated ^{13}C NMR chemical shift of **1b** and its isomers.

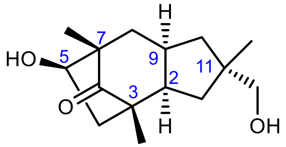

(2*R*,3*S*,5*R*,7*R*,9*R*,11*S*)
the mirror image of natural product

	C-1	C-2	C-3	C-4	C-5	C-6	C-7	C-8	C-9	C-10	C-11	C-12	C-13	C-14	C-15
	36.5	59.2	48.5	45.7	71.6	221.0	51.8	47.6	35.2	40.8	44.3	71.9	27.6	18.7	14.3
(2*R*,3*S*,5*R*,7*S*,9*R*,11*S*)	37.0	58.3	48.6	44.5	72.9	220.6	51.8	45.9	35.8	39.9	43.7	73.1	27.5	19.7	15.2
(2*R*,3*S*,5*R*,7*S*,9*R*,11*R*)	35.4	57.5	48.3	44.2	73.0	221.0	51.9	44.0	35.5	40.3	44.5	71.2	27.3	19.8	15.0
(2*R*,3*S*,5*R*,7*S*,9*S*,11*R*)	38.5	55.0	47.7	46.9	78.9	220.4	47.6	46.3	39.1	41.6	43.0	72.6	26.5	16.6	15.6
(2*R*,3*S*,5*R*,7*S*,9*S*,11*S*)	39.4	55.5	47.5	46.7	78.4	219.6	47.4	46.0	38.0	40.2	42.7	72.5	26.3	16.5	15.6
(2*R*,3*S*,5*S*,7*S*,9*R*,11*S*)	37.7	59.7	50.4	44.1	73.5	222.5	52.1	43.6	35.9	40.5	44.0	73.7	27.9	19.8	19.5
(2*S*,3*S*,5*R*,7*S*,9*R*,11*S*)	35.1	54.6	49.1	38.1	74.1	221.0	51.1	41.7	38.0	40.9	43.1	72.7	26.3	18.3	15.5
(2*S*,3*S*,5*R*,7*S*,9*S*,11*S*)	38.0	52.5	50.7	37.1	73.9	222.6	51.8	42.1	34.2	41.6	43.6	70.4	25.4	20.3	15.8
(2*S*,3*S*,5*R*,7*S*,9*R*,11*R*)	36.6	55.4	49.3	38.2	74.6	221.9	51.3	41.7	37.2	39.8	43.6	72.7	26.6	18.4	15.6
(2*S*,3*S*,5*R*,7*S*,9*S*,11*R*)	39.6	53.7	50.4	38.8	74.1	223.1	51.9	42.0	34.2	43.3	43.6	71.4	24.6	20.2	15.7
(2*R*,3*S*,5*S*,7*S*,9*R*,11*R*)	36.1	58.6	49.6	43.6	72.9	221.1	51.8	41.4	35.2	40.4	44.3	71.3	27.2	19.8	19.2
(2*R*,3*S*,5*S*,7*S*,9*S*,11*S*)	39.2	55.2	47.4	46.0	74.1	220.6	48.1	35.5	37.4	40.3	42.9	72.6	26.3	16.5	21.3
(2*R*,3*S*,5*S*,7*S*,9*S*,11*R*)	38.4	54.0	47.3	45.6	74.2	220.5	48.0	35.7	38.5	41.0	43.0	72.5	26.4	16.5	21.3
(2*S*,3*S*,5*S*,7*S*,9*R*,11*S*)	35.2	55.7	50.2	37.5	73.8	221.2	50.9	38.5	37.4	40.6	43.0	72.8	26.3	18.3	19.5
(2*S*,3*S*,5*S*,7*S*,9*S*,11*S*)	38.4	54.3	51.8	35.8	73.4	222.8	51.1	38.6	34.0	40.2	43.7	70.4	25.9	20.4	20.3
(2*S*,3*S*,5*S*,7*S*,9*S*,11*R*)	38.4	54.3	51.8	35.8	73.4	222.8	51.1	38.6	34.0	40.1	43.7	70.4	25.8	20.4	20.3
(2*S*,3*S*,5*S*,7*S*,9*R*,11*R*)	35.2	55.7	50.2	37.5	73.8	221.2	50.9	38.5	37.4	40.6	43.0	72.8	26.3	18.3	19.5

Calculated ^{1}H NMR chemical shift of **1b** and its isomers.

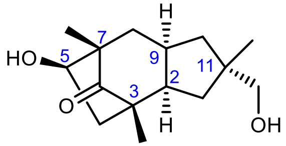

(2*R*,3*S*,5*R*,7*R*,9*R*,11*S*)
the mirror image of natural product

	Hα-1	Hβ-1	H-2	Hα-4	Hβ-4	H-5	Hα-8	Hβ-8	H-9	Hα-10	Hβ-10	H-12	H-12	H-13	H-14	H-15
	1.07	1.67	2.28	1.55	2.62	4.18	1.66	1.85	2.35	1.05	1.75	3.23	3.28	1.06	1.01	0.98
(2*R*,3*S*,5*R*,7*S*,9*R*,11*S*)	1.36	1.68	2.32	1.68	2.45	4.08	1.63	1.70	2.21	1.12	1.96	3.28	3.30	0.98	1.02	0.90
(2*R*,3*S*,5*R*,7*S*,9*R*,11*R*)	1.25	1.55	2.32	1.72	2.45	4.07	1.45	2.03	2.21	1.48	1.61	3.30	3.32	0.92	1.02	0.86
(2*R*,3*S*,5*R*,7*S*,9*S*,11*R*)	1.39	1.57	2.08	1.24	2.47	4.18	1.43	1.85	1.58	0.86	1.77	3.34	3.36	0.99	1.02	0.94
(2*R*,3*S*,5*R*,7*S*,9*S*,11*S*)	1.25	1.78	2.15	1.20	2.45	4.16	1.47	1.84	1.59	1.32	1.34	3.34	3.37	0.95	1.01	0.94
(2*R*,3*S*,5*S*,7*S*,9*R*,11*S*)	1.37	1.75	2.48	2.00	2.03	3.95	1.63	2.07	2.88	1.21	1.92	3.33	3.33	1.03	0.96	0.93
(2*S*,3*S*,5*R*,7*S*,9*R*,11*S*)	1.20	1.66	1.64	1.50	2.52	3.94	1.24	1.59	1.94	0.94	1.76	3.42	3.45	0.96	1.02	0.91
(2*S*,3*S*,5*R*,7*S*,9*S*,11*S*)	1.49	2.04	2.33	1.37	3.23	4.15	1.77	1.89	2.24	1.14	2.32	3.52	3.53	0.90	0.96	0.92
(2*S*,3*S*,5*R*,7*S*,9*R*,11*R*)	1.10	1.59	1.66	1.52	2.44	3.98	1.31	1.60	1.80	1.29	1.35	3.38	3.41	1.05	1.02	0.92
(2*S*,3*S*,5*R*,7*S*,9*S*,11*R*)	1.44	1.77	2.29	1.54	2.66	4.17	1.74	1.86	2.24	1.66	1.78	3.31	3.42	1.12	0.96	0.92
(2*R*,3*S*,5*S*,7*S*,9*R*,11*R*)	1.27	1.57	2.49	2.00	2.04	3.91	1.85	1.92	2.85	1.51	1.61	3.32	3.34	0.94	0.95	0.87
(2*R*,3*S*,5*S*,7*S*,9*S*,11*S*)	1.22	1.79	2.49	1.59	1.77	3.90	1.35	2.30	1.60	1.30	1.39	3.37	3.38	0.97	0.95	1.01
(2*R*,3*S*,5*S*,7*S*,9*S*,11*R*)	1.51	1.38	2.51	1.60	1.83	3.90	1.33	2.25	1.54	1.00	1.73	3.33	3.35	1.02	0.96	0.99
(2*S*,3*S*,5*S*,7*S*,9*R*,11*S*)	1.22	1.70	1.74	1.77	2.05	3.90	1.18	1.99	2.55	0.96	1.73	3.43	3.46	0.99	0.96	0.93
(2*S*,3*S*,5*S*,7*S*,9*S*,11*S*)	1.51	2.26	2.46	1.67	2.80	3.96	1.74	2.26	2.32	1.03	3.01	3.49	3.49	0.89	0.90	0.91
(2*S*,3*S*,5*S*,7*S*,9*S*,11*R*)	1.51	2.26	2.46	1.67	2.79	3.96	1.74	2.26	2.32	1.02	3.00	3.49	3.49	0.89	0.90	0.91
(2*S*,3*S*,5*S*,7*S*,9*R*,11*R*)	1.22	1.70	1.74	1.77	2.05	3.90	1.18	1.99	2.55	0.96	1.73	3.43	3.46	0.99	0.96	0.93

Statistical analysis of δ^{13}C and ^{1}H for **1b** and its isomers.

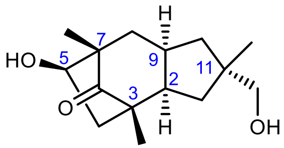

(2*R*,3*S*,5*R*,7*R*,9*R*,11*S*)
the mirror image of natural product

	^{13}C			^{1}H			^{1}H +^{13}C	
	RMSD (ppm)	max dev (ppm)	DP4	RMSD (ppm)	max dev (ppm)	DP4	DP4	
(2*R*,3*S*,5*R*,7*S*,9*R*,11*S*)	0.9	1.7	77.3%	0.12	0.29	94.9%	99.7%	proposed diasteromer
(2*R*,3*S*,5*R*,7*S*,9*R*,11*R*)	1.3	3.6	22.2%	0.17	0.43	0.9%	0.3%	11-epimer
(2*R*,3*S*,5*R*,7*S*,9*S*,11*R*)	2.9	7.3	0.0%	0.25	0.77	0.0%	0.0%	
(2*R*,3*S*,5*R*,7*S*,9*S*,11*S*)	2.7	6.8	0.0%	0.26	0.76	0.0%	0.0%	
(2*R*,3*S*,5*S*,7*S*,9*R*,11*S*)	2.0	5.2	0.1%	0.27	0.59	0.0%	0.0%	
(2*S*,3*S*,5*R*,7*S*,9*R*,11*S*)	3.0	7.6	0.0%	0.25	0.64	0.0%	0.0%	
(2*S*,3*S*,5*R*,7*S*,9*S*,11*S*)	3.4	8.6	0.0%	0.28	0.61	0.0%	0.0%	
(2*S*,3*S*,5*R*,7*S*,9*R*,11*R*)	2.9	7.5	0.0%	0.27	0.62	0.0%	0.0%	
(2*S*,3*S*,5*R*,7*S*,9*S*,11*R*)	3.2	6.9	0.0%	0.19	0.61	4.3%	0.0%	
(2*R*,3*S*,5*S*,7*S*,9*R*,11*R*)	2.2	6.2	0.3%	0.28	0.58	0.0%	0.0%	
(2*R*,3*S*,5*S*,7*S*,9*S*,11*S*)	4.1	12.1	0.0%	0.35	0.85	0.0%	0.0%	
(2*R*,3*S*,5*S*,7*S*,9*S*,11*R*)	4.2	11.9	0.0%	0.35	0.81	0.0%	0.0%	
(2*S*,3*S*,5*S*,7*S*,9*R*,11*S*)	3.7	9.1	0.0%	0.27	0.57	0.0%	0.0%	
(2*S*,3*S*,5*S*,7*S*,9*S*,11*S*)	4.3	9.9	0.0%	0.40	1.26	0.0%	0.0%	
(2*S*,3*S*,5*S*,7*S*,9*S*,11*R*)	4.3	9.9	0.0%	0.40	1.25	0.0%	0.0%	
(2*S*,3*S*,5*S*,7*S*,9*R*,11*R*)	3.7	9.1	0.0%	0.27	0.57	0.0%	0.0%	

Calculated ^{13}C NMR chemical shift of **1c** and its isomers.

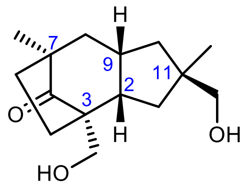

(2*S*,3*R*,7*S*,9*S*,11*R*)
natural enantiomer

	C-1	C-2	C-3	C-4	C-5	C-6	C-7	C-8	C-9	C-10	C-11	C-12	C-13	C-14	C-15
	36.5	55.5	54.6	27.7	29.7	220.9	47.3	49.9	36.5	41.1	45.1	71.7	28.4	63.3	19.9
(2*R*,3*R*,7*S*,9*R*,11*R*)	37.0	48.2	55.5	20.1	30.3	229.8	46.3	43.9	34.2	41.5	43.5	70.3	25.2	65.3	21.1
(2*R*,3*R*,7*S*,9*R*,11*S*)	38.6	49.4	55.2	21.9	30.3	229.5	46.2	43.7	34.2	43.3	43.5	71.1	24.5	65.1	21.0
(2*R*,3*R*,7*S*,9*S*,11*R*)	34.1	51.5	54.2	21.4	30.9	227.6	46.4	43.3	37.9	40.5	43.4	72.8	26.2	64.7	20.2
(2*R*,3*R*,7*S*,9*S*,11*S*)	35.9	52.2	53.8	21.4	31.0	227.6	46.5	43.5	37.0	38.7	43.4	72.6	26.3	64.6	20.2
(2*S*,3*R*,7*S*,9*R*,11*R*)	36.7	55.4	52.1	29.4	35.1	228.4	42.8	47.5	38.8	40.3	43.3	72.6	26.4	65.4	21.5
(2*S*,3*R*,7*S*,9*R*,11*S*)	35.5	53.9	53.6	28.7	35.3	228.0	42.8	47.1	40.2	43.9	45.0	75.6	26.1	66.6	21.5
(2*S*,3*R*,7S,9*S*,11*R*)	37.8	55.6	54.2	26.7	29.0	226.8	46.7	46.8	36.7	39.0	43.6	73.1	27.7	65.3	20.2
(2*S*,3*R*,7*S*,9*S*,11*S*)	36.0	55.0	53.6	26.8	29.2	227.0	46.7	45.2	36.3	39.2	44.4	71.1	27.1	65.6	20.0

Calculated ^{1}H NMR chemical shift of **1c** and its isomers.

	Hα-1	Hβ-1	H-2	Hα-4	Hβ-H	Hα-5	Hβ-5	H-8	H-8	H-9	Hα-10	Hβ-10	H-12	H-12	H-13	H-14	H-14	H-15
	0.96	1.71	2.52	2.06	1.88	1.56	2.01	1.58	1.75	2.56	1.03	1.78	3.13	3.19	1.03	3.37	3.59	0.87
(2*R*,3R,7*S*,9*R*,11*R*)	1.52	2.02	2.98	2.48	1.41	1.63	1.99	1.91	1.85	2.34	1.20	2.54	3.53	3.53	0.92	3.39	3.46	0.94
(2*R*,3R,7*S*,9*R*,11*S*)	1.36	1.78	2.93	1.84	1.58	1.67	1.99	1.88	1.82	2.33	1.82	1.87	3.33	3.46	1.11	3.38	3.44	0.94
(2*R*,3R,7*S*,9*S*,11*R*)	1.36	1.62	2.14	1.75	1.49	1.55	1.81	1.32	1.62	2.21	1.00	1.80	3.44	3.46	1.00	3.51	3.55	0.94
(2*R*,3R,7*S*,9*S*,11*S*)	1.12	1.64	2.26	1.65	1.38	1.57	1.82	1.43	1.65	2.05	1.39	1.56	3.36	3.43	1.02	3.41	3.60	0.95
(2*S*,3*R*,7*S*,9*R*,11*R*)	1.77	2.30	2.36	1.49	1.41	1.81	1.97	1.88	1.58	1.75	1.39	1.40	3.36	3.41	0.98	3.66	3.75	1.02
(2*S*,3*R*,7*S*,9*R*,11*S*)	1.59	2.11	2.33	1.47	1.13	1.80	1.98	1.87	1.40	2.73	1.16	1.85	3.37	3.45	0.88	3.66	3.82	1.03
(2*S*,3*R*,7S,9*S*,11*R*)	1.34	1.90	2.30	2.07	1.64	1.56	1.90	1.78	1.66	2.47	1.15	1.97	3.29	3.31	1.00	3.32	3.73	0.94
(2*S*,3*R*,7*S*,9*S*,11*S*)	1.57	2.31	2.31	2.03	1.65	1.56	1.89	2.12	1.50	2.46	1.63	1.47	3.32	3.35	0.92	3.36	3.71	0.92

Statistical analysis of δ^{13}C and ^{1}H for **1c** and its isomers.

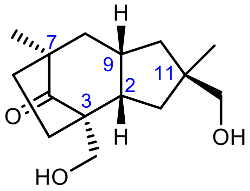

(2*S*,3*R*,7*S*,9*S*,11*R*)
natural enantiomer

	^{13}C			^{1}H			^{1}H+^{13}C	
	RMSD	max dev	DP4	RMSD	max dev	DP4	DP4	
(2*R*,3*R*,7*S*,9*R*,11*R*)	4.1	8.9	0.0%	0.34	0.76	0.0%	0.0%	
(2*R*,3*R*,7*S*,9*R*,11*S*)	3.8	8.6	0.0%	0.28	0.79	0.0%	0.0%	
(2*R*,3*R*,7*S*,9*S*,11*R*)	3.3	6.7	0.0%	0.24	0.40	0.0%	0.0%	
(2*R*,3*R*,7*S*,9*S*,11*S*)	3.2	6.7	0.0%	0.25	0.51	0.0%	0.0%	
(2*S*,3*R*,7*S*,9*R*,11*R*)	3.1	7.5	0.0%	0.40	0.81	0.0%	0.0%	
(2*S*,3*R*,7*S*,9*R*,11*S*)	3.3	7.1	0.0%	0.34	0.75	0.0%	0.0%	
(2*S*,3*R*,7S,9*S*,11*R*)	2.0	5.9	69.5%	0.16	0.38	100.0%	100.0%	proposed diastereomer
(2*S*,3*R*,7*S*,9*S*,11*S*)	2.2	6.1	30.5%	0.31	0.61	0.0%	0.0%	11-epimer

Calculated ^{13}C NMR chemical shift of **2a** and its isomers.

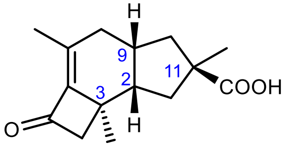

(2*S*,3*R*,9*R*,11*R*)
the natural enantiomer

	C-1	C-2	C-3	C-4	C-5	C-6	C-7	C-8	C-9	C-10	C-11	C-12	C-13	C-14	C-15
experimental	37.8	47.2	36.5	60.9	197.0	150.6	143.1	35.4	42.0	45.1	51.0	182.0	24.8	20.4	20.4
(2*R*,3*R*,9*R*11*R*)	38.1	49.9	39.3	47.9	195.0	147.3	138.8	35.5	44.6	41.5	47.5	178.4	26.6	28.6	19.9
(2*R*,3*R*,9*S*,11*R)*	40.1	45.2	35.9	53.1	195.0	143.8	139.0	32.5	37.4	43.2	47.5	178.7	25.3	26.4	19.4
(2*R*,3*R*,9*R*,11*S*)	39.2	50.2	39.0	47.9	195.2	147.5	138.7	35.5	44.1	40.1	47.5	178.4	26.4	28.6	19.9
(2*R*,3*R*,9*S*,11*S*)	42.0	45.1	36.2	54.3	195.2	146.7	139.9	33.3	38.4	45.4	48.7	178.1	24.0	24.2	20.6
(2*S*,3*R*,9*R*,11*R*)	37.7	47.8	36.7	58.6	194.3	150.3	142.0	34.7	41.9	45.9	50.6	178.2	24.2	20.8	19.3
(2*S*,3*R*,9*S*,11*R*)	40.0	49.8	36.4	54.3	195.4	146.8	137.7	37.4	37.3	42.2	47.3	178.9	26.3	21.2	18.5
(2*S*,3*R*,9*R*,11*S*)	38.0	49.0	37.1	58.7	194.3	150.3	141.5	34.7	42.1	45.5	49.2	177.7	23.6	20.7	19.3
(2*S*,3*R*,9*S*,11*S*)	37.0	50.4	36.9	54.4	195.4	147.3	137.5	36.8	37.6	44.7	47.3	179.1	26.1	21.4	18.5

Calculated ^{1}H NMR chemical shift of **2a** and its isomers.

	Hβ-1	Hα-1	H-2	Hβ-4	Hα-4	Hβ-8	Hα-8	H-9	Hα-10	Hβ-10	H-13	H-14	H-15
experimental	1.51	2.37	2.29	2.74	2.68	2.25	1.82	2.45	1.17	2.41	1.40	1.16	2.01
(2*R*,3*R*,9*R*11*R*)	1.8	2.0	1.80	2.66	1.97	1.85	1.77	1.86	1.13	2.26	1.35	1.32	1.86
(2*R*,3*R*,9*S*,11*R)*	1.7	1.8	2.36	2.74	2.31	2.13	2.14	2.41	1.64	2.28	1.33	1.28	1.87
(2*R*,3*R*,9*R*,11*S*)	1.2	2.5	2.02	2.54	1.96	1.86	1.86	1.62	1.58	2.01	1.31	1.32	1.86
(2*R*,3*R*,9*S*,11*S*)	1.2	2.3	2.51	2.85	2.40	2.13	1.80	2.72	1.03	2.51	1.27	1.22	1.94
(2*S*,3*R*,9*R*,11*R*)	1.5	2.4	2.40	2.66	2.47	2.11	1.81	2.49	1.19	2.38	1.37	1.16	1.92
(2*S*,3*R*,9*S*,11*R*)	1.4	2.3	1.85	2.59	2.30	2.29	1.73	1.98	1.76	2.12	1.35	1.16	1.82
(2*S*,3*R*,9*R*,11*S*)	1.7	2.5	2.17	2.59	2.50	2.16	1.93	2.51	1.83	2.07	1.28	1.23	1.93
(2*S*,3*R*,9*S*,11*S*)	1.6	2.3	1.66	2.55	2.32	2.27	1.60	2.24	1.09	2.42	1.34	1.24	1.82

Statistical analysis of δ^{13}C and ^{1}H for **2a** and its isomers.

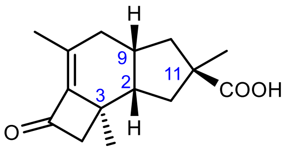

(2*S*,3*R*,9*R*,11*R*)
the natural enantiomer

	^{13}C			^{1}H			^{1}H+^{13}C	
	RMSD (ppm)	max dev (ppm)	DP4	RMSD (ppm)	max dev (ppm)	DP4	DP4	
(2*R*,3*R*,9*R*11*R*)	4.7	13.0	0.0%	0.34	0.71	0.0%	0.0%	
(2*R*,3*R*,9*S*,11*R*)	3.9	7.8	0.0%	0.27	0.62	0.0%	0.0%	
(2*R*,3*R*,9*R*,11*S*)	4.8	13.0	0.0%	0.39	0.83	0.0%	0.0%	
(2*R*,3*R*,9*S*,11*S*)	3.1	6.6	0.0%	0.18	0.34	0.1%	0.0%	
(2*S*,3*R*,9*R*,11*R*)	1.5	3.8	87.3%	0.09	0.21	99.9%	100.0%	proposed diastereomer
(2*S*,3*R*,9*S*,11*R*)	3.3	6.6	0.0%	0.29	0.59	0.0%	0.0%	
(2*S*,3*R*,9*R*,11*S*)	1.7	4.3	12.7%	0.24	0.66	0.0%	0.0%	11-epimer
(2*S*,3*R*,9*S*,11*S*)	3.2	6.5	0.0%	0.24	0.63	0.0%	0.0%	

Calculated ^{13}C NMR chemical shift of **2b** and its isomers.

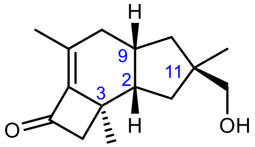

(2*S*,3*R*,9*R*,11*R*)
the natural enantiomer

	C-1	C-2	C-3	C-4	C-5	C-6	C-7	C-8	C-9	C-10	C-11	C-12	C-13	C-14	C-15
experiential	36.2	47.4	36.8	60.9	197.2	150.7	143.3	35.8	41.8	43.6	45.5	69.4	24.5	20.4	20.4
(2*R*,3*R*,9*R*,11*R*)	36.3	49.8	39.4	47.8	195.4	147.2	139.1	35.9	44.9	39.9	43.3	72.5	26.5	28.7	19.9
(2*R*,3*R*,9*S*,11*R*)	37.6	45.2	36.0	53.4	195.3	144.8	139.2	33.4	37.5	43.1	43.3	71.4	24.5	26.0	19.8
(2*R*,3*S*,9*R*,11*R*)	35.7	50.1	36.8	54.5	195.7	147.2	137.6	37.5	38.0	43.4	42.7	72.9	26.7	21.5	18.5
(2*R*,3*S*,9*S*,11*R*)	35.2	47.9	36.9	58.8	194.5	150.4	142.2	35.3	42.4	42.6	45.6	71.5	22.4	20.9	19.2
(2*S*,3*R*,9*R*,11*R*)	35.9	48.8	36.9	58.7	194.5	150.3	142.4	35.3	42.5	43.8	45.5	69.9	24.3	20.7	19.3
(2*S*,3*R*,9*S*,11*R*)	37.1	50.6	36.5	54.5	195.7	147.1	137.5	37.4	37.0	42.2	42.6	72.8	26.6	21.5	18.5
(2*S*,3*S*,9*R*,11*R*)	39.0	45.7	36.2	53.6	195.4	145.6	138.6	34.3	38.0	43.6	43.2	71.0	25.6	25.6	20.0
(2*S*,3*S*,9*S*,11*R*)	37.4	50.2	39.3	47.8	195.3	147.3	139.1	35.8	44.0	38.7	43.2	72.6	26.3	28.7	20.0

Calculated ^{1}H NMR chemical shift of **2b** and its isomers.

	Hα-1	Hβ-1	H-2	H-4	H-4	H-8	H-8	H-9	Hα-10	Hβ-10	H-12	H-12	H-13	H-14	H-15
experiential	1.43	1.73	2.18	2.66	2.69	1.43	2.25	2.41	1.06	1.92	3.4	3.43	1.13	1.18	2.01
(2*R*,3*R*,9*R*,11*R*)	1.43	1.73	2.18	2.66	2.69	1.43	2.25	2.41	1.06	1.92	3.4	3.43	1.13	1.18	2.01
(2*R*,3*R*,9*S*,11*R*)	1.54	1.54	1.82	2.62	1.95	1.76	1.83	1.71	1.07	1.79	3.40	3.42	1.05	1.31	1.85
(2*R*,3*S*,9*R*,11*R*)	1.33	1.46	2.45	2.32	2.78	2.07	2.10	2.48	1.53	1.59	3.40	3.41	1.02	1.27	1.89
(2*R*,3*S*,9*S*,11*R*)	1.46	1.79	1.70	2.31	2.56	1.60	2.27	2.07	1.02	2.04	1.04	3.42	3.45	1.20	1.81
(2*S*,3*R*,9*R*,11*R*)	1.44	1.83	2.30	2.48	2.60	1.86	2.12	2.55	1.43	1.53	3.49	3.50	1.05	1.18	1.93
(2*S*,3*R*,9*S*,11*R*)	1.52	1.74	2.18	2.48	2.59	1.83	2.12	2.51	1.13	1.99	3.49	3.52	1.08	1.18	1.92
(2*S*,3*S*,9*R*,11*R*)	1.32	1.81	1.73	2.30	2.56	1.65	2.28	2.02	1.43	1.65	3.44	3.43	1.06	1.19	1.81
(2*S*,3*S*,9*S*,11*R*)	1.17	1.77	2.43	2.34	2.83	1.80	2.17	2.48	1.16	1.93	3.44	3.45	1.01	1.25	1.91
(2*R*,3*R*,9*R*,11*R*)	1.09	1.90	1.86	1.94	2.56	1.84	1.83	1.64	1.37	1.54	3.43	3.45	1.04	1.30	1.86

Statistical analysis of δ^{13}C and ^{1}H for **2b** and its isomers.

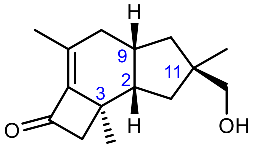

(2*S*,3*R*,9*R*,11*R*)
the natural enantiomer

	^{13}C RMSD (ppm)	max dev (ppm)	DP4	^{1}H RMSD (ppm)	max dev (ppm)	DP4	^{1}H+^{13}C DP4	
2R3R9R11R	4.7	13.1	0.0%	0.32	0.74	0.0%	0.0%	
(2R3R9S11R)	3.5	7.5	0.0%	0.27	0.64	0.0%	0.0%	
(2R3S9R11R)	3.1	6.4	0.0%	0.88	2.36	0.0%	0.0%	
(2R3S9S11R)	1.3	2.7	12.3%	0.20	0.43	0.2%	0.0%	11-epimer
(2S3R9R11R)	1.1	2.7	87.7%	0.14	0.40	97.2%	100.0%	proposed diastereomer
(2S3R9S11R)	3.2	6.4	0.0%	0.24	0.45	0.0%	0.0%	
(2S3S9R11R)	3.4	7.3	0.0%	0.17	0.37	2.6%	0.0%	
(2S3S9S11R)	4.7	13.1	0.0%	0.37	0.77	0.0%	0.0%	

Calculated ^{13}C NMR chemical shift of **3** and its isomers.

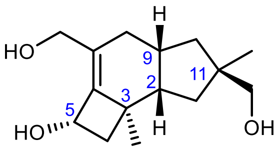

(2*S*,3*R*,5*S*,9*R*,11*R*)
the natural enantiomer

	C-1	C-2	C-3	C-4	C-5	C-6	C-7	C-8	C-9	C-10	C-11	C-12	C-13	C-14	C-15
experiential	35.7	47.3	43.1	46.6	69.3	146.1	134.1	29.4	41.6	43.7	45.1	69.4	24.4	21.8	63.7
2S3R5R9S11R	36.1	50.3	37.0	48.0	68.8	143.6	135.6	30.2	40.5	43.1	45.2	69.5	24.4	21.6	64.5
2S3R5S9S11R	35.8	48.0	40.8	44.8	69.3	144.2	137.4	30.1	41.8	43.6	45.1	69.8	24.4	22.1	65.6
2S3S5R9S11R	41.2	45.1	40.9	39.9	69.4	141.6	136.8	27.9	39.0	44.1	43.5	69.6	24.2	25.3	66.7
2R3R5R9R11R	39.6	47.4	39.7	41.5	70.4	146.8	134.2	33.1	37.8	45.3	43.3	71.8	27.7	24.6	64.2
2R3R5R9S11R	35.2	51.1	40.6	38.0	72.9	147.3	131.9	31.8	44.8	39.8	43.2	72.6	26.4	27.0	64.6
2R3R5S9R11R	39.4	45.7	42.9	40.0	69.5	143.7	135.7	28.7	38.7	43.3	43.2	70.9	24.2	24.7	64.4
2R3S5R9R11R	35.2	47.3	40.8	44.8	69.3	144.2	137.5	30.0	41.6	43.1	45.1	72.5	22.2	22.1	65.6
2R3S5R9S11R	35.3	50.1	43.8	42.2	73.2	141.7	134.0	32.2	37.4	43.2	42.5	72.8	26.6	21.9	65.7
2R3S5S9R11R	35.1	49.6	36.8	48.4	69.0	143.2	136.1	30.2	40.4	42.2	45.2	71.6	22.2	21.8	64.4
2S3R5R9R11R	36.3	52.0	37.4	43.9	72.2	143.9	130.2	33.3	37.0	41.9	42.5	72.8	26.5	21.2	64.5
2S3R5S9R11R	36.4	51.1	43.6	42.2	73.2	141.5	134.4	32.0	36.8	42.0	42.4	72.6	26.7	21.7	65.7
2S3S5R9R11R	36.2	52.2	47.3	36.5	74.9	141.8	137.5	30.5	44.7	38.4	43.3	72.6	26.3	28.8	65.4
2S3S5S9R11R	36.2	52.0	40.8	37.8	72.8	148.2	130.9	31.8	43.8	38.5	43.2	72.6	26.3	26.9	64.7
2R3R5S9S11R	34.9	51.5	47.6	36.1	75.2	143.1	137.3	30.8	45.7	40.0	43.2	72.7	26.4	28.6	65.2
2R3S5S9S11R	34.9	51.3	37.8	43.8	72.1	145.0	129.2	33.4	37.8	43.4	42.5	73.0	26.6	21.3	64.5
2S3S5S9S11R	38.0	46.3	37.0	40.4	71.3	140.2	129.0	31.2	35.6	42.1	42.3	73.4	27.8	27.3	64.1

Calculated ^{1}H NMR chemical shift of **3** and its isomers.

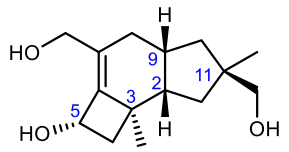

(2*S*,3*R*,5*S*,9*R*,11*R*)
the natural enantiomer

	Hα-1	Hβ-1	H-2	Hα-4	Hβ-4	H-5	Hα-8	Hβ-8	H-9	Hα-10	Hβ-10	H-12A	H-12B	H-13	H-14	H-15A	H-15B
experimental	1.29	1.59	2.09	1.88	2.18	4.97	1.72	2.04	2.35	0.98	1.82	3.37	3.38	1.05	1.25	4.15	4.21
2S3R5R9S11R	1.39	1.58	2.14	1.74	2.38	4.90	1.64	1.72	2.34	0.98	1.96	3.53	3.44	1.06	1.06	4.02	4.17
2S3R5S9S11R	1.38	1.62	2.17	1.86	2.07	4.95	1.74	1.72	2.43	1.04	1.90	3.49	3.45	1.03	1.29	4.14	4.15
2S3S5R9S11R	1.11	1.82	2.22	1.73	2.35	4.89	1.38	2.03	2.46	1.02	1.82	3.45	3.45	1.01	1.31	4.08	4.20
2R3R5R9R11R	1.31	1.82	2.14	1.94	2.07	4.91	2.12	2.35	2.33	1.73	1.90	3.31	3.42	0.91	1.12	3.59	4.43
2R3R5R9S11R	1.36	1.43	1.53	1.59	1.95	4.97	1.57	1.65	1.65	1.01	1.70	3.37	3.39	1.01	1.18	4.05	4.07
2R3R5S9R11R	1.23	1.54	2.26	1.74	2.29	4.87	1.81	2.02	2.51	1.57	1.66	3.40	3.36	0.94	1.35	3.91	4.25
2R3S5R9R11R	1.34	1.53	2.25	1.85	2.08	4.96	1.72	1.76	2.47	1.30	1.52	3.45	3.46	1.04	1.26	4.14	4.16
2R3S5R9S11R	1.31	1.54	1.65	1.83	1.90	5.07	1.41	1.96	1.98	0.92	1.97	3.38	3.39	1.01	1.40	4.00	4.14
2R3S5S9R11R	1.34	1.68	2.27	1.74	2.41	4.91	1.66	1.71	2.37	1.25	1.44	3.45	3.45	1.03	1.05	4.01	4.16
2S3R5R9R11R	1.19	1.65	1.65	1.58	2.25	5.01	1.56	1.94	1.92	1.43	1.54	3.38	3.35	0.99	1.07	3.97	4.07
2S3R5S9R11R	1.23	1.68	1.67	1.82	1.90	5.07	1.47	1.96	1.96	1.42	1.54	3.34	3.38	3.38	1.39	4.00	4.15
2S3S5R9R11R	0.99	1.75	1.56	1.50	1.89	5.12	1.56	1.66	1.54	1.26	1.35	3.34	3.38	0.96	1.61	4.11	4.10
2S3S5S9R11R	1.06	1.79	1.56	1.55	1.95	4.97	1.58	1.69	1.65	1.31	1.34	3.36	3.39	0.99	1.17	4.08	4.08
2R3R5S9S11R	1.35	1.56	1.57	1.56	1.99	5.07	1.58	1.61	1.58	1.02	1.65	3.35	3.39	0.96	1.62	4.10	4.05
2R3S5S9S11R	1.34	1.71	1.65	1.61	2.26	5.01	1.50	1.93	1.97	0.98	1.91	3.40	3.38	1.00	1.10	3.98	4.08
2S3S5S9S11R	1.41	1.61	1.91	1.73	2.11	5.00	1.65	1.95	2.16	1.33	1.86	3.36	3.34	1.07	1.73	4.02	3.99

Statistical analysis of δ^{13}C and ^{1}H for **3** and its isomers

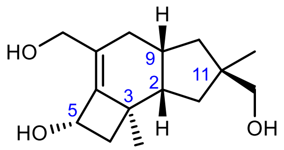

(2*S*,3*R*,5*S*,9*R*,11*R*)
the natural enantiomer

	^{13}C			^{1}H			^{1}H+^{13}C	
	RMSD (ppm)	max dev (ppm)	DP4	RMSD (ppm)	max dev (ppm)	DP4	DP4	
2S3R5R9S11R	2.0	6.1	3.6%	0.13	0.32	4.1%	0.2%	
2S3R5S9S11R	1.4	3.3	88.5%	0.10	0.32	90.4%	99.5%	proposed diastereomer
2S3S5R9S11R	3.1	6.7	0.0%	0.14	0.34	2.4%	0.0%	
2R3R5R9R11R	2.7	5.1	0.0%	0.28	0.75	0.0%	0.0%	
2R3R5R9S11R	3.6	8.6	0.0%	0.27	0.70	0.0%	0.0%	
2R3R5S9R11R	2.5	6.6	0.0%	0.18	0.59	0.1%	0.0%	
2R3S5R9R11R	1.7	3.4	7.6%	0.14	0.32	2.9%	0.3%	enantiomer of the 11-epimer
2R3S5R9S11R	2.8	4.4	0.0%	0.19	0.44	0.0%	0.0%	
2R3S5S9R11R	2.3	6.3	0.2%	0.17	0.38	0.0%	0.0%	
2S3R5R9R11R	3.2	5.7	0.0%	0.23	0.45	0.0%	0.0%	
2S3R5S9R11R	3.0	4.8	0.0%	0.61	2.33	0.0%	0.0%	
2S3S5R9R11R	4.6	10.1	0.0%	0.34	0.81	0.0%	0.0%	
2S3S5S9R11R	3.8	8.8	0.0%	0.30	0.70	0.0%	0.0%	
2R3R5S9S11R	4.5	10.5	0.0%	0.29	0.77	0.0%	0.0%	
2R3S5S9S11R	3.1	5.3	0.0%	0.18	0.44	0.0%	0.0%	
2S3S5S9S11R	4.1	6.2	3.6%	0.18	0.48	4.1%	0.2%	

Calculated ^{13}C NMR chemical shift of **4** and its isomers.

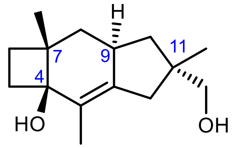

(4*S*,7*R*,9*R*,11*S*)
the mirror image of natural product

	C-1	C-2	C-3	C-4	C-5	C-6	C-7	C-8	C-9	C-10	C-11	C-12	C-13	C-14	C-15
experimental	39.9	140.1	126.6	73.4	34.5	22.0	43.8	35.4	37.1	43.0	42.1	70.5	25.4	12.8	23.4
(4*S*,7*R*,9*R*,11*S*)	39.4	140.2	131.1	73.1	34.2	21.6	42.6	35.1	37.5	42.6	42.1	71.3	24.9	14.8	24.0
(4*S*,7*R*,9*S*,11*S*)	39.5	141.8	132.2	78.5	29.6	28.6	40.7	43.7	35.8	41.6	42.5	72.4	24.0	13.2	19.9
(4*S*,7*R*,9*R*,11*R*)	40.1	140.8	130.9	73.0	34.2	21.6	42.5	34.7	37.1	41.6	42.5	72.8	24.5	14.8	23.9
(4*S*,7*R*,9*S*,11*R*)	39.0	141.5	132.4	78.6	29.7	28.6	40.8	44.4	36.0	42.1	42.4	71.0	24.5	13.3	19.9
(4*R*,7*R*,9*R*,11*S*)	37.2	140.0	134.4	79.5	30.0	31.4	43.1	31.6	38.5	44.7	41.4	71.0	25.2	15.0	26.3
(4*R*,7*R*,9*S*,11*S*)	40.4	140.4	132.0	79.1	31.1	32.3	42.4	35.5	39.2	40.6	41.7	76.6	25.4	14.0	24.3
(4*R*,7*R*,9*R*,11*R*)	36.8	139.7	134.7	79.6	30.1	31.4	43.2	31.7	38.4	43.7	41.8	71.8	24.2	15.1	26.2
(4*R*,7*R*,9*S*11*R*)	36.3	139.4	135.9	80.1	31.0	32.3	42.6	33.7	40.0	43.2	41.7	72.3	26.3	14.1	24.3

Calculated ^{1}H NMR chemical shift of **4** and its isomers.

	Hβ-1	Hα-1	Hβ-5	Hα-5	Hβ-6	Hα-6	Hβ-8	Hα-8	H-9	Hβ-10	Hα-10	H-12	H-12	H-13	H-14	H-15
experimental	2.25	2.07	2.00	2.12	1.48	1.21	1.57	0.84	2.47	1.95	1.05	3.41	3.49	1.09	1.65	1.20
(4*S*,7*R*,9*R*,11*S*)	2.30	2.05	2.01	2.10	1.58	1.28	1.47	0.86	2.68	1.93	1.13	3.43	3.53	1.04	1.64	1.17
(4*S*,7*R*,9*S*,11*S*)	2.33	1.98	1.91	1.99	1.49	1.99	1.80	1.69	2.56	1.51	1.35	3.43	3.44	1.01	1.59	1.07
(4*S*,7*R*,9*R*,11*R*)	2.29	2.10	2.02	2.09	1.60	1.29	1.46	0.99	2.68	1.58	1.40	3.42	3.45	1.07	1.67	1.17
(4*S*,7*R*,9*S*,11*R*)	2.20	2.05	1.92	1.98	1.99	1.48	1.83	1.57	2.55	1.86	1.09	3.38	3.46	1.02	1.58	1.07
(4*R*,7*R*,9*R*,11*S*)	2.27	2.11	2.25	1.83	2.47	1.58	2.29	1.10	2.74	1.88	1.25	3.38	3.47	1.02	1.66	0.89
(4*R*,7*R*,9*S*,11*S*)	2.13	2.01	2.24	1.84	1.48	2.51	2.13	1.55	2.64	1.79	1.71	3.53	3.37	0.91	1.68	1.17
(4*R*,7*R*,9*R*,11*R*)	2.43	1.96	2.27	1.84	1.58	2.47	2.32	1.12	2.77	1.45	1.44	3.40	3.41	1.04	1.65	0.87
(4*R*,7*R*,9*S*11*R*)	2.20	1.87	2.29	1.89	1.51	2.52	1.97	1.48	2.66	1.89	0.89	3.42	3.41	0.96	1.68	1.18

Statistical analysis of $\delta^{13}C$ and ^{1}H for **4** and its isomers.

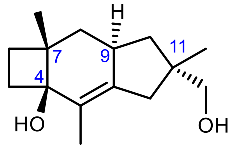

(4*S*,7*R*,9*R*,11*S*)
the mirror image of natural product

	^{13}C			^{1}H			$^{1}H+^{13}C$	
	RMSD (ppm)	max dev (ppm)	DP4	RMSD (ppm)	max dev (ppm)	DP4	DP4	
(4*S*,7*R*,9*R*,11*S*)	1.4	4.5	83.5%	0.07	0.21	99.7%	99.9%	proposed diastereomer
(4*S*,7*R*,9*S*,11*S*)	3.9	8.3	0.0%	0.33	0.85	0.0%	0.0%	11-epimer
(4*S*,7*R*,9*R*,11*R*)	1.5	4.3	16.5%	0.15	0.37	0.3%	0.1%	
(4*S*,7*R*,9*S*,11*R*)	4.0	9.0	0.0%	0.25	0.73	0.0%	0.0%	
(4*R*,7*R*,9*R*,11*S*)	4.1	9.4	0.0%	0.36	0.99	0.0%	0.0%	
(4*R*,7*R*,9*S*,11*S*)	3.9	10.3	0.0%	0.45	1.30	0.0%	0.0%	
(4*R*,7*R*,9*R*,11*R*)	4.1	9.4	0.0%	0.44	1.26	0.0%	0.0%	
(4*R*,7*R*,9*S*11*R*)	4.3	10.3	0.0%	0.40	1.31	0.0%	0.0%	

Calculated ^{13}C NMR chemical shift of **5** and its isomers.

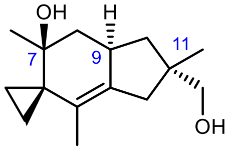

(7*S*,9*R*,11*S*)
the mirror image of natural product

	C-1	C-2	C-3	C-4	C-5	C-6	C-7	C-8	C-9	C-10	C-11	C-12	C-13	C-14	C-15
experimental	39.9	135.4	125.2	5.7	7.7	30.2	70.4	43.4	39.1	42.5	42.4	70.6	25.6	13.2	25.8
(7*S*,9*S*,11*S*)	40.7	141.2	125.1	6.7	11.4	31.0	72.0	41.9	36.0	40.8	42.7	72.6	24.4	14.1	26.3
(7S,9*S*,11*R*)	40.2	140.5	125.4	7.0	11.0	30.7	71.8	42.1	36.7	42.1	42.1	71.4	25.3	14.4	26.0
(7*S*,9*R*,11*R*)	39.0	136.7	128.5	7.5	8.9	30.6	70.0	42.7	39.3	42.0	42.5	72.5	25.2	15.2	25.8
(7*S*,9*R*,11*S*)	39.1	136.8	128.5	7.3	9.0	30.6	69.8	43.3	39.8	42.3	42.3	71.7	25.6	15.1	25.8

Calculated ^{13}C NMR chemical shift of **5** and its isomers.

	Hα-1	Hβ-1	H_S-4	H_R-4	H_S-5	H_R-5	Hβ-8	Hα-8	H-9	Ha-10	Hb10	H-12a	H-12b	H-13	H-14	H-15
experimental	2.05	2.14	0.75	0.69	0.81	0.48	1.37	1.87	2.55	1.02	1.94	3.49	3.40	1.09	1.28	1.20
(7*S*,9*S*,11*S*)	2.07	2.36	0.73	0.68	0.79	0.65	1.33	1.89	3.15	1.44	1.55	3.46	3.43	1.07	1.21	0.80
(7S,9*S*,11*R*)	2.04	2.29	0.64	0.84	0.71	0.66	1.26	1.87	3.05	1.19	1.98	3.57	3.41	1.05	1.20	0.83
(7*S*,9*R*,11*R*)	1.96	2.34	0.85	0.72	0.45	0.82	1.44	1.68	2.76	1.34	1.48	3.44	3.43	1.09	1.24	1.22
(7*S*,9*R*,11*S*)	2.02	2.20	0.84	0.43	0.89	0.73	1.40	1.65	2.73	1.13	1.90	3.51	3.41	1.05	1.24	1.22

Statistical analysis of δ^{13}C and ^{1}H for **5** and its isomers.

	δ^{13}C			δ^{1}H			^{1}H+^{13}C	
	RMSD	max dev	DP4	RMSD	max dev	DP4	DP4	
(7*S*,9*S*,11*S*)	2.2	5.8	0.1%	0.24	0.60	0.0%	0.0	
(7S,9*S*,11*R*)	1.8	5.1	2.5%	0.18	0.50	0.6%	0.0	
(7*S*,9*R*,11*R*)	1.3	3.3	30.3%	0.21	0.46	0.0%	0.0	11-epimer
(7*S*,9*R*,11*S*)	1.2	3.3	67.0%	0.12	0.26	99.3%	100.0	proposed diastereomer

Calculated ^{13}C NMR chemical shift of **6** and its isomers.

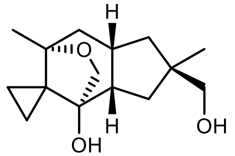

(2*S*,3*R*,7*R*,9*S*,11*R*)
the natural enantiomer

	C-1	C-2	C-3	C-4	C-5	C-6	C-7	C-8	C-9	C-10	C-11	C-12	C-13	C-14	C-15
experimental	37.2	46.6	78.0	2.3	3.2	34.3	83.1	39.2	35.4	43.5	43.2	70.3	24.3	71.2	21.7
(2*S*,3*R*,7*R*,9*S*,11*R*)	37.3	47.7	78.2	4.1	4.8	35.3	83.7	39.0	36.7	43.4	42.9	72.2	24.5	73.1	22.7
(2*S*,3*R*,7*R*,9*S*,11*S*)	36.6	46.7	78.4	4.1	4.9	35.4	83.6	39.1	36.5	42.2	43.1	71.0	26.0	72.4	22.8
(2*S*,3*R*,7*R*,9*R*,11*R*)	36.0	51.5	76.8	4.8	4.6	38.1	83.6	41.7	38.6	39.9	42.2	72.5	26.4	72.9	22.2
(2*S*,3*R*,7*R*,9*R*,11*S*)	34.9	50.5	76.7	4.6	4.9	38.0	83.5	41.8	39.6	41.0	42.2	72.6	26.4	73.1	22.1
(2*R*,3*R*,7*R*,9*S*,11*R*)	36.1	51.5	74.5	2.2	4.9	32.5	83.1	41.6	39.1	40.9	42.5	72.6	26.3	78.4	23.3
(2*R*,3*R*,7*R*,9*R*,11*S*)	35.5	51.0	76.3	2.1	6.6	33.1	82.8	43.4	36.6	39.9	43.7	72.2	27.2	78.5	21.9
(2*R*,3*R*,7*R*,9*R*,11*R*)	34.4	50.6	76.4	2.1	6.7	33.1	83.1	41.7	36.8	40.0	44.2	71.6	26.7	78.4	21.9
(2*R*,3*R*,7*R*,9*S*,11*S*)	37.2	52.4	74.4	2.1	4.9	32.4	83.1	41.6	38.0	39.9	42.5	72.5	26.5	78.5	23.4
7,12,14-triol (2*S*,3*R*,7*R*,9*S*,11*R*)	34.7	46.2	73.4	6.4	6.0	29.5	74.7	46.4	33.6	41.8	44.2	69.5	24.5	71.3	27.8

Calculated ^{1}H NMR chemical shift of **6** and its isomers.

	Hα-1	Hβ-1	H-2	H_R-4	H_S-4	H_R-5	H_S-5	Hα-8	Hβ-8	H-9	Hα-10	Hβ-10	H-12	H-12	H-13	Hβ-14	Hα-14	H-15
experimental	1.53	1.78	2.32	0.52	0.30	0.65	0.68	1.63	1.67	2.45	1.62	1.75	3.31	3.42	1.15	3.48	3.97	1.2
(2*S*,3*R*,7*R*,9*S*,11*R*)	1.80	1.60	2.19	0.33	0.55	0.65	0.74	1.48	1.59	2.45	1.70	2.08	3.35	3.44	1.08	3.53	3.95	0.88
(2*S*,3*R*,7*R*,9*S*,11*S*)	1.50	2.15	2.29	0.59	0.32	0.64	0.73	1.53	1.60	2.50	1.20	2.34	3.50	3.50	0.97	3.39	4.34	0.87
(2*S*,3*R*,7*R*,9*R*,11*R*)	1.18	1.70	1.95	0.75	0.64	0.36	0.54	1.21	1.60	2.24	1.48	1.41	3.42	3.39	1.05	3.48	3.89	0.87
(2*S*,3*R*,7*R*,9*R*,11*S*)	1.31	1.55	1.95	0.59	0.35	0.65	0.79	1.17	1.58	2.21	1.08	1.76	3.43	3.46	1.02	3.48	3.91	0.86
(2*R*,3*R*,7*R*,9*S*,11*R*)	1.41	1.63	2.11	0.58	0.43	0.77	0.51	1.38	1.56	2.37	1.02	1.76	3.38	3.42	1.01	3.39	3.51	0.89
(2*R*,3*R*,7*R*,9*R*,11*S*)	1.95	1.72	2.32	0.61	0.51	0.61	0.86	1.50	1.56	2.56	1.21	1.84	3.28	3.38	1.07	3.65	3.70	0.83
(2*R*,3*R*,7*R*,9*R*,11*R*)	1.22	2.45	2.38	0.54	0.49	0.64	0.85	1.42	1.67	2.58	1.49	1.57	3.39	3.39	0.96	3.63	3.73	0.81
(2*R*,3*R*,7*R*,9*S*,11*S*)	1.24	1.80	2.09	0.58	0.43	0.53	0.79	1.42	1.59	2.38	1.29	1.41	3.39	3.41	1.03	3.48	3.36	0.90
7,12,14-triol (2*S*,3*R*,7*R*,9*S*,11*R*)	1.79	1.28	2.40	0.91	0.18	0.66	0.73	1.65	1.70	2.23	0.86	1.92	3.42	3.48	1.03	3.50	4.10	0.82

Statistical analysis of δ^{13}C and ^{1}H for **6** and its isomers.

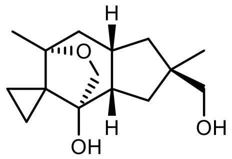

(2*S*,3*R*,7*R*,9*S*,11*R*)
the natural enantiomer

	^{13}C			^{1}H			^{1}H+^{13}C	
	RMSD (ppm)	max dev (ppm)	DP4	RMSD (ppm)	max dev (ppm)	DP4	DP4	
(2*S*,3*R*,7*R*,9*S*,11*R*)	1.07	1.9	47.9%	0.16	0.33	98.5%	99.9%	proposed diastereomer
(2*S*,3*R*,7*R*,9*S*,11*S*)	1.01	1.7	52.1%	0.24	0.59	0.1%	0.1%	11-epimer
(2*S*,3*R*,7*R*,9*R*,11*R*)	2.44	4.9	0.0%	0.24	0.42	0.0%	0.0%	
(2S,3R,7*R*,9R,11S)	2.41	4.2	0.0%	0.23	0.54	0.1%	0.0%	
(2*R*,3*R*,7*R*,9*S*,11*R*)	3.01	7.2	0.0%	0.23	0.60	0.1%	0.0%	
(2*R*,3*R*,7*R*,9*R*,11*S*)	3.01	7.3	0.0%	0.20	0.42	1.1%	0.0%	
(2*R*,3*R*,7*R*,9*R*,11*R*)	2.84	7.2	0.0%	0.24	0.67	0.1%	0.0%	
(2*R*,3*R*,7*R*,9*S*,11*S*)	3.11	7.3	0.0%	0.23	0.61	0.1%	0.0%	
7,12,14-triol (2*S*,3*R*,7*R*,9*S*,11*R*)	3.99	8.4	0.0%	0.27	0.76	0.0%	0.0%	

Calculated ^{13}C NMR chemical shift of **7** and its isomers.

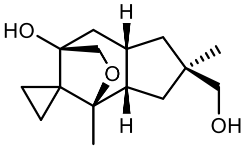

(2*S*,3*R*,7*R*.9*S*,11*R*)
the natural enantiomer

	C-1	C-2	C-3	C-4	C-5	C-6	C-7	C-8	C-9	C-10	C-11	C-12	C-13	C-14	C-15
experimental	37.3	53.1	83.7	0.1	4.1	32.2	75.8	41.9	35.9	41.8	44.2	70.9	27.1	20.5	73.7
(2*R*,3*S*,7*S*,9*R*,11*R*)	36.3	53.2	83.7	2.0	6.2	33.3	75.6	40.3	36.7	40.2	44.6	71.3	26.7	21.8	74.9
(2*R*,3*S*,7*S*,9*S*,11*R*)	37.8	55.0	83.3	4.6	1.2	33.0	76.9	41.7	38.0	40.3	42.7	72.6	26.2	19.0	79.7
(2*R*,3*S*,7*S*,9*S*,11*S*)	38.9	55.8	83.1	4.5	1.1	33.0	76.9	41.6	36.8	39.5	42.7	72.4	26.5	18.9	79.7
(2*S*,3*R*,7*R*,9*S*,11*R*)	37.4	53.6	83.8	1.9	6.1	33.5	75.7	41.8	36.7	40.4	44.2	72.2	27.3	21.8	74.9
(2*S*,3*S*,7*S*,9*R*,11*R*)	35.9	53.8	83.2	4.6	4.9	38.0	76.6	39.6	37.8	40.1	42.3	72.7	26.2	19.1	77.1
(2*S*,3*S*,7*S*,9*S*,11*R*)	37.3	46.7	85.5	3.7	4.8	35.0	74.7	38.5	34.5	43.1	43.0	70.0	24.3	20.6	76.1
(2*S*,3*S*,7*S*,9*S*,11*S*)	37.7	49.5	87.9	4.6	5.2	36.2	74.8	37.8	37.2	43.1	43.0	71.4	28.3	19.9	75.7
(2*S*,3*S*,7*S*,9*R*,11*S*)	35.4	52.6	83.2	4.6	4.8	37.9	76.3	39.9	38.8	40.6	42.3	72.6	26.4	19.1	77.3

Calculated ^{1}H NMR chemical shift of **7** and its isomers.

	Hα-1	Hb-1	H-2	H_R-4	H_S-4	H_R-5	H_S-5	Hα-8	Hβ-8	H-9	Hα-10	Hβ-10	H-12	H-12	H-13	H-14	Hα-15	Hβ-15
experimental	1.54	1.51	2.18	0.49	0.58	0.75	0.65	1.7	1.89	2.53	1.19	1.82	3.25	3.35	1.17	0.98	3.57	3.91
(2*R*,3*S*,7*S*,9*R*,11*R*)	1.02	2.19	2.29	0.49	0.66	0.78	0.62	1.91	1.45	2.53	1.52	1.58	3.35	3.37	0.95	0.93	3.57	3.79
(2*R*,3*S*,7*S*,9*S*,11*R*)	1.41	1.35	2.01	0.59	0.75	0.44	0.33	1.68	1.92	2.31	0.98	1.69	3.34	3.39	0.98	0.90	3.67	3.47
(2*R*,3*S*,7*S*,9*S*,11*S*)	1.79	0.90	1.98	0.56	0.77	0.44	0.34	1.72	1.94	2.29	1.26	1.36	3.38	3.37	0.99	0.88	3.47	3.67
(2*S*,3*R*,7*R*,9*S*,11*R*)	1.70	1.56	2.23	0.51	0.58	0.80	0.68	1.74	1.64	2.48	1.19	1.82	3.27	3.37	1.08	0.92	3.51	3.80
(2*S*,3*S*,7*S*,9*R*,11*R*)	1.50	1.31	1.65	0.35	0.56	0.68	0.67	1.45	1.75	2.21	1.34	1.43	3.34	3.37	1.05	0.83	3.59	3.83
(2*S*,3*S*,7*S*,9*S*,11*R*)	1.84	1.47	1.90	0.35	0.57	0.63	0.74	1.69	1.81	2.47	1.65	1.80	3.30	3.47	1.06	0.77	3.63	3.85
(2*S*,3*S*,7*S*,9*S*,11*S*)	2.26	1.41	1.88	0.34	0.66	0.70	0.79	1.72	1.85	2.50	1.39	2.30	3.36	3.41	0.90	0.95	3.62	3.94
(2*S*,3*S*,7*S*,9*R*,11*S*)	1.48	1.15	1.66	0.36	0.63	0.68	0.72	1.48	1.67	2.15	1.03	1.83	3.42	3.42	0.99	0.83	3.60	3.79

Statistical analysis of δ^{13}C and ^{1}H for **7** and its isomers.

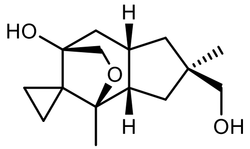

(2*S*,3*R*,7*R*.9*S*,11*R*)
the natural enantiomer

	^{13}C			^{1}H		^{1}H+^{13}C	
	RMSD (ppm)	max dev (ppm)	DP4	RMSD (ppm)	max dev (ppm)	DP4	
(2*R*,3*S*,7*S*,9*R*,11*R*)	1.1	2.1	30.4%	0.25	0.65	0.0%	11-epimer
(2*R*,3*S*,7*S*,9*S*,11*R*)	2.4	6.0	0.0%	0.19	0.44	0.0%	
(2*R*,3*S*,7*S*,9*S*,11*S*)	2.5	6.0	0.0%	0.25	0.64	0.0%	
(2*S*,3*R*,7*R*,9*S*,11*R*)	1.1	2.0	69.6%	0.09	0.25	100.0%	proposed diastereomer
(2*S*,3*S*,7*S*,9*R*,11*R*)	2.5	5.8	0.0%	0.21	0.53	0.0%	
(2*S*,3*S*,7*S*,9*S*,11*R*)	2.6	6.4	0.0%	0.17	0.46	0.0%	
(2*S*,3*S*,7*S*,9*S*,11*S*)	2.6	4.5	0.0%	0.24	0.72	0.0%	
(2*S*,3*S*,7*S*,9*R*,11*S*)	2.5	5.7	0.0%	0.21	0.52	0.0%	

Calculated ^{13}C NMR chemical shift of **8** and its isomers.

	C-1	C-2	C-3	C-4	C-5	C-6	C-7	C-8	C-9	C-10	C-11	C-12	C-13	C-14	C-15
	42.1	48.2	81.1	41.4	23.2	49.0	19.5	22.6	29.7	81	38	19.1	28.7	25.7	19.4
(2*R*,3*R*,6*R*,7*R*,9*R*,10*R*)	35.2	48.8	82.3	36.5	25.3	44.7	18.6	14.7	28.2	75.9	38.0	24.0	32.1	26.2	30.2
(2*R*,3*R*,6*R*,7*R*,9*S*,10*R*)	41.2	55.4	82.4	38.5	22.4	45.5	17.3	18.9	24.6	76.0	42.2	28.9	28.7	25.8	25.2
(2*R*,3*R*,6*R*,7*S*,9*R*,10*R*)	37.3	52.6	79.3	42.8	25.7	52.2	17.7	21.6	29.2	78.0	41.8	26.0	28.3	25.3	21.0
(2*R*,3*R*,6*R*,7*S*,9*S*,10*R*)	37.4	47.8	79.2	41.4	26.3	45.1	17.4	21.5	27.3	79.1	37.5	23.4	25.1	23.3	24.0
(2*R*,3*R*,6*S*,7*R*,9*R*,10*R*)	36.8	48.2	78.9	42.2	23.8	45.1	21.6	17.0	27.3	76.8	37.7	23.9	31.3	23.3	20.1
(2*R*,3*R*,6*S*,7*R*,9*S*,10*R*)	40.2	52.8	77.0	40.1	21.9	52.8	16.5	20.7	31.7	76.3	41.5	28.5	28.7	25.1	18.7
(2*R*,3*R*,6*S*,7*S*,9*R*,10*R*)	36.0	57.6	75.1	40.1	23.4	48.7	16.8	22.7	24.0	78.5	41.9	27.7	27.4	25.5	30.2
(2*R*,3*R*,6*S*,7*S*,9*S*,10*R*)	37.4	46.9	79.0	40.3	27.1	44.1	23.7	14.5	32.4	78.1	36.3	26.8	27.7	22.4	27.1
(2*R*,3*S*,6*R*,7*R*,9*R*,10*R*)	31.9	46.4	80.1	37.9	25.7	45.7	20.1	13.9	28.7	75.8	37.5	24.0	31.8	29.9	30.3
(2*R*,3*S*,6*R*,7*R*,9*S*,10*R*)	38.8	55.7	79.1	41.3	25.5	45.5	19.3	20.1	23.3	77.6	42.5	25.0	32.7	27.5	25.8
(2*R*,3*S*,6*R*,7*S*,9*R*,10*R*)	35.1	52.1	78.9	42.3	25.8	54.6	17.7	20.7	29.6	78.1	41.2	28.5	26.2	26.9	19.7
(2*R*,3*S*,6*R*,7*S*,9*S*,10*R*)	36.2	47.2	79.8	40.7	26.3	47.4	17.9	20.1	28.3	79.1	37.3	23.5	25.5	26.1	23.2
(2*S*,3*R*,6*R*,7*R*,9*R*,10*R*)	35.5	44.4	80.7	39.3	29.2	45.0	26.0	9.9	30.5	76.9	36.1	28.0	31.4	25.6	27.5
(2*S*,3*R*,6*R*,7*R*,9*S*,10*R*)	37.4	56.4	76.1	40.4	23.6	46.6	17.3	24.1	23.6	77.4	41.1	20.5	29.9	27.2	29.5
(2*S*,3*R*,6*R*,7*S*,9*R*,10*R*)	40.9	53.0	78.1	40.4	23.0	53.8	16.2	19.2	30.8	77.4	41.0	22.9	34.5	27.1	17.9
(2*S*,3*R*,6*R*,7*S*,9*S*,10*R*)*	41.5	47.9	80.1	41.2	24.3	49.3	19.9	21.6	29.8	80.8	37.4	19.9	28.8	25.6	19.8
(2*S*,3*R*,6*S*,7*R*,9*R*,10*R*)	35.5	46.9	79.8	41.1	26.4	44.2	17.7	16.2	27.0	74.4	37.3	24.3	30.5	26.2	24.1
(2*S*,3*R*,6*S*,7*R*,9*S*,10*R*)	37.9	51.4	79.1	42.0	26.2	53.0	18.1	21.3	30.5	76.5	40.8	19.1	30.4	26.9	20.4
(2*S*,3*R*,6*S*,7*S*,9*R*,10*R*)	39.9	53.7	79.2	39.8	22.1	47.0	18.2	17.8	23.5	77.3	41.9	25.1	33.7	29.3	25.4
(2S,3*R*,6*S*,7*S*,9*S*,10*R*)	38.8	46.2	80.0	37.3	26.8	45.6	17.0	18.2	30.5	80.2	38.1	19.4	29.2	30.5	29.5
(2*S*,3*S*,6*R*,7*R*,9*R*,10*R*)	37.2	45.3	79.3	40.4	27.7	44.5	25.7	10.2	30.5	76.9	36.4	28.8	30.1	22.2	27.1
(2*S*,3*S*,6*R*,7*R*,9*S*,10*R*)	37.9	57.1	75.2	39.7	23.5	46.7	17.3	23.8	23.5	77.2	41.3	20.5	30.0	25.3	23.5
(2*S*,3*S*,6*R*,7*S*,9*R*,10*R*)	41.6	53.7	76.9	40.6	22.9	53.5	16.4	19.2	30.6	77.3	40.8	22.5	34.2	25.7	17.8
(2*S*,3*S*,6*R*,7*S*,9*S*,10*R*)	46.2	48.7	80.2	42.8	26.2	54.6	18.6	21.3	29.9	81.1	38.0	19.5	28.9	25.9	20.7
(2*S*,3*S*,6*S*,7*R*,9*R*,10*R*)	36.6	47.7	79.3	41.8	26.4	41.7	17.1	17.4	25.8	74.5	37.6	24.1	30.2	22.9	17.4
(2*S*,3*S*,6*S*,7*R*,9*S*,10*R*)	40.0	53.1	79.7	42.4	25.7	50.2	18.1	22.4	29.8	76.4	41.4	18.9	30.4	24.9	21.6
(2*S*,3*S*,6*S*,7*S*,9*R*,10*R*)	43.5	55.3	82.6	38.6	22.3	46.8	16.8	17.5	23.3	76.9	41.6	22.2	34.8	25.5	24.6
(2*S*,3*S*,6*S*,7*S*,9*S*,10*R*)	41.6	48.6	82.0	36.2	27.2	44.2	16.2	18.7	30.4	79.8	38.3	19.6	29.2	25.9	29.3
(2*R*,3*S*,6*S*,7*R*,9*R*,10*R*)	35.5	47.6	80.0	41.5	24.3	45.7	21.6	17.3	27.2	76.8	37.5	24.3	31.3	25.9	19.9
(2*R*,3*S*,6*S*,7*R*,9*S*,10*R*)	39.7	52.9	78.0	40.1	23.5	52.8	16.2	20.7	31.7	76.2	41.6	28.7	28.9	27.4	18.8
(2R,3S,6*S*,7*S*,9*S*,10*R*)	36.8	46.4	80.8	39.0	28.7	45.1	23.5	14.5	32.7	78.2	36.1	26.0	28.4	25.7	14.5
(2R,3S,6*S*,7*S*,9*R*,10*R*)	35.8	57.2	75.8	40.2	23.4	48.5	16.6	22.9	24.5	78.5	41.8	27.5	27.8	27.3	30.3

Calculated ^{1}H NMR chemical shift of **8** and its isomers.

	H-1	H-1	H-2	H-4	H-4	H-5	H-5	H-6	H-8	H-8	H-9	H-10	H-12	H-13	H-13	H-15
	1.45	1.19	2.61	1.60	1.70	1.80	1.70	1.42	0.45	0.74	0.55	3.14	0.97	1.02	1.27	0.99
(2R,3R,6R,7R,9R,10R)	1.88	1.07	2.02	1.53	1.67	2.17	1.43	3.12	0.36	0.57	0.72	3.91	1.25	1.09	1.13	1.19
(2R,3R,6R,7R,9S,10R)	1.69	1.15	2.19	1.64	1.75	1.61	0.97	3.12	0.58	0.76	0.86	3.48	1.10	1.15	1.13	1.12
(2R,3R,6R,7S,9R,10R)	2.19	1.19	2.37	1.60	1.79	1.72	1.64	1.96	0.84	0.91	0.42	4.06	1.01	1.03	1.16	1.37
(2R,3R,6R,7S,9S,10R)	1.64	1.45	1.87	1.60	1.63	1.72	1.71	1.74	0.23	0.56	0.77	3.00	1.11	1.08	1.12	0.98
(2R,3R,6S,7R,9R,10R)	1.80	1.36	2.09	1.66	1.52	1.75	1.52	2.05	0.53	0.47	0.62	3.87	1.14	1.12	1.08	1.11
(2R,3R,6S,7R,9S,10R)	1.73	1.35	2.17	1.83	1.75	1.63	1.44	1.30	0.87	0.66	0.60	3.42	1.13	1.11	1.03	1.01
(2R,3R,6S,7S,9R,10R)	2.02	1.32	1.58	1.74	1.73	1.37	1.03	1.90	1.00	1.10	0.53	4.09	1.08	1.02	1.07	1.41
(2R,3R,6S,7S,9S,10R)	1.52	1.33	1.14	1.57	1.54	1.78	1.60	2.04	0.54	0.71	0.89	3.68	1.15	0.96	1.03	1.05
(2R,3S,6R,7R,9R,10R)	1.92	1.37	2.09	1.66	1.55	2.23	1.31	2.26	0.31	0.63	0.78	3.89	1.10	1.23	1.23	1.14
(2R,3S,6R,7R,9S,10R)	1.88	1.60	2.13	1.46	1.42	1.38	1.30	2.31	0.76	0.95	1.21	3.34	1.19	1.15	1.16	1.09
(2R,3S,6R,7S,9R,10R)	2.43	0.89	2.11	1.74	1.57	2.20	1.48	1.78	0.89	0.94	0.38	4.08	1.04	0.99	1.11	1.49
(2R,3S,6R,7S,9S,10R)	1.85	1.16	1.70	1.67	1.48	2.25	1.65	1.67	0.25	0.59	0.77	3.01	1.12	1.06	1.12	1.15
(2S,3R,6R,7R,9R,10R)	1.60	1.39	1.86	1.57	1.52	2.17	1.59	2.72	0.26	0.80	0.93	4.00	1.16	1.12	1.07	0.26
(2S,3R,6R,7R,9S,10R)	1.58	1.52	1.12	1.72	1.71	1.53	0.80	2.42	1.01	1.10	0.40	3.52	1.00	1.18	1.06	1.14
(2S,3R,6R,7S,9R,10R)	1.74	1.20	2.00	1.83	1.82	1.60	1.44	1.86	0.70	0.99	0.56	4.30	1.16	1.16	1.11	1.27
(2S,3R,6R,7S,9S,10R)*	1.33	1.43	1.83	1.56	1.60	1.66	2.01	1.57	0.42	0.69	0.64	3.20	1.14	1.08	1.12	0.97
(2S,3R,6S,7R,9R,10R)	1.97	1.13	1.97	1.63	1.53	2.22	1.63	2.48	0.47	0.50	0.89	3.78	1.04	1.03	1.13	1.22
(2S,3R,6S,7R,9S,10R)	1.83	1.03	2.13	1.71	1.54	2.12	1.52	1.72	0.73	0.94	0.94	3.34	1.02	1.15	1.11	1.13
(2S,3R,6S,7S,9R,10R)	1.90	1.54	2.32	1.60	1.59	1.37	1.07	2.50	0.66	0.84	0.97	4.21	1.16	1.20	1.25	1.43
(2S,3R,6S,7S,9S,10R)	1.55	1.29	2.03	1.67	1.60	1.34	1.40	2.21	0.40	0.57	0.79	3.19	1.22	1.05	1.22	1.11
(2S,3S,6R,7R,9R,10R)	1.75	1.19	1.96	1.59	1.55	1.81	1.63	2.14	0.29	0.92	0.93	3.97	1.11	1.11	1.03	1.06
(2S,3S,6R,7R,9S,10R)	1.61	1.41	1.60	1.73	1.72	1.37	1.02	1.75	0.96	1.11	0.56	3.53	1.07	1.19	1.03	1.73
(2S,3S,6R,7S,9R,10R)	1.59	1.42	2.34	1.76	1.84	1.67	1.43	1.25	0.70	0.91	0.42	4.27	1.17	1.15	1.00	1.32
(2S,3S,6R,7S,9S,10R)	1.67	0.98	2.10	1.51	1.40	2.03	1.43	1.02	0.37	0.70	0.67	3.16	1.18	1.05	1.07	1.07
(2S,3S,6S,7R,9R,10R)	1.73	1.46	2.06	1.62	1.60	1.70	1.68	2.53	0.46	0.57	0.90	3.77	1.04	1.02	1.13	1.04
(2S,3S,6S,7R,9S,10R)	1.59	1.32	2.23	1.65	1.61	1.72	1.61	1.93	0.72	0.89	0.44	3.32	1.03	1.14	1.12	1.00
(2S,3S,6S,7S,9R,10R)	1.50	1.21	2.37	1.68	1.62	1.60	0.89	3.19	0.59	0.78	0.71	4.26	1.23	1.13	1.10	1.46
(2S,3S,6S,7S,9S,10R)	1.36	1.03	1.94	1.61	1.60	1.59	1.59	3.14	0.42	0.60	0.76	3.19	1.24	1.03	1.12	1.15
(2R,3S,6S,7R,9R,10R)	1.96	1.15	1.89	1.64	1.59	1.97	1.61	2.36	0.49	0.59	0.61	3.88	1.11	1.14	1.13	1.03
(2R,3S,6S,7R,9S,10R)	1.66	1.33	1.77	1.86	1.79	1.62	1.36	1.85	0.67	0.94	0.72	3.42	1.14	1.09	1.11	0.94
(2R,3S,6S,7S,9S,10R)	1.44	1.32	0.97	1.61	1.51	2.13	1.55	2.57	0.53	0.53	0.88	3.60	1.19	0.98	1.05	1.09
(2R,3S,6S,7S,9R,10R)	1.73	1.54	1.09	1.73	1.82	1.53	0.81	2.54	1.01	1.10	0.43	4.08	0.98	1.09	1.07	1.44

Statistical analysis of δ^{13}C and ^{1}H for **8** and its isomers.

	^{13}C			^{1}H			^{1}H+^{13}C	
	RMSD (ppm)	max dev (ppm)	DP4	RMSD (ppm)	max dev (ppm)	DP4	DP4	
(2*R*,3*R*,6*R*,7*R*,9*R*,10*R*)	4.8	10.8	0.0%	0.54	1.98	0.0%	0.0%	
(2*R*,3*R*,6*R*,7*R*,9*S*,10*R*)	4.4	9.8	0.0%	0.52	1.87	0.0%	0.0%	
(2*R*,3*R*,6*R*,7*S*,9*R*,10*R*)	3.1	6.9	0.0%	0.36	2.01	0.0%	0.0%	
(2*R*,3*R*,6*R*,7*S*,9*S*,10*R*)	2.9	4.7	0.0%	0.25	1.73	3.4%	0.0%	
(2*R*,3*R*,6*S*,7*R*,9*R*,10*R*)	3.1	5.6	0.0%	0.32	1.97	0.0%	0.0%	
(2*R*,3*R*,6*S*,7*R*,9*S*,10*R*)	3.6	9.4	0.0%	0.23	1.85	1.5%	0.0%	
(2*R*,3*R*,6*S*,7*S*,9*R*,10*R*)	5.3	10.8	0.0%	0.49	2.02	0.0%	0.0%	
(2*R*,3*R*,6*S*,7*S*,9*S*,10*R*)	4.5	8.1	0.0%	0.46	1.92	0.0%	0.0%	
(2*R*,3*S*,6*R*,7*R*,9*R*,10*R*)	5.2	10.9	0.0%	0.39	1.97	0.0%	0.0%	
(2*R*,3*S*,6*R*,7*R*,9*S*,10*R*)	4.2	7.5	0.0%	0.41	1.83	0.0%	0.0%	
(2*R*,3*S*,6*R*,7*S*,9*R*,10*R*)	3.9	9.4	0.0%	0.44	2.02	0.0%	0.0%	
(2*R*,3*S*,6*R*,7*S*,9*S*,10*R*)	2.7	5.9	0.0%	0.31	1.73	0.0%	0.0%	
(2*S*,3*R*,6*R*,7*R*,9*R*,10*R*)	5.7	12.7	0.0%	0.48	2.00	0.0%	0.0%	
(2*S*,3*R*,6*R*,7*R*,9*S*,10*R*)	4.4	10.1	0.0%	0.57	1.88	0.0%	0.0%	
(2*S*,3*R*,6*R*,7*S*,9*R*,10*R*)	3.2	5.8	0.0%	0.40	2.07	0.0%	0.0%	
(2*S*,3*R*,6*R*,7*S*,9*S*,10*R*)*	0.6	1.1	99.9%	0.24	1.79	30.7%	100.0%	proposed diastereomer
(2*S*,3*R*,6*S*,7*R*,9*R*,10*R*)	3.9	6.6	0.0%	0.42	1.95	0.0%	0.0%	
(2*S*,3*R*,6*S*,7*R*,9*S*,10*R*)	2.5	4.5	0.0%	0.26	1.83	0.0%	0.0%	
(2*S*,3*R*,6*S*,7*S*,9*R*,10*R*)	4.1	6.2	0.0%	0.49	2.05	0.0%	0.0%	
(2S,3*R*,6*S*,7*S*,9*S*,10*R*)	3.7	10.1	0.0%	0.31	1.79	0.0%	0.0%	
(2*S*,3*S*,6*R*,7*R*,9*R*,10*R*)	5.5	12.4	0.0%	0.37	1.99	0.0%	0.0%	
(2*S*,3*S*,6*R*,7*R*,9*S*,10*R*)	3.9	8.9	0.0%	0.41	1.88	0.0%	0.0%	
(2*S*,3*S*,6*R*,7*S*,9*R*,10*R*)	3.2	5.5	0.0%	0.35	2.07	0.0%	0.0%	
(2*S*,3*S*,6*R*,7*S*,9*S*,10*R*)	2.1	5.6	0.1%	0.24	1.78	1.4%	0.0%	
(2*S*,3*S*,6*S*,7*R*,9*R*,10*R*)	3.9	7.3	0.0%	0.39	1.94	0.0%	0.0%	
(2*S*,3*S*,6*S*,7*R*,9*S*,10*R*)	2.3	4.9	0.0%	0.21	1.82	63.0%	0.0%	
(2*S*,3*S*,6*S*,7*S*,9*R*,10*R*)	4.0	7.1	0.0%	0.59	2.06	0.0%	0.0%	
(2*S*,3*S*,6*S*,7*S*,9*S*,10*R*)	3.6	9.9	0.0%	0.49	1.79	0.0%	0.0%	
(2*R*,3*S*,6*S*,7*R*,9*R*,10*R*)	3.2	6.6	0.0%	0.40	1.97	0.0%	0.0%	
(2*R*,3*S*,6*S*,7*R*,9*S*,10*R*)	3.7	9.6	0.0%	0.31	1.85	0.0%	0.0%	
(2R,3S,6*S*,7*S*,9*S*,10*R*)	4.1	8.1	0.0%	0.56	1.90	0.0%	0.0%	
(2R,3S,6*S*,7*S*,9*R*,10*R*)	5.2	10.9	0.0%	0.64	2.02	0.0%	0.0%	

ECD calculations

- Sets of stable conformers obtained in the chemical shift calculations were used as the initial geometries.
- Geometries were further optimized with B3LYP/def2-TZVP and the ECD calculations were performed with the same level.
- Relative Gibbs free energy was calculated by summing the SCF energy and the chemical potential obtained by the vibrational analysis.
- The UV ECD spectra were build based on the oscillator strength (UV) and rotatory strength (ECD) using the normal distribution function in Microsoft Excel (NORM.DIST) for Office365.
- The widths were set to appropriately reproduce the spectra.
- The UV/ECD spectra of individual conformers were composited by Boltzmann averaging based on the free energy.
- The wavelengths UV spectra were corrected based on the experimental spectra, and those of ECD spectra were also corrected using identical parameters.
- The ECD spectra of **1a**, **1b**, **2,** and **4** were reversed by using negative magnitude for the intensities.

SCF energy (au), chemical potential (kJ/mol). relative free energy, and Boltzmann distribution of *ent*-**1a** based on B3LYP/def2-TZVP.

	M0001	M0002	M0003	M0004	M0005	M0006	M0007	M0008	M0009
SCF (au)	-736.2984287	-736.2980099	-736.298485	-736.2975939	-736.2980739	-736.2979987	-736.2978283	-736.297762	-736.2976151
CP (kJ/mol)	850.59	850.56	848.17	850.79	846.85	846.36	847.6	847.6	850.4
rel G (kJ/mol)	3.1	4.17	0.53	5.49	0.29	0	1.69	1.86	5.05
Boltzmann dist.	6.5%	4.2%	18.4%	2.5%	20.3%	22.8%	11.5%	10.8%	3.0%

Wavelength (nm) oscillator strength (UV), and rotatory strength (CD) of *ent*-**1a** based on B3LYP/def2-TZVP.

M0001			M0002			M0003			M0004			M0005		
λ	UV	CD	λ	UV	CD	λ	UV	CD	λ	UV	CD	λ	UV	CD
158.1	5.8.E-03	-20.1	159.2	2.1.E-03	-0.4	158.0	2.7.E-03	1.1	159.3	2.E-03	0.3	158.5	3.E-03	-0.2
158.9	1.4.E-02	19.3	159.9	5.0.E-03	-7.9	159.4	1.8.E-03	-2.7	159.5	2.E-02	-3.0	159.5	6.E-04	2.0
158.9	8.5.E-04	-3.1	160.5	1.2.E-03	-0.4	159.7	7.1.E-03	4.6	160.2	1.E-03	3.7	159.7	7.E-04	0.9
159.8	4.5.E-04	-0.1	160.6	1.6.E-03	0.8	161.9	2.7.E-03	-4.4	161.5	1.E-03	1.2	160.4	1.E-02	-7.8
161.6	1.2.E-03	1.5	161.1	4.4.E-03	-3.2	162.4	5.4.E-03	12.9	161.7	8.E-03	3.7	160.7	1.E-03	-2.2
163.0	3.6.E-03	2.8	162.1	3.0.E-02	15.2	164.7	3.2.E-03	-2.9	162.6	2.E-02	15.0	165.5	5.E-03	2.0
164.0	4.7.E-03	-4.7	165.9	1.0.E-03	5.0	167.1	7.2.E-03	1.4	165.8	5.E-03	-0.6	168.0	5.E-03	0.1
169.2	2.5.E-03	0.3	169.4	1.4.E-03	3.2	168.8	3.2.E-03	-2.4	168.7	3.E-03	-3.8	168.5	6.E-03	6.9
172.9	8.1.E-03	4.3	170.5	7.9.E-03	-5.3	168.9	2.5.E-03	-1.0	171.5	1.E-02	-6.4	171.7	1.E-02	-1.6
175.2	8.1.E-03	2.7	172.0	1.5.E-02	-0.8	170.8	7.0.E-03	-3.2	172.3	8.E-03	5.4	172.4	3.E-03	-2.0
177.1	6.0.E-03	-6.8	176.5	6.3.E-03	-6.3	175.4	8.1.E-03	0.4	176.2	5.E-03	-4.6	175.4	7.E-03	2.2
177.7	2.4.E-03	-2.9	176.8	5.5.E-03	0.8	176.5	1.0.E-02	-5.1	176.4	1.E-02	-3.0	176.7	1.E-02	-8.1
178.8	2.8.E-03	-15.6	180.4	1.5.E-02	5.1	179.3	1.5.E-02	5.9	179.0	2.E-02	9.7	179.9	5.E-03	8.5
181.0	1.8.E-02	2.4	182.7	3.5.E-03	-6.3	179.7	1.3.E-03	-6.6	182.2	4.E-03	-8.6	180.1	7.E-03	1.4
183.5	1.1.E-03	-2.4	183.8	4.6.E-03	1.6	183.1	1.2.E-02	4.7	183.9	5.E-03	3.3	182.9	1.E-02	3.3
185.9	1.1.E-02	-2.1	184.5	2.7.E-03	-14.5	184.4	2.4.E-03	-6.0	185.1	2.E-03	-1.4	184.2	3.E-03	-6.4
187.2	1.5.E-03	7.1	187.5	9.6.E-03	-3.7	189.4	6.2.E-03	-3.0	189.8	6.E-03	2.1	189.3	4.E-03	-1.2
189.9	3.5.E-03	-0.6	195.9	2.3.E-03	-0.8	194.1	2.5.E-04	0.0	195.3	4.E-03	7.1	194.4	1.E-04	-0.8
198.3	4.4.E-03	2.9	204.0	8.7.E-03	2.0	199.2	5.0.E-03	2.6	204.0	6.E-03	6.9	197.9	6.E-03	2.1
294.0	3.5.E-05	2.2	294.2	4.3.E-05	1.7	294.1	4.1.E-05	1.9	294.3	4.E-05	1.6	294.1	5.E-05	1.7

M0006			M0007			M0008			M0009		
λ	UV	CD	λ	UV	CD	λ	UV	CD	λ	UV	CD
158.3	2.6.E-03	2.0	158.3	2.6.E-03	2.0	158.5	1.6.E-04	-2.0	158.5	2.5.E-03	5.7
158.8	1.5.E-03	1.8	158.8	1.5.E-03	1.8	159.4	9.7.E-04	1.2	158.9	3.0.E-03	-0.3
160.3	1.0.E-02	-4.6	160.3	1.0.E-02	-4.6	160.4	5.0.E-03	-7.5	159.3	1.0.E-03	1.2
160.6	7.2.E-04	-3.2	160.6	7.2.E-04	-3.2	161.4	9.2.E-03	-5.0	160.4	1.2.E-02	-3.4
164.8	2.9.E-03	-7.7	164.8	2.9.E-03	-7.7	162.6	9.6.E-04	3.0	161.2	6.7.E-04	0.5
165.3	3.9.E-03	0.4	165.3	3.9.E-03	0.4	165.1	1.3.E-02	4.3	165.7	7.3.E-03	6.9
166.0	3.2.E-03	3.5	166.0	3.2.E-03	3.5	165.4	5.3.E-03	5.9	165.7	2.3.E-03	1.3
167.0	5.6.E-03	-1.7	167.0	5.6.E-03	-1.7	165.9	2.4.E-03	4.8	167.9	8.2.E-03	-8.4
173.5	1.7.E-02	6.7	173.5	1.7.E-02	6.7	169.7	4.7.E-03	-3.4	171.6	1.8.E-02	0.9
174.0	3.4.E-03	0.5	174.0	3.4.E-03	0.5	171.4	1.5.E-02	0.9	173.1	3.7.E-03	-0.5
177.2	9.6.E-03	-8.7	177.2	9.6.E-03	-8.7	176.3	5.9.E-03	-2.9	177.1	9.7.E-03	-7.0
177.8	2.1.E-03	-0.2	177.8	2.1.E-03	-0.2	177.5	2.1.E-03	-2.2	177.7	1.3.E-03	-3.4
179.9	1.8.E-03	2.1	179.9	1.8.E-03	2.1	179.6	1.5.E-02	1.2	179.2	1.5.E-02	2.1
180.5	1.2.E-02	-1.4	180.5	1.2.E-02	-1.4	183.8	8.6.E-03	11.4	182.3	4.2.E-03	-4.7
183.8	1.6.E-02	-3.0	183.8	1.6.E-02	-3.0	183.9	9.7.E-03	-6.6	183.1	1.1.E-02	-9.2
185.4	2.5.E-03	-2.6	185.4	2.5.E-03	-2.6	184.5	2.1.E-03	-0.3	183.6	3.0.E-03	-7.8
187.0	8.4.E-04	0.8	187.0	8.4.E-04	0.8	185.4	5.3.E-03	-0.2	186.3	2.2.E-03	1.9
188.8	8.7.E-04	3.0	188.8	8.7.E-04	3.0	196.8	3.3.E-04	2.5	196.8	5.7.E-04	1.2
198.8	7.3.E-03	2.1	198.8	7.3.E-03	2.1	202.7	6.1.E-03	4.1	200.5	6.8.E-03	2.7
294.0	4.6.E-05	1.5	294.0	4.6.E-05	1.5	294.1	4.2.E-05	1.7	294.3	3.7.E-05	1.4

Parameters used for ECD reproduction of *ent*-**1a.**

wavelength	3 nm
UV width	15 nm
UV intensity	40000
CD width	12 nm
CD intensity	-100 (because the enantiomer was applied for the calculations)

SCF energy (au), chemical potential (kJ/mol). relative free energy, and Boltzmann distribution of *ent*-**1b** based on B3LYP/def2-TZVP.

	M0001	M0002	M0003	M0004	M0005	M0006	M0007	M0008	M0009	M0010	M0014
SCF (au)	-811.516922	-811.516468	-811.516919	-811.516603	-811.515945	-811.516378	-811.5165	-811.516228	-811.515979	-811.515459	-811.515448
CP (kJ/mol)	856.71	857.12	856.45	856.59	856.49	855.38	855.66	855.6	856.5	855.84	855.52
rel. G (kJ/mol)	0	1.6	-0.25	0.72	2.35	0.1	0.06	0.71	2.27	2.97	2.68
Boltz. dist.	13.35%	6.98%	14.78%	9.98%	5.17%	12.82%	13.03%	10.01%	5.34%	4.01%	4.52%

Wavelength (nm) oscillator strength (UV), and rotatory strength (CD) of *ent*-**1b** based on B3LYP/def2-TZVP.

M0001			M0002			M0003			M0004			M0005		
λ	UV	CD	λ	UV	CD	λ	UV	CD	λ	UV	CD	λ	UV	CD
169.0	2.6.E-03	-1.8	169.3	5.1.E-03	3.0	168.0	4.5.E-03	-0.8	168.5	3.2.E-03	-3.8	167.9	3.E-03	-4.6
172.8	4.2.E-04	-4.0	171.5	7.9.E-04	-1.1	171.3	3.7.E-03	-6.2	171.5	3.5.E-03	-3.6	170.6	2.E-03	-3.9
174.6	5.5.E-03	6.7	172.4	2.7.E-03	4.1	171.8	2.6.E-03	-7.2	173.2	2.6.E-03	-8.0	172.8	5.E-03	-2.6
175.3	9.1.E-03	-8.8	173.8	2.3.E-03	-2.2	174.2	3.6.E-03	1.8	173.4	4.3.E-03	-1.6	174.6	9.E-03	-3.9
175.7	8.2.E-03	-4.2	175.3	3.3.E-03	-3.8	174.9	5.3.E-03	-5.4	175.0	6.9.E-03	3.9	175.6	4.E-03	-2.3
177.1	9.4.E-03	-1.4	177.2	2.8.E-02	-0.2	176.0	5.5.E-03	-2.9	176.1	3.0.E-02	-0.3	176.7	3.E-02	1.2
178.7	8.3.E-03	-25.4	178.2	2.7.E-03	-7.5	176.6	3.1.E-02	4.0	178.6	2.9.E-03	-9.8	178.4	1.E-03	-1.8
180.3	1.1.E-02	1.4	180.1	7.3.E-03	-9.8	179.8	1.7.E-03	-2.8	179.8	1.2.E-03	7.5	180.0	5.E-03	-8.2
181.8	2.4.E-04	-2.2	184.0	4.1.E-03	-10.4	180.0	3.8.E-03	-12.6	180.2	5.4.E-03	-7.9	185.1	4.E-03	15.4
185.5	1.9.E-03	5.4	184.9	8.2.E-03	-5.9	189.1	9.9.E-03	-4.5	188.5	1.1.E-02	-2.7	185.8	5.E-03	-9.9
186.6	9.2.E-03	-6.4	193.8	6.9.E-04	-2.6	191.7	1.6.E-03	2.2	190.7	8.6.E-04	1.7	193.1	3.E-03	-2.7
193.6	2.8.E-03	2.0	195.7	7.4.E-03	-6.2	193.4	4.8.E-05	-0.5	193.5	4.4.E-04	-1.4	196.9	5.E-03	2.1
201.6	1.1.E-03	-0.2	203.8	1.6.E-03	9.0	201.9	1.0.E-03	2.0	201.6	4.7.E-03	-2.5	202.9	2.E-03	16.3
203.2	8.7.E-03	23.6	205.1	1.1.E-02	17.2	203.6	9.1.E-03	20.6	203.4	5.5.E-03	25.1	206.1	9.E-03	13.2
293.8	1.6.E-05	0.4	294.2	2.2.E-05	-0.2	293.9	2.1.E-05	0.0	293.9	2.4.E-05	-0.3	294.3	2.E-05	-0.2

M0006			M0007			M0008			M0009			M0010		
λ	UV	CD	λ	UV	CD	λ	UV	CD	λ	UV	CD	λ	UV	CD
167.8	4.1.E-03	1.6	166.8	9.7.E-03	-9.8	166.7	2.3.E-03	-12.1	168.5	4.6.E-03	-12.7	170.7	4.7.E-03	1.3
172.0	1.2.E-03	-6.5	171.2	4.3.E-04	-4.6	170.7	2.6.E-03	0.9	171.2	7.0.E-04	-5.1	171.5	6.6.E-03	-0.3
174.5	2.8.E-03	-4.1	174.3	4.9.E-03	11.1	171.5	6.3.E-04	-2.2	173.5	9.1.E-03	-11.3	175.9	5.7.E-03	-16.7
175.2	7.8.E-03	-0.8	175.1	2.1.E-02	-6.6	174.4	1.6.E-02	-3.3	174.5	2.1.E-02	2.0	176.3	3.6.E-03	-7.5
176.1	3.3.E-02	-0.5	175.3	1.0.E-03	-2.7	175.0	2.0.E-03	-4.0	175.2	8.8.E-03	-4.6	176.8	6.9.E-03	-8.7
177.1	5.1.E-03	-1.4	176.6	2.1.E-02	-5.3	177.3	5.9.E-03	5.4	177.3	1.1.E-02	1.8	178.5	9.6.E-03	-24.7
179.5	3.6.E-04	5.3	178.4	3.9.E-03	-5.2	177.6	1.8.E-02	-10.6	179.5	5.5.E-03	-11.5	179.3	1.2.E-02	4.3
180.0	6.8.E-03	-19.0	180.2	2.7.E-03	-10.3	179.6	4.3.E-03	-10.7	180.4	1.5.E-03	-5.1	181.9	4.3.E-03	12.3
180.7	1.9.E-03	-5.3	180.6	8.5.E-04	4.3	184.2	5.1.E-03	17.2	182.7	3.4.E-03	-7.5	183.1	3.0.E-03	-6.8
185.5	5.4.E-03	-0.4	186.1	7.1.E-03	-9.3	185.2	8.3.E-03	-11.3	183.6	7.9.E-03	-7.3	184.7	1.6.E-03	7.5
188.8	7.8.E-04	1.7	188.4	4.5.E-04	-0.6	193.6	4.7.E-03	3.7	192.6	3.4.E-03	3.1	185.9	8.2.E-03	-2.9
191.4	5.4.E-03	1.2	193.0	4.4.E-03	4.4	196.1	1.8.E-04	-0.4	195.7	1.1.E-03	-3.0	193.0	1.3.E-03	-1.5
201.8	3.5.E-03	-3.8	201.7	1.5.E-03	7.8	203.0	1.8.E-03	9.4	202.7	2.4.E-03	-1.6	199.7	6.4.E-03	5.7
203.2	7.7.E-03	27.3	204.1	9.6.E-03	16.4	204.8	8.8.E-03	15.9	203.9	8.5.E-03	25.0	200.7	1.0.E-03	-0.6
293.9	2.5.E-05	-0.5	293.8	2.3.E-05	-0.1	293.9	2.2.E-05	-0.2	294.2	2.0.E-05	-0.5	293.7	4.9.E-05	3.1

M0014		
λ	UV	CD
168.4	2.5.E-03	-2.9
168.9	6.9.E-03	-0.7
173.4	7.1.E-03	2.6
175.7	5.5.E-03	-6.4
176.0	5.7.E-03	-23.6
177.7	7.7.E-03	-15.1
178.5	1.8.E-02	-0.9
179.6	1.7.E-03	-3.4
180.2	4.4.E-03	10.2
186.3	7.9.E-03	-1.4
192.2	1.7.E-03	-3.5
193.2	2.1.E-04	0.8
200.6	7.4.E-03	4.2
201.0	9.6.E-04	1.0
293.7	5.6.E-05	2.6

Parameters used for ECD reproduction of *ent*-**1b.**

wavelength	-7 nm
UV width	15 nm
UV intensity	40000
CD width	12 nm
CD intensity	-50 (because the enantiomer was applied for the calculations)

SCF energy (au), chemical potential (kJ/mol). relative free energy, and Boltzmann distribution of **1c** based on B3LYP/def2-TZVP.

	M0001	M0002	M0003	M0004	M0005	M0006	M0007	M0008	M0009	M0010
SCF (au)	-736.26236	-736.26196	-736.26213	-736.26209	-736.2616	-736.26158	-736.26177	-736.26186	-736.26132	-736.26122
CP (kJ/mol)	840.16	841.15	839.38	840.48	840.29	841.16	839.64	840.49	840.46	840.37
Boltz. Dist.	12.6%	5.6%	13.6%	8.3%	5.4%	3.7%	8.4%	6.6%	3.7%	3.5%

	M0011	M0012	M0013	M0014	M0015	M0016	M0017	M0018	M0019
SCF (au)	-736.26089	-736.26113	-736.26051	-736.26133	-736.26057	-736.26091	-736.26059	-736.26011	-736.26039
CP (kJ/mol)	839.63	838.83	840.43	839.1	839.67	840.08	838.9	839.76	839.98
Boltz. Dist.	3.3%	5.9%	1.6%	6.5%	2.3%	2.8%	3.2%	1.4%	1.7%

Wavelength (nm) oscillator strength (UV), and rotatory strength (CD) of **1c** based on B3LYP/def2-TZVP.

M0001			M0002			M0003			M0004			M0005		
λ	UV	CD	λ	UV	CD	λ	UV	CD	λ	UV	CD	λ	UV	CD
165.3	1.4.E-02	-37.4	165.7	4.3.E-03	-6.0	165.6	1.2.E-02	-6.9	165.1	2.3.E-04	-2.3	165.8	4.6.E-03	1.1
166.3	3.6.E-03	4.2	168.3	7.2.E-03	-4.9	165.8	8.6.E-03	-22.7	166.1	1.3.E-03	-6.9	168.7	7.7.E-03	-1.5
169.9	1.5.E-02	5.2	170.0	1.2.E-02	-4.8	170.2	1.6.E-02	8.8	170.0	1.2.E-02	1.4	169.9	5.8.E-03	-2.1
173.3	4.5.E-03	2.2	172.8	4.6.E-04	1.3	172.0	5.1.E-03	0.9	174.4	6.8.E-02	28.4	170.7	1.1.E-02	-4.5
174.2	1.1.E-03	-4.1	173.9	1.4.E-02	0.8	173.9	1.9.E-02	33.1	174.8	4.2.E-03	0.5	172.9	3.6.E-03	8.0
175.3	4.3.E-02	15.8	175.8	3.8.E-02	-16.5	174.4	3.8.E-02	7.7	175.8	3.2.E-03	7.4	176.1	3.6.E-02	-19.8
176.1	4.2.E-02	33.0	177.3	8.1.E-03	0.2	176.1	1.5.E-02	-5.0	176.6	4.2.E-03	5.5	178.2	5.1.E-02	36.1
178.0	4.9.E-02	51.7	179.1	8.6.E-02	108.3	180.2	4.0.E-02	49.1	178.9	5.2.E-02	31.4	181.6	3.3.E-02	47.2
181.0	5.6.E-03	7.5	180.8	4.8.E-03	5.4	182.0	5.9.E-04	5.6	180.0	4.5.E-03	0.4	182.2	3.3.E-03	6.6
183.9	1.4.E-02	3.1	184.2	1.5.E-03	-10.4	183.9	1.0.E-02	-0.4	183.3	3.5.E-02	30.9	184.6	3.8.E-03	-7.8
188.0	2.9.E-03	-8.2	188.7	1.7.E-02	-9.7	188.0	6.1.E-03	1.0	187.8	2.9.E-03	-9.6	188.3	1.6.E-02	17.3
193.4	5.1.E-02	1.9	192.6	7.9.E-02	35.5	190.8	1.1.E-01	22.0	196.7	1.6.E-02	-4.9	191.8	1.1.E-01	17.0
199.5	9.4.E-02	36.4	197.1	2.7.E-02	-13.5	200.3	4.9.E-02	27.1	199.1	9.6.E-02	44.3	195.9	3.4.E-02	-5.5
206.5	1.3.E-01	-81.6	202.7	6.1.E-02	-41.3	206.8	8.6.E-02	-72.5	206.6	1.5.E-01	-76.1	203.3	4.6.E-02	-32.7
207.3	9.6.E-03	10.4	206.8	9.7.E-02	-3.8	208.6	5.7.E-02	-2.7	208.4	4.4.E-03	1.4	209.2	8.7.E-02	-14.5

M0006			M0007			M0008			M0009			M0010		
λ	UV	CD	λ	UV	CD	λ	UV	CD	λ	UV	CD	λ	UV	CD
166.0	4.0.E-03	6.5	165.7	1.2.E-02	4.4	164.8	9.7.E-03	-11.6	165.8	9.7.E-03	4.1	165.6	5.5.E-03	2.0
170.0	2.2.E-02	-2.0	166.5	1.3.E-02	-24.4	165.4	9.0.E-03	-41.8	168.2	9.0.E-03	-1.3	170.5	2.6.E-02	1.3
171.0	4.7.E-03	-7.4	168.7	1.2.E-02	7.1	171.4	1.6.E-02	0.7	171.4	1.6.E-02	-3.8	171.5	2.7.E-03	0.2
173.0	3.4.E-04	0.2	173.2	3.1.E-02	33.7	173.5	2.5.E-03	-0.4	171.8	2.5.E-03	-1.3	172.1	2.2.E-03	-6.6
174.2	1.4.E-02	-5.2	173.9	1.3.E-03	-4.5	174.5	8.2.E-04	2.2	173.2	8.2.E-04	15.6	173.1	2.7.E-03	5.1
175.4	2.7.E-02	-2.8	174.0	6.2.E-04	-5.6	174.9	5.1.E-02	14.7	176.1	5.1.E-02	7.5	175.5	2.4.E-02	-21.1
177.7	4.6.E-02	69.5	176.9	4.0.E-02	7.5	178.3	7.2.E-02	73.9	177.6	7.2.E-02	-10.4	179.3	9.6.E-02	94.0
179.8	1.2.E-03	-9.5	180.3	3.6.E-02	43.5	180.6	1.4.E-02	6.3	181.8	1.4.E-02	62.5	181.3	2.2.E-04	-2.7
181.5	2.4.E-02	-1.7	183.9	1.1.E-02	0.8	181.1	1.8.E-03	-0.4	184.1	1.8.E-03	-5.5	182.8	1.0.E-02	16.0
184.5	3.2.E-03	-6.7	187.2	1.1.E-01	48.0	181.6	2.7.E-02	23.0	186.4	2.7.E-02	29.4	184.8	3.4.E-03	-8.1
187.6	8.3.E-02	22.3	188.5	1.2.E-02	-10.2	187.7	2.0.E-03	-6.9	188.3	2.0.E-03	-12.7	187.2	4.7.E-02	-6.7
192.9	3.5.E-02	32.6	188.7	1.4.E-02	-17.4	194.5	5.3.E-02	-4.3	190.8	5.3.E-02	-18.7	192.6	4.6.E-02	29.0
199.8	8.1.E-03	-7.6	201.8	4.6.E-02	20.9	200.0	4.8.E-02	33.9	196.1	4.8.E-02	17.0	198.0	3.7.E-02	-18.1
203.7	9.2.E-02	-56.9	206.2	4.1.E-02	-43.8	207.0	1.2.E-01	-85.4	202.4	1.2.E-01	-23.6	204.1	6.6.E-02	-37.5
207.2	9.1.E-02	6.2	212.1	9.7.E-02	-27.5	209.3	4.7.E-02	10.2	213.1	4.7.E-02	-24.5	210.0	9.0.E-02	-5.0

M0011			M0012			M0013			M0014			M0015		
λ	UV	CD	λ	UV	CD	λ	UV	CD	λ	UV	CD	λ	UV	CD
166.8	0.0	2.8	166.5	0.0	12.7	167.2	0.0	-7.0	165.3	0.0	-2.2	166.2	0.0	-2.2
167.2	0.0	0.6	167.5	0.0	-0.4	167.5	0.0	10.4	166.2	0.0	-36.4	168.5	0.0	20.4
167.4	0.0	5.7	169.7	0.0	-4.6	169.8	0.0	-2.6	169.4	0.0	4.8	168.8	0.0	2.4
170.4	0.0	4.2	170.1	0.0	18.1	175.5	0.0	1.1	173.5	0.1	33.9	170.7	0.0	1.8
176.4	0.1	45.2	172.1	0.0	7.1	175.8	0.0	-0.8	174.7	0.0	10.8	175.3	0.0	-5.8
177.0	0.0	3.3	175.7	0.0	-4.7	177.2	0.0	-3.5	176.6	0.0	-4.4	176.0	0.0	-12.6
177.6	0.0	-0.3	177.4	0.0	-4.5	177.8	0.0	4.2	177.3	0.0	0.4	179.3	0.1	32.0
179.1	0.0	3.7	180.6	0.0	12.1	178.6	0.0	33.7	178.8	0.0	-0.7	180.7	0.0	5.3
187.0	0.0	14.3	181.4	0.0	17.6	185.6	0.0	-8.4	180.4	0.1	57.8	183.1	0.0	3.6
187.8	0.0	-8.3	187.7	0.0	-6.9	187.1	0.0	-7.2	187.4	0.0	10.6	185.6	0.0	-13.8
189.7	0.0	10.9	188.9	0.0	20.0	188.5	0.0	29.0	188.2	0.0	-3.1	190.8	0.0	59.2
192.3	0.1	-3.0	194.6	0.1	24.3	192.9	0.1	21.6	192.5	0.1	-18.4	191.8	0.0	3.0
200.9	0.1	30.1	200.2	0.0	24.7	197.0	0.0	-3.5	200.6	0.1	36.6	198.9	0.1	-26.8
207.1	0.1	-78.7	206.6	0.1	-83.3	203.2	0.1	-57.9	206.5	0.1	-72.1	204.1	0.1	-34.0
215.3	0.0	20.7	209.2	0.0	14.9	215.7	0.0	18.3	211.6	0.1	18.0	210.1	0.1	8.4

M0016			M0017			M0018			M019		
λ	UV	CD	λ	UV	CD	λ	UV	CD	λ	UV	CD
165.5	0.0	-5.1	166.9	0.0	-0.8	166.0	0.0	10.6	165.5	0.0	0.0
168.5	0.0	0.8	167.6	0.0	-1.1	167.2	0.0	8.9	166.4	0.0	0.0
171.4	0.0	-9.2	167.9	0.0	1.3	168.6	0.0	12.2	170.3	0.0	0.0
173.6	0.0	11.2	171.2	0.0	13.7	169.2	0.0	3.8	172.0	0.0	0.0
174.8	0.1	30.6	171.3	0.0	6.0	173.0	0.0	6.0	174.7	0.0	0.0
175.3	0.0	-9.9	174.1	0.0	0.1	177.3	0.0	-2.2	175.0	0.0	0.0
177.3	0.0	-6.2	177.8	0.0	5.4	178.2	0.0	-0.7	176.1	0.1	0.1
180.4	0.0	14.0	180.1	0.0	20.0	180.8	0.0	21.4	180.3	0.0	0.0
183.9	0.0	29.7	186.0	0.1	52.1	185.0	0.0	-8.1	181.3	0.1	0.1
184.8	0.0	0.5	187.4	0.0	-1.7	186.6	0.1	63.6	182.6	0.0	0.0
186.7	0.0	13.7	188.6	0.0	-9.5	189.1	0.0	12.3	183.4	0.0	0.0
193.2	0.1	12.6	189.6	0.0	8.2	191.1	0.1	-38.8	192.9	0.1	0.1
196.6	0.0	-0.1	202.2	0.1	20.0	196.7	0.0	21.9	195.8	0.0	0.0
202.4	0.1	-54.0	205.7	0.0	-30.7	202.4	0.0	-20.6	202.8	0.1	0.1
211.7	0.1	15.8	215.1	0.1	-33.5	215.9	0.1	-26.0	205.3	0.1	0.1

Parameters used for ECD reproduction of **1c.**

wavelength	-0 nm
UV width	8 nm
UV intensity	40000
CD width	6 nm
CD intensity	+10

SCF energy (au), chemical potential (kJ/mol). relative free energy, and Boltzmann distribution of **2a** based on B3LYP/def2-TZVP

	M0001	M0002	M0003
SCF (au)	-809.1107141	-809.1107323	-809.1107375
CP (kJ/mol)	728.38	728.04	728.47
rel G (kJ/mol)	0.00	-0.39	0.03
Boltzmann dist.	31.7%	37.0%	31.3%

Wavelength (nm) oscillator strength (UV), and rotatory strength (CD) of **2a** based on B3LYP/def2-TZVP.

M0001				M0002				M0003		
λ	UV	CD	λ	UV	CD	λ	UV	CD	UV	
173.6	1.3.E-02	173.6	-17.1	172.8	6.3.E-03	172.8	-12.0	172.8	6.4.E-03	172.8
173.9	6.6.E-03	173.9	2.9	174.1	3.5.E-03	174.1	1.7	174.1	3.3.E-03	174.1
174.1	1.5.E-03	174.1	-2.3	174.9	5.2.E-03	174.9	-9.1	174.9	5.5.E-03	174.9
175.3	3.6.E-03	175.3	-5.8	175.2	4.2.E-03	175.2	4.5	175.1	4.1.E-03	175.1
177.2	3.6.E-03	177.2	7.2	177.2	6.5.E-03	177.2	8.5	177.2	6.5.E-03	177.2
178.0	3.3.E-03	178.0	-6.1	177.9	1.8.E-03	177.9	-6.5	177.9	1.7.E-03	177.9
179.2	7.0.E-03	179.2	-4.0	178.7	7.7.E-03	178.7	-4.8	178.7	7.6.E-03	178.7
183.6	5.9.E-03	183.6	4.3	184.1	6.7.E-03	184.1	-2.8	184.1	6.6.E-03	184.1
186.7	2.9.E-03	186.7	15.4	187.5	3.0.E-03	187.5	6.7	187.5	3.0.E-03	187.5
188.4	2.5.E-03	188.4	1.1	187.8	3.6.E-03	187.8	10.9	187.8	3.6.E-03	187.8
193.5	3.9.E-03	193.5	3.2	193.0	5.1.E-03	193.0	6.1	192.9	5.1.E-03	192.9
200.9	1.0.E-03	200.9	1.1	200.9	2.4.E-04	200.9	-0.1	200.9	2.3.E-04	200.9
201.1	1.6.E-02	201.1	-2.0	201.2	1.5.E-02	201.2	-1.8	201.2	1.5.E-02	201.2
207.2	7.1.E-04	207.2	-1.2	206.7	1.2.E-05	206.7	0.0	206.7	1.4.E-05	206.7
211.9	7.8.E-03	211.9	-1.5	210.5	5.3.E-03	210.5	2.0	210.4	5.0.E-03	210.4
215.3	3.2.E-03	215.3	4.4	215.6	1.7.E-03	215.6	6.4	215.6	1.7.E-03	215.6
217.0	5.3.E-03	217.0	-18.8	218.0	1.1.E-02	218.0	-15.9	218.1	1.2.E-02	218.1
221.6	4.1.E-02	221.6	-34.6	221.2	3.8.E-02	221.2	-34.7	221.2	3.8.E-02	221.2
245.4	2.0.E-01	245.4	32.0	245.7	2.1.E-01	245.7	40.4	245.7	2.1.E-01	245.7
346.8	6.3.E-04	346.8	-13.7	346.0	6.1.E-04	346.0	-13.5	345.9	6.1.E-04	345.9

Parameters used for ECD reproduction of **2a.**

wavelength	7 nm
UV width	12 nm
UV intensity	6000
CD width	10 nm
CD intensity	+0.63

SCF energy (au), chemical potential (kJ/mol). relative free energy, and Boltzmann distribution of **2b** based on B3LYP/def2-TZVP

	M0001	M0002	M0003	M0004	M0005	M0006	M0007	M0008	M0009
SCF (au)	-735.0551692	-735.0549887	-735.0548794	-735.0546431	-735.0543656	-735.0542393	-735.0532329	-735.0537391	-735.0527031
CP (kJ/mol)	780.16	779.47	780.71	779.5	779.72	780.07	780.61	779.62	778.11
rel G (kJ/mol)	0.00	-0.22	1.31	0.72	1.67	2.35	5.53	3.21	4.42
Boltzmann dist.	20.54%	22.42%	12.09%	15.34%	10.45%	7.93%	2.19%	5.60%	3.43%

Wavelength (nm) oscillator strength (UV), and rotatory strength (CD) of **2b** based on B3LYP/def2-TZVP.

M0001			M0002			M0003			M0004			M0005		
λ	UV	CD	λ	UV	CD	λ	UV	CD	λ	UV	CD	λ	UV	CD
168.9	9.3.E-03	-12.5	168.6	1.7.E-02	-6.2	168.6	1.2.E-02	-4.8	169.6	1.3.E-02	-1.1	169.0	4.3.E-03	-4.8
170.3	4.2.E-03	-1.9	170.9	4.7.E-03	-5.9	170.1	6.4.E-03	-4.9	170.0	4.5.E-03	1.4	170.8	4.0.E-03	0.6
174.2	1.9.E-03	-3.1	174.9	4.4.E-03	3.0	171.7	3.4.E-03	-8.2	172.9	5.3.E-03	-11.0	174.1	3.5.E-03	0.5
175.1	7.9.E-03	15.2	175.4	2.8.E-03	-5.4	175.1	7.8.E-03	6.4	175.3	5.8.E-03	-0.8	174.4	6.2.E-03	0.6
176.2	4.3.E-03	-6.8	177.5	9.6.E-03	7.9	175.7	1.3.E-03	5.9	178.4	7.3.E-03	11.0	175.9	4.5.E-04	-2.8
178.3	2.4.E-03	-4.5	178.2	1.6.E-03	-7.8	178.1	1.1.E-02	-4.8	179.0	4.2.E-03	-11.1	177.4	6.6.E-03	6.0
179.1	3.3.E-03	3.3	179.9	7.6.E-03	-9.4	179.5	4.5.E-03	-4.1	179.9	1.0.E-02	-2.9	178.2	1.3.E-03	-8.3
180.7	1.4.E-03	-8.0	181.5	1.2.E-03	5.6	180.4	9.2.E-04	4.8	181.5	1.6.E-04	0.1	179.4	9.5.E-03	-9.9
182.1	5.6.E-03	10.0	183.3	7.7.E-03	5.2	182.8	8.8.E-03	1.0	182.2	1.4.E-03	-1.3	182.5	5.2.E-03	0.6
183.5	3.4.E-03	3.4	184.1	2.8.E-03	4.8	184.2	3.1.E-03	-3.1	184.8	3.1.E-03	0.2	184.3	5.1.E-03	11.2
184.2	5.3.E-03	-8.3	188.1	2.1.E-03	1.7	186.2	3.0.E-04	-1.3	190.1	3.1.E-03	-2.3	187.3	3.0.E-03	13.0
189.5	3.7.E-03	0.1	190.5	4.1.E-03	1.8	189.7	4.9.E-03	7.5	191.1	6.3.E-03	-1.5	194.5	6.3.E-03	1.6
193.5	2.4.E-03	4.6	194.5	2.4.E-03	7.9	190.1	3.4.E-03	9.2	192.1	4.1.E-03	17.5	195.5	3.9.E-03	13.3
194.6	6.5.E-03	11.0	195.3	7.2.E-03	1.7	194.9	4.4.E-03	3.6	195.9	4.8.E-03	1.7	199.2	4.0.E-03	-2.4
202.3	1.5.E-02	-2.1	203.1	1.5.E-02	-4.6	202.3	1.7.E-02	-3.1	203.1	1.8.E-02	-3.2	201.2	1.5.E-02	-1.3
212.2	4.7.E-03	3.2	213.9	8.0.E-03	3.8	213.6	5.4.E-03	3.5	212.1	4.0.E-03	1.3	213.4	5.3.E-03	2.6
220.5	4.6.E-02	-49.0	218.5	8.0.E-03	-9.2	219.7	4.0.E-02	-41.8	220.5	2.0.E-02	-17.0	221.7	5.0.E-02	-50.4
226.2	5.2.E-03	-0.6	224.0	4.0.E-02	-35.2	226.5	1.2.E-02	-6.6	224.0	3.3.E-02	-26.9	228.4	2.4.E-03	-0.6
246.2	2.0.E-01	36.8	246.7	2.0.E-01	35.7	246.2	2.0.E-01	38.5	246.8	2.0.E-01	38.8	246.1	2.0.E-01	38.5
346.4	6.0.E-04	-13.4	346.3	6.0.E-04	-13.5	346.9	6.0.E-04	-13.5	346.8	5.9.E-04	-13.5	347.0	6.1.E-04	-13.6

M0006			M0007			M0008			M0009		
λ	UV	CD	λ	UV	CD	λ	UV	CD	λ	UV	CD
167.7	1.3.E-02	9.1	167.7	1.1.E-02	2.2	168.6	4.5.E-03	-0.2	168.3	9.5.E-03	2.0
169.7	2.1.E-03	8.8	169.8	1.4.E-03	4.3	170.6	1.4.E-03	4.3	169.7	2.6.E-03	7.5
170.8	9.9.E-03	-17.7	170.4	1.3.E-03	-4.9	175.7	7.2.E-03	-10.6	170.7	1.9.E-03	-3.0
174.2	4.5.E-03	2.3	171.8	1.3.E-02	-11.4	177.6	7.7.E-03	-5.2	175.0	1.5.E-02	-19.5
175.1	5.7.E-03	-1.5	176.7	5.6.E-03	-7.0	178.6	7.6.E-03	-2.7	177.0	3.0.E-03	-8.9
177.8	5.9.E-03	2.4	177.3	1.1.E-02	-8.2	179.8	2.6.E-03	-2.4	178.5	1.1.E-02	-4.7
179.0	7.3.E-03	-7.4	179.3	5.3.E-03	-2.1	181.0	3.6.E-04	-1.2	180.1	4.6.E-03	-1.5
179.7	5.9.E-04	-1.2	180.9	9.9.E-04	8.1	181.1	9.4.E-03	-5.0	180.8	9.0.E-04	5.0
183.0	4.4.E-03	1.3	182.4	1.7.E-03	1.8	181.5	2.1.E-03	-1.5	183.1	1.6.E-03	1.6
186.0	3.2.E-03	4.2	186.6	2.8.E-03	6.0	185.7	9.4.E-04	-7.3	186.3	3.6.E-03	4.5
186.7	2.7.E-03	-15.0	187.5	4.2.E-03	15.8	186.5	7.5.E-03	15.5	187.9	3.1.E-03	-6.7
193.7	6.9.E-03	-0.4	192.7	6.0.E-03	19.0	191.3	4.2.E-03	-3.9	190.1	5.4.E-03	21.2
194.7	5.1.E-03	18.5	196.7	3.8.E-03	-1.5	192.7	5.3.E-03	10.9	197.6	5.8.E-03	-7.2
199.5	4.8.E-03	-0.9	201.7	3.2.E-03	-2.1	196.0	3.3.E-03	2.1	201.6	2.6.E-03	-3.1
201.4	1.7.E-02	-5.4	204.3	1.3.E-02	-5.7	202.1	9.9.E-03	-4.1	204.5	1.5.E-02	0.7
213.7	8.6.E-03	1.9	215.3	1.3.E-02	0.8	211.7	1.3.E-03	-2.7	214.4	7.1.E-03	3.1
221.0	4.3.E-02	-44.3	222.2	4.6.E-02	-45.0	220.9	8.8.E-03	8.8	222.5	5.7.E-02	-50.7
229.3	1.4.E-03	-0.9	226.7	6.9.E-03	-1.0	223.9	5.1.E-02	-46.7	225.9	1.0.E-02	-3.3
246.2	2.0.E-01	38.5	247.6	1.9.E-01	39.5	247.3	2.0.E-01	38.9	247.5	1.8.E-01	45.0
346.6	5.9.E-04	-13.5	346.0	6.1.E-04	-13.5	346.0	6.2.E-04	-13.7	347.1	6.1.E-04	-13.8

Parameters used for ECD reproduction of **2b.**

wavelength	8 nm
UV width	12 nm
UV intensity	6000
CD width	15 nm
CD intensity	+1.1

SCF energy (au), chemical potential (kJ/mol). relative free energy, and Boltzmann distribution of **3** based on BHLYP/def2-TZVP.

	M0001	M0002	M0003	M0004	M0005	M0006	M0007	M0008	M0009	M0010
SCF (au)	-811.47736	-811.47722	-811.47736	-811.47676	-811.47714	-811.4766	-811.47678	-811.47627	-811.47601	-811.47613
CP (kJ/mol)	891.24	890.98	890.73	890.84	890.47	890.68	889.67	890.35	891.25	890.76
rel G (kJ/mol)	0.0	0.4	0.0	1.6	0.6	2.0	1.5	2.8	3.5	3.2
Boltzmann dist	16.6%	14.4%	16.7%	8.8%	13.3%	7.5%	9.0%	5.2%	4.0%	4.5%

Wavelength (nm) oscillator strength (UV), and rotatory strength (CD) of **3** based on BHLYP/def2-TZVP.

M0001			M0002			M0003			M0004			M0005		
λ	UV	CD	λ	UV	CD	λ	UV	CD	λ	UV	CD	λ	UV	CD
144.5	7.4.E-03	5.8	144.7	9.4.E-03	-3.6	144.8	1.3.E-03	-2.5	145.9	1.5.E-03	-1.2	145.1	3.3.E-04	-0.5
146.9	8.1.E-03	6.0	147.1	1.3.E-02	-16.5	147.2	1.3.E-02	5.4	147.1	8.4.E-03	11.2	147.4	1.5.E-02	-16.9
147.6	1.5.E-02	0.8	147.5	1.6.E-02	5.5	148.3	8.8.E-03	-3.2	149.0	2.3.E-02	9.0	148.4	2.2.E-02	5.5
149.3	1.1.E-02	14.2	148.5	1.8.E-02	16.3	149.4	1.0.E-02	8.2	149.4	7.8.E-04	-3.3	148.5	7.3.E-03	6.1
150.6	4.9.E-03	-6.0	150.4	1.6.E-03	4.2	150.7	1.2.E-02	-14.6	151.2	2.0.E-02	-15.7	150.3	2.4.E-03	5.0
151.6	1.2.E-02	-12.0	151.5	1.6.E-02	-11.7	151.7	7.5.E-03	-5.2	152.5	3.1.E-03	-3.2	151.5	1.4.E-02	-8.3
156.5	1.9.E-02	-21.5	156.6	1.8.E-02	-16.2	155.4	1.5.E-02	-23.3	155.2	1.7.E-02	-22.4	155.4	1.8.E-02	-23.6
158.9	8.4.E-03	2.4	158.6	9.3.E-03	-5.0	159.8	8.2.E-03	7.2	159.7	7.0.E-03	12.2	159.9	8.4.E-03	4.0
160.4	3.0.E-03	3.7	160.2	2.3.E-03	9.8	160.7	8.8.E-03	17.8	160.6	9.1.E-03	19.9	160.7	8.6.E-03	19.2
160.6	6.2.E-03	5.7	160.6	7.1.E-03	16.0	162.9	1.3.E-03	7.3	164.1	2.7.E-02	35.5	163.1	6.3.E-04	-1.6
163.9	3.2.E-02	7.4	164.0	3.0.E-02	6.0	163.9	3.5.E-02	-1.3	164.4	1.1.E-02	-29.3	164.1	3.2.E-02	-1.9
166.1	1.3.E-02	-33.3	166.1	1.4.E-02	-34.4	166.1	1.4.E-02	-35.4	166.0	1.4.E-02	-36.8	166.2	1.5.E-02	-36.2
171.5	2.9.E-02	-6.4	172.1	2.8.E-02	-5.2	172.5	2.7.E-02	-2.9	174.4	2.5.E-02	3.0	173.0	2.8.E-02	-2.4
177.4	3.5.E-03	-0.8	177.8	3.1.E-03	-1.8	177.6	3.5.E-03	-0.8	177.6	2.7.E-03	-1.8	177.9	3.1.E-03	-2.1
192.7	2.4.E-01	20.3	192.7	2.4.E-01	23.7	192.8	2.4.E-01	19.1	192.6	2.4.E-01	19.5	192.8	2.4.E-01	23.0

M0006			M0007			M0008			M0009			M0010		
λ	UV	CD	λ	UV	CD	λ	UV	CD	λ	UV	CD	λ	UV	CD
145.7	1.6.E-03	-4.6	144.3	3.E-03	7.9	144.5	2.E-02	-13.6	144.3	4.E-03	14.6	144.6	8.E-03	13.2
146.9	6.2.E-03	-7.2	146.0	4.E-03	0.8	146.0	2.E-02	19.3	147.3	1.E-02	29.7	147.7	2.E-02	22.2
148.1	3.8.E-02	17.3	148.5	1.E-02	-21.8	149.0	1.E-02	-6.5	147.7	1.E-02	14.0	148.2	1.E-02	8.5
150.5	2.6.E-03	-1.8	151.5	5.E-04	3.2	150.0	1.E-03	-6.9	151.0	3.E-03	-1.9	150.2	4.E-03	-8.2
150.6	1.2.E-03	5.3	152.4	1.E-02	-8.4	150.7	9.E-03	-4.1	151.7	2.E-02	-37.1	151.7	2.E-02	-38.3
151.5	1.4.E-02	-10.1	155.7	8.E-03	14.8	154.4	2.E-02	-4.7	152.9	1.E-02	-19.3	152.7	9.E-03	-17.6
156.0	1.4.E-02	-23.2	158.0	1.E-02	-9.6	155.1	4.E-03	12.0	157.7	1.E-02	25.9	158.2	1.E-02	33.7
160.2	7.4.E-03	13.5	158.6	9.E-04	-6.8	158.9	1.E-02	-8.0	159.5	1.E-03	-3.8	159.3	2.E-03	9.4
161.1	7.5.E-03	16.8	159.7	1.E-02	15.0	160.4	1.E-03	-7.9	160.3	3.E-03	-7.3	160.1	5.E-03	-32.9
163.9	1.7.E-02	-17.1	160.6	1.E-03	-6.7	161.9	1.E-02	-12.8	160.5	4.E-03	-15.5	162.6	3.E-04	5.9
164.2	2.2.E-02	-7.8	162.8	7.E-03	-14.6	164.4	8.E-03	-19.9	165.1	2.E-02	4.5	165.6	3.E-02	-4.5
166.1	1.3.E-02	-33.9	171.9	9.E-03	11.1	168.5	1.E-03	1.4	171.2	3.E-02	-6.2	171.5	3.E-03	0.4
173.8	2.7.E-02	1.6	181.4	7.E-02	-45.9	177.8	1.E-01	19.1	171.8	4.E-02	-13.9	172.2	5.E-02	-15.0
177.5	3.0.E-03	-2.1	183.0	9.E-03	-13.2	183.8	6.E-03	-4.8	178.6	8.E-03	-25.4	178.7	8.E-03	-25.7
192.6	2.4.E-01	21.9	189.1	2.E-01	45.6	193.1	2.E-01	12.0	195.3	2.E-01	34.4	195.3	2.E-01	33.5

Parameters used for ECD reproduction of **3.**

wavelength	20 nm
UV width	12 nm
UV intensity	800000
CD width	10 nm
CD intensity	1.7

SCF energy (au), chemical potential (kJ/mol). relative free energy, and Boltzmann distribution of *ent*-**4** based on B3LYP/def2-TZVP.

	M0001	M0002	M0003	M0004	M0005	M0006	M0007	M0008	M0009	M0010
SCF (AU)	-736.262356	-736.261964	-736.262126	-736.262087	-736.261596	-736.261584	-736.261771	-736.261863	-736.26132	-736.261217
CP (kJ/mol)	840.16	841.15	839.38	840.48	840.29	841.16	839.64	840.49	840.46	840.37
rel. ΔG (kJ/mol)	0.00	2.02	-0.18	1.03	2.12	3.02	1.01	1.62	3.02	3.20
Boltz. Dist.	12.6%	5.6%	13.6%	8.3%	5.4%	3.7%	8.4%	6.6%	3.7%	3.5%

	M0011	M0012	M0013	M0014	M0015	M0016	M0017	M0018	M0019
SCF (AU)	-736.260887	-736.261129	-736.260512	-736.261326	-736.260573	-736.260905	-736.260587	-736.26011	-736.260385
CP (kJ/mol)	839.63	838.83	840.43	839.1	839.67	840.08	838.9	839.76	839.98
rel. ΔG (kJ/mol)	3.32	1.89	5.11	1.64	4.19	3.73	3.38	5.49	4.99
Boltz. Dist.	3.3%	5.9%	1.6%	6.5%	2.3%	2.8%	3.2%	1.4%	1.7%

Wavelength (nm) oscillator strength (UV), and rotatory strength (CD) of *ent*-**4** based on B3LYP/def2-TZVP.

M0001			M0002			M0003			M0004			M0005		
λ	UV	CD	λ	UV	CD	λ	UV	CD	λ	UV	CD	λ	UV	CD
165.3	1.4.E-02	-37.4	165.7	4.3.E-03	-6.0	165.6	1.2.E-02	-6.9	165.1	2.3.E-04	-2.3	165.8	4.6.E-03	1.1
166.3	3.6.E-03	4.2	168.3	7.2.E-03	-4.9	165.8	8.6.E-03	-22.7	166.1	1.3.E-03	-6.9	168.7	7.7.E-03	-1.5
169.9	1.5.E-02	5.2	170.0	1.2.E-02	-4.8	170.2	1.6.E-02	8.8	170.0	1.2.E-02	1.4	169.9	5.8.E-03	-2.1
173.3	4.5.E-03	2.2	172.8	4.6.E-04	1.3	172.0	5.1.E-03	0.9	174.4	6.8.E-02	28.4	170.7	1.1.E-02	-4.5
174.2	1.1.E-03	-4.1	173.9	1.4.E-02	0.8	173.9	1.9.E-02	33.1	174.8	4.2.E-03	0.5	172.9	3.6.E-03	8.0
175.3	4.3.E-02	15.8	175.8	3.8.E-02	-16.5	174.4	3.8.E-02	7.7	175.8	3.2.E-03	7.4	176.1	3.6.E-02	-19.8
176.1	4.2.E-02	33.0	177.3	8.1.E-03	0.2	176.1	1.5.E-02	-5.0	176.6	4.2.E-03	5.5	178.2	5.1.E-02	36.1
178.0	4.9.E-02	51.7	179.1	8.6.E-02	108.3	180.2	4.0.E-02	49.1	178.9	5.2.E-02	31.4	181.6	3.3.E-02	47.2
181.0	5.6.E-03	7.5	180.8	4.8.E-03	5.4	182.0	5.9.E-04	5.6	180.0	4.5.E-03	0.4	182.2	3.3.E-03	6.6
183.9	1.4.E-02	3.1	184.2	1.5.E-03	-10.4	183.9	1.0.E-02	-0.4	183.3	3.5.E-02	30.9	184.6	3.8.E-03	-7.8
188.0	2.9.E-03	-8.2	188.7	1.7.E-02	-9.7	188.0	6.1.E-03	1.0	187.8	2.9.E-03	-9.6	188.3	1.6.E-02	17.3
193.4	5.1.E-02	1.9	192.6	7.9.E-02	35.5	190.8	1.1.E-01	22.0	196.7	1.6.E-02	-4.9	191.8	1.1.E-01	17.0
199.5	9.4.E-02	36.4	197.1	2.7.E-02	-13.5	200.3	4.9.E-02	27.1	199.1	9.6.E-02	44.3	195.9	3.4.E-02	-5.5
206.5	1.3.E-01	-81.6	202.7	6.1.E-02	-41.3	206.8	8.6.E-02	-72.5	206.6	1.5.E-01	-76.1	203.3	4.6.E-02	-32.7
207.3	9.6.E-03	10.4	206.8	9.7.E-02	-3.8	208.6	5.7.E-02	-2.7	208.4	4.4.E-03	1.4	209.2	8.7.E-02	-14.5

M0006			M0007			M0008			M0009			M0010		
λ	UV	CD	λ	UV	CD	λ	UV	CD	λ	UV	CD	λ	UV	CD
166.0	4.0.E-03	6.5	165.7	1.2.E-02	4.4	164.8	9.7.E-03	-11.6	165.8	2.8.E-03	4.1	165.6	5.5.E-03	2.0
170.0	2.2.E-02	-2.0	166.5	1.3.E-02	-24.4	165.4	9.0.E-03	-41.8	168.2	1.2.E-02	-1.3	170.5	2.6.E-02	1.3
171.0	4.7.E-03	-7.4	168.7	1.2.E-02	7.1	171.4	1.6.E-02	0.7	171.4	8.5.E-03	-3.8	171.5	2.7.E-03	0.2
173.0	3.4.E-04	0.2	173.2	3.1.E-02	33.7	173.5	2.5.E-03	-0.4	171.8	7.6.E-04	-1.3	172.1	2.2.E-03	-6.6
174.2	1.4.E-02	-5.2	173.9	1.3.E-03	-4.5	174.5	8.2.E-04	2.2	173.2	6.3.E-03	15.6	173.1	2.7.E-03	5.1
175.4	2.7.E-02	-2.8	174.0	6.2.E-04	-5.6	174.9	5.1.E-02	14.7	176.1	5.1.E-02	7.5	175.5	2.4.E-02	-21.1
177.7	4.6.E-02	69.5	176.9	4.0.E-02	7.5	178.3	7.2.E-02	73.9	177.6	6.0.E-03	-10.4	179.3	9.6.E-02	94.0
179.8	1.2.E-03	-9.5	180.3	3.6.E-02	43.5	180.6	1.4.E-02	6.3	181.8	4.7.E-02	62.5	181.3	2.2.E-04	-2.7
181.5	2.4.E-02	-1.7	183.9	1.1.E-02	0.8	181.1	1.8.E-03	-0.4	184.1	8.0.E-03	-5.5	182.8	1.0.E-02	16.0
184.5	3.2.E-03	-6.7	187.2	1.1.E-01	48.0	181.6	2.7.E-02	23.0	186.4	4.8.E-02	29.4	184.8	3.4.E-03	-8.1
187.6	8.3.E-02	22.3	188.5	1.2.E-02	-10.2	187.7	2.0.E-03	-6.9	188.3	6.0.E-03	-12.7	187.2	4.7.E-02	-6.7
192.9	3.5.E-02	32.6	188.7	1.4.E-02	-17.4	194.5	5.3.E-02	-4.3	190.8	1.1.E-01	-18.7	192.6	4.6.E-02	29.0
199.8	8.1.E-03	-7.6	201.8	4.6.E-02	20.9	200.0	4.8.E-02	33.9	196.1	1.3.E-02	17.0	198.0	3.7.E-02	-18.1
203.7	9.2.E-02	-56.9	206.2	4.1.E-02	-43.8	207.0	1.2.E-01	-85.4	202.4	2.8.E-02	-23.6	204.1	6.6.E-02	-37.5
207.2	9.1.E-02	6.2	212.1	9.7.E-02	-27.5	209.3	4.7.E-02	10.2	213.1	9.9.E-02	-24.5	210.0	9.0.E-02	-5.0

M0011			M0012			M0013			M0014			M0015		
λ	UV	CD	λ	UV	CD	λ	UV	CD	λ	UV	CD	λ	UV	CD
166.8	4.3.E-03	2.8	166.5	2.7.E-02	12.7	167.2	5.8.E-03	-7.0	165.3	5.1.E-03	-2.2	166.2	3.8.E-03	-2.2
167.2	2.5.E-03	0.6	167.5	3.0.E-03	-0.4	167.5	8.4.E-03	10.4	166.2	1.4.E-02	-36.4	168.5	3.0.E-02	20.4
167.4	1.1.E-02	5.7	169.7	3.4.E-03	-4.6	169.8	1.2.E-02	-2.6	169.4	1.1.E-02	4.8	168.8	7.1.E-03	2.4
170.4	1.0.E-02	4.2	170.1	3.2.E-02	18.1	175.5	9.9.E-03	1.1	173.5	5.0.E-02	33.9	170.7	1.9.E-02	1.8
176.4	7.2.E-02	45.2	172.1	4.5.E-02	7.1	175.8	2.4.E-02	-0.8	174.7	7.0.E-03	10.8	175.3	4.8.E-03	-5.8
177.0	3.5.E-03	3.3	175.7	8.7.E-03	-4.7	177.2	6.7.E-03	-3.5	176.6	2.3.E-02	-4.4	176.0	4.1.E-03	-12.6
177.6	4.8.E-03	-0.3	177.4	2.7.E-03	-4.5	177.8	2.2.E-02	4.2	177.3	2.9.E-04	0.4	179.3	6.5.E-02	32.0
179.1	4.9.E-04	3.7	180.6	8.8.E-04	12.1	178.6	2.7.E-02	33.7	178.8	1.3.E-03	-0.7	180.7	4.2.E-04	5.3
187.0	5.2.E-03	14.3	181.4	1.8.E-02	17.6	185.6	2.7.E-03	-8.4	180.4	5.8.E-02	57.8	183.1	2.3.E-03	3.6
187.8	3.0.E-03	-8.3	187.7	1.4.E-03	-6.9	187.1	2.2.E-03	-7.2	187.4	2.8.E-03	10.6	185.6	4.3.E-03	-13.8
189.7	1.3.E-02	10.9	188.9	6.9.E-03	20.0	188.5	1.9.E-02	29.0	188.2	1.5.E-03	-3.1	190.8	4.3.E-02	59.2
192.3	9.9.E-02	-3.0	194.6	1.4.E-01	24.3	192.9	1.1.E-01	21.6	192.5	5.8.E-02	-18.4	191.8	4.8.E-02	3.0
200.9	7.3.E-02	30.1	200.2	2.2.E-02	24.7	197.0	2.0.E-02	-3.5	200.6	9.4.E-02	36.6	198.9	8.9.E-02	-26.8
207.1	1.1.E-01	-78.7	206.6	1.3.E-01	-83.3	203.2	1.2.E-01	-57.9	206.5	7.7.E-02	-72.1	204.1	6.4.E-02	-34.0
215.3	2.8.E-02	20.7	209.2	3.1.E-02	14.9	215.7	4.0.E-02	18.3	211.6	6.6.E-02	18.0	210.1	6.7.E-02	8.4

M0016			M0017			M0018			M0019		
λ	UV	CD	λ	UV	CD	λ	UV	CD	λ	UV	CD
165.5	4.1.E-03	-5.1	166.9	1.1.E-02	-0.8	166.0	2.5.E-03	10.6	165.5	1.1.E-02	0.0
168.5	2.0.E-02	0.8	167.6	5.7.E-03	-1.1	167.2	9.0.E-03	8.9	166.4	3.4.E-04	0.0
171.4	9.8.E-03	-9.2	167.9	1.5.E-03	1.3	168.6	3.1.E-02	12.2	170.3	2.3.E-02	0.0
173.6	8.1.E-03	11.2	171.2	3.2.E-02	13.7	169.2	4.3.E-03	3.8	172.0	2.5.E-03	0.0
174.8	5.2.E-02	30.6	171.3	9.6.E-03	6.0	173.0	1.7.E-03	6.0	174.7	5.9.E-03	0.0
175.3	5.2.E-03	-9.9	174.1	5.4.E-05	0.1	177.3	2.7.E-02	-2.2	175.0	7.6.E-03	0.0
177.3	1.2.E-03	-6.2	177.8	3.0.E-02	5.4	178.2	2.0.E-03	-0.7	176.1	7.5.E-02	0.1
180.4	7.1.E-03	14.0	180.1	3.5.E-02	20.0	180.8	4.4.E-02	21.4	180.3	1.8.E-02	0.0
183.9	4.0.E-02	29.7	186.0	1.1.E-01	52.1	185.0	2.4.E-03	-8.1	181.3	5.3.E-02	0.1
184.8	3.6.E-02	0.5	187.4	2.6.E-02	-1.7	186.6	1.3.E-01	63.6	182.6	1.6.E-02	0.0
186.7	4.4.E-03	13.7	188.6	1.1.E-02	-9.5	189.1	2.6.E-03	12.3	183.4	1.4.E-02	0.0
193.2	9.0.E-02	12.6	189.6	2.1.E-03	8.2	191.1	5.0.E-02	-38.8	192.9	6.3.E-02	0.1
196.6	1.1.E-02	-0.1	202.2	5.2.E-02	20.0	196.7	1.3.E-02	21.9	195.8	5.1.E-03	0.0
202.4	7.6.E-02	-54.0	205.7	2.4.E-02	-30.7	202.4	2.1.E-02	-20.6	202.8	1.2.E-01	0.1
211.7	9.3.E-02	15.8	215.1	1.3.E-01	-33.5	215.9	1.3.E-01	-26.0	205.3	5.0.E-02	0.1

Parameters used for ECD reproduction of *ent*-**4.**

wavelength	0 nm
UV width	8 nm
UV intensity	4000
CD width	6 nm
CD intensity	-0.2 (because the enantiomer was applied for the calculations)

SCF energy (au), chemical potential (kJ/mol). relative free energy, and Boltzmann distribution of *ent*-**5** based on B3LYP/def2-TZVP.

	M0001	M0002	M0003	M0004	M0005	M0006	M0007	M0008	M0009	M0010
SCF (au)	-736.2551045	-736.254876	-736.2548473	-736.2546731	-736.2545079	-736.2545639	-736.2544778	-736.2542072	-736.2543734	-736.254286
CP (kJ/mol)	840.06	839.74	838.66	840.08	839.61	838.9	838.33	838.06	838.93	838.52
rel G (kJ/mol)	0.72	1.00	0.00	1.88	1.84	0.98	0.64	1.08	1.51	1.33
Boltz. Dist.	12.4%	8.3%	35.0%	2.4%	2.5%	8.6%	14.0%	7.5%	4.0%	5.2%

Wavelength (nm) oscillator strength (UV), and rotatory strength (CD) of *ent*-**5** based on B3LYP/def2-TZVP.

M0001			M0002			M0003			M0004			M0005		
λ	UV	CD	λ	UV	CD	λ	UV	CD	λ	UV	CD	λ	UV	CD
176.1	9.0.E-03	9.0	176.0	9.1.E-03	7.4	178.1	2.2.E-03	-8.7	176.2	9.0.E-03	11.3	176.0	8.7.E-03	8.0
179.4	1.4.E-02	-1.7	180.0	1.2.E-02	-1.9	179.1	2.2.E-02	2.3	176.3	3.1.E-03	-3.1	179.3	4.8.E-03	0.6
180.4	1.4.E-03	6.9	181.6	2.6.E-04	3.1	180.4	1.9.E-03	7.2	179.9	1.1.E-03	-8.3	185.5	1.1.E-03	-2.6
184.3	2.0.E-04	1.1	185.3	6.5.E-03	13.0	184.3	4.2.E-03	0.2	184.3	6.8.E-03	11.5	186.7	4.7.E-03	12.7
185.2	7.8.E-03	14.0	187.0	1.1.E-02	13.1	185.4	2.4.E-02	37.0	188.3	2.8.E-03	-4.9	187.5	1.2.E-02	-3.3
189.4	7.7.E-02	87.6	190.1	2.9.E-02	46.5	189.8	1.7.E-02	29.6	190.5	1.1.E-01	94.5	190.2	1.6.E-02	31.3
199.4	6.6.E-02	4.4	197.4	1.6.E-01	35.3	197.5	1.3.E-01	58.7	199.5	9.0.E-03	0.0	194.7	2.2.E-01	52.8
203.4	6.5.E-02	-32.2	204.3	3.3.E-02	-4.2	204.8	1.6.E-01	-65.3	203.2	1.4.E-01	-36.1	202.9	3.1.E-02	-12.2
205.3	1.3.E-01	-57.2	204.9	9.8.E-02	-68.8	208.1	1.7.E-02	-21.6	208.9	8.9.E-02	-56.7	204.7	5.0.E-02	-43.2
216.0	9.8.E-02	20.0	217.7	1.1.E-01	3.4	219.6	8.9.E-02	0.8	216.5	9.2.E-02	26.2	221.6	1.3.E-01	-11.7

M0006			M0007			M0008			M0009			M0010		
λ	UV	CD	λ	UV	CD	λ	UV	CD	λ	UV	CD	λ	UV	CD
176.5	9.7.E-03	7.0	178.2	2.6.E-03	-11.5	176.9	8.9.E-03	8.7	176.3	6.5.E-03	-3.8	176.9	1.1.E-02	-16.5
177.0	2.3.E-03	3.5	179.9	1.9.E-02	2.2	180.1	9.1.E-04	-2.9	177.8	1.9.E-03	-10.3	180.1	2.2.E-02	-0.7
180.8	4.3.E-04	0.4	181.9	3.8.E-04	3.0	181.4	1.5.E-03	0.6	179.9	1.0.E-03	-7.7	180.5	9.1.E-03	9.7
184.4	6.9.E-03	9.5	185.9	2.2.E-02	34.8	184.2	1.1.E-02	25.3	185.1	2.5.E-02	37.8	181.7	3.3.E-03	3.2
190.3	1.5.E-02	-5.7	187.5	6.1.E-03	3.4	185.4	4.2.E-02	33.9	188.5	1.5.E-02	-8.2	183.0	6.2.E-03	11.0
190.7	1.1.E-01	103.8	188.8	2.7.E-03	3.9	195.5	2.2.E-02	-4.1	191.3	6.6.E-02	56.7	190.3	6.0.E-02	70.0
198.4	1.6.E-03	4.0	198.1	2.0.E-01	87.1	199.5	1.3.E-01	49.2	196.9	2.0.E-02	21.7	198.9	5.1.E-02	19.5
203.7	1.8.E-01	-75.6	205.3	1.1.E-01	-76.3	203.6	1.6.E-01	-97.5	203.8	2.3.E-01	-37.9	204.5	2.2.E-01	-89.6
207.2	2.6.E-02	-13.7	206.8	1.9.E-02	-3.1	207.3	1.7.E-02	2.5	211.9	1.6.E-02	-28.7	208.1	1.8.E-03	6.1
218.0	1.1.E-01	9.7	222.0	8.7.E-02	-10.8	218.8	8.4.E-02	24.4	220.6	8.9.E-02	-0.9	217.6	8.8.E-02	10.3

Parameters used for ECD reproduction of *ent*-**5.**

wavelength	0 nm
UV width	12 nm
UV intensity	10000
CD width	10 nm
CD intensity	-3.2 (because the enantiomer was applied for the calculations)

SCF energy (au), chemical potential (kJ/mol). relative free energy, and Boltzmann distribution of **8-OBz** based on B3LYP/def2-TZVP.

	M0001	M0002	M0003	M0004	M0005	M0006
SCF (au)	-1081.801	-1081.801	-1081.799	-1081.799	-1081.798	-1081.798
CP (kJ/mol)	1113.44	1114.89	1113.65	1112.42	1112.2	1112.94
rel G (kJ/mol)	0	1.71	3.90	3.82	4.77	6.30
Boltzmann dist	46.7%	23.3%	9.6%	9.9%	6.8%	3.7%

Wavelength (nm) oscillator strength (UV), and rotatory strength (CD) of **8-OBz** based on B3LYP/def2-TZVP.

M0001			M0002			M0003			M0004			M0005		
λ	UV	CD	λ	UV	CD	λ	UV	CD	λ	UV	CD	λ	UV	CD
176.1	3.1.E-03	4.3	177.5	1.3.E-03	-0.8	176.0	5.6.E-04	-0.1	175.7	3.3.E-04	1.0	175.8	2.4.E-03	1.2
176.3	6.6.E-03	-15.6	180.0	4.8.E-04	-0.2	178.0	2.2.E-03	-8.9	175.8	5.5.E-03	-9.3	177.2	8.2.E-04	-0.6
179.7	7.8.E-04	-1.0	181.2	8.7.E-03	1.6	179.5	4.1.E-04	-0.7	179.3	1.3.E-03	-1.2	179.5	3.0.E-03	0.9
183.9	5.8.E-03	16.8	182.5	3.9.E-03	-1.6	184.2	3.3.E-03	-1.5	184.2	1.9.E-02	-8.5	180.4	9.6.E-04	-0.4
183.9	5.3.E-03	-7.5	184.5	2.2.E-02	0.6	185.3	5.4.E-02	-7.2	184.6	1.9.E-01	0.9	185.5	1.4.E-01	36.0
185.1	3.4.E-01	11.4	185.5	7.7.E-02	-15.6	185.6	5.5.E-01	3.2	186.3	4.5.E-02	32.0	185.7	4.3.E-01	-43.0
186.9	3.9.E-01	-10.3	185.9	6.3.E-01	5.3	187.6	1.5.E-02	10.2	186.7	4.9.E-01	-20.2	187.7	1.7.E-01	-6.4
188.9	3.2.E-02	-12.5	190.0	1.7.E-01	-26.2	188.2	1.2.E-01	-7.4	187.4	1.4.E-03	-1.1	188.5	3.8.E-03	5.9
190.4	9.1.E-02	-19.7	190.6	1.1.E-02	15.0	189.6	6.6.E-02	-4.2	190.3	1.8.E-01	-14.1	190.2	1.4.E-01	-27.1
190.5	7.2.E-02	21.5	191.4	5.3.E-02	-10.6	189.9	9.8.E-04	4.8	191.0	5.8.E-03	6.7	190.9	3.0.E-02	13.0
192.0	3.2.E-02	-7.8	192.9	1.6.E-03	0.2	191.1	1.6.E-01	-19.1	192.0	4.2.E-02	-7.8	191.7	5.5.E-02	-5.4
197.7	1.3.E-02	2.6	197.9	2.2.E-02	1.7	197.4	1.3.E-02	2.3	198.0	9.9.E-03	2.7	197.2	1.0.E-02	2.1
201.1	1.1.E-02	5.6	201.9	2.1.E-03	-1.4	201.5	3.1.E-03	-1.2	201.2	1.1.E-02	5.8	201.8	4.2.E-03	-1.7
202.7	3.6.E-03	-1.5	202.8	1.3.E-02	5.8	201.6	1.1.E-02	5.5	202.6	3.5.E-03	-1.5	203.1	1.3.E-02	4.8
216.2	2.3.E-03	-1.0	214.1	2.7.E-04	-0.2	219.5	2.4.E-03	-0.5	215.0	3.2.E-03	-0.5	218.8	1.9.E-03	0.8
226.3	1.6.E-01	-7.0	225.7	2.9.E-02	-3.7	224.1	2.2.E-02	-5.9	226.0	1.3.E-01	-8.6	226.2	8.5.E-02	-11.5
227.6	1.1.E-01	16.6	226.2	2.2.E-01	8.5	227.3	2.5.E-01	15.7	227.8	1.4.E-01	19.0	227.6	1.8.E-01	21.8
238.2	4.9.E-02	3.0	236.0	7.1.E-02	7.0	236.4	4.9.E-02	3.3	238.0	4.6.E-02	1.6	236.7	5.4.E-02	1.0
248.9	1.2.E-02	-0.6	248.6	1.2.E-02	0.4	249.0	1.2.E-02	-0.1	248.9	1.2.E-02	-0.6	249.1	1.2.E-02	-1.3
255.4	3.4.E-05	-0.5	256.0	1.1.E-04	-1.2	255.1	1.6.E-04	-1.5	255.3	1.6.E-05	-0.3	254.8	1.2.E-05	0.1

M0006

λ	UV	CD
176.6	8.4.E-03	3.1
177.7	1.2.E-03	-0.8
179.9	1.5.E-04	-0.5
182.0	7.4.E-03	1.8
183.0	5.1.E-03	3.6
185.8	6.6.E-01	-2.8
187.8	6.2.E-02	-3.4
189.5	2.4.E-02	-12.1
190.3	1.5.E-01	2.8
191.3	5.4.E-02	-12.5
193.4	1.7.E-03	1.2
196.8	2.7.E-02	1.5
201.4	1.1.E-03	-1.3
203.3	1.3.E-02	5.8
218.3	7.1.E-04	-0.1
225.5	1.0.E-03	0.1
226.1	2.3.E-01	4.7
234.3	9.0.E-02	8.1
248.7	1.2.E-02	-0.2
255.5	2.1.E-04	-1.7

Parameters used for ECD reproduction of **8-OBz.**

wavelength	4 nm
UV width	10 nm
UV intensity	35000
CD width	6 nm
CD intensity	+3.0

Wavelength (nm) and rotatory strength (CD) of *ent*-model **I**, *ent*-model **II**, and *ent*-model **III** based on B3LYP/def2-TZVP, and their calculated ECD spectra

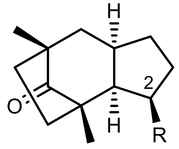

ent-model **I** (R = H)
ent-model **II** (R = Cl)

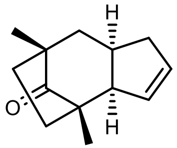

ent-model **III**

ent-model **I**		*ent*-model **II**		*ent*-model **III**	
λ	CD	λ	CD	λ	CD
157.5	1.6	166.6	-0.1	170.4	-5.0
159.1	2.1	168.0	11.2	171.0	0.0
159.6	-1.4	170.0	9.3	171.1	0.6
161.8	-3.0	173.2	-18.4	172.1	-3.2
166.5	7.2	174.4	-10.4	175.5	-0.6
170.0	-1.0	179.1	33.1	176.4	18.9
171.6	1.0	180.2	-45.6	177.2	-7.8
175.6	-3.0	181.8	-11.7	177.8	-7.1
176.9	-4.7	182.6	-3.0	179.4	-4.0
177.1	2.4	184.9	-1.0	185.7	-5.4
180.3	3.2	187.9	13.7	189.1	-1.0
185.1	-14.6	198.7	-26.4	194.1	3.6
185.5	3.5	209.7	37.8	197.6	2.8
199.7	2.4	233.3	7.2	209.1	2.1
295.0	0.8	301.7	-19.9	292.7	5.2

Parameters used for ECD reproduction of *ent*-model **I**, *ent*-model **II**, and *ent*-model **III.**

wavelength	4 nm
CD width	6 nm
intensity	-1

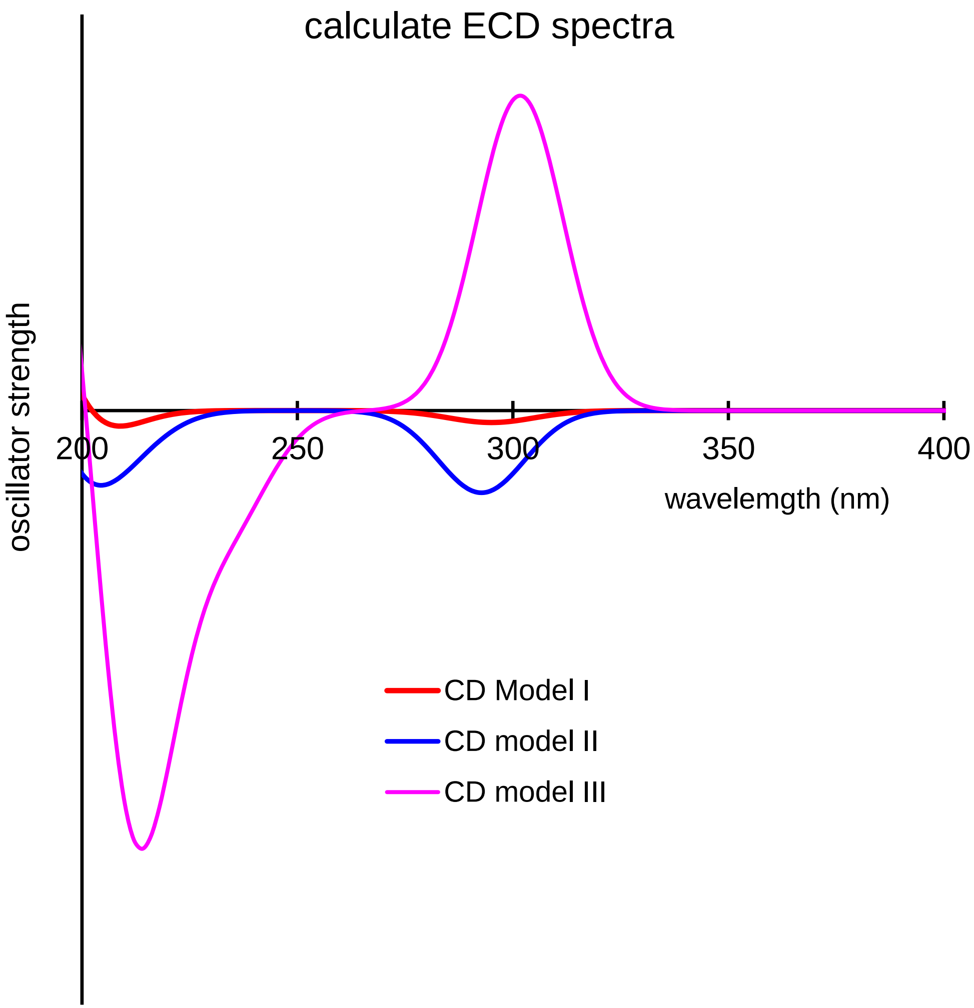

XYZ data of *ent*-model **I** used for reproduction of the ECD spectrum.

34			
C	1.811005	-2.492120	0.227582
C	-0.613461	1.566304	-1.505766
C	-0.544431	-1.543690	0.695973
C	-1.292361	-0.290834	0.144592
C	1.451351	1.762160	2.981635
C	1.525762	-0.479737	1.746819
C	1.438668	2.542456	0.638777
C	0.675792	0.744418	2.131633
C	0.250372	1.579028	0.898430
C	0.959715	-0.235404	-0.607775
C	2.242089	2.611414	1.964662
C	0.967608	-1.261576	0.525516
C	-0.202946	0.711515	-0.314404
O	1.726224	-0.179703	-1.537504
H	2.093523	1.288110	3.727435
H	-0.207308	0.394307	2.670993
H	-0.620637	2.178086	1.188481
H	1.079985	3.525397	0.331678
H	-0.817559	-2.430025	0.120525
H	-0.797740	-1.752967	1.736760
H	-1.955525	0.156780	0.887031
H	-1.914312	-0.568669	-0.708488
H	2.550747	-0.173282	1.514135
H	1.593892	-1.161433	2.600991
H	-0.934439	0.940415	-2.340564
H	0.214851	2.180694	-1.858900
H	-1.442415	2.226770	-1.238342
H	2.069153	2.176466	-0.173116
H	2.851789	-2.217703	0.047944
H	1.449819	-3.009790	-0.663042
H	1.780402	-3.193177	1.065365
H	0.741981	2.392676	3.524964
H	3.243992	2.202783	1.821147
H	2.374519	3.635983	2.314616

XYZ data of *ent*-model **II** used for reproduction of the ECD spectrum.

40			
H	1.755916	0.706760	2.357815
C	1.645872	0.314147	1.345015
C	0.199722	-0.171098	1.054026
C	-0.229464	0.586378	-0.237766
C	1.085888	1.008769	-0.906863
C	1.997182	1.395879	0.289534
H	2.341201	-0.522555	1.253828
H	-0.461075	0.139781	1.865506
H	-0.665244	1.540182	0.076747
H	0.960068	1.825292	-1.614263
C	0.099066	-1.702749	0.930283
H	0.121222	-2.149475	1.930148
C	-1.366893	-0.093697	-1.049668
C	-1.096761	-1.596017	-1.213736
C	-1.171015	-2.195595	0.192569
C	-2.611396	-0.191515	-0.118273
H	-3.509148	-0.262266	-0.735015
H	-2.719262	0.701892	0.499487
C	-2.441755	-1.489173	0.730545
H	-3.307216	-2.140636	0.601942
H	-2.360290	-1.278119	1.798194
C	3.493618	1.437147	-0.030376
H	4.053747	1.717654	0.865008
H	3.713803	2.176769	-0.804230
H	3.861432	0.472180	-0.373099
C	1.586441	2.798802	0.788725
H	2.222547	3.087839	1.627864
H	1.714946	3.548067	0.003464
C	-1.651524	0.618922	-2.364348
H	-2.491699	0.150809	-2.878938
H	-0.793732	0.570458	-3.034960
H	-1.902310	1.669268	-2.191959
C	-1.251111	-3.714729	0.195002
H	-0.350886	-4.154390	-0.237669
H	-2.101046	-4.063326	-0.393931
H	-1.361036	-4.092956	1.214532
O	-0.994939	-2.201420	-2.248488
H	0.553417	2.845193	1.134218
H	0.971851	-2.087607	0.396360
Cl	1.813376	-0.332501	-1.913615

XYZ data of *ent*-model **III** used for reproduction of the ECD spectrum.

32			
C	1.406067	-2.920837	0.421288
C	-0.100478	1.386984	-1.713094
C	-0.680603	-1.446004	0.784993
C	-1.157802	-0.130221	0.096364
C	2.006540	1.621528	2.718527
C	1.587453	-0.775067	1.762369
C	1.164205	2.638862	0.722792
C	1.052943	0.648465	1.989352
C	0.767359	1.367740	0.681402
C	1.055335	-0.623226	-0.626119
C	1.763653	3.000278	2.059540
C	0.856834	-1.513437	0.604521
C	0.116486	0.586778	-0.439349
O	1.785434	-0.827325	-1.560302
H	3.039179	1.312509	2.536860
H	0.113555	0.571420	2.552954
H	-1.142623	-2.310649	0.305431
H	-0.959901	-1.483624	1.839203
H	-1.719824	0.514198	0.772843
H	-1.812570	-0.359951	-0.746094
H	2.653613	-0.728849	1.517760
H	1.495427	-1.372550	2.675023
H	-0.523270	0.753790	-2.494600
H	0.841592	1.787366	-2.088535
H	-0.786487	2.218227	-1.537986
H	2.479785	-2.898507	0.228477
H	0.930674	-3.421185	-0.424351
H	1.230304	-3.522446	1.316432
H	1.855989	1.633557	3.798883
H	2.681807	3.586372	1.968270
H	1.065560	3.611871	2.644632
H	1.050251	3.356940	-0.078810

Wavelength (nm) oscillator strength (UV), and rotatory strength (CD) of model **IV** based on B3LYP/def2-TZVP when dihedral angle ∠O/C-5/C-9/C-7 was set to15°.

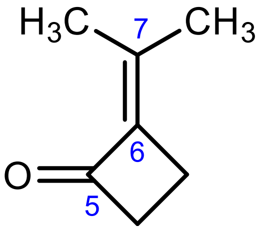

model **IV**

λ	UV	CD
134.8	9.8.E-03	21.6
135.8	5.0.E-02	-15.2
136.1	1.4.E-02	-5.1
136.9	6.0.E-03	-2.0
138.5	5.5.E-03	15.4
139.9	1.5.E-01	-0.9
140.0	1.9.E-02	0.4
141.9	2.0.E-02	-1.8
145.3	2.1.E-02	-9.2
146.3	8.3.E-02	9.9
146.5	3.7.E-02	7.7
148.3	7.4.E-02	-19.4
148.7	7.4.E-03	-17.5
149.0	3.6.E-02	17.7
152.8	1.1.E-01	4.4
153.1	3.7.E-03	-3.7
154.6	8.1.E-03	0.8
158.4	1.3.E-04	1.3
164.3	7.7.E-04	-0.1
165.5	2.7.E-02	-7.8
166.0	9.4.E-03	0.3
167.4	4.3.E-04	5.4
171.3	2.0.E-03	10.3
174.1	1.5.E-03	-18.0
181.5	8.3.E-03	2.2
183.5	3.3.E-03	1.5
185.8	3.5.E-02	-1.6
194.5	1.2.E-03	-7.7
237.2	3.5.E-01	9.5
348.4	5.8.E-04	-5.3

Parameters used for ECD reproduction of model **V.**

wavelength	20 nm
UV width	11 nm
UV intensity	30000
CD width	10 nm
CD intensity	+2.0

XYZ data of model **V** used for reproduction of the ECD spectrum.

C	-1.0314	1.2228	-0.8196
C	-0.356	0.096	-0.1109
C	-2.3385	0.9708	-0.0474
C	-1.6525	-0.2665	0.6126
H	-2.0871	-1.2203	0.3083
H	-1.6057	-0.2384	1.7021
O	-0.7283	1.9886	-1.6986
C	0.9194	-0.3042	-0.0283
C	2.0009	0.349	-0.8379
H	2.7889	0.7309	-0.1806
H	1.6174	1.1647	-1.4466
H	2.4763	-0.3864	-1.4957
C	1.3411	-1.4274	0.8714
H	2.0979	-1.0839	1.5842
H	1.8071	-2.2289	0.289
H	0.5062	-1.849	1.4293
H	-3.2017	0.7689	-0.6831
H	-2.5899	1.7769	0.6436

Wavelength (nm) and rotatory strength (CD) of model **V** based on B3LYP/def2-TZVP when dihedral angle ∠C-8/C-9/C-10/O was set to180°.

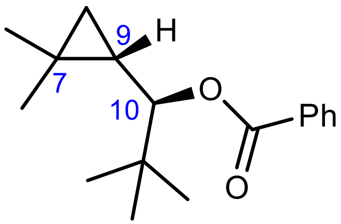

model **V**

λ	CD
162.52	0.47
164.96	-2.58
169.72	-0.26
173.42	-0.31
178.45	-4.99
178.69	4.03
181.43	4.03
184.49	7.29
188.16	-2.09
197.38	0.84
204.85	0.10
206.42	-0.59
223.00	15.85
245.63	1.38
255.28	-1.96

Parameters used for ECD reproduction of model **V.**

wavelength	14 nm
CD width	8.0 nm
CD intensity	+2.8

XYZ data of model **V** used for reproduction of the ECD spectrum.

C	-2.9578	1.3481	2.639
C	-3.1552	0.9629	1.1929
H	-3.4814	-0.0481	0.9687
H	-3.5249	1.7173	0.5055
C	-1.7699	1.1253	1.7416
H	-1.2172	2.0149	1.4473
C	-0.8807	-0.0594	2.0181
H	-1.4413	-0.9981	1.9911
O	0.2131	-0.1155	1.0914
C	-0.0251	-0.7463	-0.0727
C	1.1411	-0.6781	-0.9985
C	3.2552	-0.5907	-2.806
C	1.0295	-1.3146	-2.2361
C	2.3141	0.0033	-0.6671
C	3.3685	0.0455	-1.5721
C	2.0858	-1.2715	-3.1373
H	0.1082	-1.8361	-2.4734
H	2.3944	0.4955	0.2953
H	4.28	0.5764	-1.3151
H	1.9973	-1.7672	-4.0991
H	4.0805	-0.5558	-3.5112
O	-1.0651	-1.3091	-0.3327
H	-3.1843	2.364	2.9468
H	-3.1722	0.5944	3.3929
H	-0.3924	0.0428	2.9907

Calculated and experimental UV/ECD spectra of **3** with B3LYP/def2-TZVP

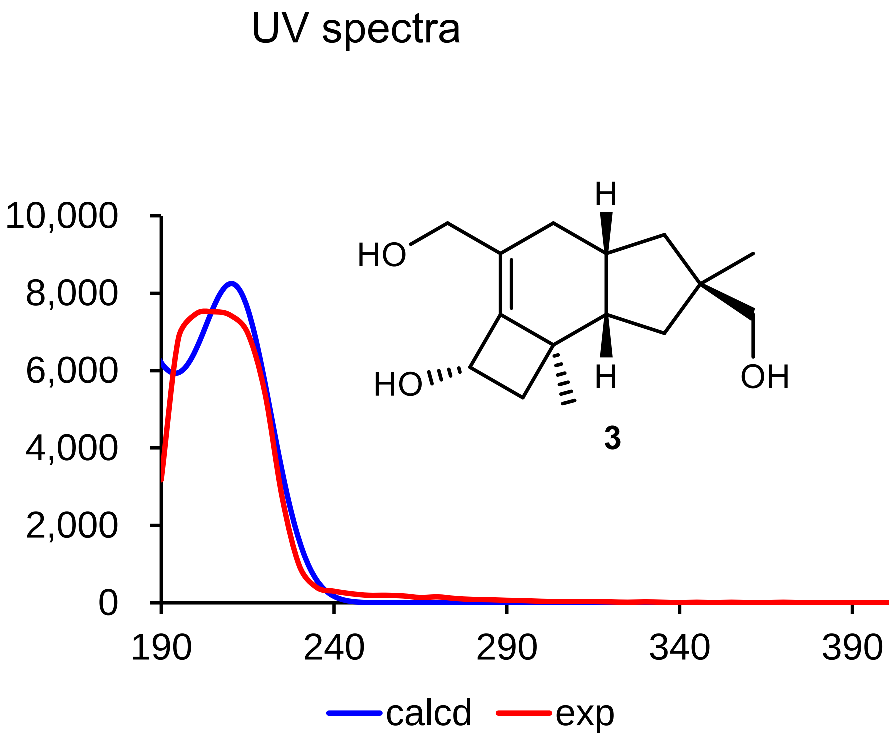

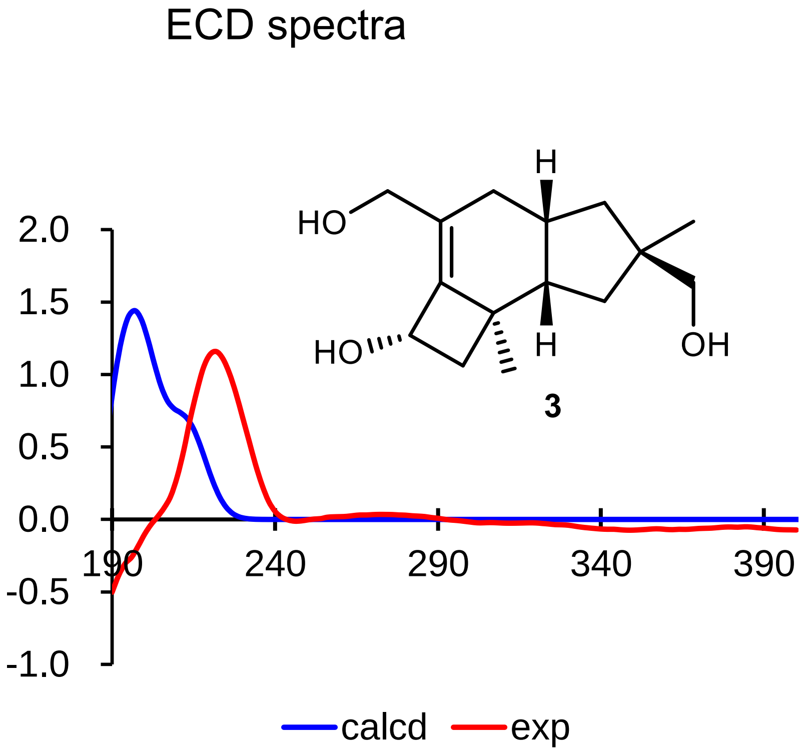

Hyphal growth inhibitions of *Cochliobolus miyabeanus* by **2a** and **5** at 5.0 μg/mL

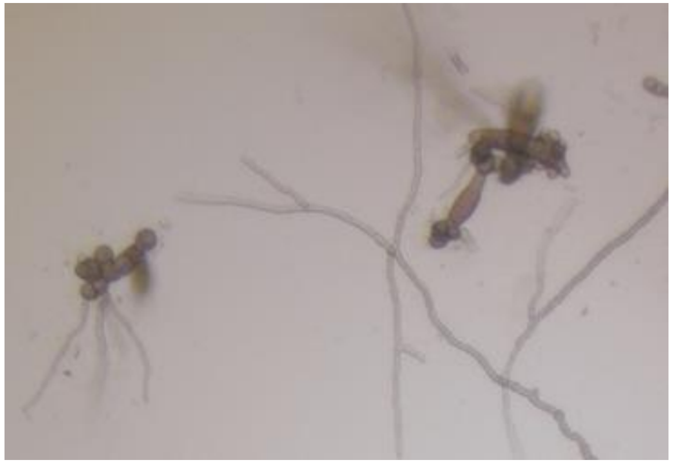

2a (5.0 μg/mL)

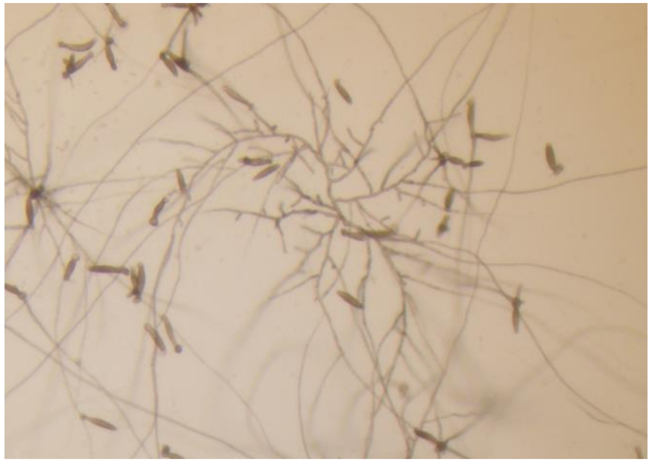

5 (5.0 μg/mL)
